# Supplementary material for: Nanoscale Material Heterogeneity of Glowworm Capture Threads Revealed by AFM
Source: Molecules. 2021 Jun 8;26(12):3500. doi: 10.3390/molecules26123500 (PMC8226719; doi:10.3390/molecules26123500)
Supplement: Supplementary file 1 [file molecules-26-03500-s001.zip › Glowworm AFM MS ESM Figures S1-S8.pdf]

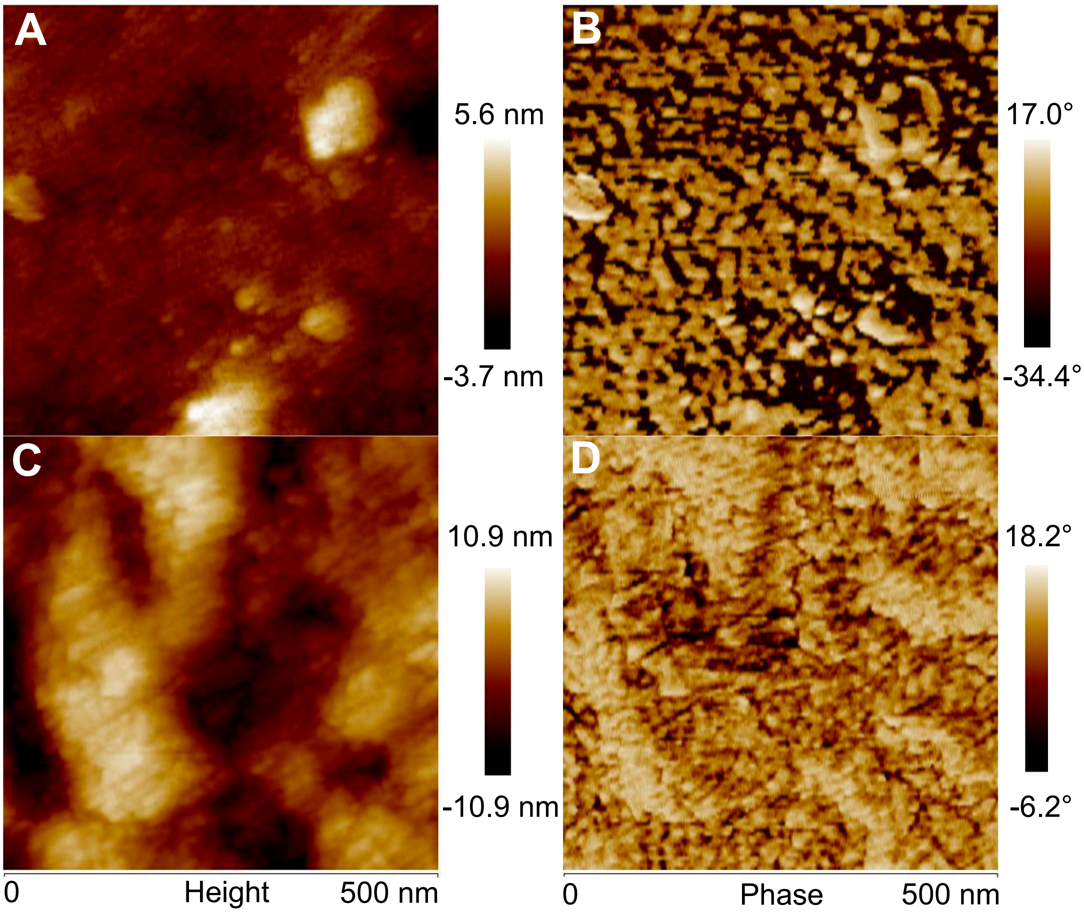

Figure S1. Height and phase imaging of a glass slide surface (A, B) and *Arachnocampa tasmaniensis* glue droplet dispersed on a glass slide (C, D) away from the silk fiber.

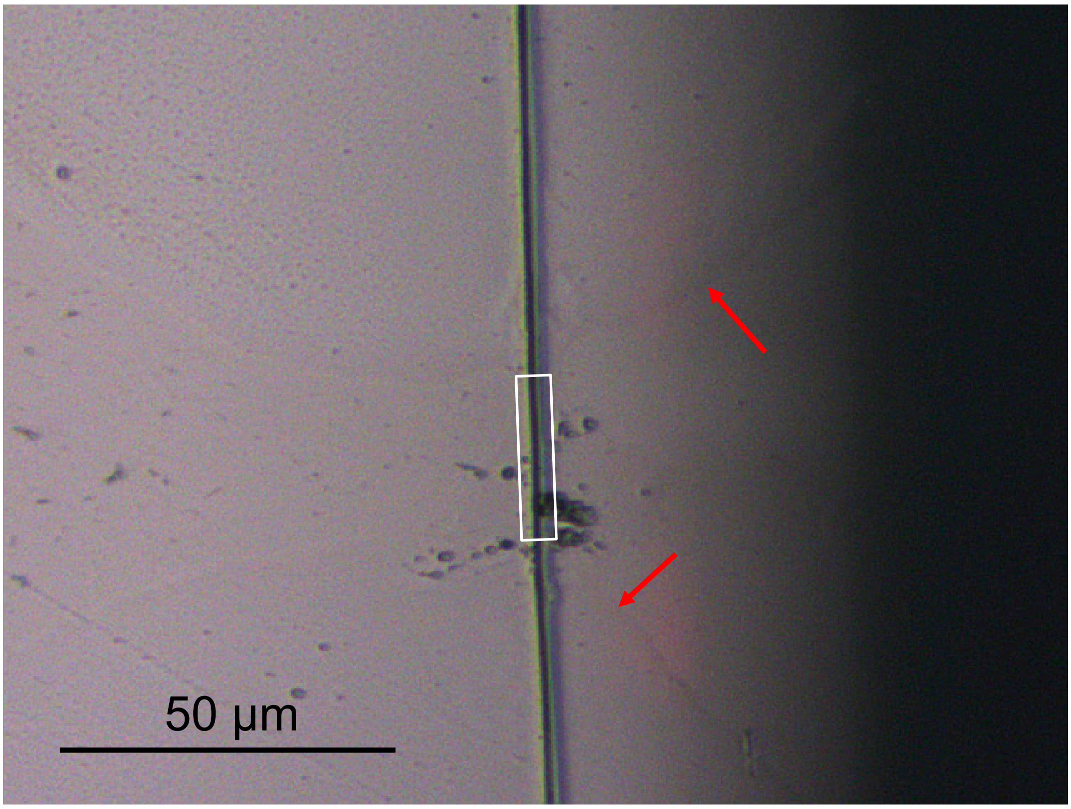

Figure S2. *Arachnocampa tasmaniensis* silk thread mounted to a glass microscope slide. Red arrows indicate dispersed glue droplets and white box represents the region of the silk fiber where AFM imaging was conducted.

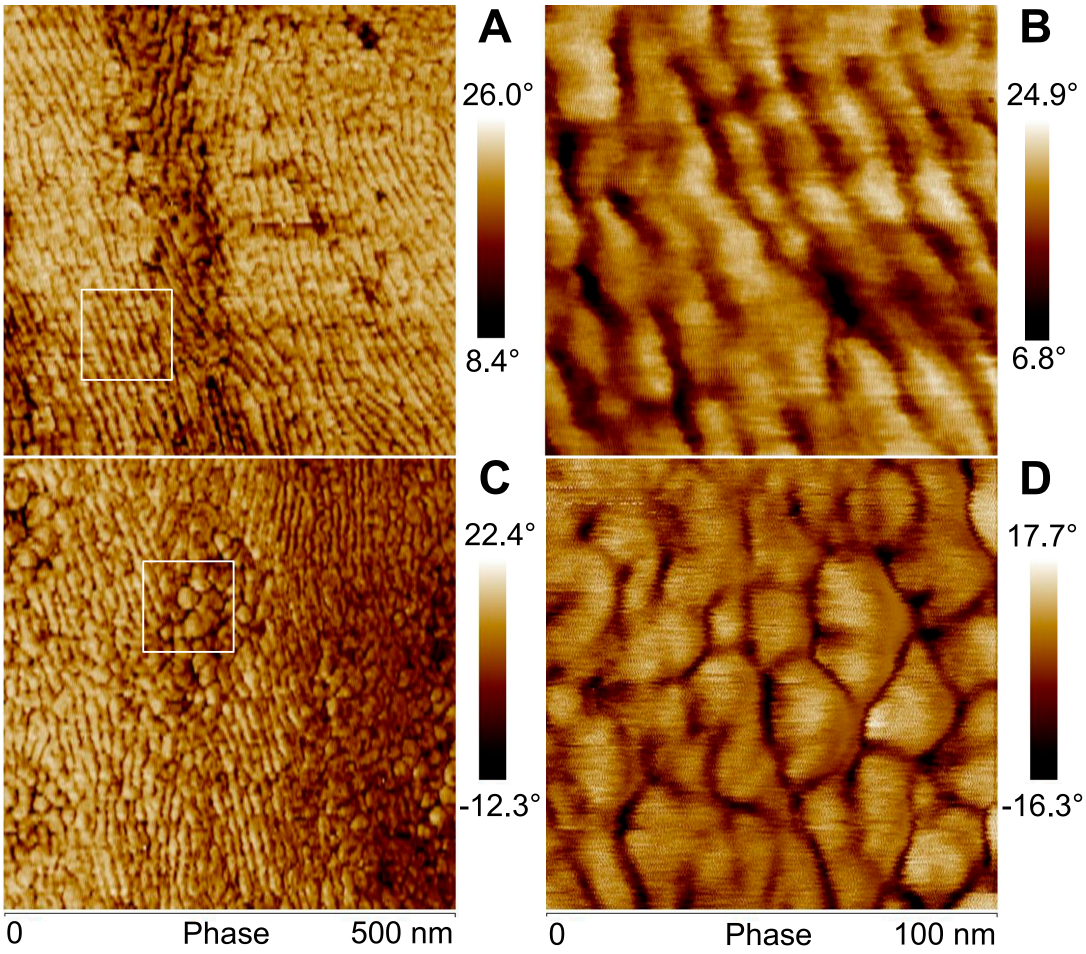

Figure S3. Phase images of surfaces of *Arachnocampa tasmaniensis* glowworm silk fibers at 500 nm (A, C) and 100 nm (B, D) scales. Images taken from two fibers from two glowworm individuals. Inset white squares in A and C represent the regions where 100 nm scale imaging was conducted in B and D, respectively.

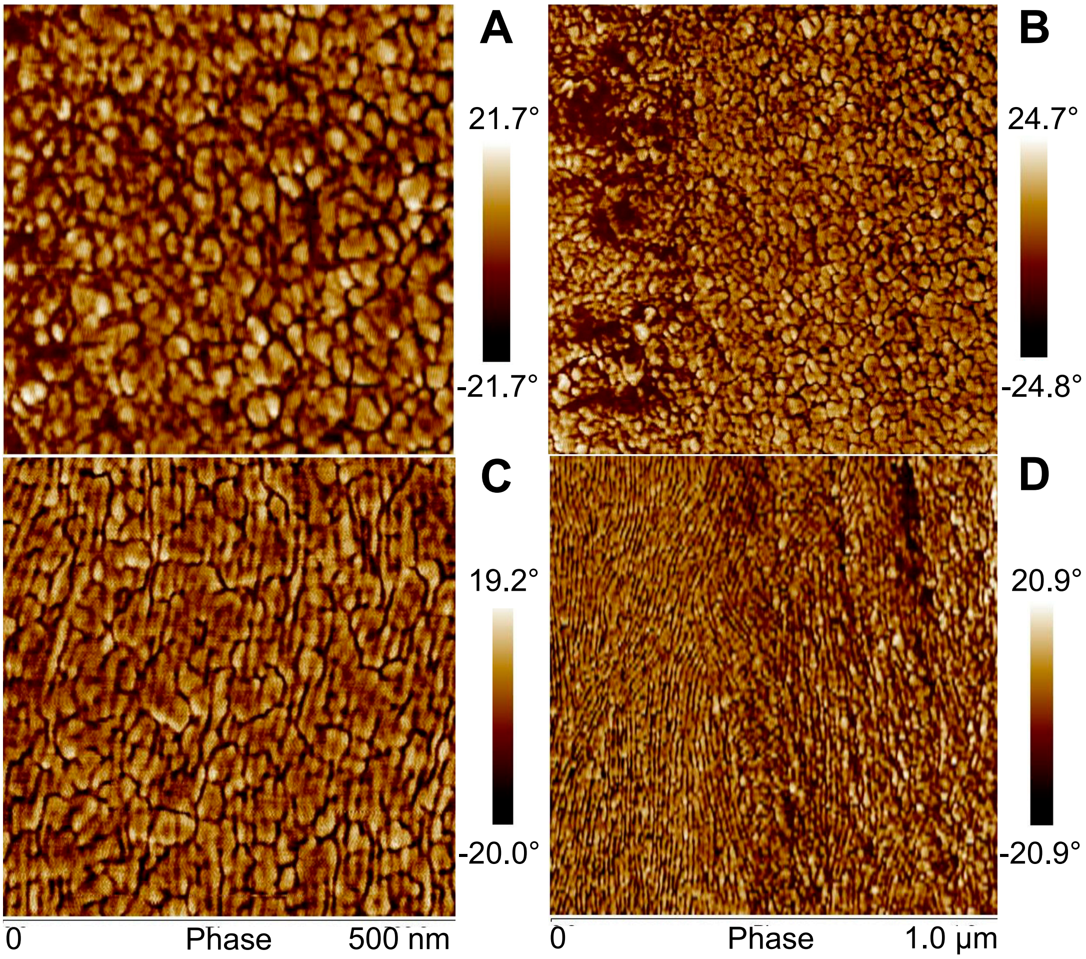

Figure S4. Phase images of surfaces of *Arachnocampa tasmaniensis* glowworm silk fibers at 500 nm (A, C) and 1.0 µm (B, D) scales. Images taken from four fibers from two glowworm individuals.

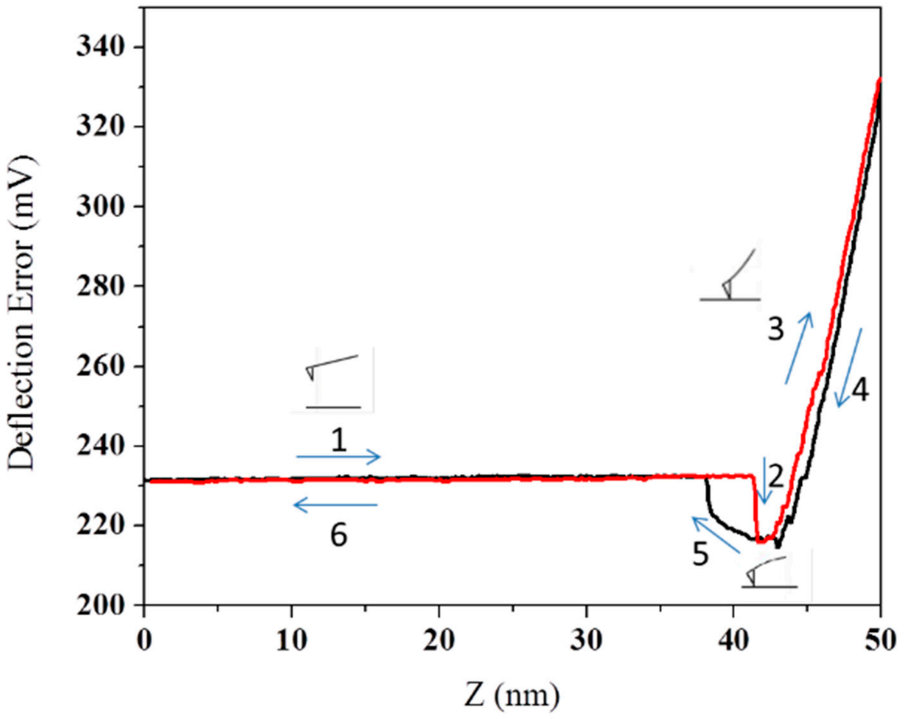

Figure S5. Deflection of the cantilever versus Z position obtained from the calibration process. It contains the approaching (numbers 1–3, red curve) and retracting positions during the test (numbers 4–6, black curve). Slope of 4 is used to determine deflection.

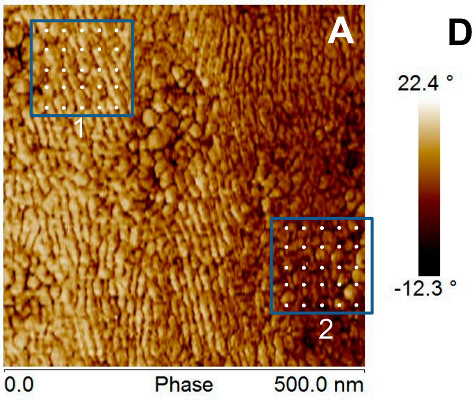

B

Sens. DeflSens	59.9 nm/V
Spring Constant	26.1 N/m
Tip Radius	2.00 nm
Tip Half Angle	18.0
Sample Poisson's Ratio	0.300

C

Indentation force	10 nN
Indentation velocity	3 um/s

D

1 Image File Name		Young's Modulus (MPa)	Reduced Modulus (MPa)	2 Image File Name		Young's Modulus (MPa)	Reduced Modulus (MPa)
silk_500000.005		1.34	1.47	silk_500025.005		3.19	3.51
silk_500001.005		1.2	1.32	silk_500026.005		3.23	3.55
silk_500002.005		1.08	1.19	silk_500027.005		3.36	3.69
silk_500003.005		1.09	1.19	silk_500028.005		2.52	2.77
silk_500004.005		1.12	1.23	silk_500029.005		2.57	2.82
silk_500005.005		2.04	2.25	silk_500030.005		2.42	2.66
silk_500006.005		2.16	2.38	silk_500031.005		2.94	3.23
silk_500007.005		2.17	2.38	silk_500032.005		3.47	3.81
silk_500008.005		2.33	2.56	silk_500033.005		3.4	3.73
silk_500009.005		1.96	2.15	silk_500034.005		3.49	3.84
silk_500010.005		1.93	2.12	silk_500035.005		3.3	3.63
silk_500011.005		2.24	2.46	silk_500036.005		3.8	4.17
silk_500012.005		2.06	2.27	silk_500037.005		3.84	4.22
silk_500013.005		2.01	2.21	silk_500038.005		3.52	3.87
silk_500014.005		2.16	2.37	silk_500039.005		1.37	1.5
silk_500015.005		2.71	2.98	silk_500040.005		1.47	1.62
silk_500016.005		1.95	2.14	silk_500041.005		3.32	3.64
silk_500017.005		1.89	2.08	silk_500042.005		3.64	3.99
silk_500018.005		2.13	2.34	silk_500043.005		3.57	3.92
silk_500019.005		1.94	2.13	silk_500044.005		2.99	3.28
silk_500020.005		1.91	2.1	silk_500045.005		1.64	1.81
silk_500021.005		2.41	2.65	silk_500046.005		3.46	3.8
silk_500022.005		2.03	2.23	silk_500047.005		3.68	4.04
silk_500023.005		1.8	1.98	silk_500048.005		3.73	4.1
silk_500024.005		2.12	2.33	silk_500049.005		2.36	2.6
avg.		1.91	2.10	avg.		3.05	3.35
stdev.		0.43	0.47	stdev.		0.72	0.79
max.		2.71	2.98	max.		3.84	4.22
min.		1.08	1.19	min.		1.37	1.50

Figure S6. Settings and full results of AFM quantitative nano-mechanical mapping of two regions along a single *Arachnocampa tasmaniensis* glowworm capture thread. Two 5 x 5 grids were mapped along known regions of a phase image (A). Cantilever parameters (B), indentation settings (C), and raw data from indentation tests (D) are given.

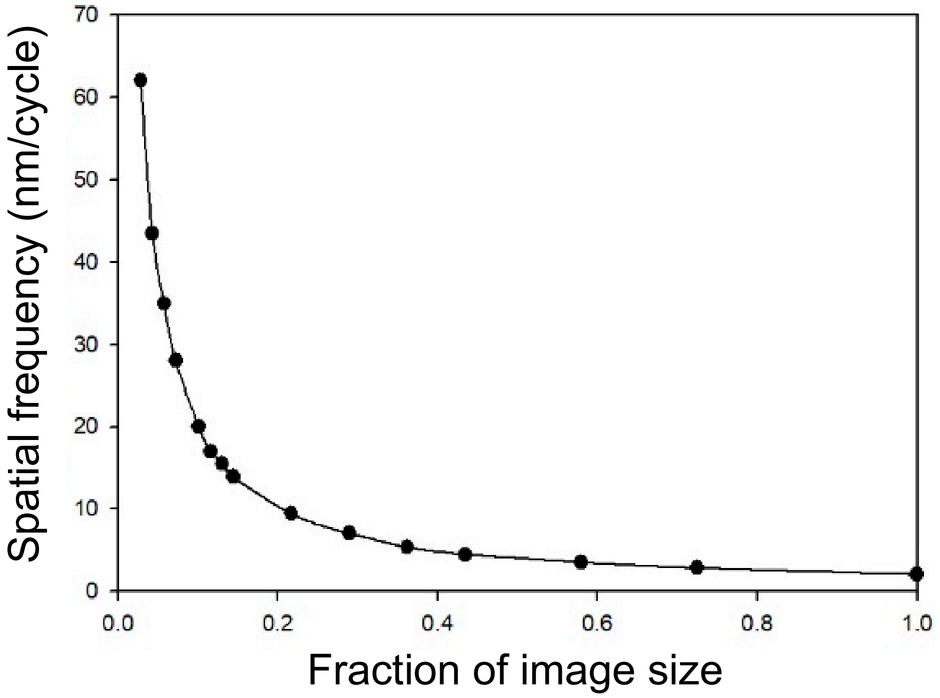

Figure S7. Calibration curve from the linear scale of FFT images to the spatial period/cycle (sp) of the features in the original phase images. Zero (infinite sp) corresponds to the centre of the FFT image.

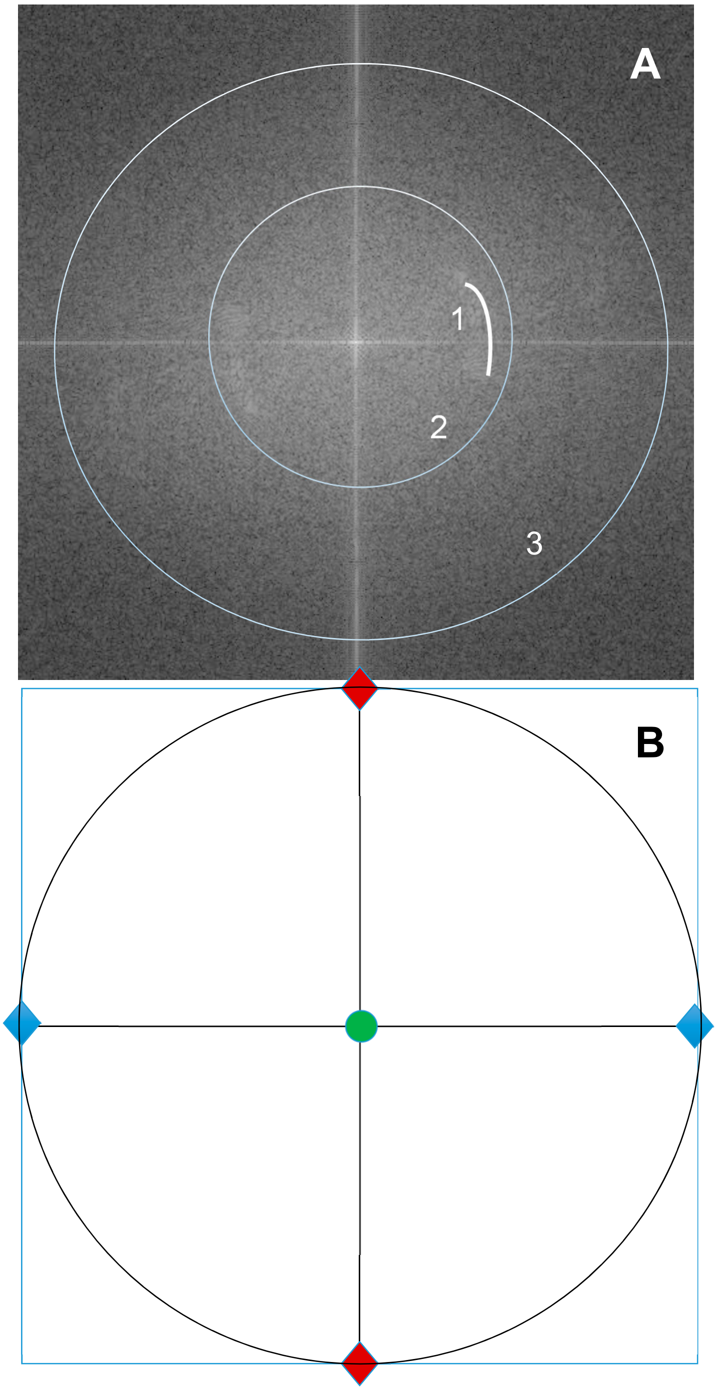

Figure S8. Qualitative characterization of the fast Fourier transform (FFT) of the of phase images *Arachnocampa tasmaniensis* silk threads. From FFT images (A), three primary features were identified: arcs (1), a near dense cloud (2), and far diffuse cloud (3). The right arc (1, one of the symmetric pair) has a spatial period range of 10.6 – 12.9 nm and angle range of 54°: (35.84° – 341.85°). Note the left arc has angle range of 52° (from 164.95° to 216.75°). The arcs indicate a stronger presence of the specified range of spatial periods compared to all others in the original image. These are preferentially oriented from -36 to +18 relative to the vertical axis of the original image. The boundary of near dense cloud (2) marks a spatial period of 12 nm. The boundary of far diffuse cloud (3) marks a spatial period of 5 nm. This indicates the original image contains all spatial periods larger than this value at all angular orientations. The diagram (B) represents 0.912 of the full FFT image scale with the spatial period of circular boundary ~4.7 nm. Green circles represent infinite spatial period with a 0 spatial frequency. Blue diamonds represent features on the horizontal axis that are associated with vertically oriented features in the original phase image. Red diamonds represent features appearing on the vertical axis that are associated with horizontally oriented spatial features in the original phase image.
