# Supplementary material for: Nanoscale Material Heterogeneity of Glowworm Capture Threads Revealed by AFM
Source: Molecules. 2021 Jun 8;26(12):3500. doi: 10.3390/molecules26123500 (PMC8226719; doi:10.3390/molecules26123500)
Supplement: Supplementary file 1 [file molecules-26-03500-s001.zip › Glowworm ESM Figures S9-S106.pdf]

Figure S9 – Individual 1, Stretched, Fiber 1, Image 1, 2017/12/18

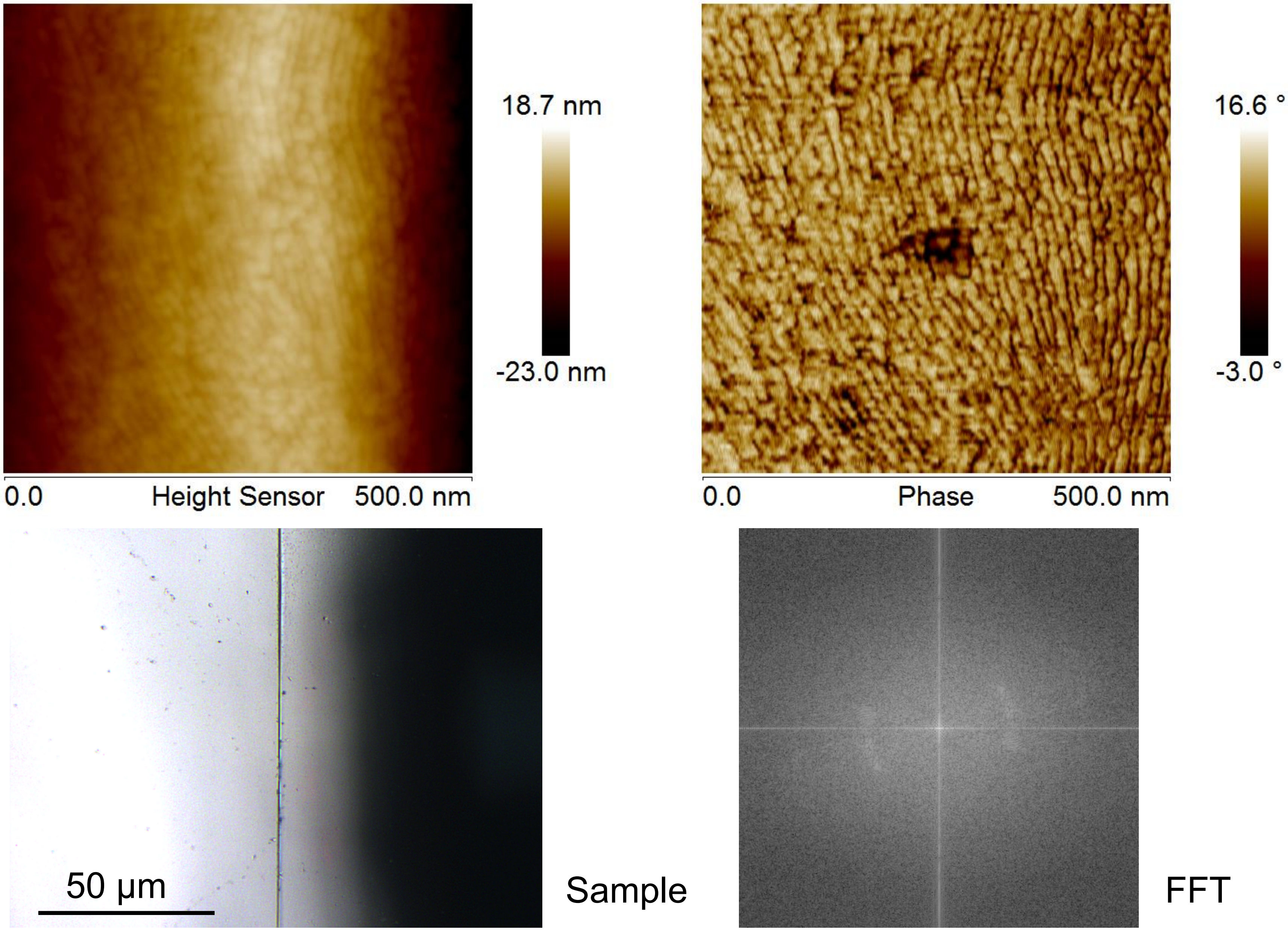

Figure S10 – Individual 1, Stretched, Fiber 1, Image 2, 2017/12/18

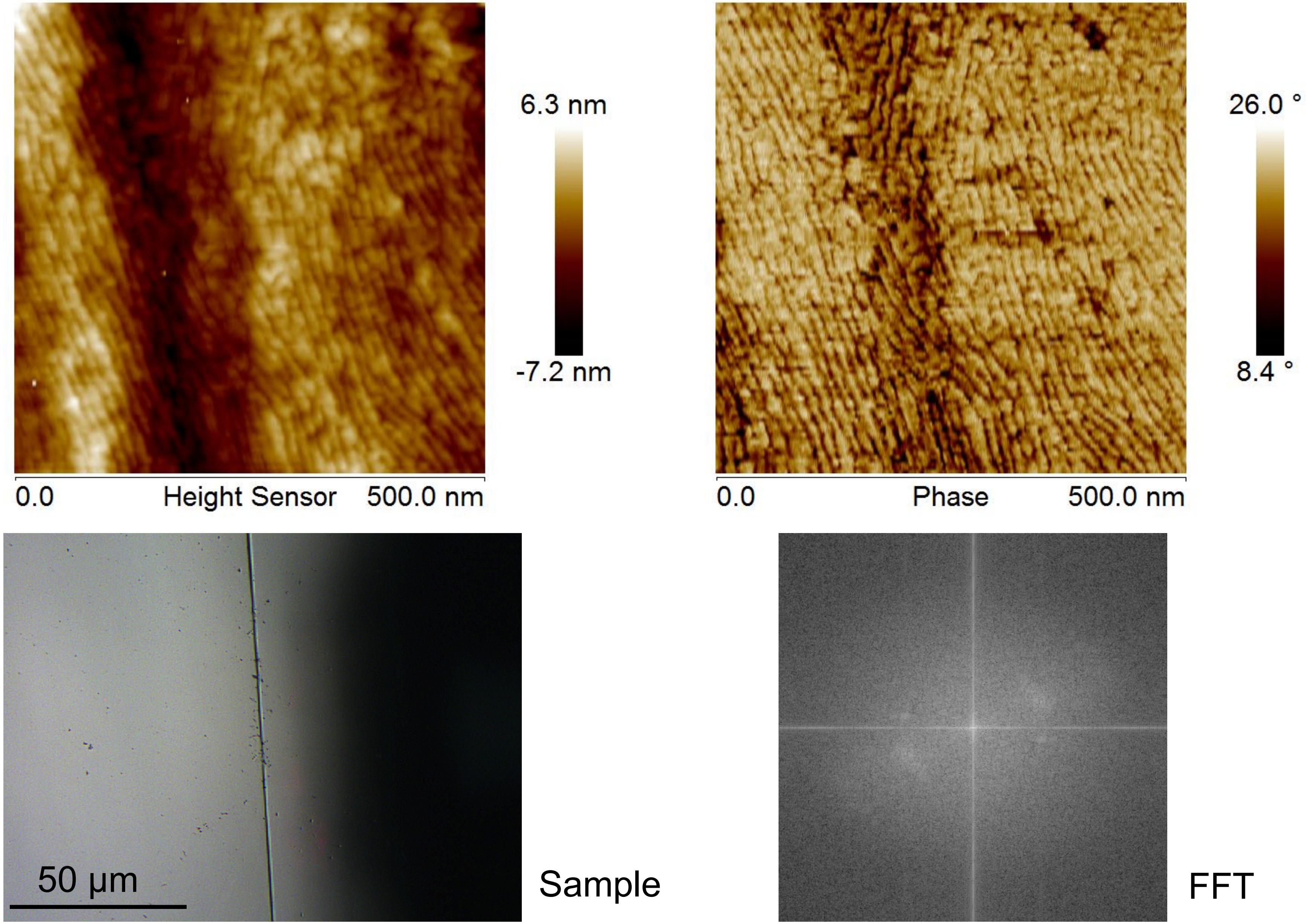

Figure S11 – Individual 1, Stretched, Fiber 1, Image 3, 2017/12/18

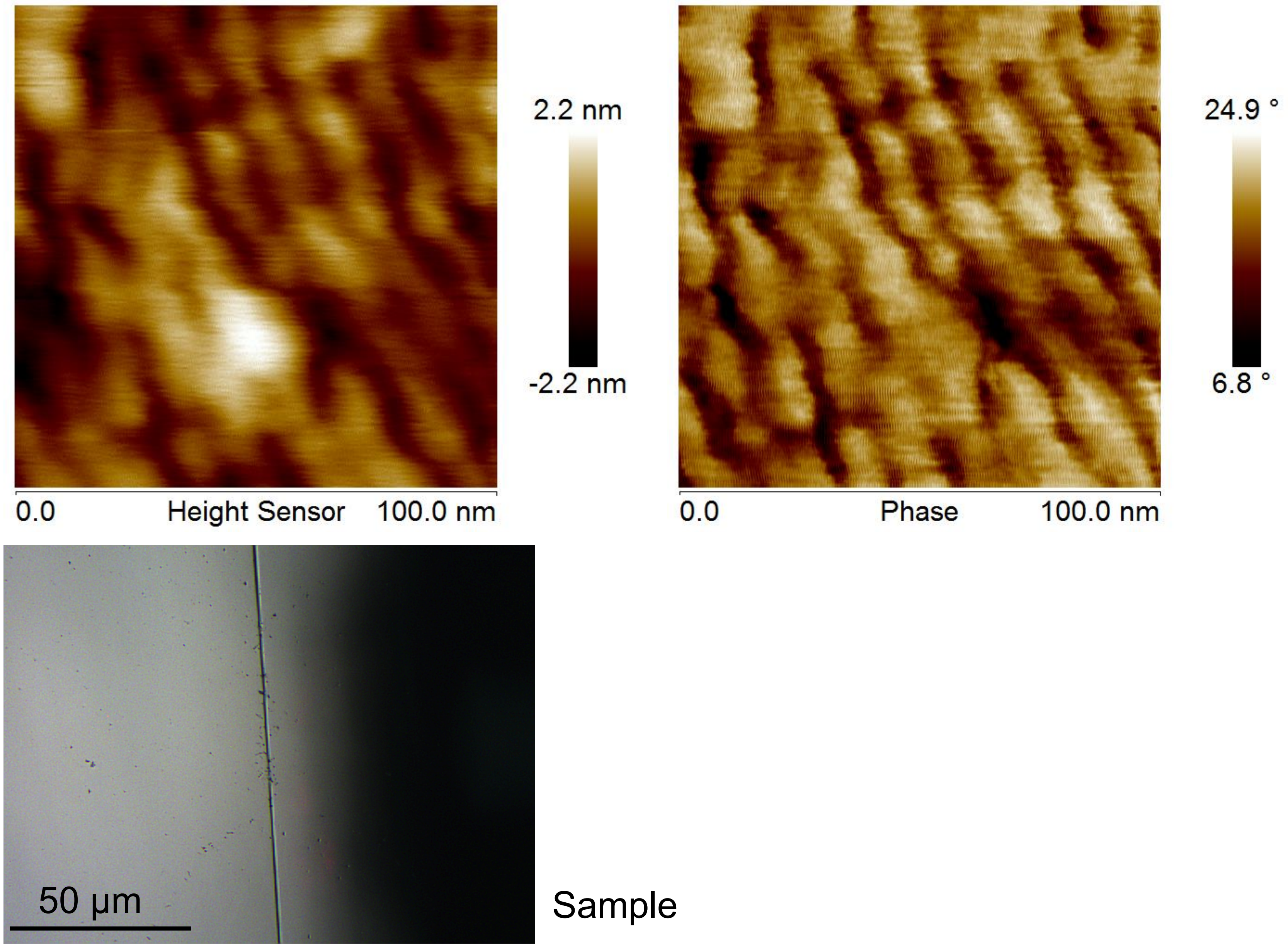

Figure S12 – Individual 1, Unstretched, Fiber 2, Image 4, 2017/12/18

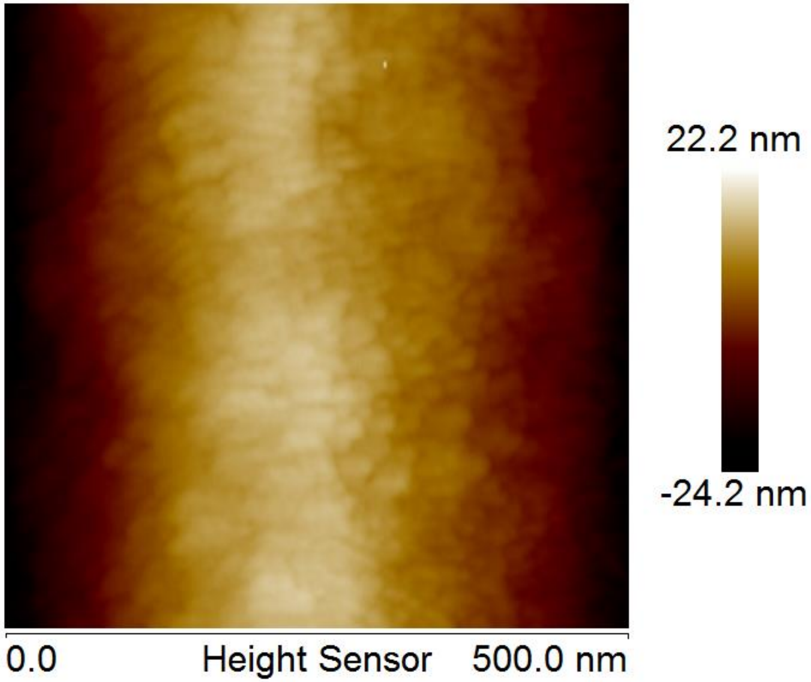

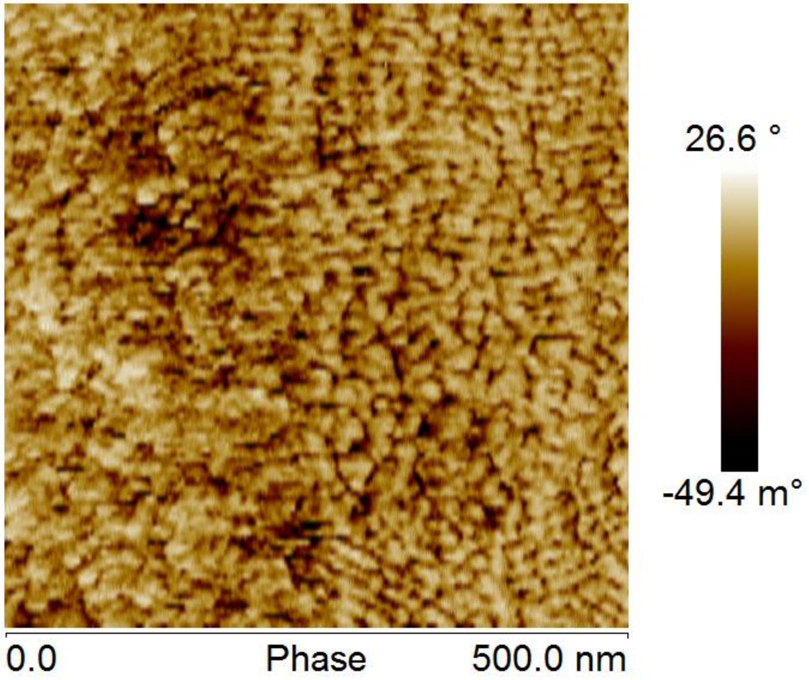

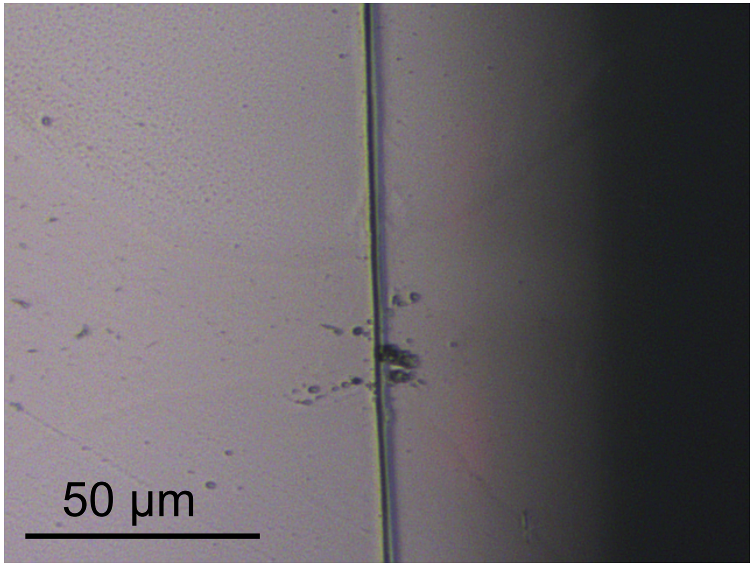

Sample

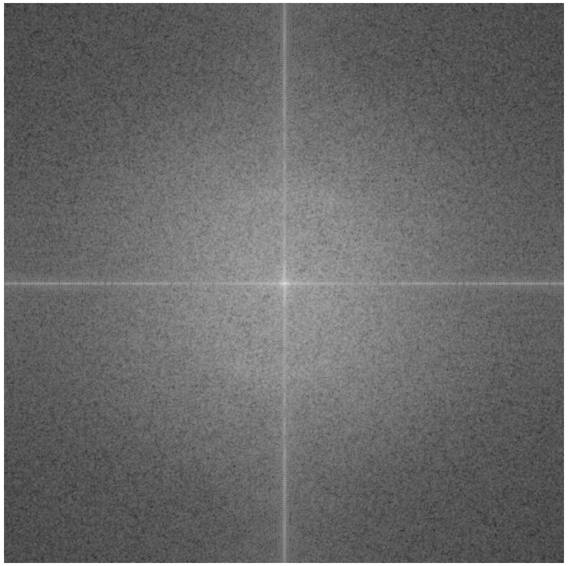

FFT

Figure S13 – Individual 1, Unstretched, Fiber 2, Image 5, 2017/12/18

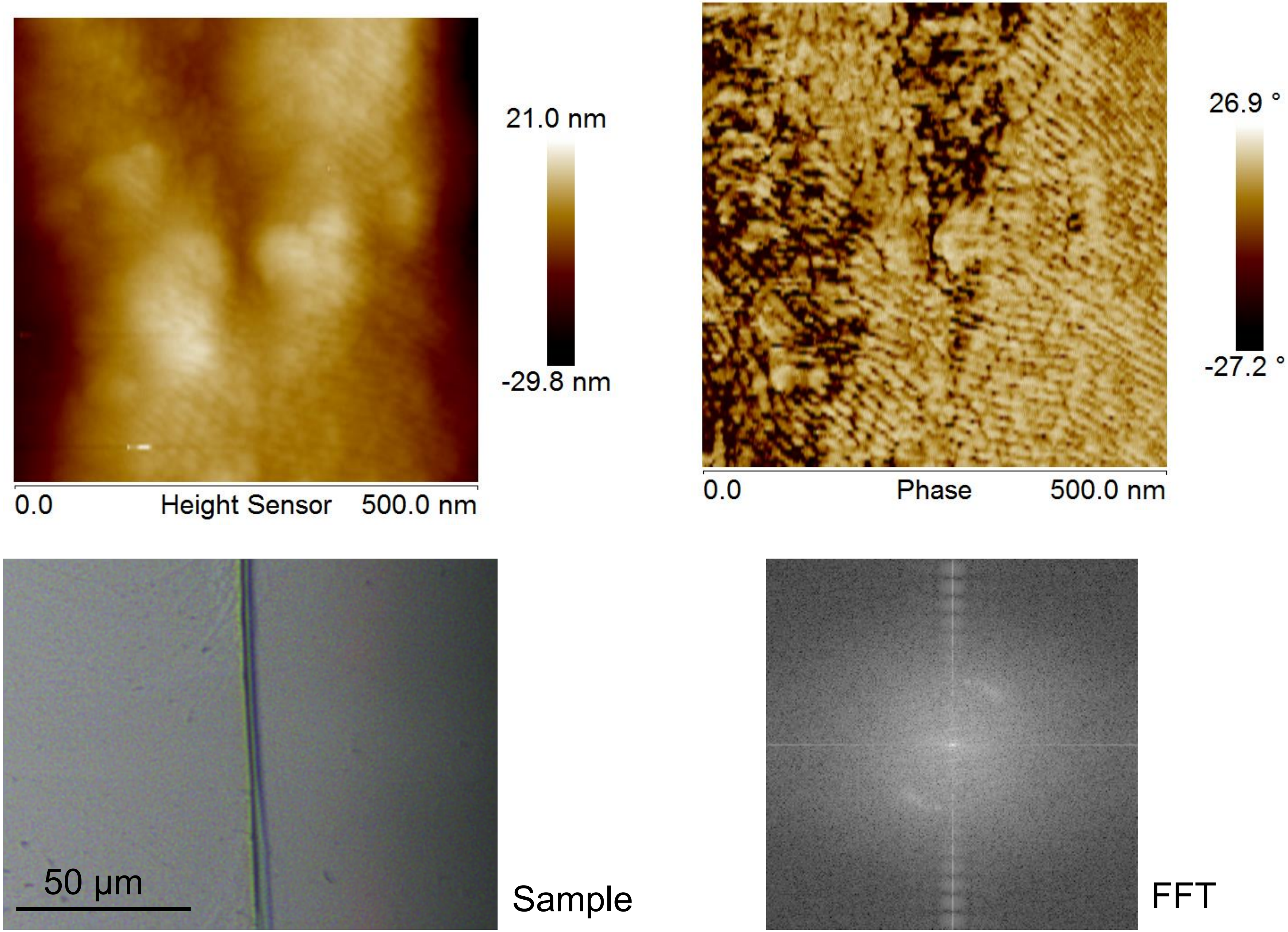

Figure S14 – Individual 1, Unstretched, Fiber 2, Image 6, 2017/12/18

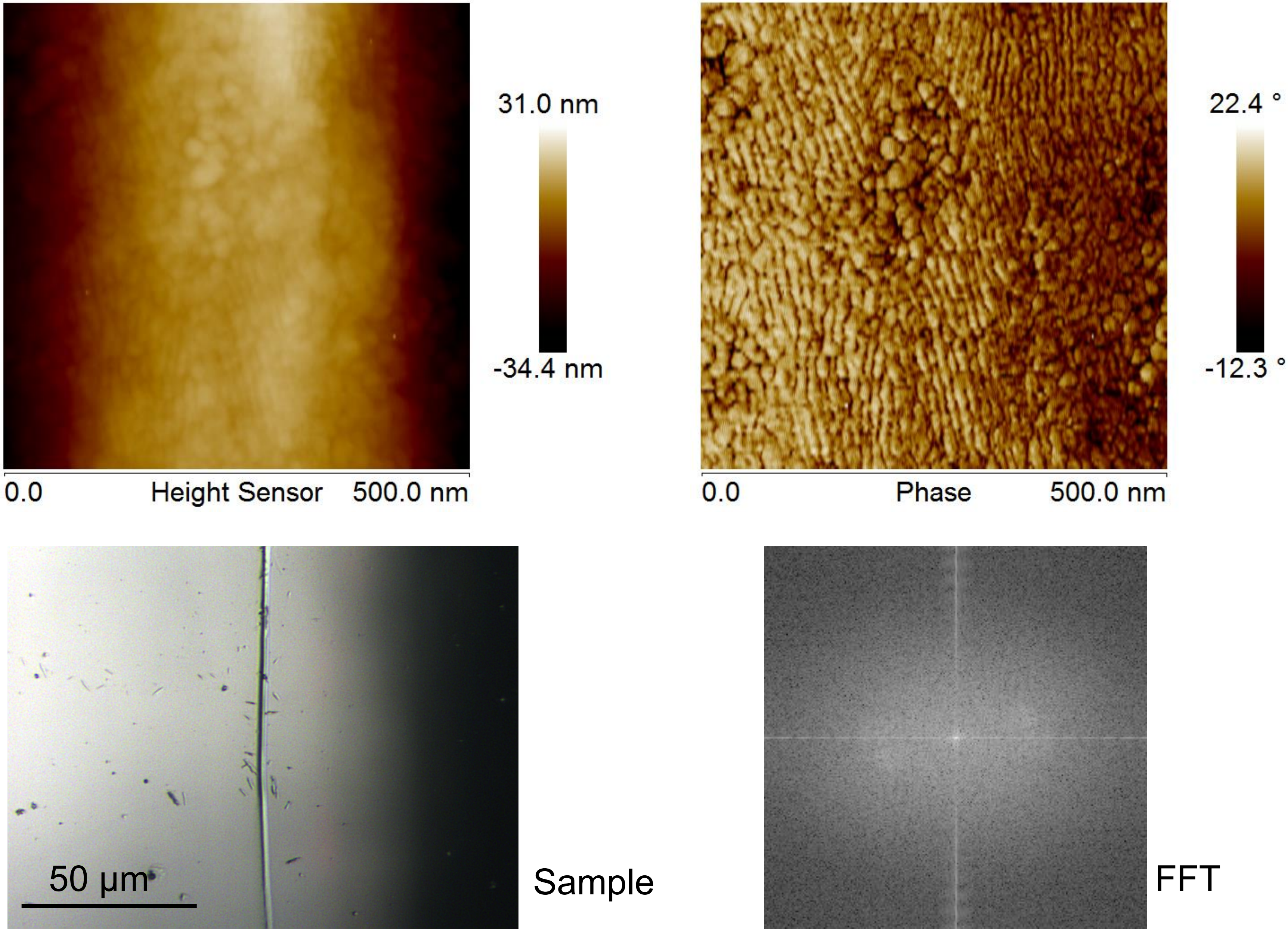

Figure S15 – Individual 1, Unstretched, Fiber 2, Image 7, 2017/12/18

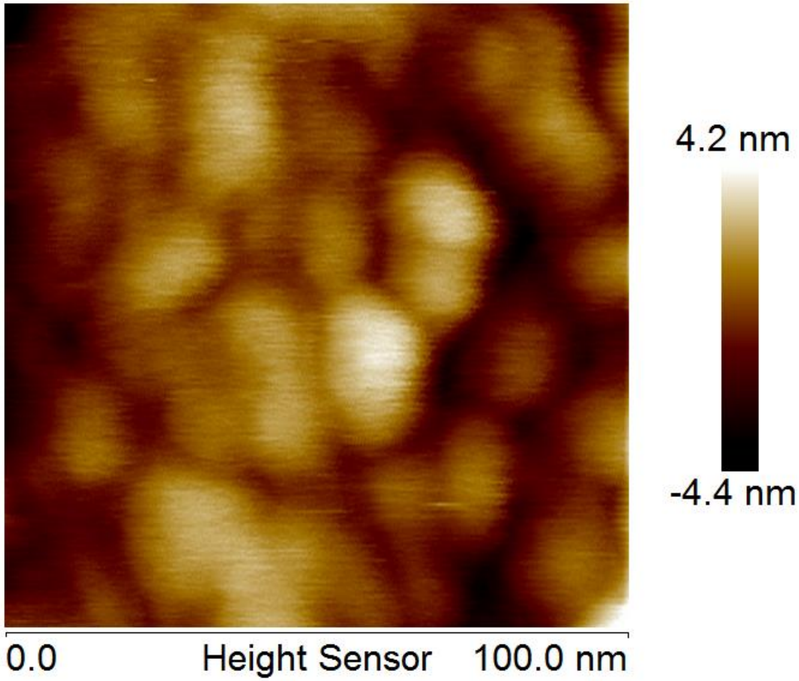

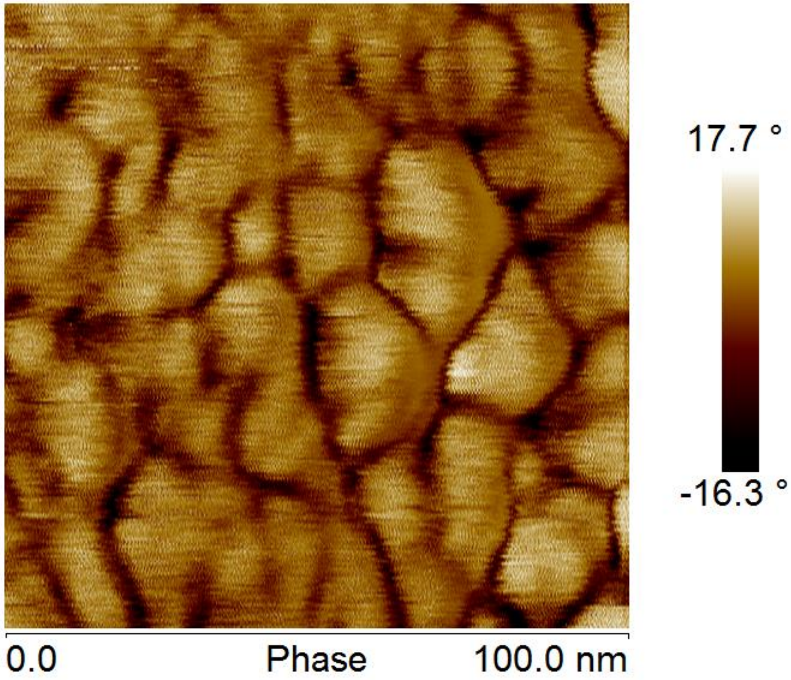

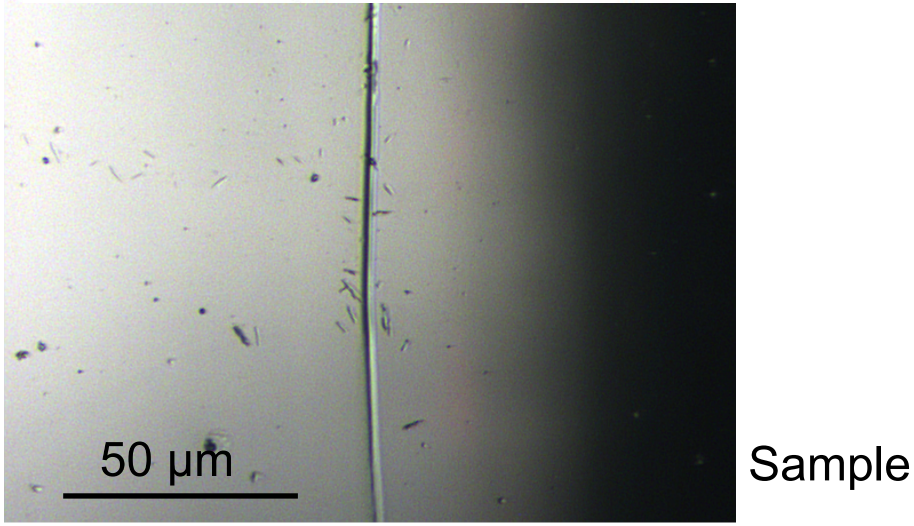

Sample

Figure S16 – Individual 1, Unstretched, Fiber 2, Image 8, 2017/12/18

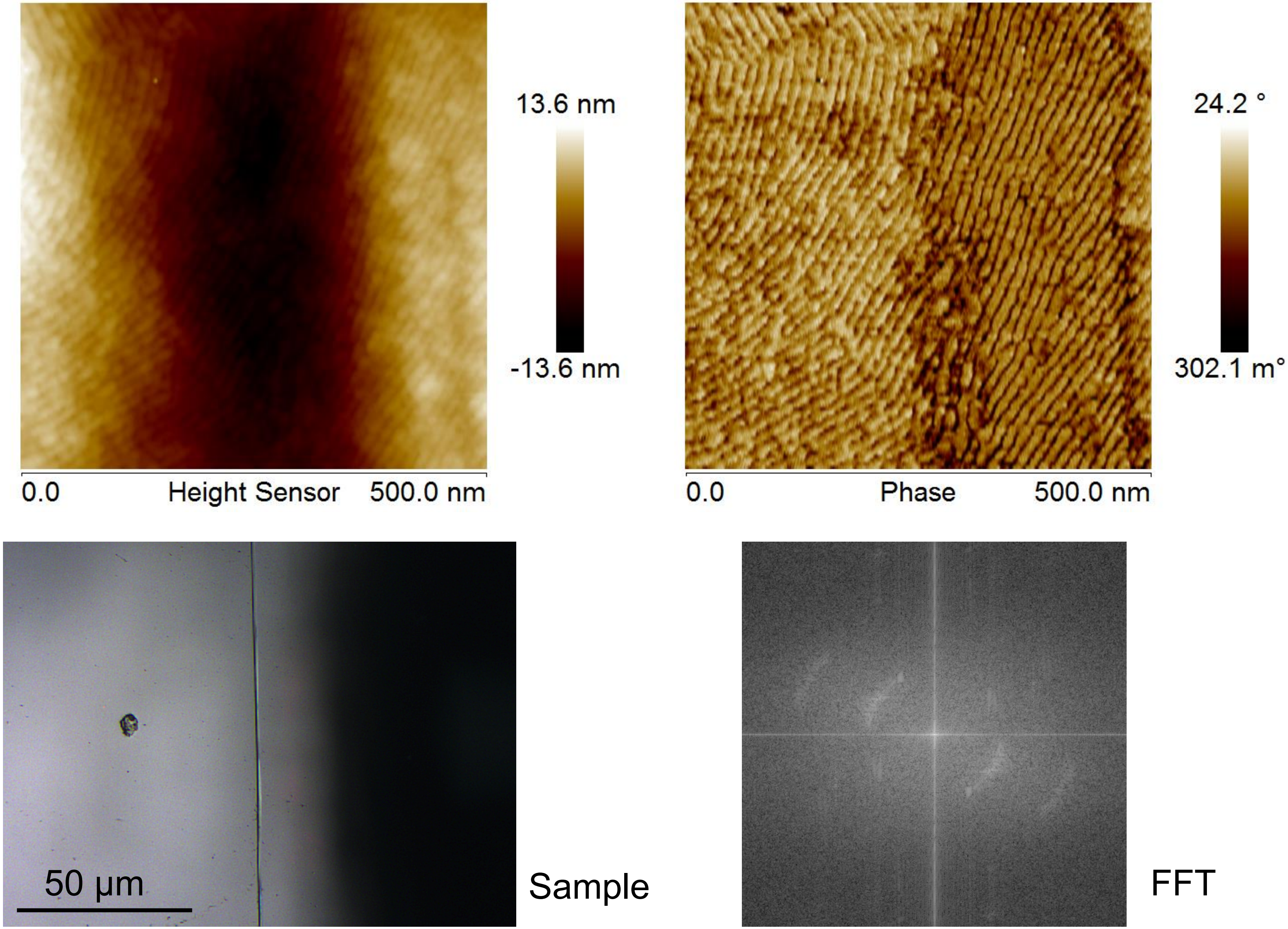

Figure S17 – Individual 1, Unstretched, Fiber 2, Image 9, 2017/12/18

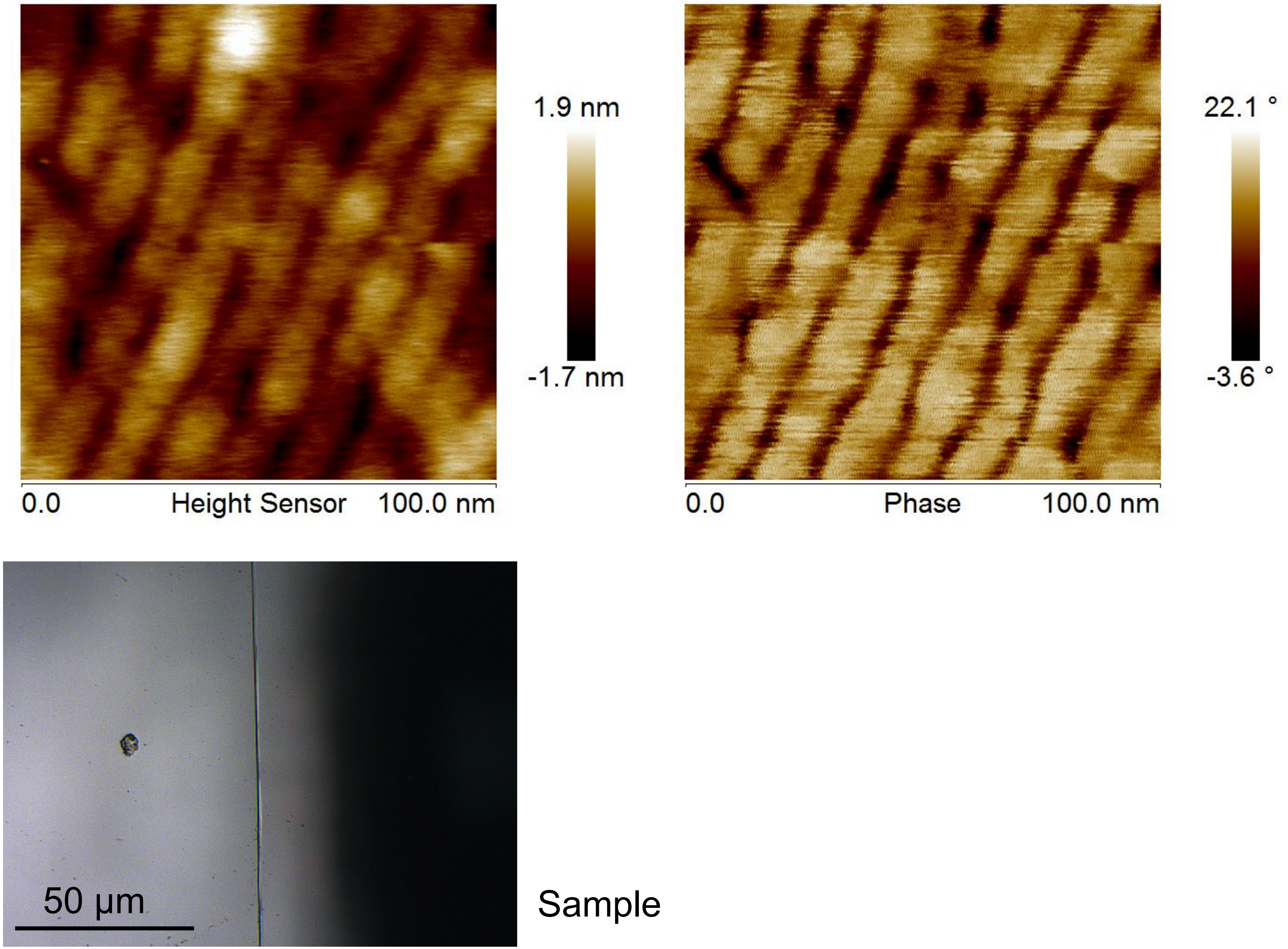

Figure S18 – Individual 1, Unstretched, Fiber 2, Image 10, 2017/12/18

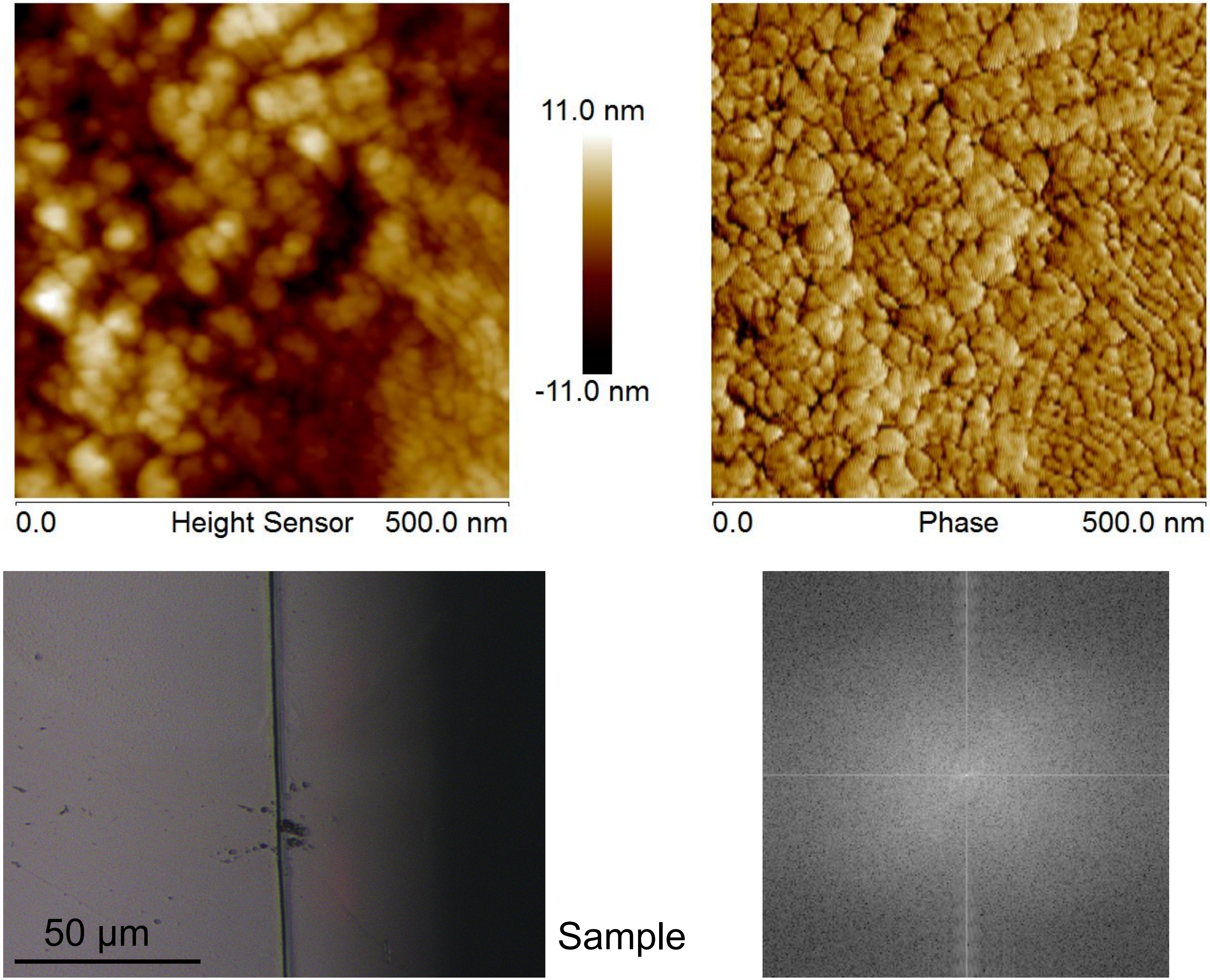

Figure S19 – Individual 1, Unstretched, Fiber 2, Image 11, 2017/12/18

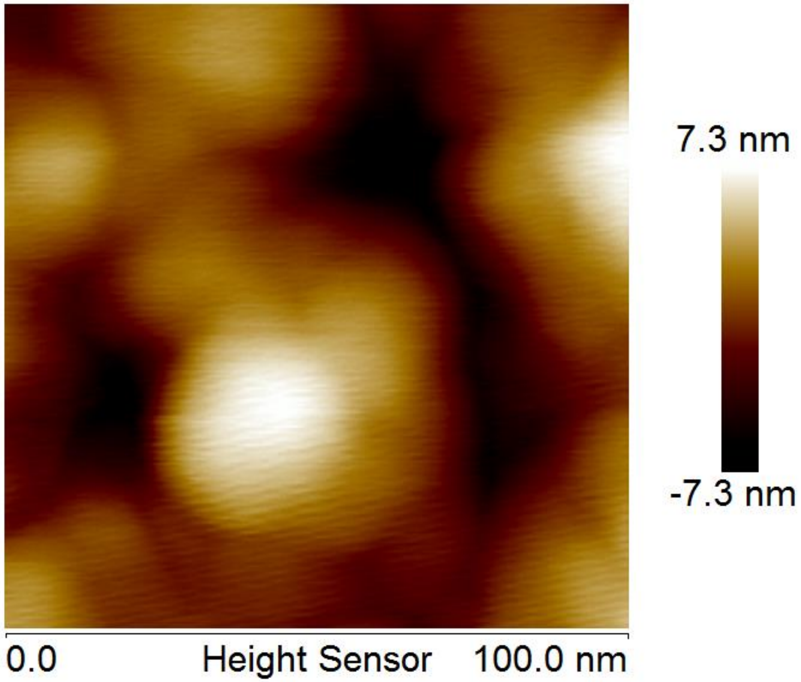

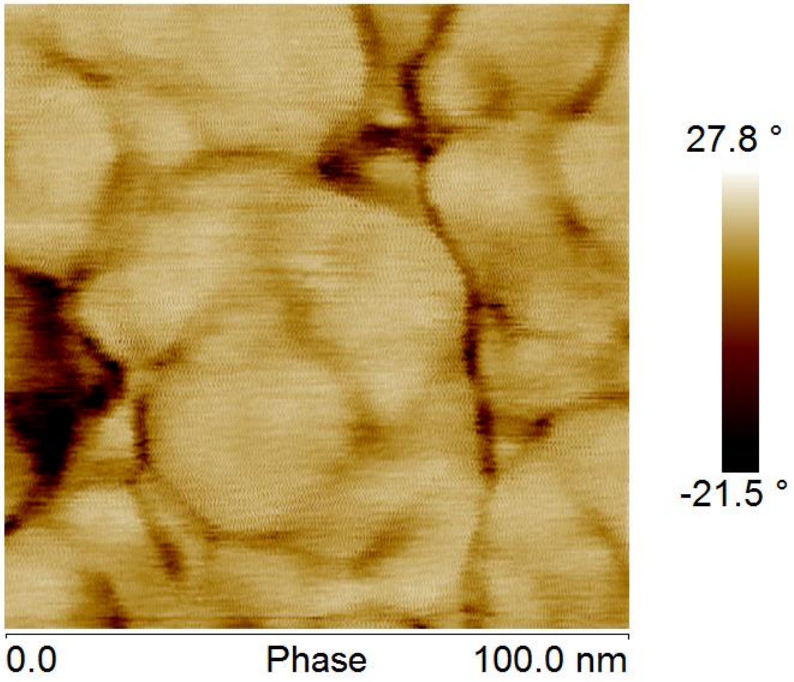

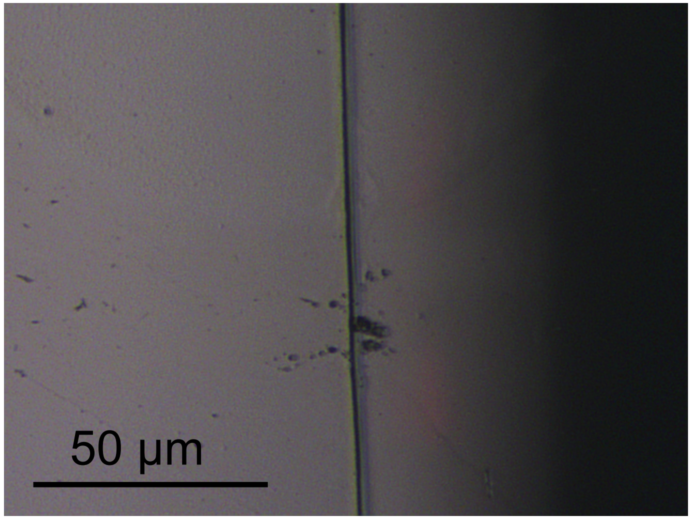

Sample

Figure S20 – Individual 2, Stretched, Fiber 3, Image 12

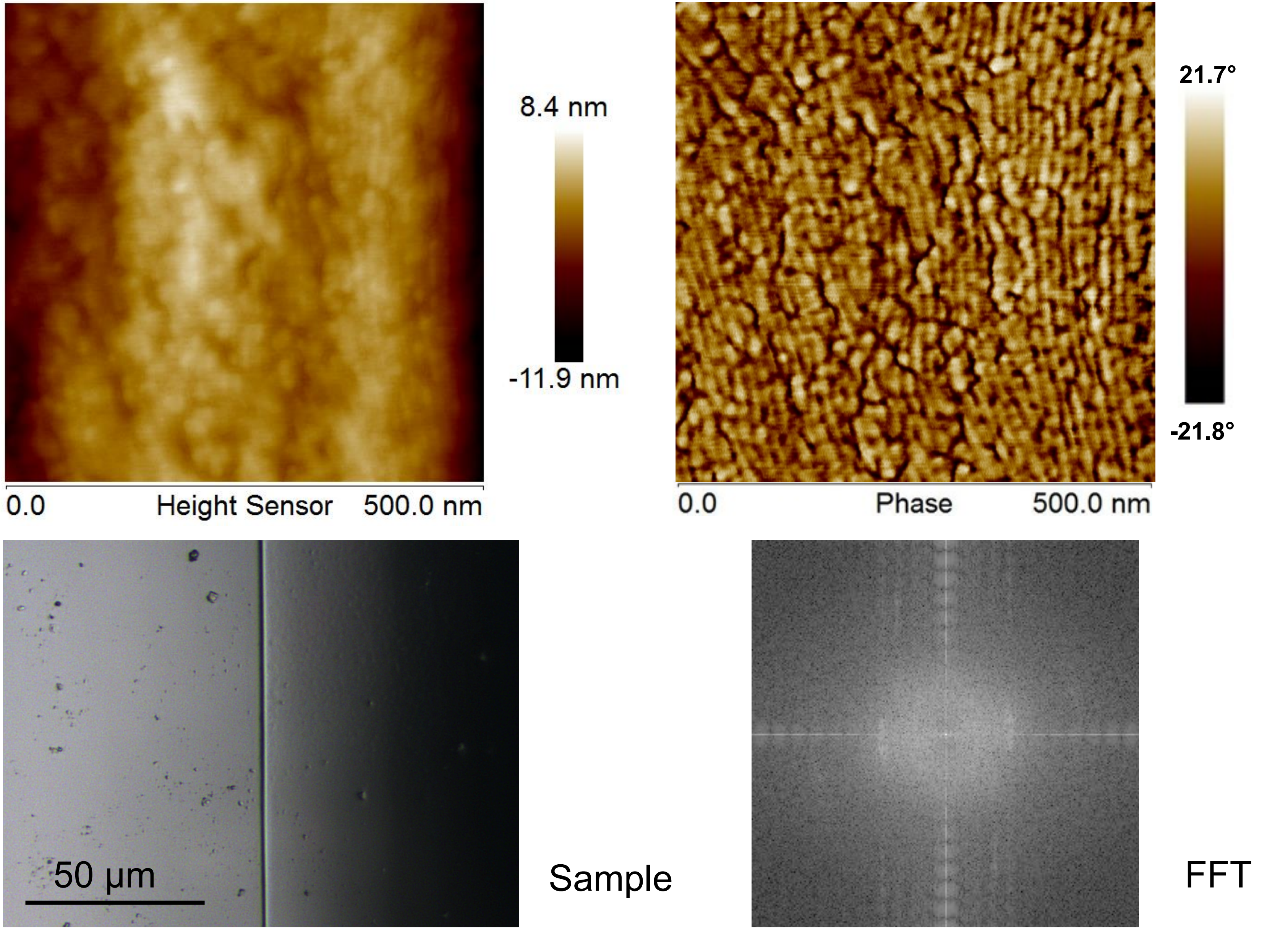

Figure S21 – Individual 2, Stretched, Fiber 3, Image 13, 2018/01/26

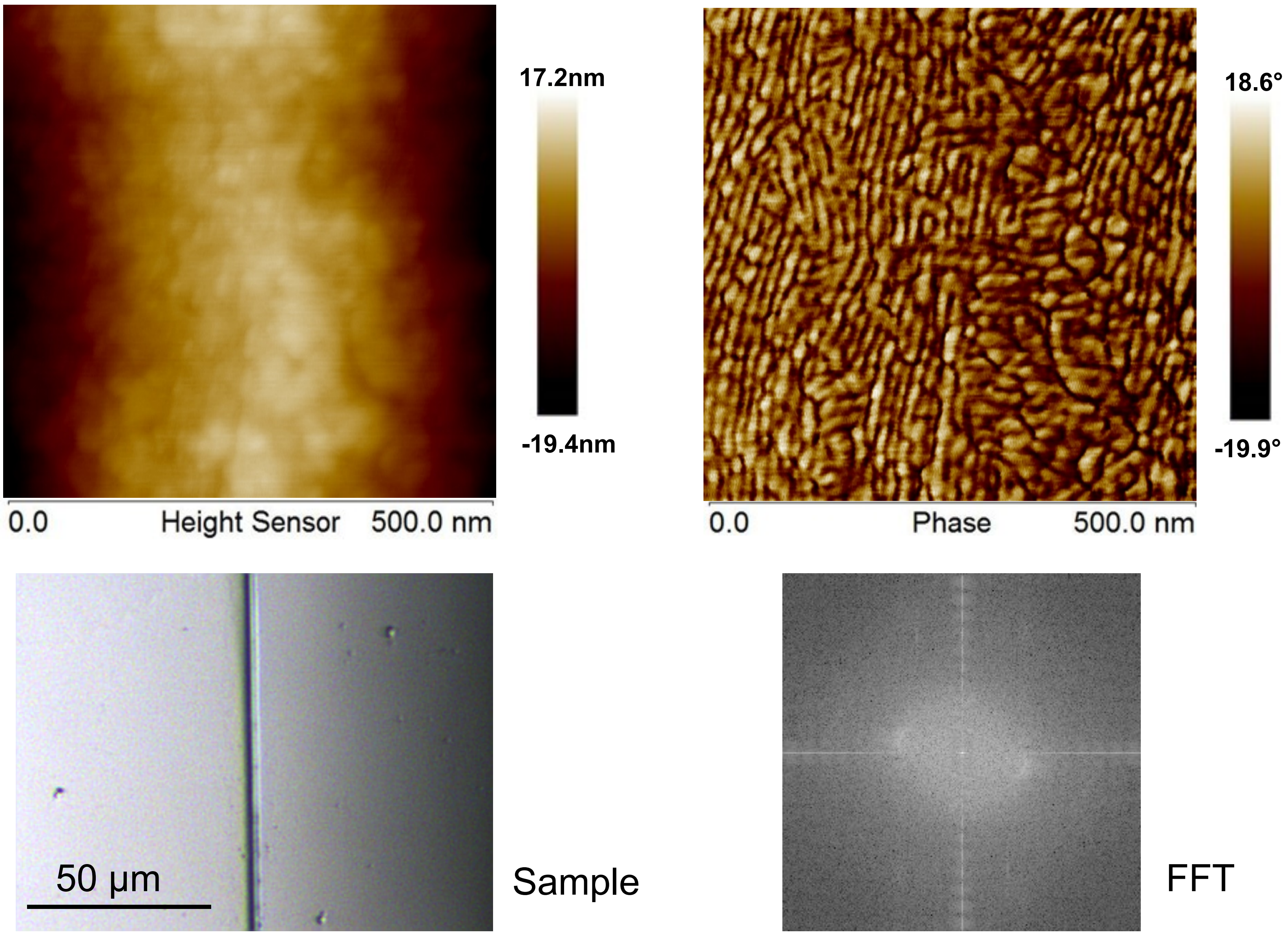

Figure S22 – Individual 2, Stretched, Fiber 3, Image 14, 2018/01/26

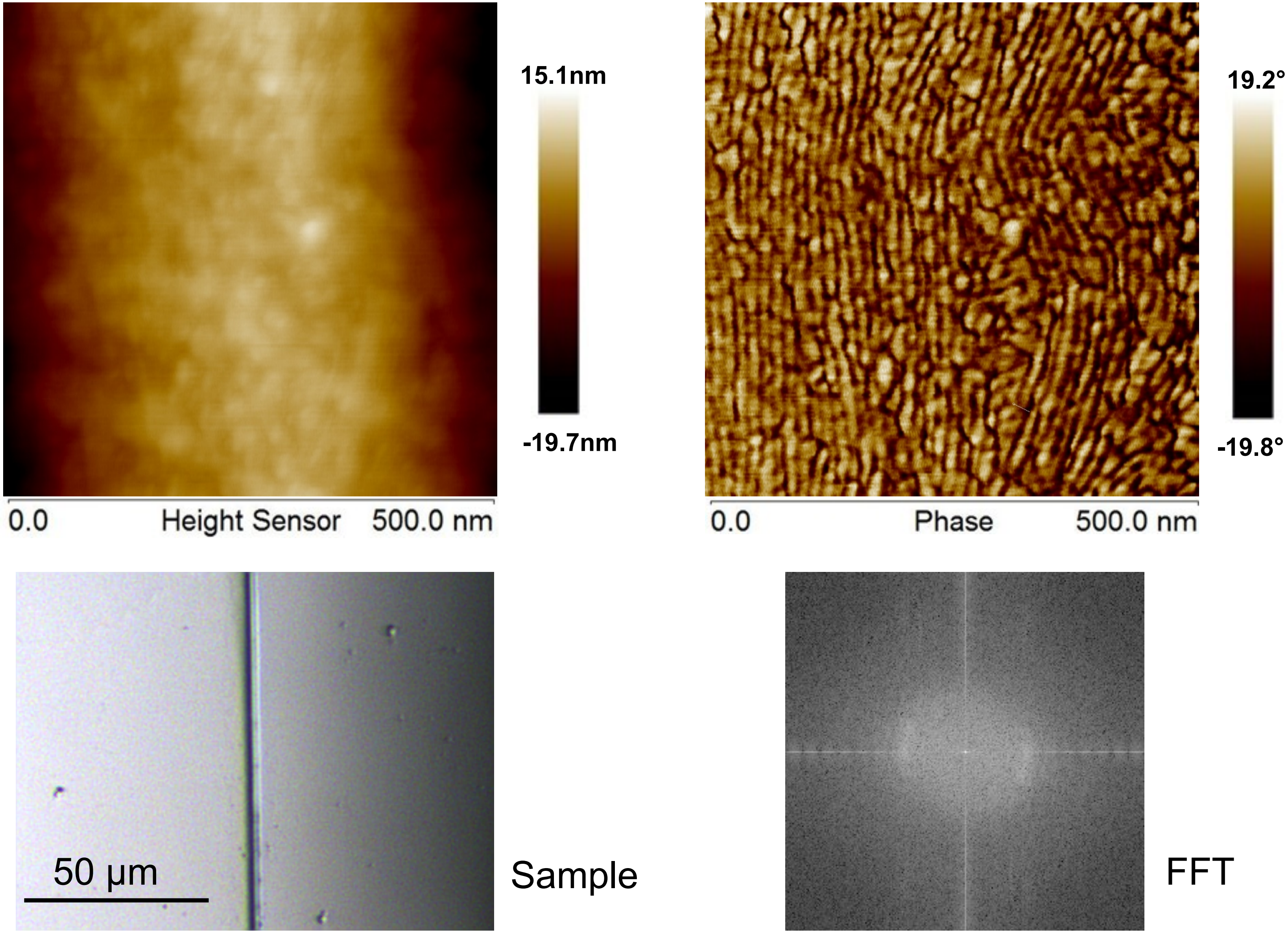

Figure S23 – Individual 3, Stretched, Fiber 4, Image 15, 2018/01/26

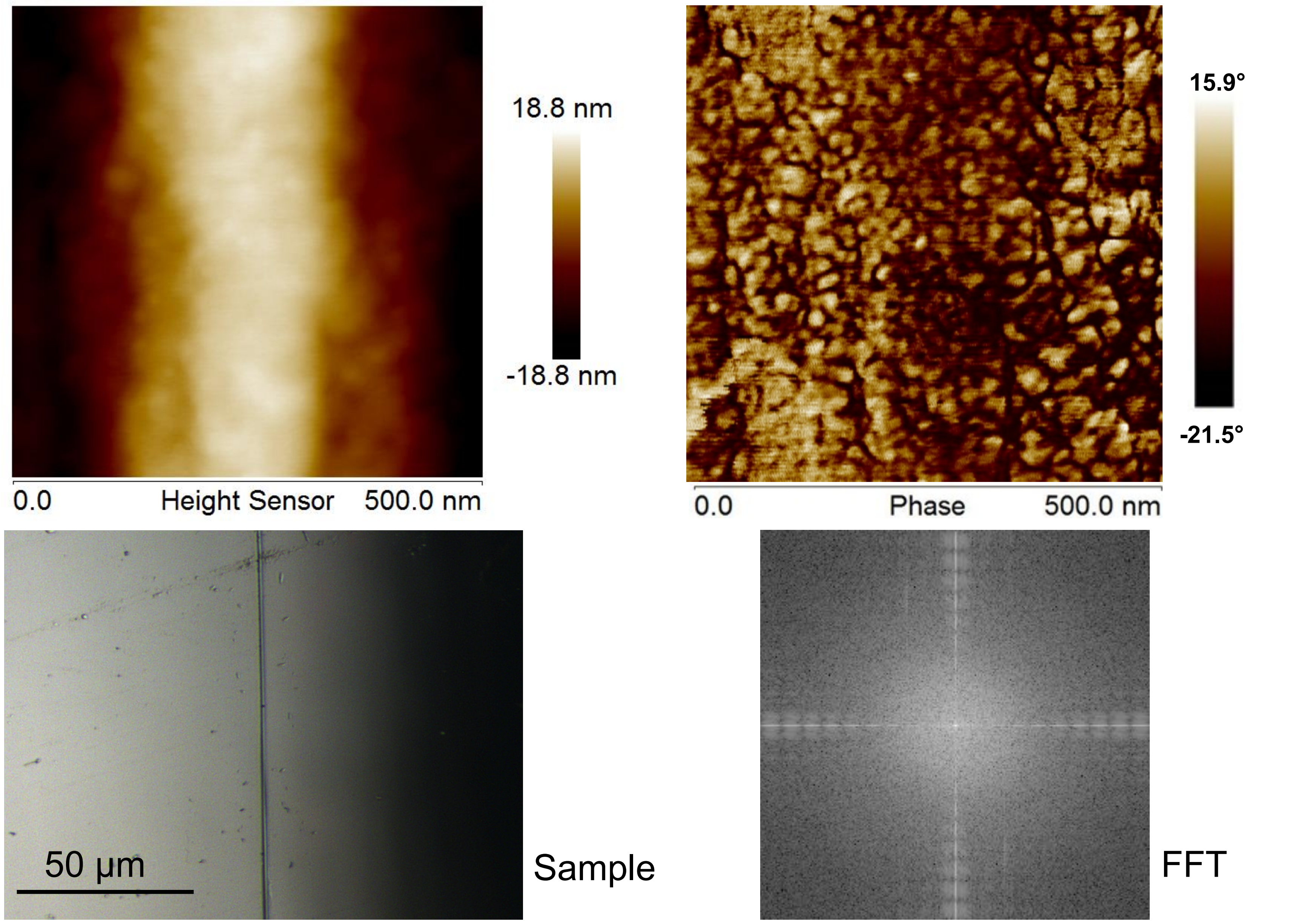

Figure S24 – Individual 3, Stretched, Fiber 4, Image 16, 2018/01/26

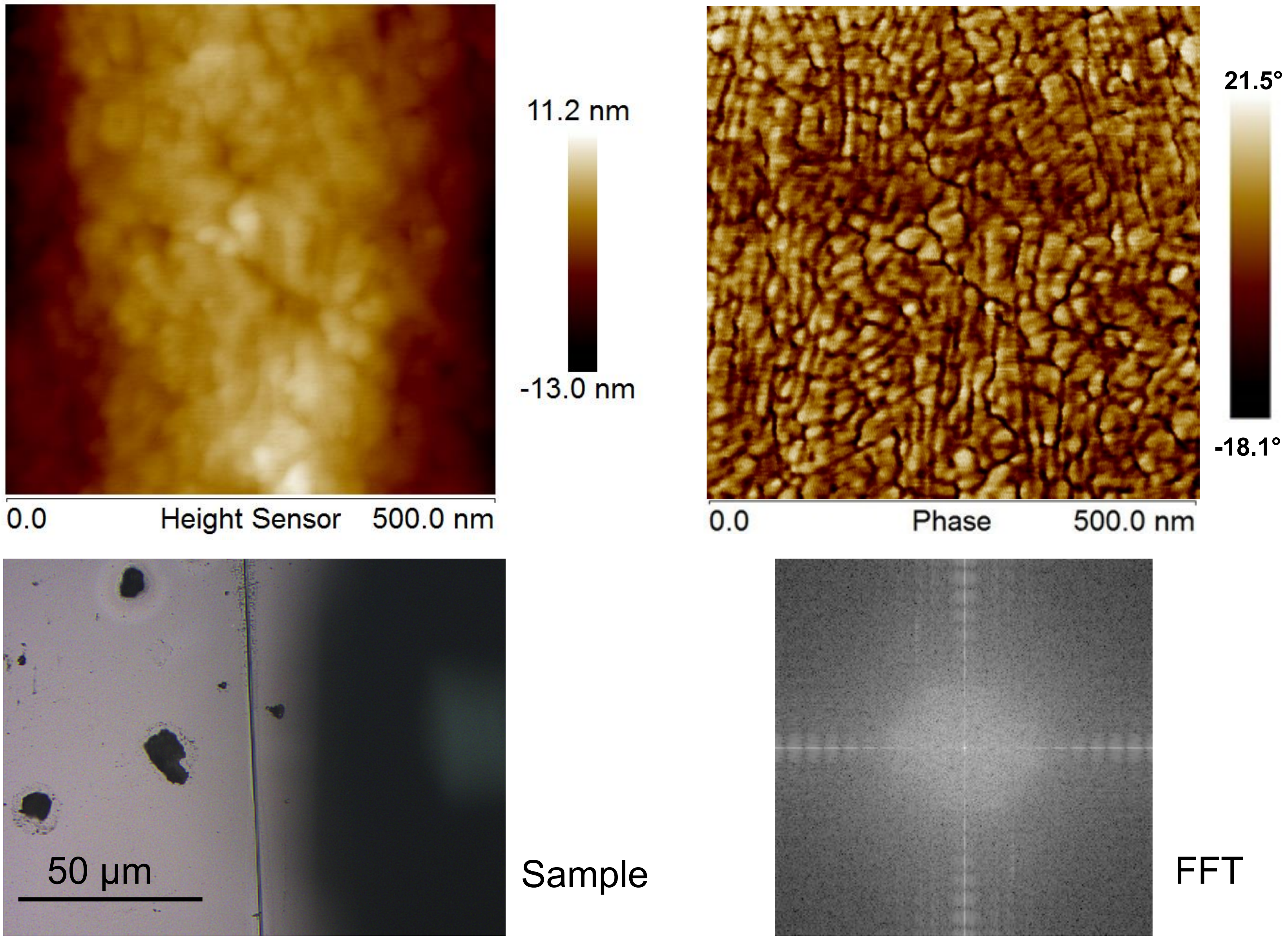

Figure S25 – Individual 3, Stretched, Fiber 4, Image 17, 2018/01/26

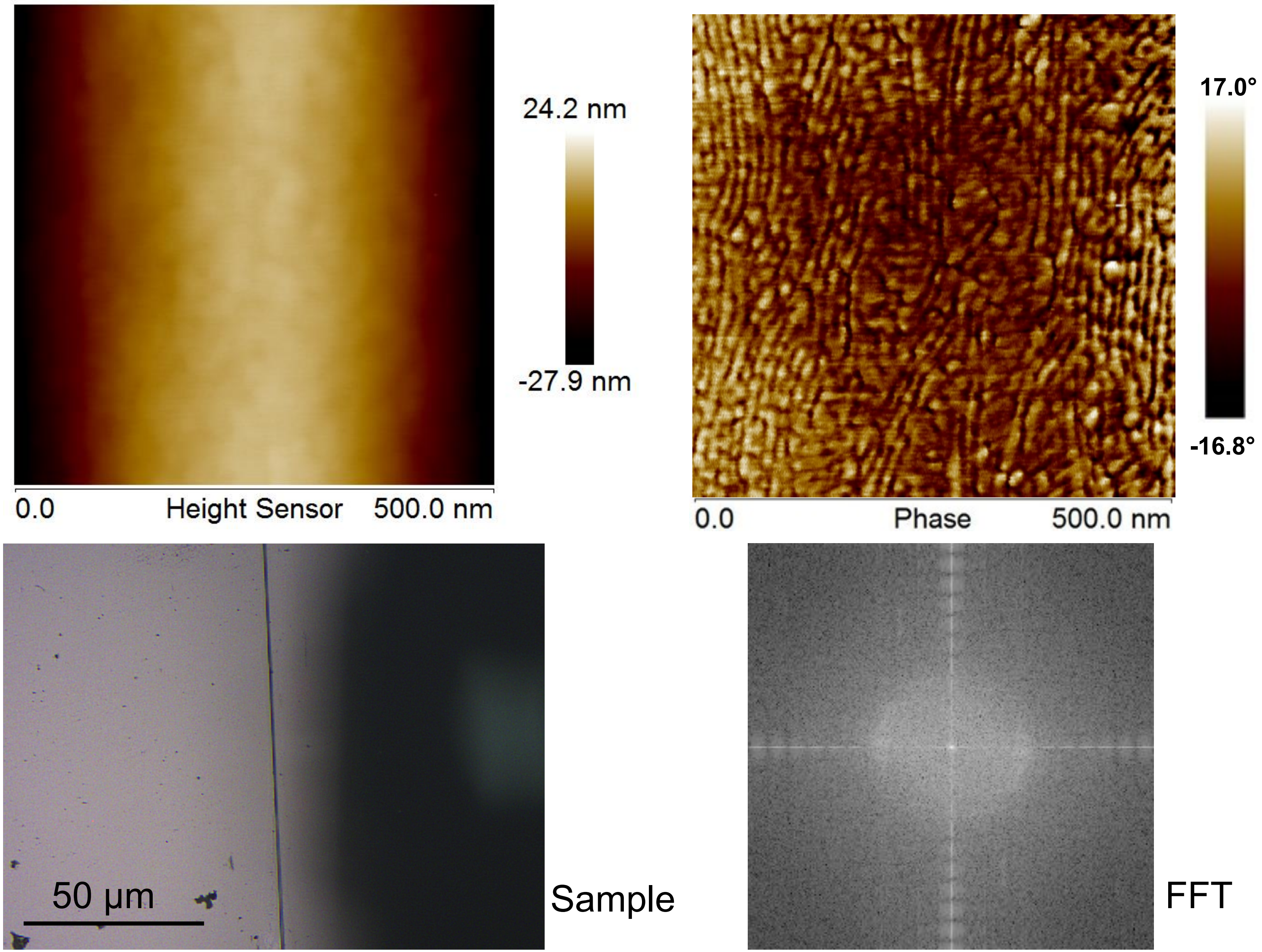

Figure S26 – Individual 3, Stretched, Fiber 4, Image 18, 2018/01/26

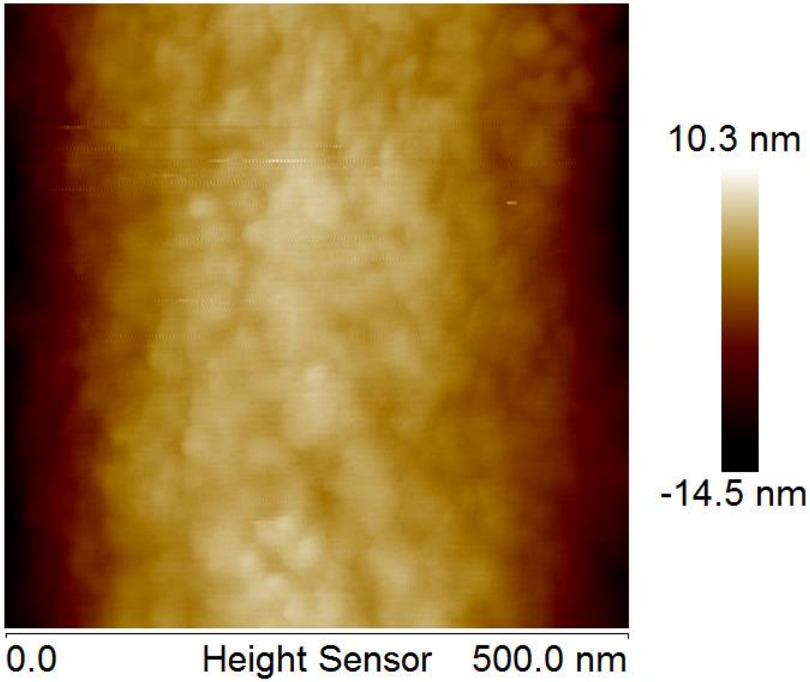

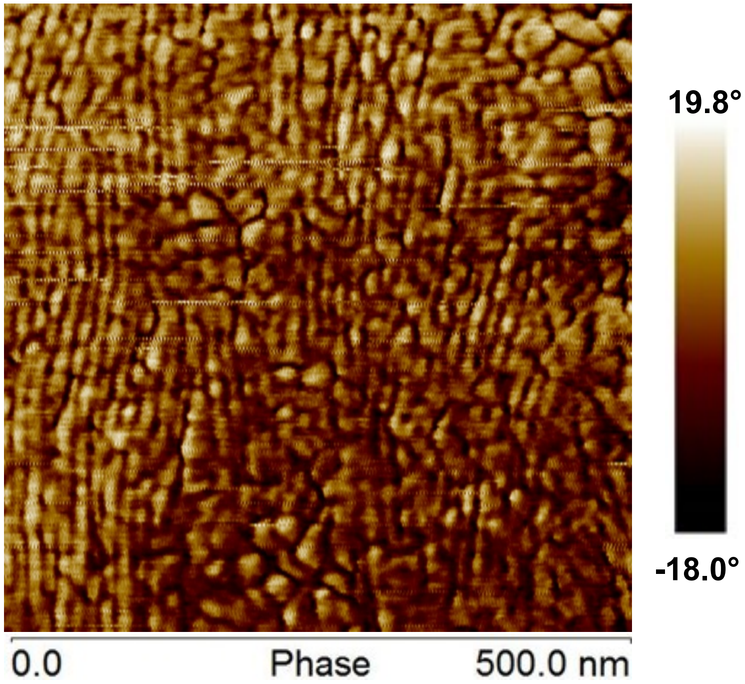

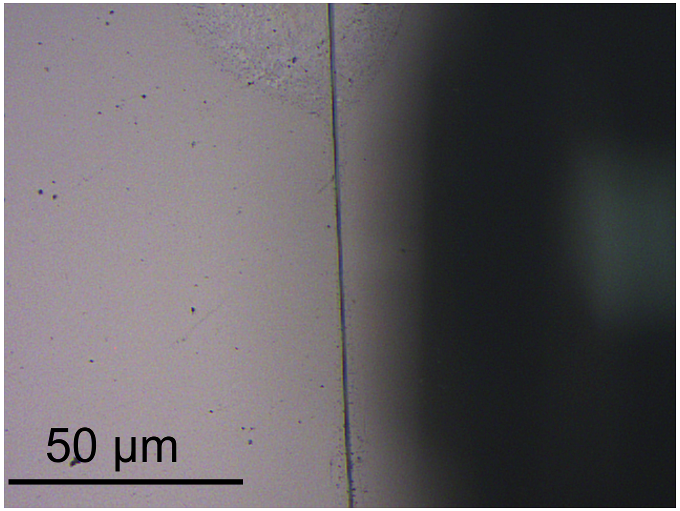

Sample

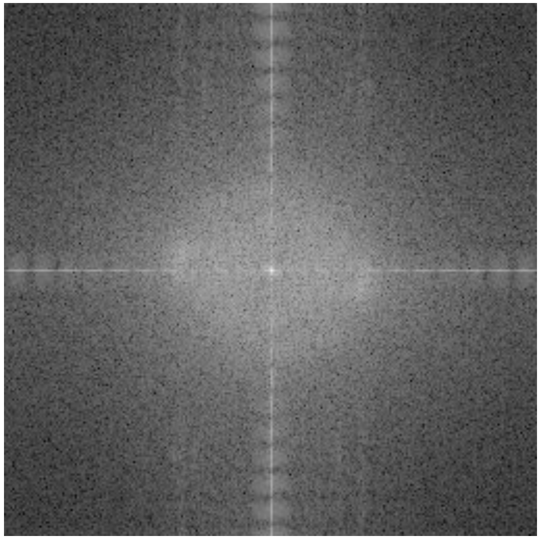

FFT

Figure S27 – Individual 2, Unstretched, Fiber 5, Image 19, 2018/01/26

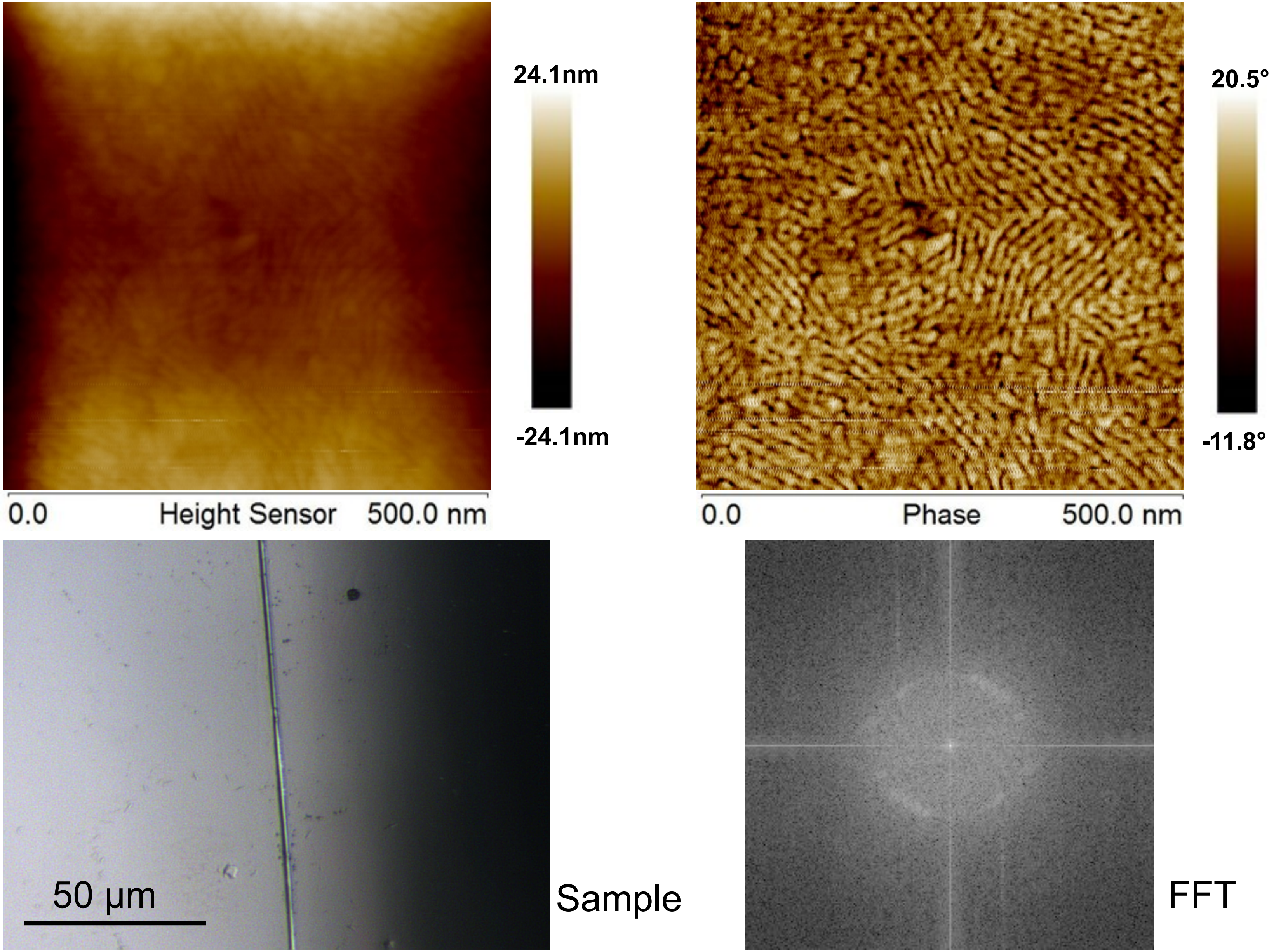

Figure S28 – Individual 2, Unstretched, Fiber 5, Image 20, 2018/01/26

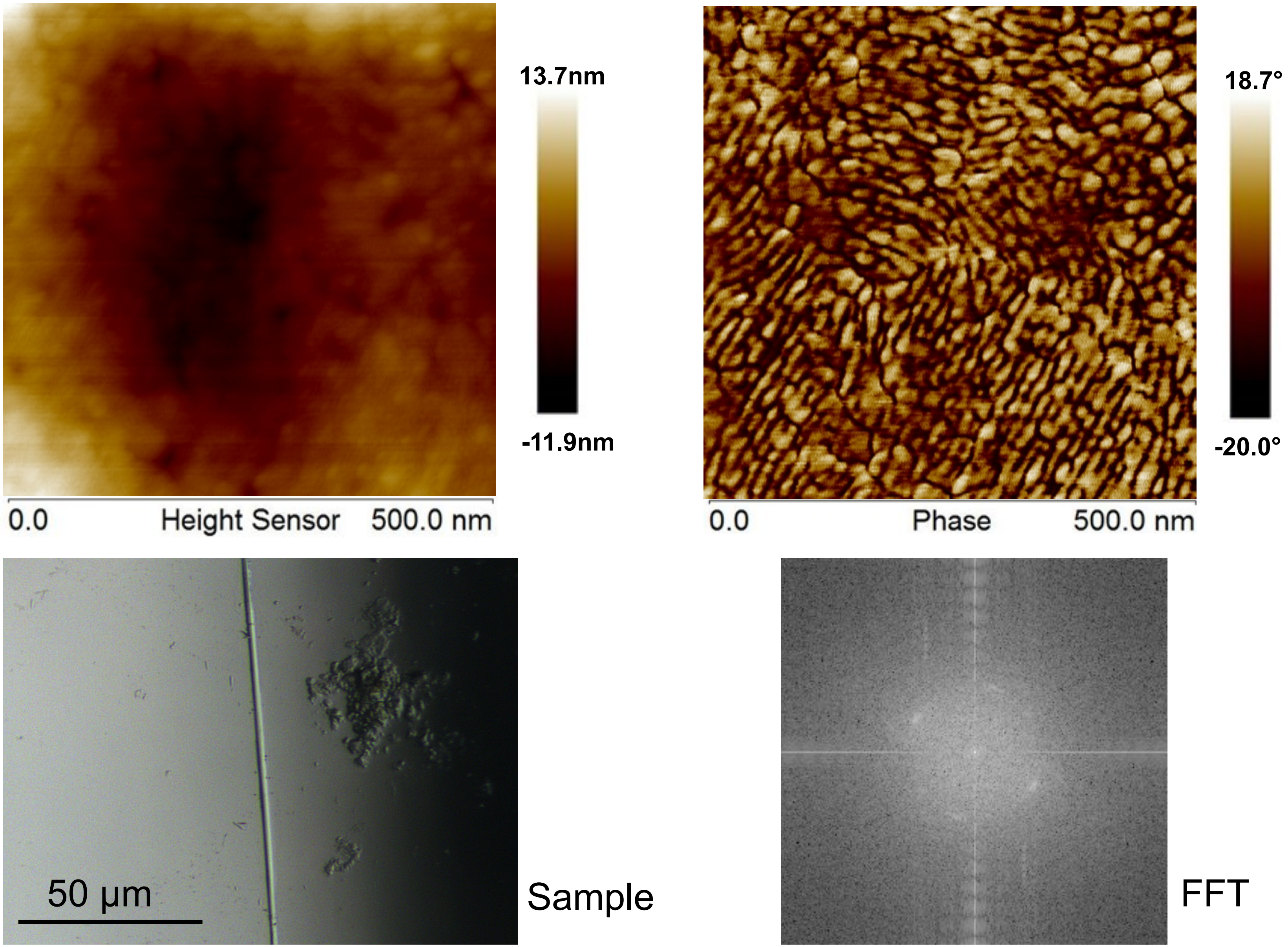

Figure S29 – Individual 2, Stretched, Fiber 5, Image 21, 2018/01/26

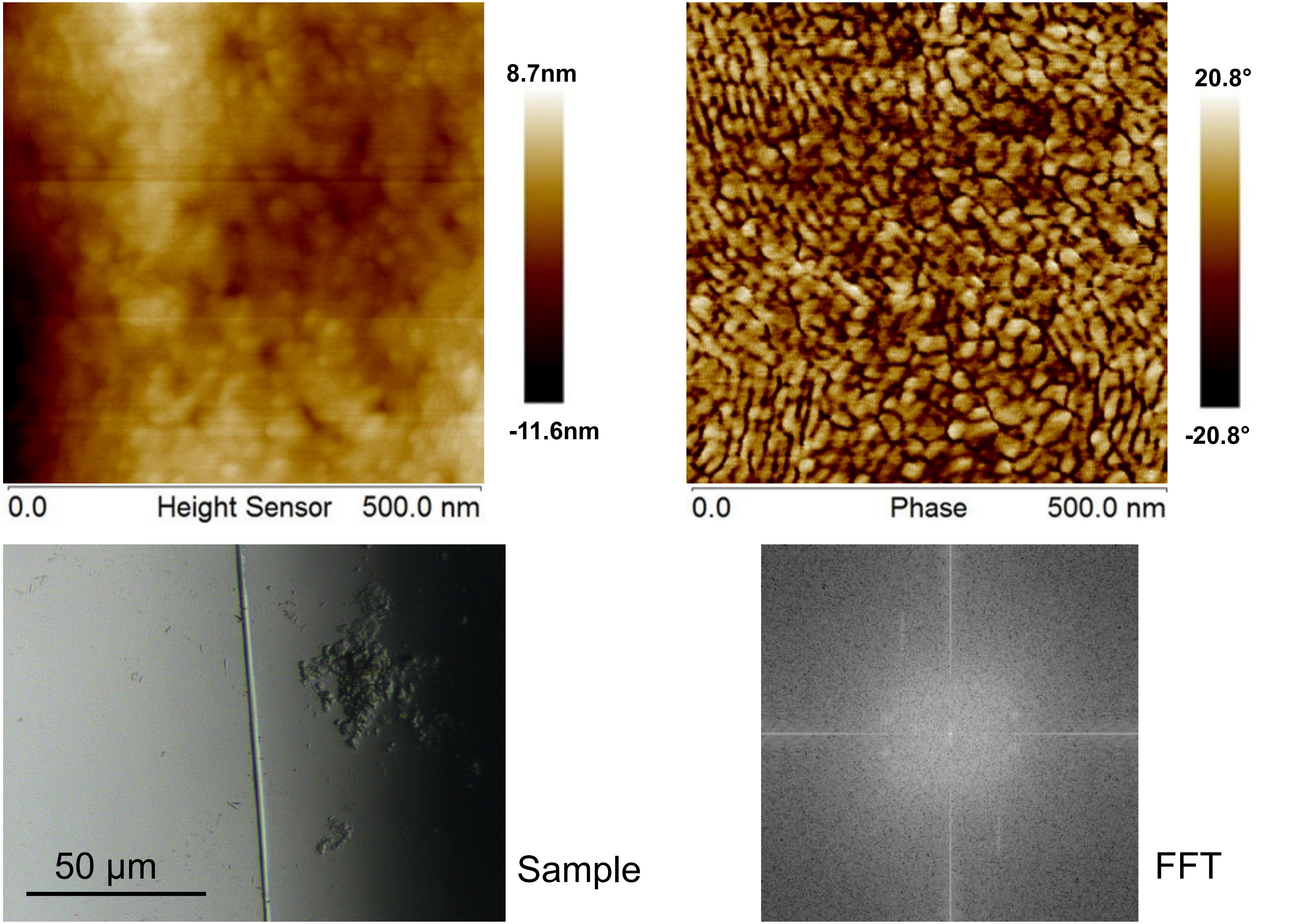

Figure S30 – Individual 2, Unstretched, Fiber 5, Image 22, 2018/01/26

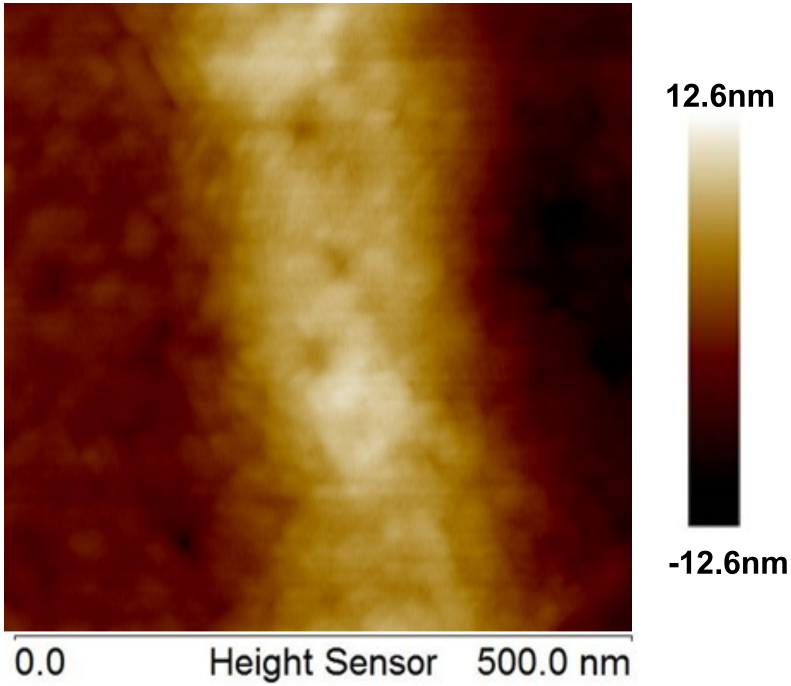

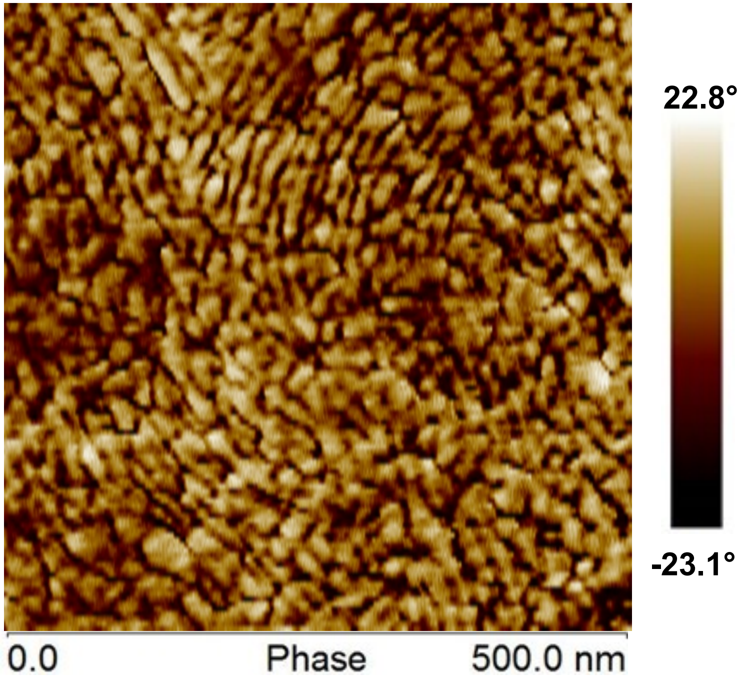

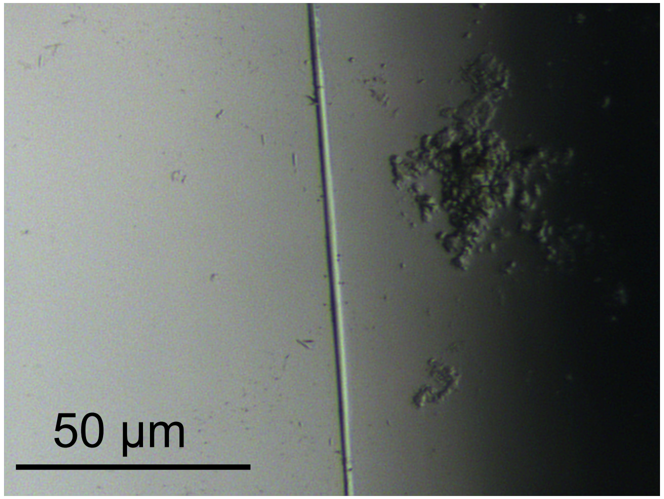

Sample

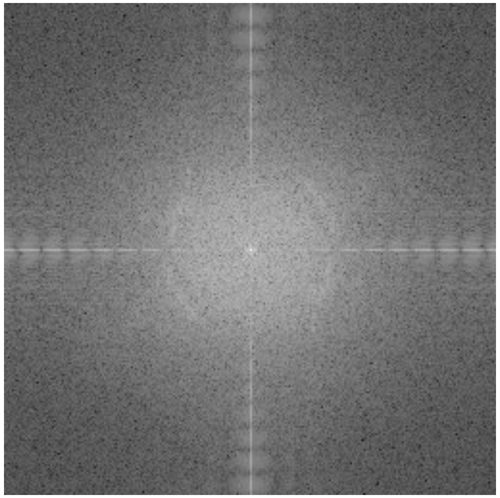

FFT

Figure S31 – Individual 2, Unstretched, Fiber 5, Image 23, 2018/01/26

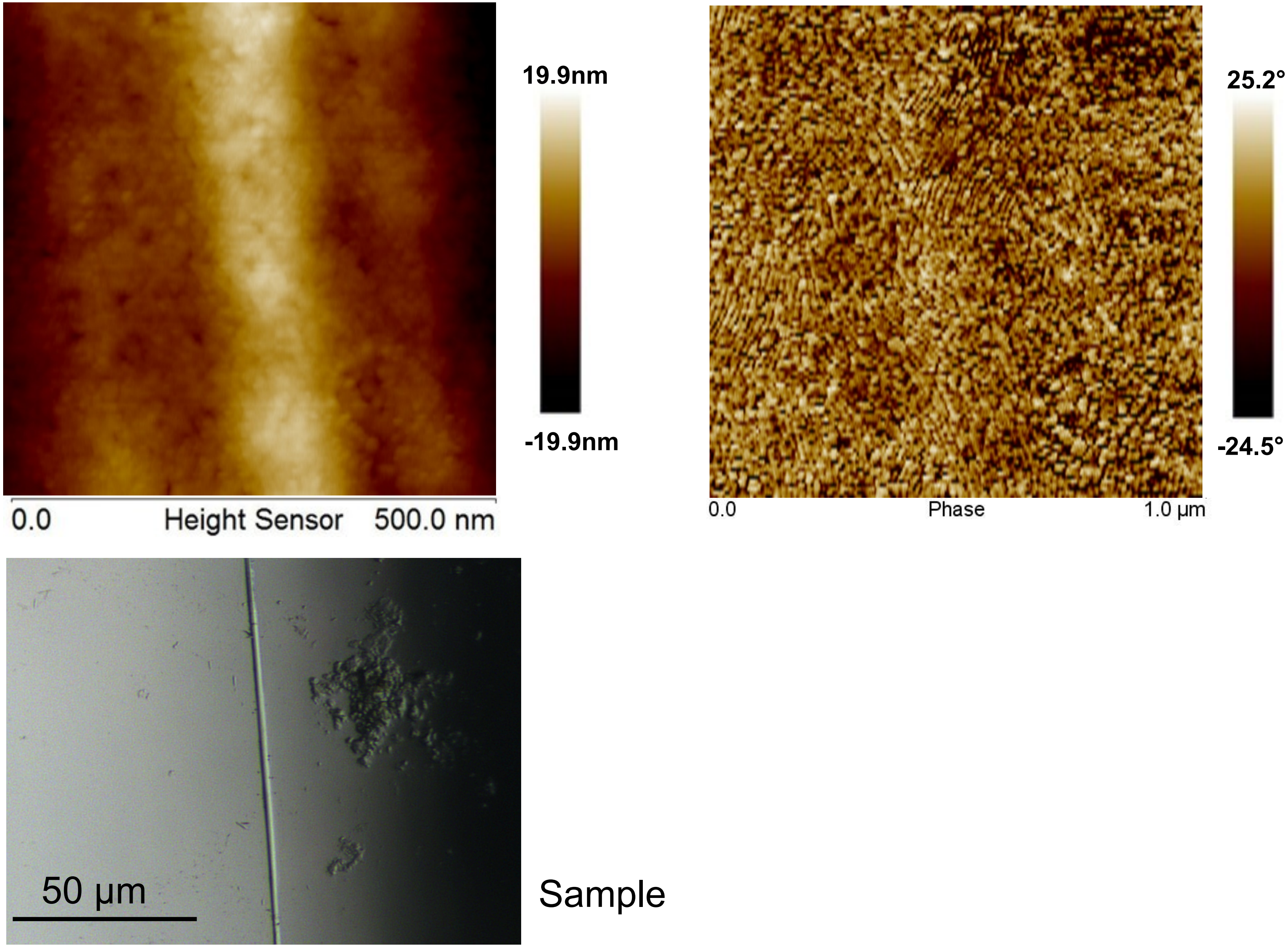

Figure S32 – Individual 3, Unstretched, Fiber 6, Image 24, 2018/01/26

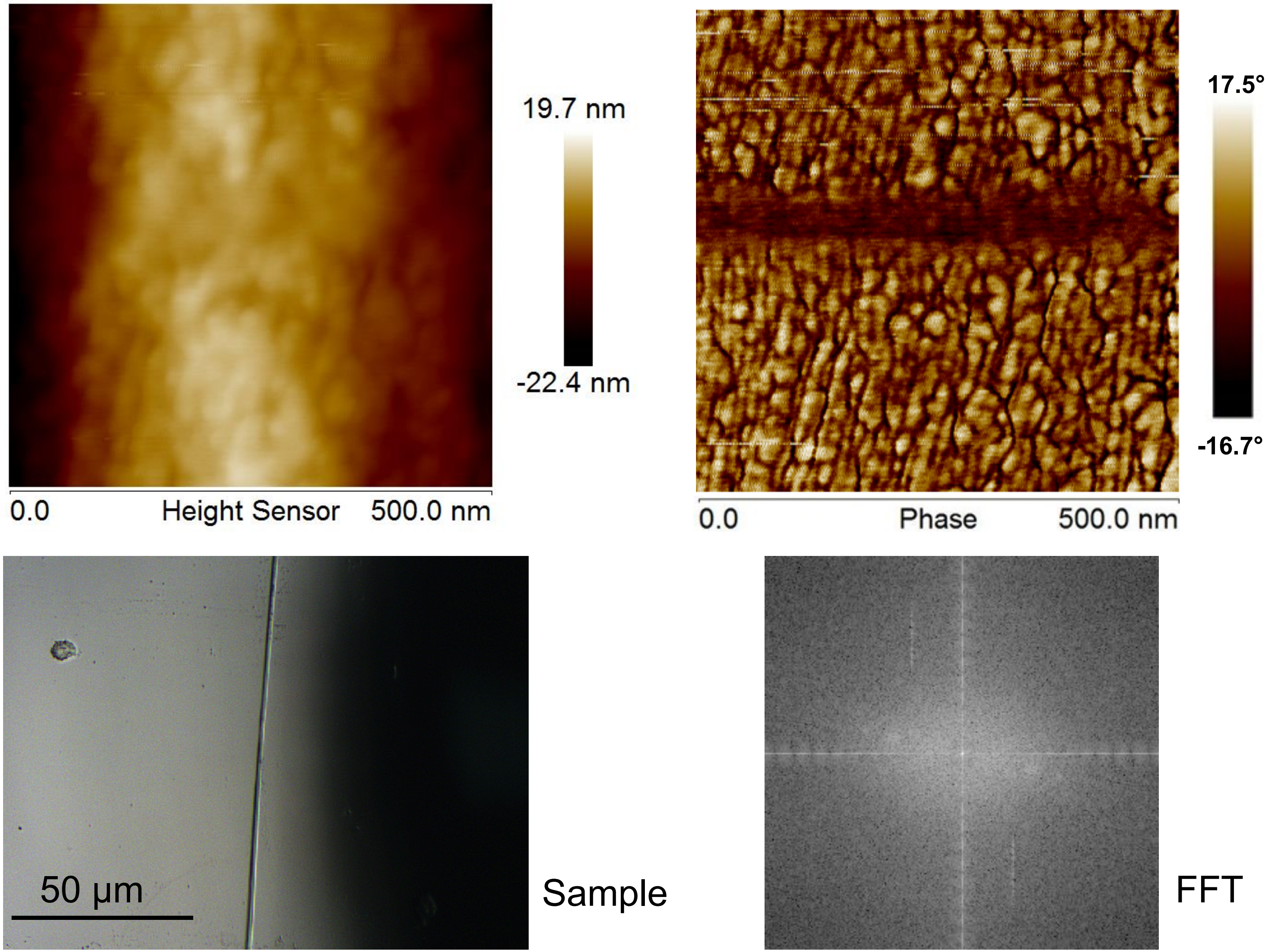

Figure S33 – Individual 3, Unstretched, Fiber 6, Image 25, 2018/01/26

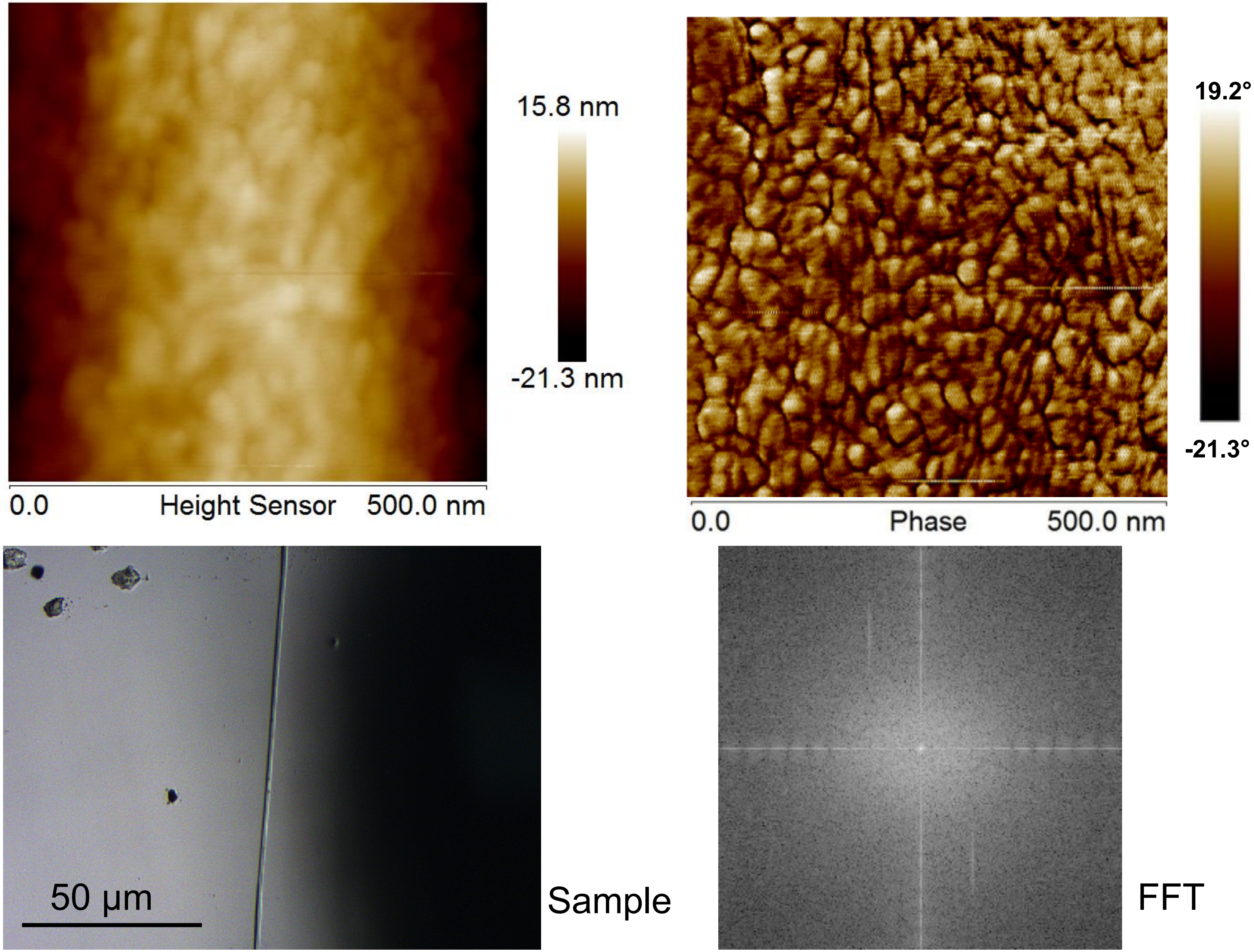

Figure S34 – Individual 4, Stretched, Fiber 7, Image 26, 2018/02/08

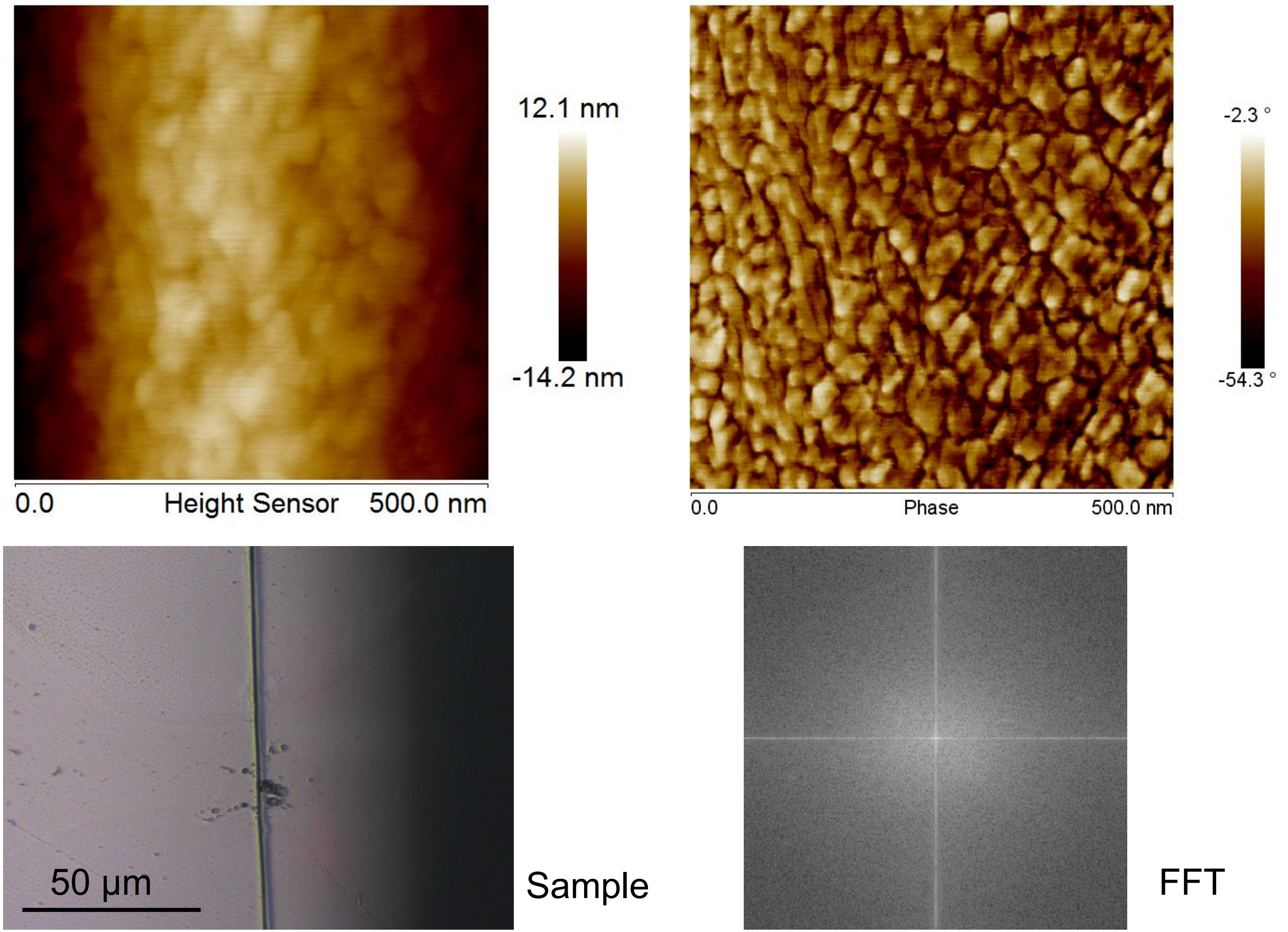

Figure S35 – Individual 4, Stretched, Fiber 7, Image 27, 2018/02/08

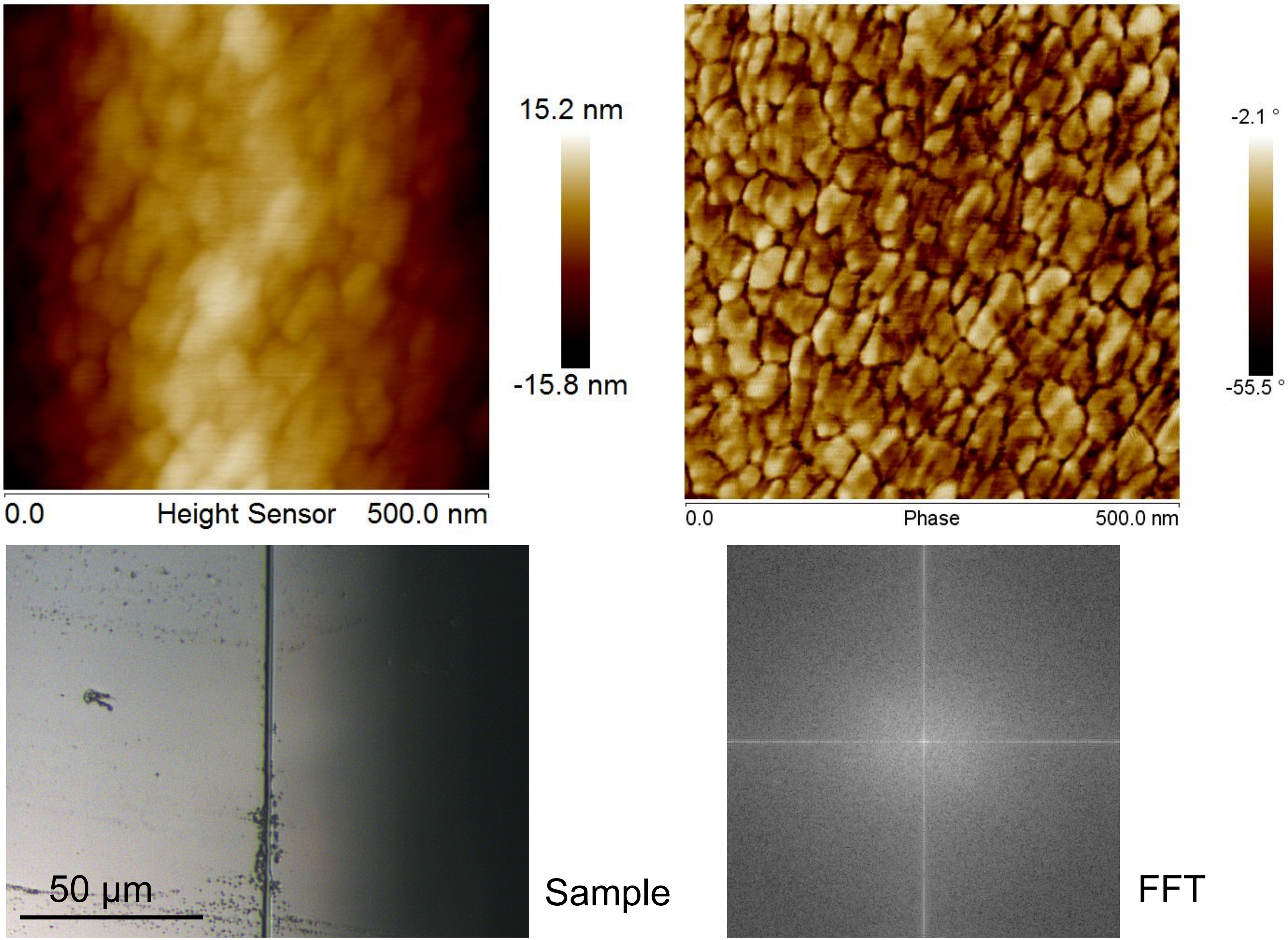

Figure S36 – Individual 4, Stretched, Fiber 7, Image 28, 2018/02/08

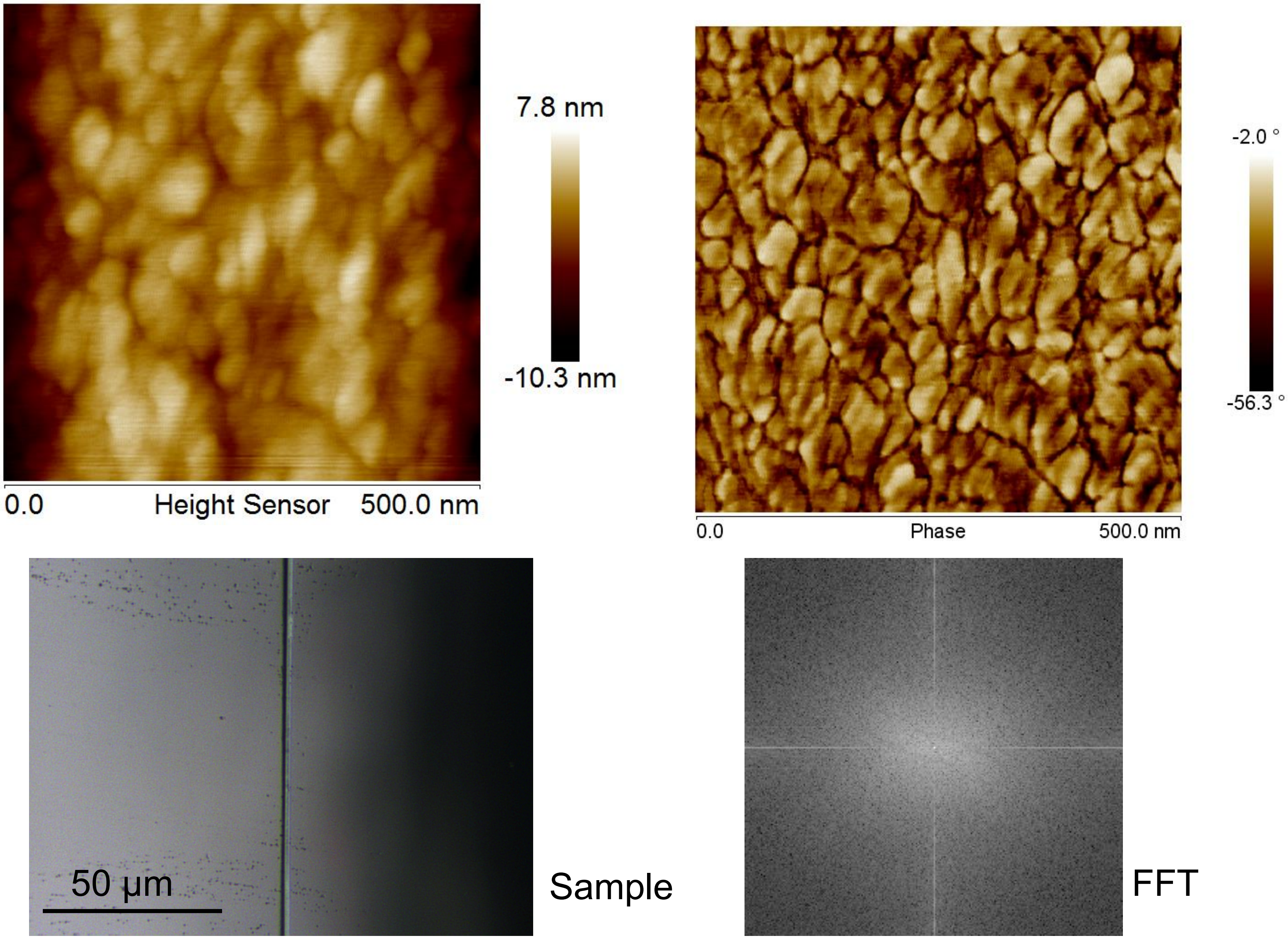

Figure S37 – Individual 4, Stretched, Fiber 7, Image 29, 2018/02/08

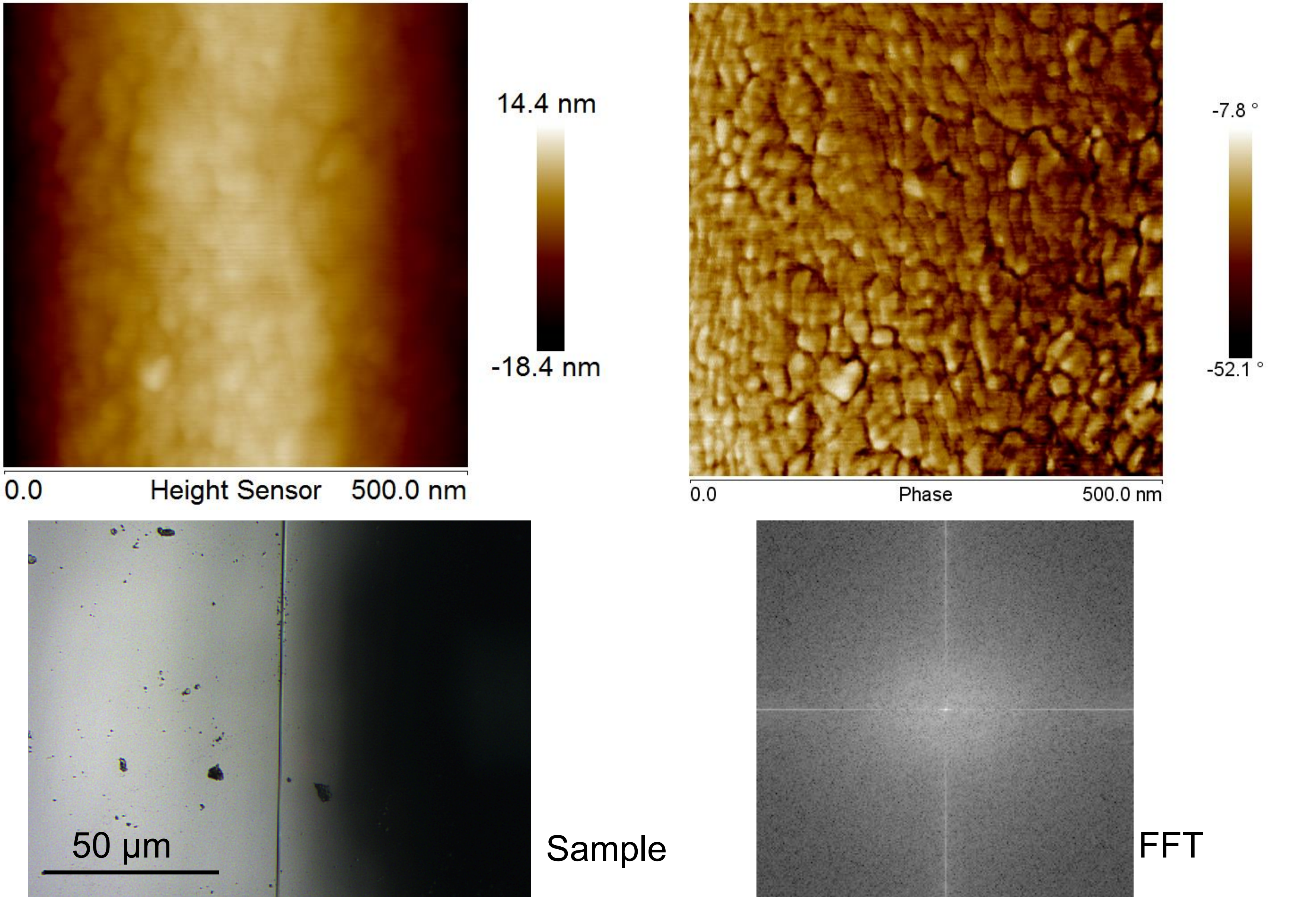

Figure S38 – Individual 4, Stretched, Fiber 7, Image 30, 2018/02/08

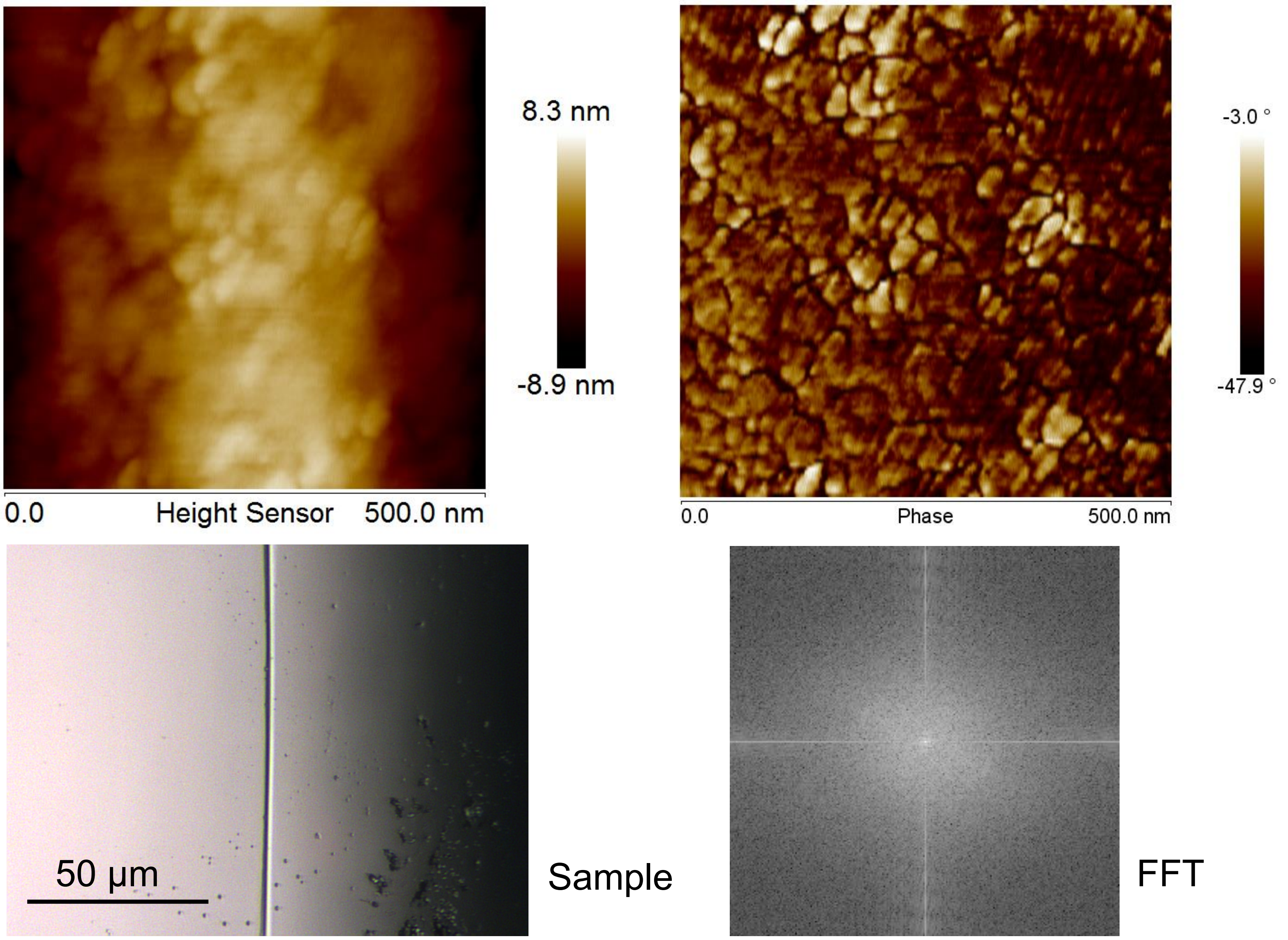

Figure S39 – Individual 5, Stretched, Fiber 8, Image 31, 2018/02/08

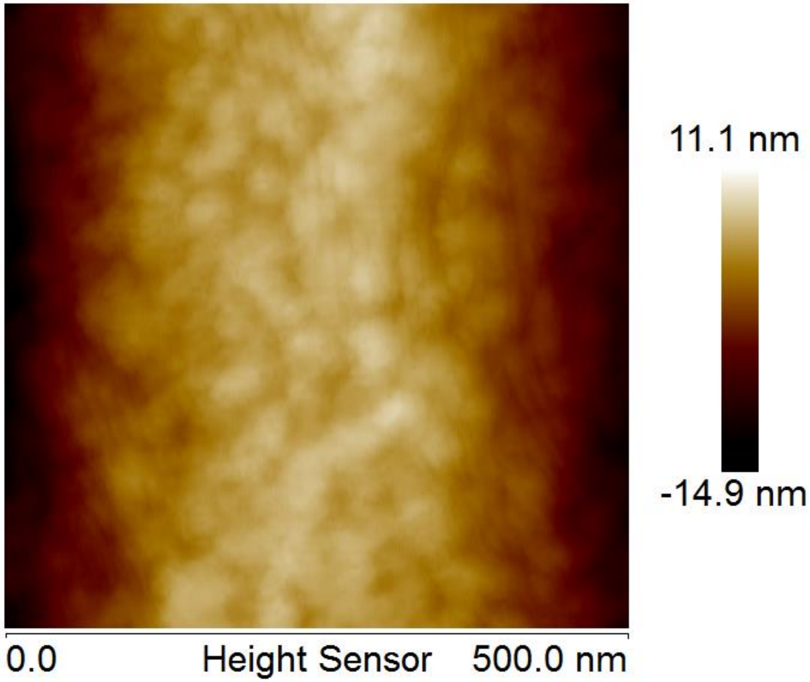

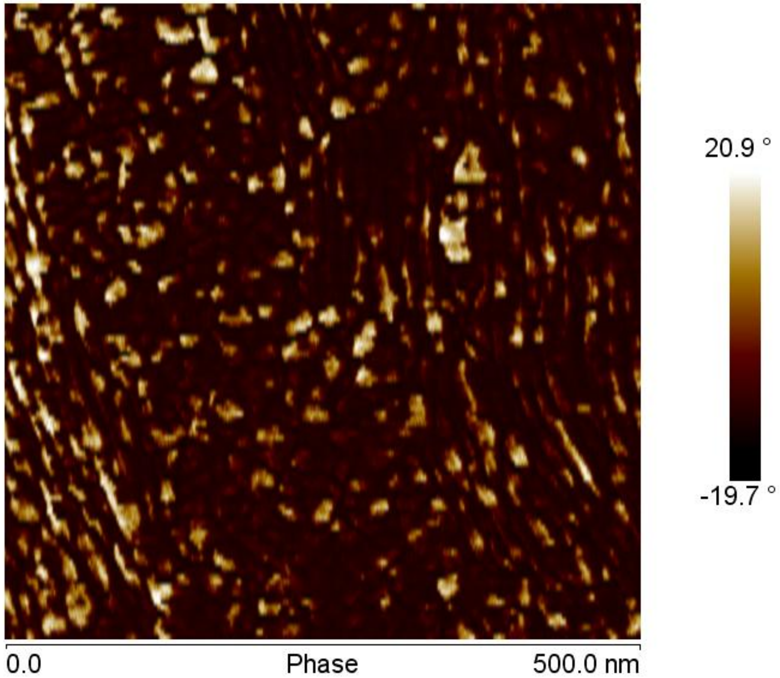

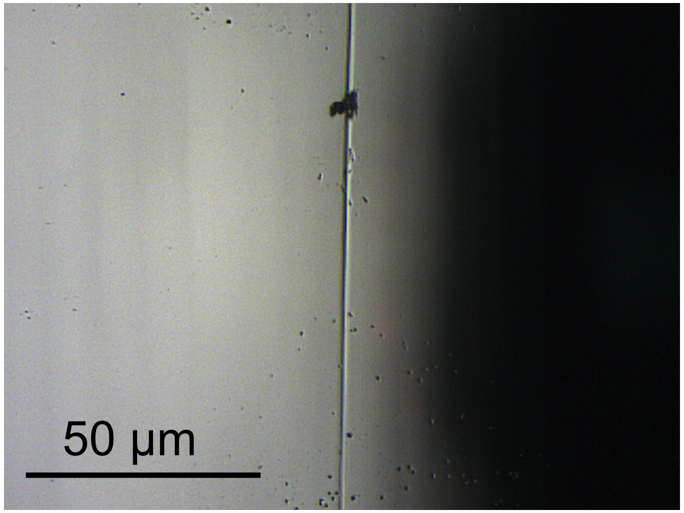

Sample

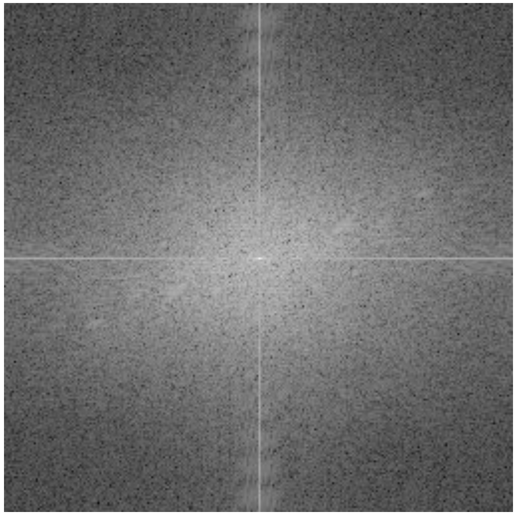

FFT

Figure S40 – Individual 5, Stretched, Fiber 8, Image 32, 2018/02/08

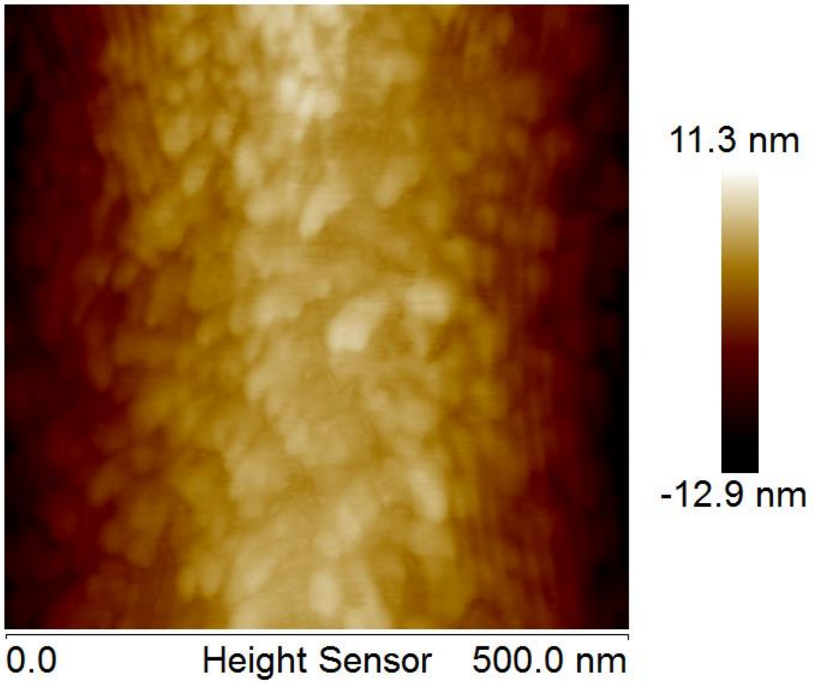

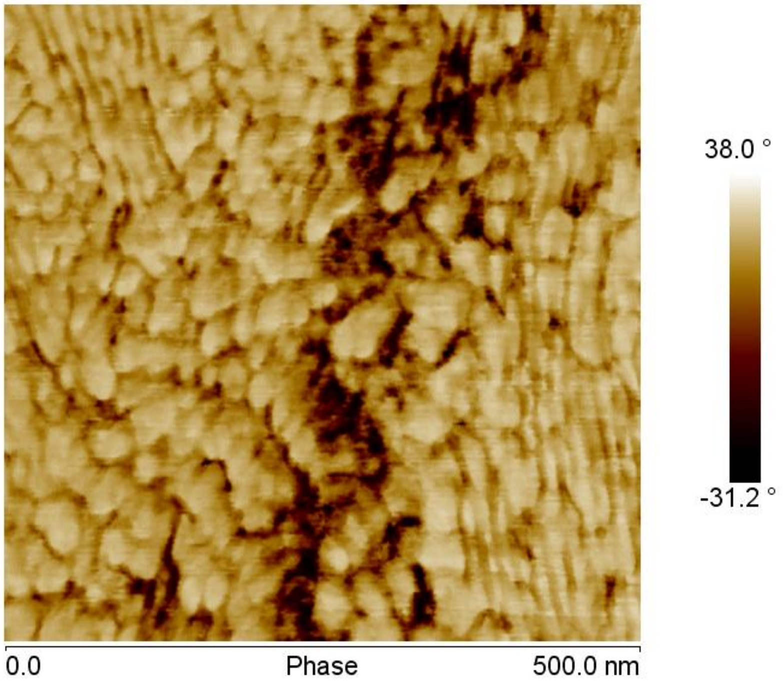

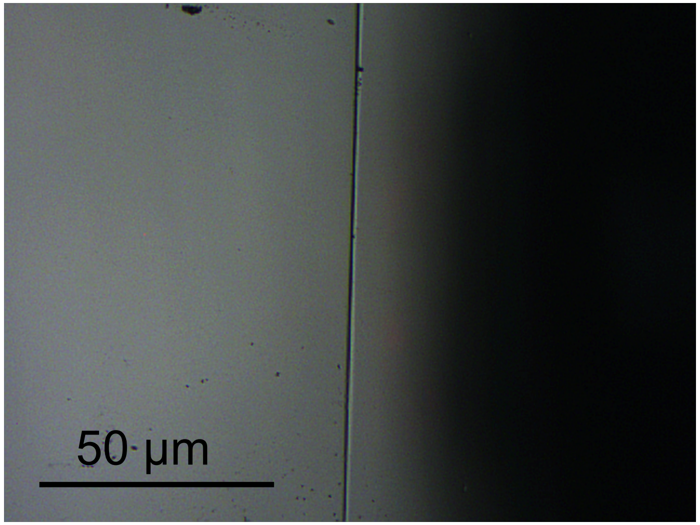

Sample

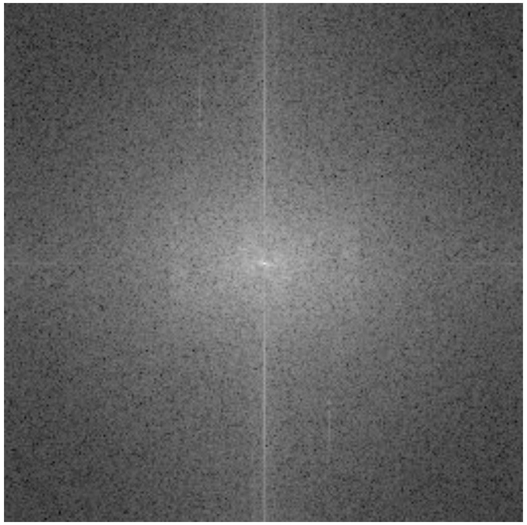

FFT

Figure S41 – Individual 5, Stretched, Fiber 8, Image 33, 2018/02/08

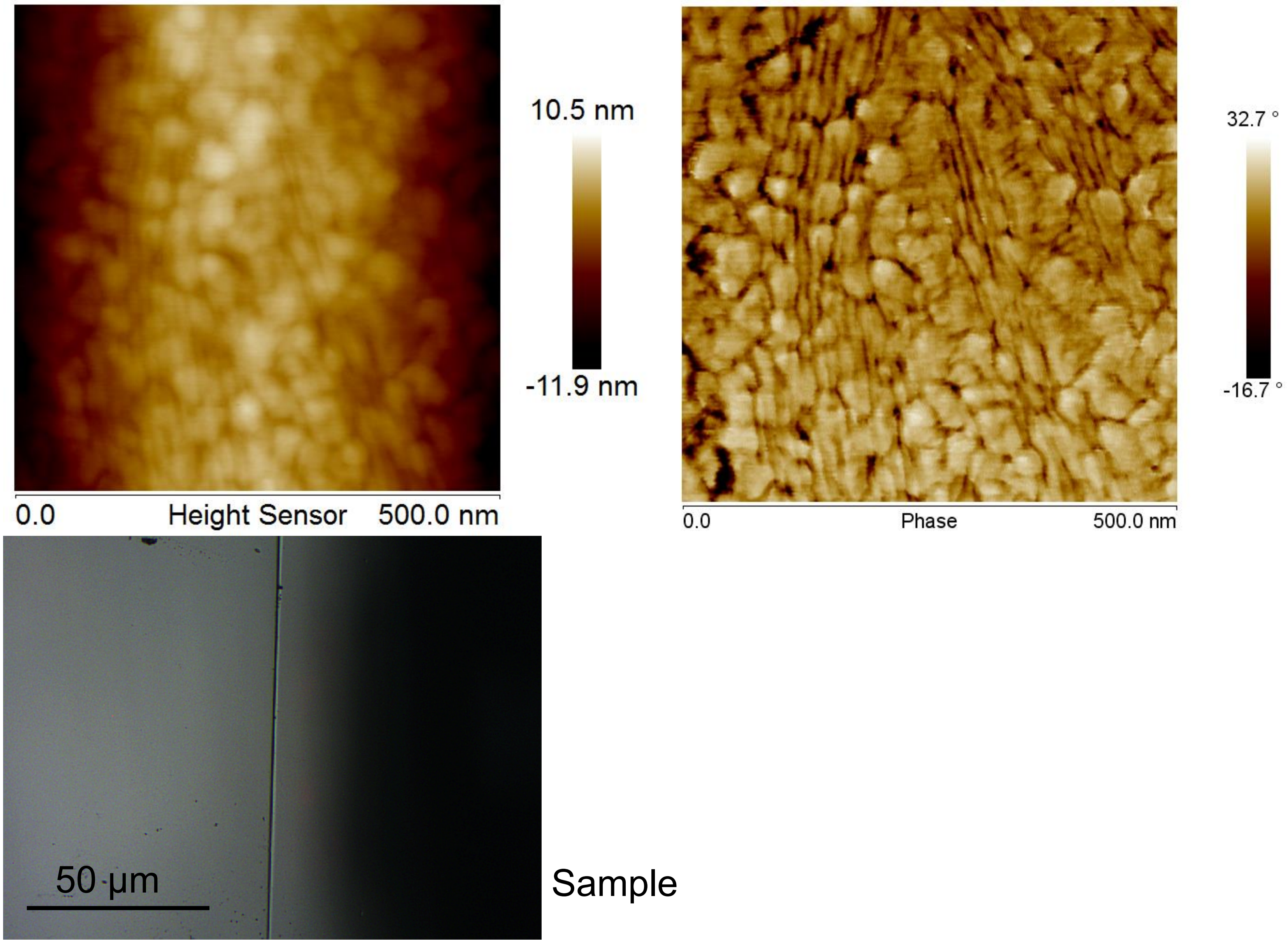

Figure S42 – Individual 4, Unstretched, Fiber 9, Image 34, 2018/02/08

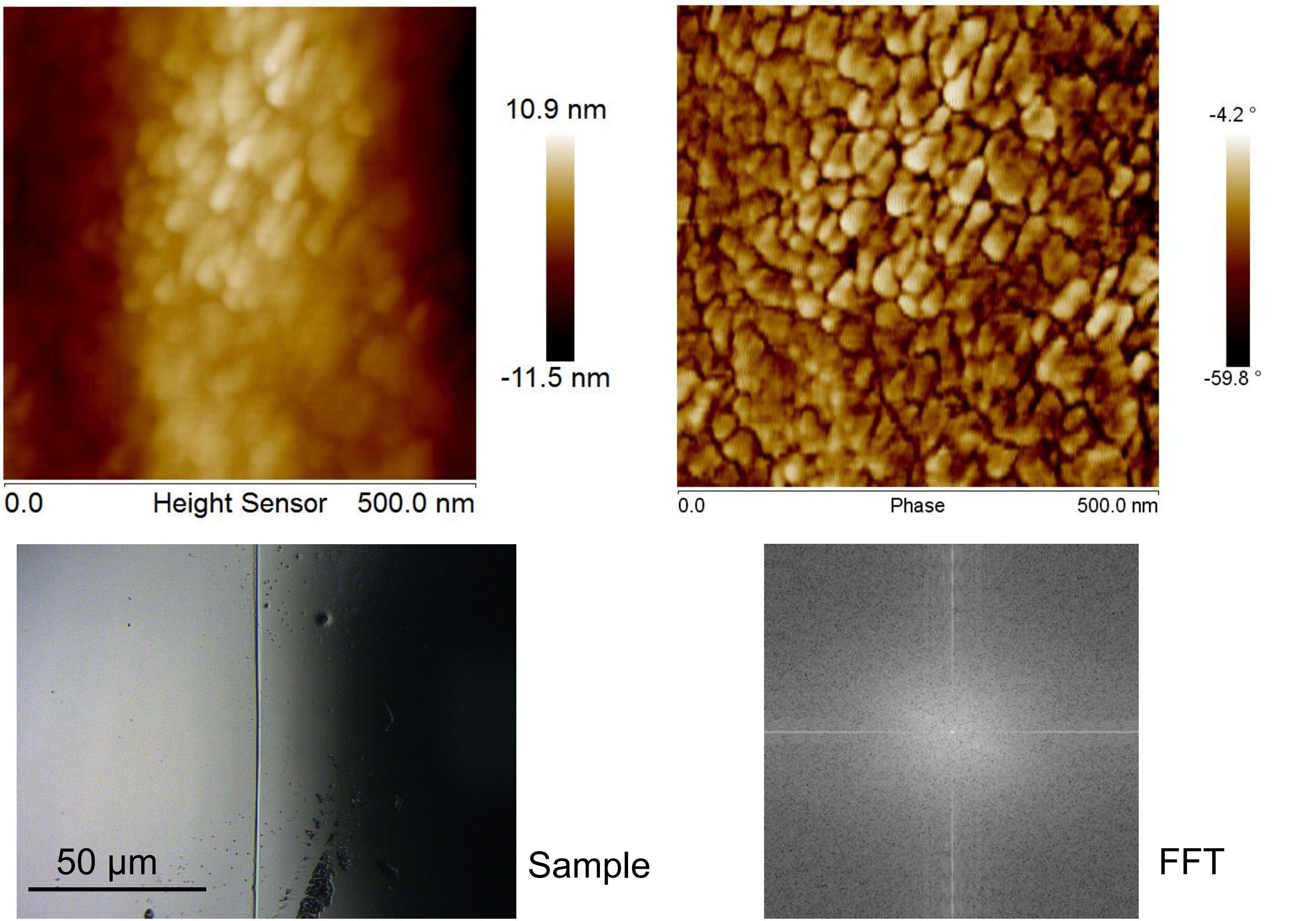

Figure S43 – Individual 4, Unstretched, Fiber 9, Image 35, 2018/02/08

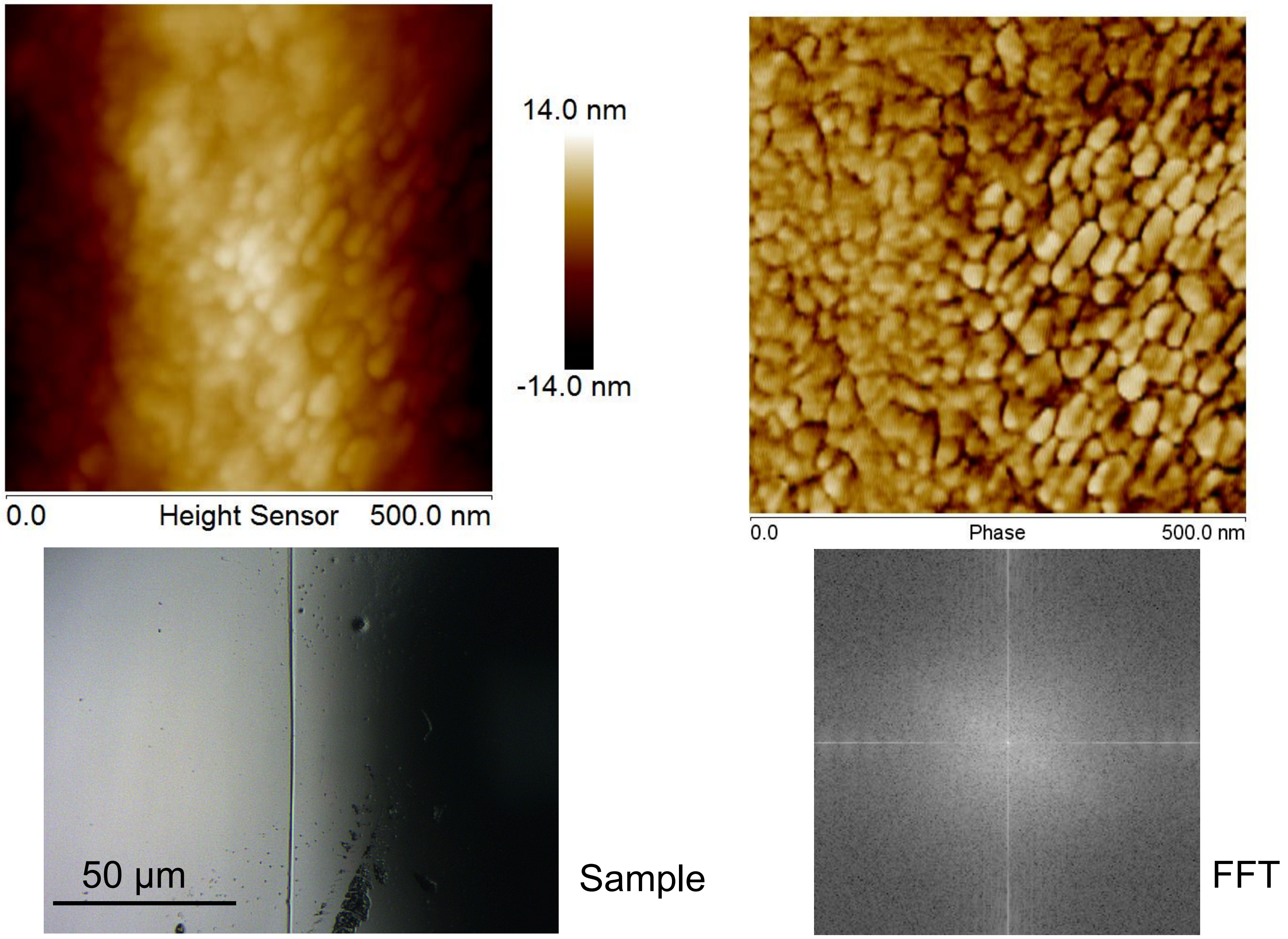

Figure S44 – Individual 4, Unstretched, Fiber 9, Image 36, 2018/02/08

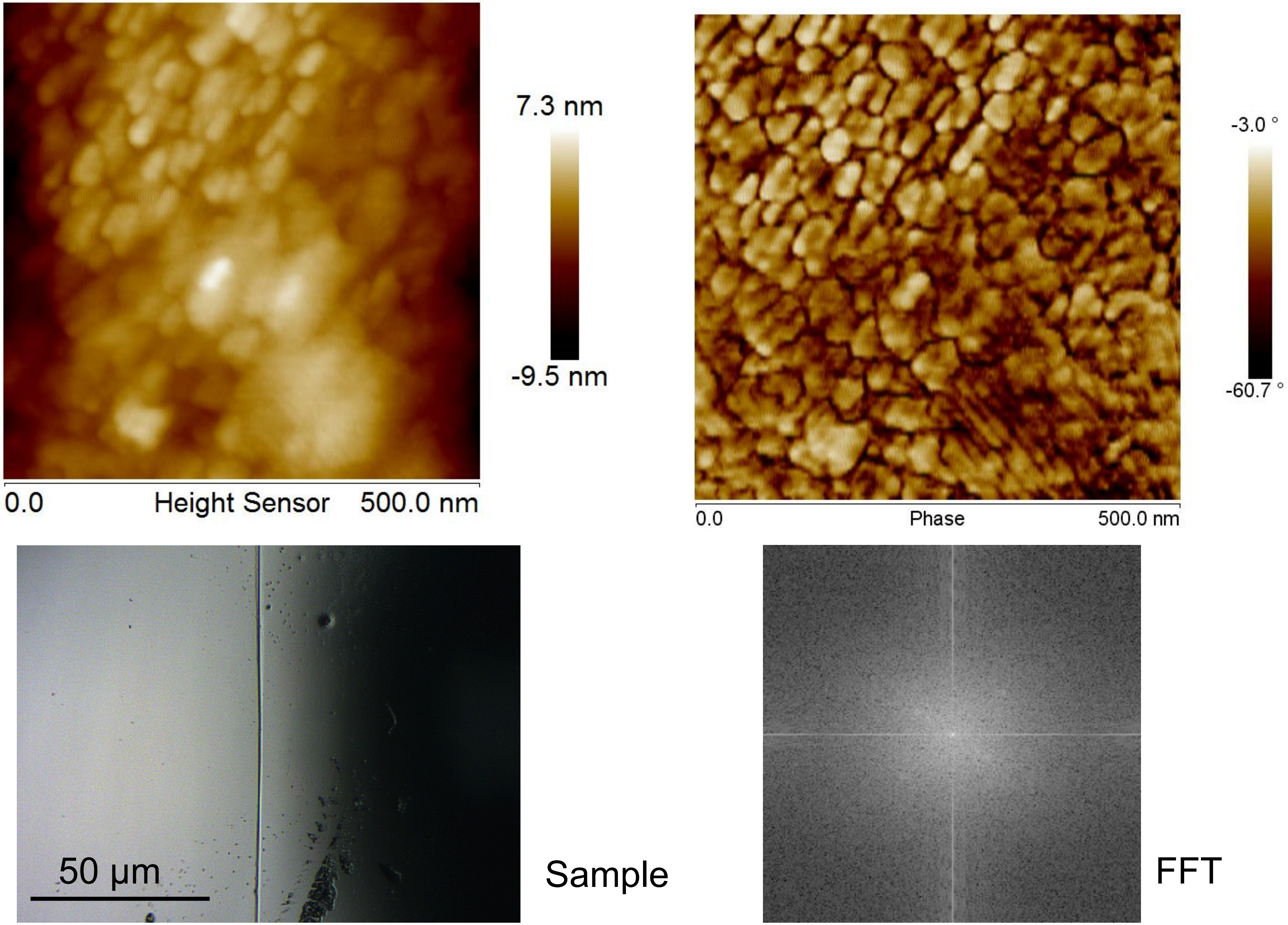

Figure S45 – Individual 5, Unstretched, Fiber 10, Image 37, 2018/02/08

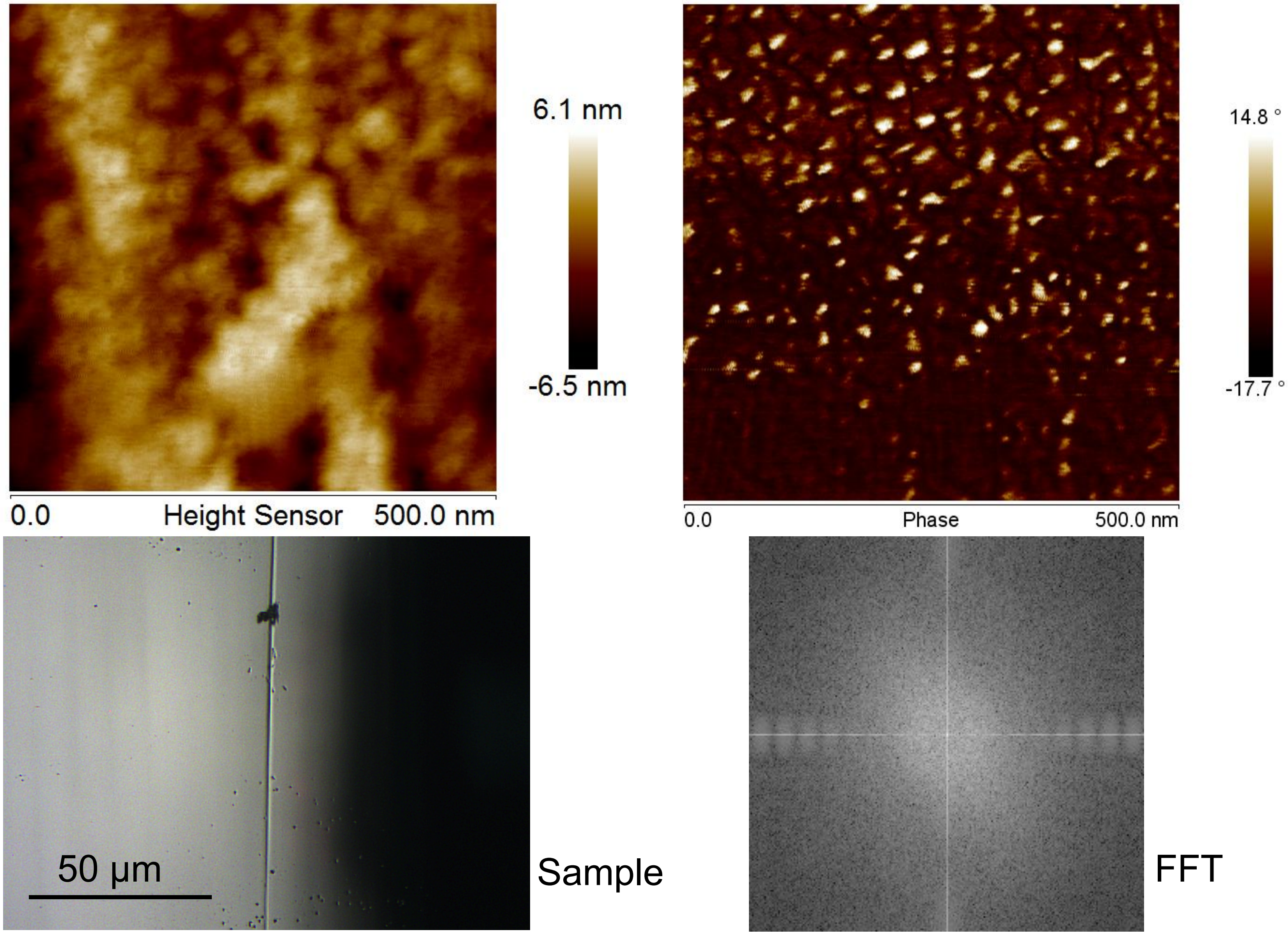

Figure S46 – Individual 5, Unstretched, Fiber 10, Image 38, 2018/02/08

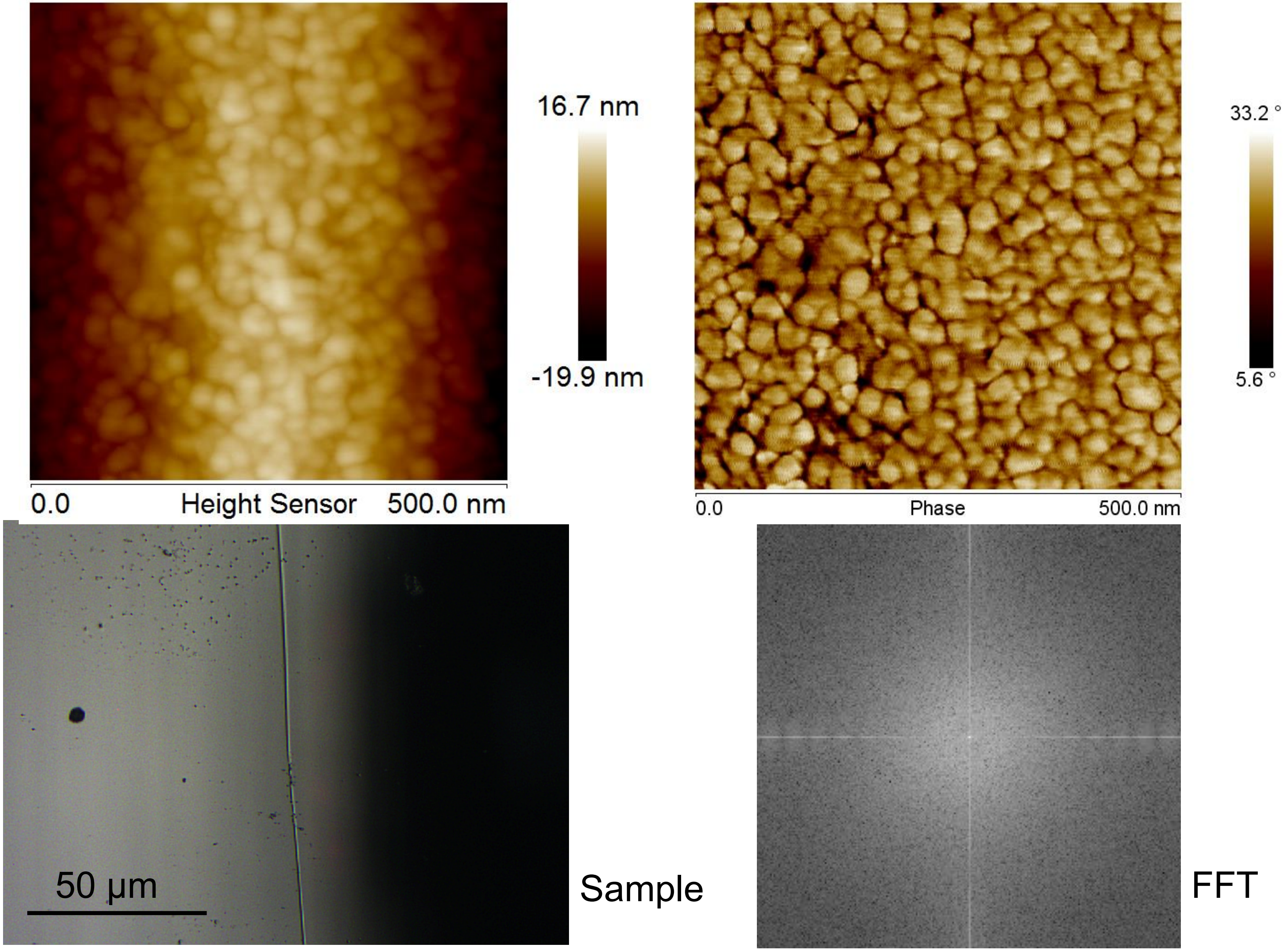

Figure S47– Individual 5, Unstretched, Fiber 10, Image 39, 2018/02/08

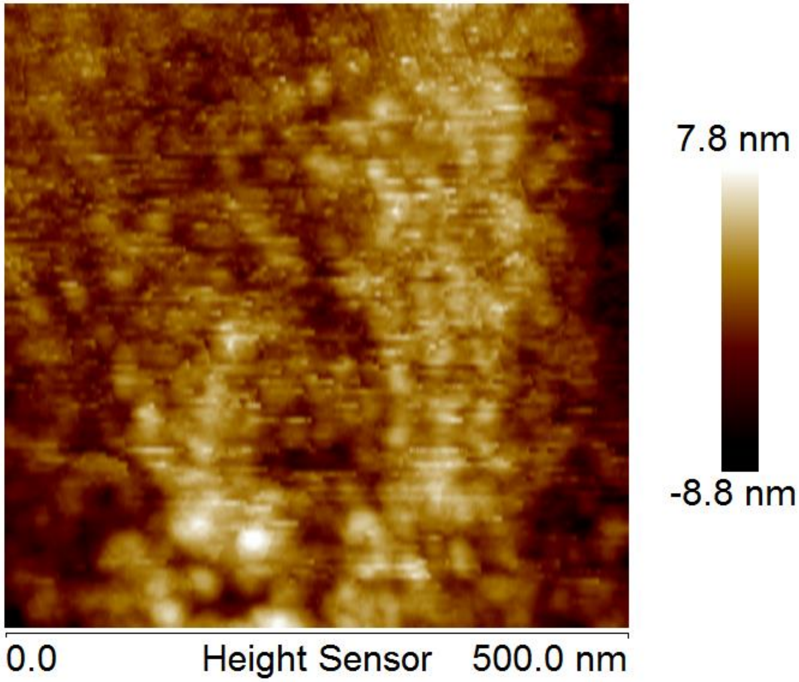

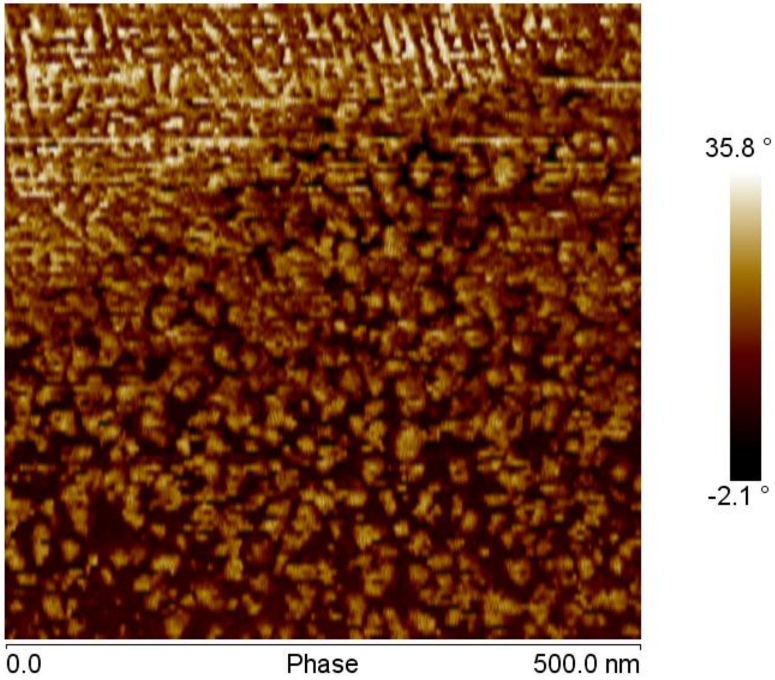

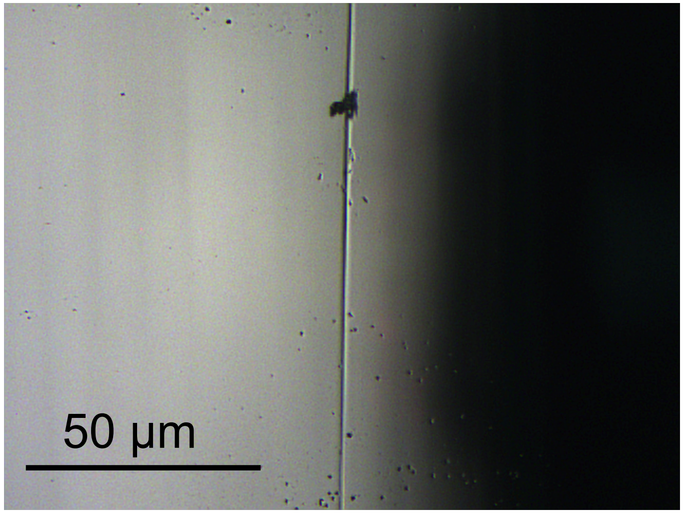

Sample

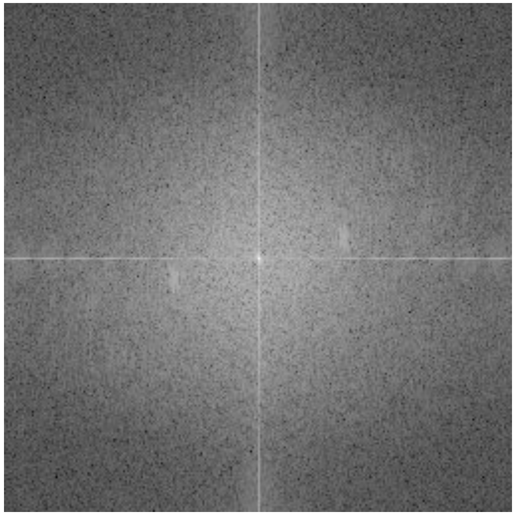

FFT

Figure S48 – Individual 5, Unstretched, Fiber 10, Image 40, 2018/02/08

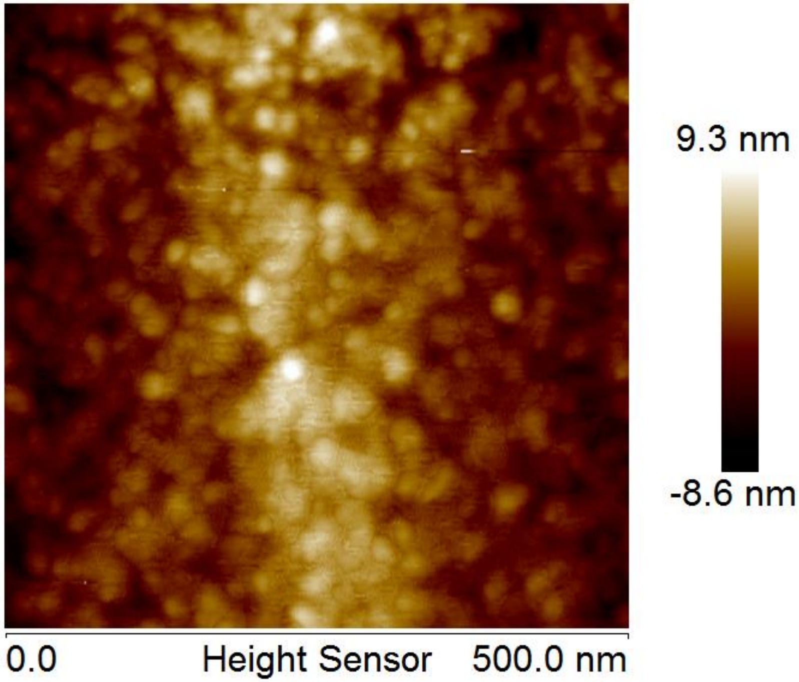

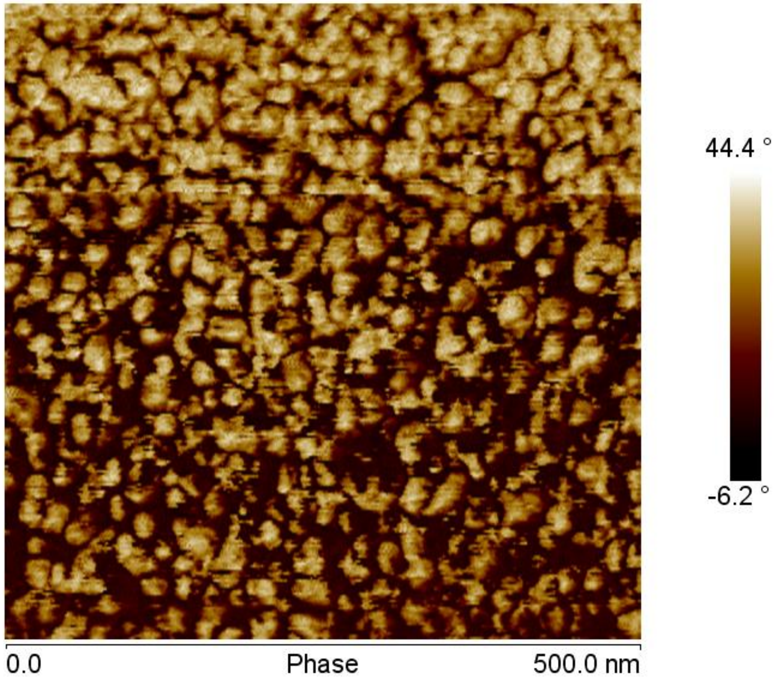

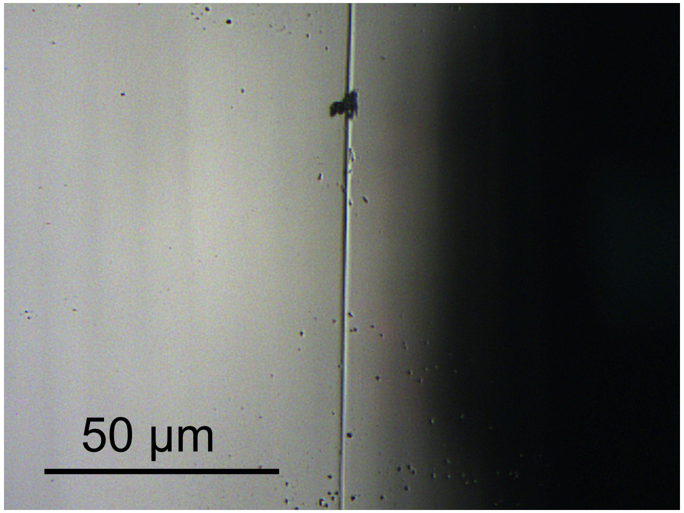

Sample

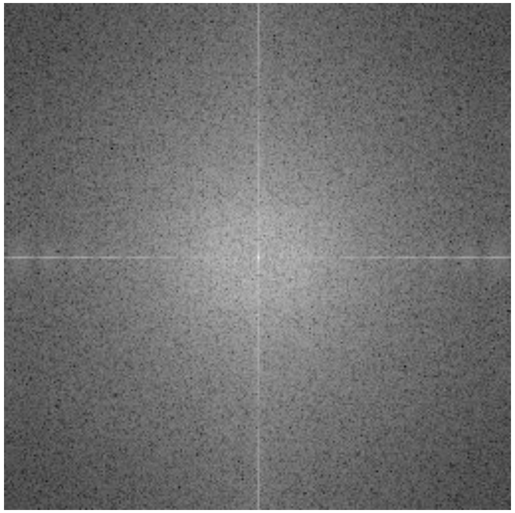

FFT

Figure S49 – Individual 5, Unstretched, Fiber 10, Image 41, 2018/02/08

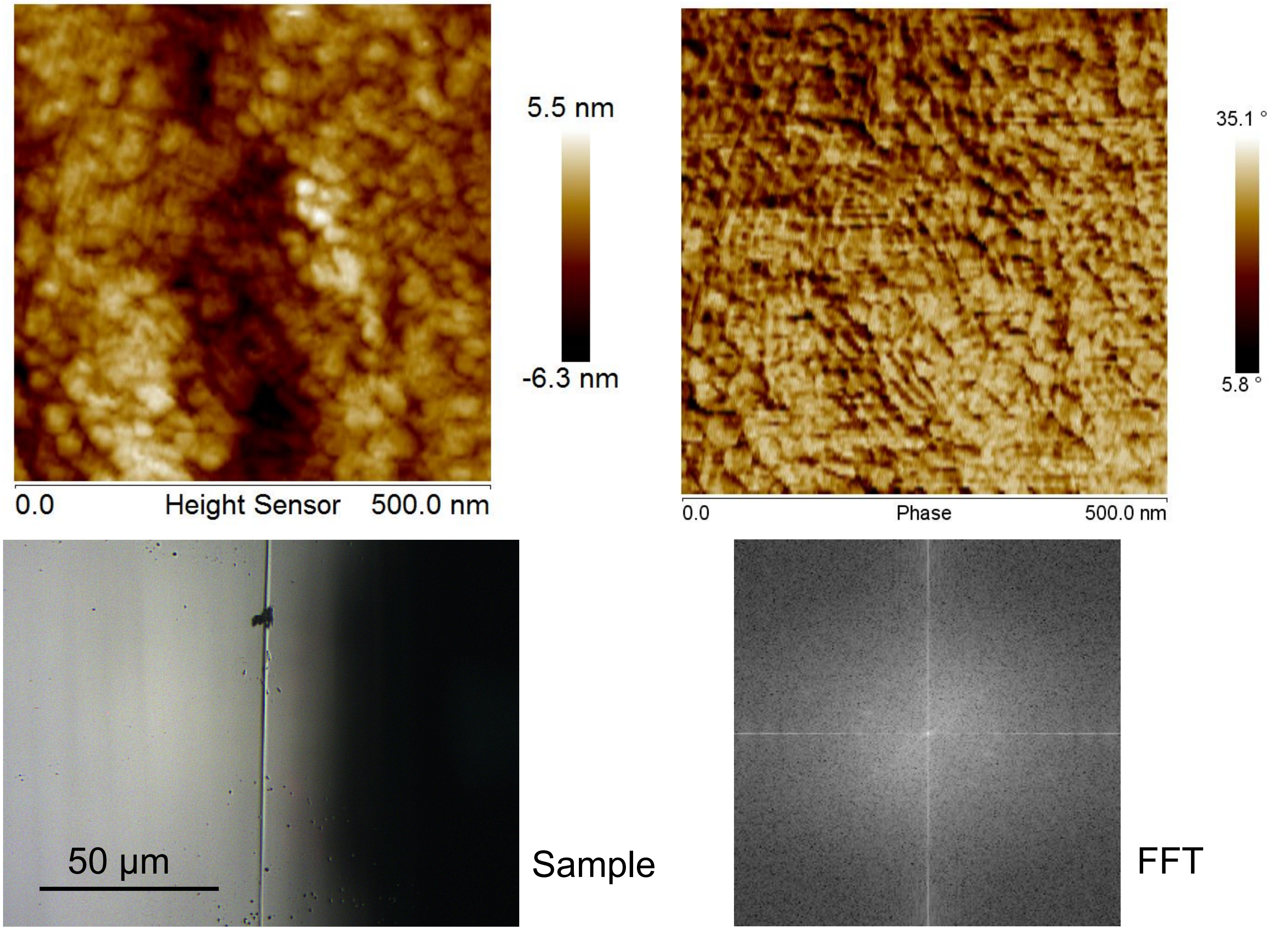

Figure S50 – Individual 5, Unstretched, Fiber 10, Image 42, 2018/02/08

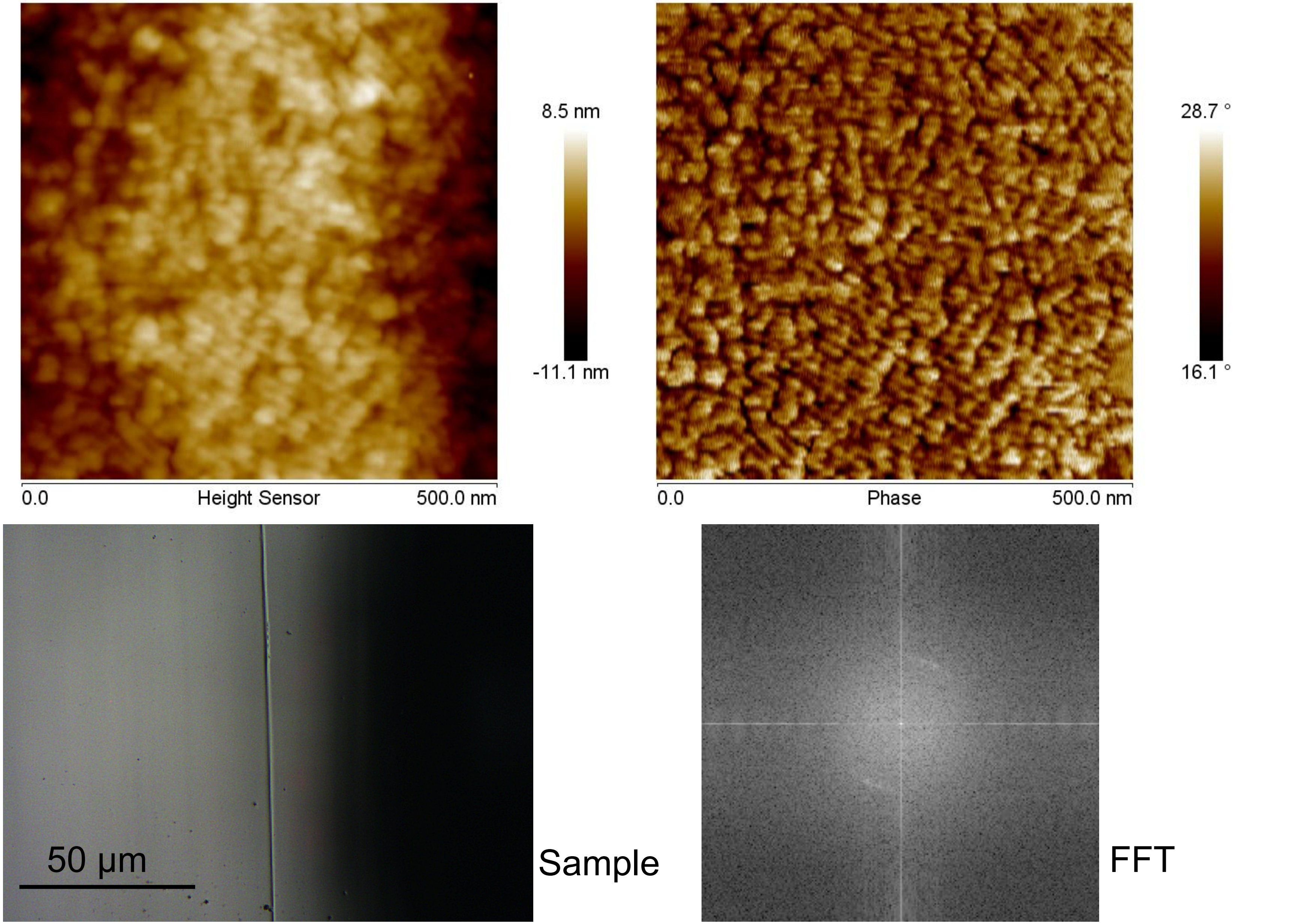

Figure S51 – Individual 5, Unstretched, Fiber 10, Image 43, 2018/02/08

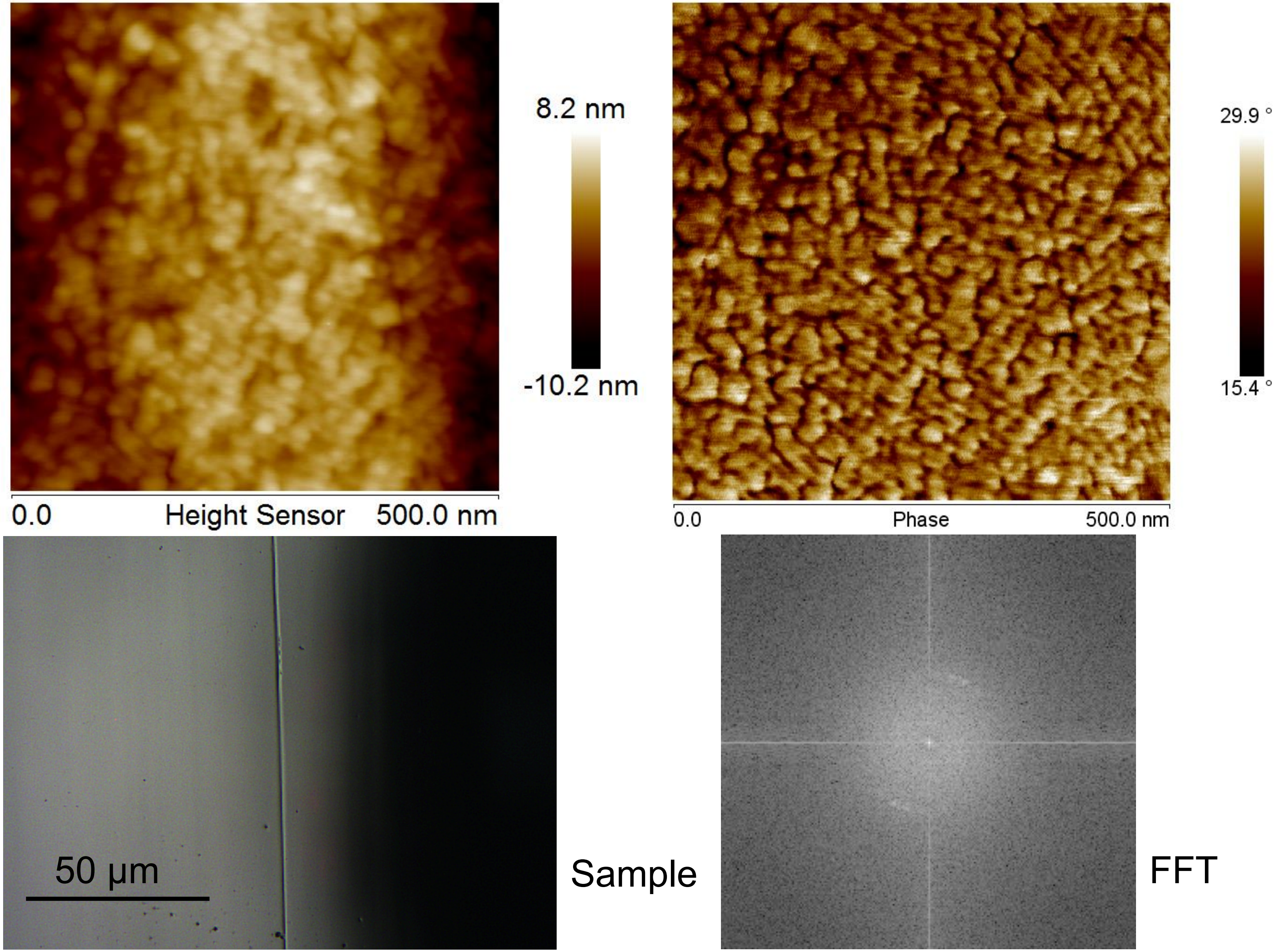

Figure S52 – Individual 5, Unstretched, Fiber 10, Image 44, 2018/02/08

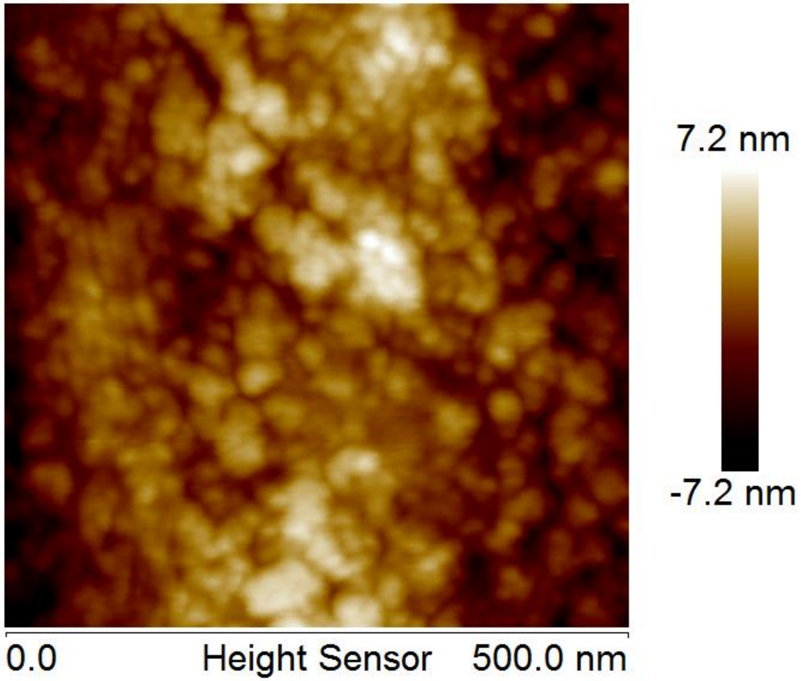

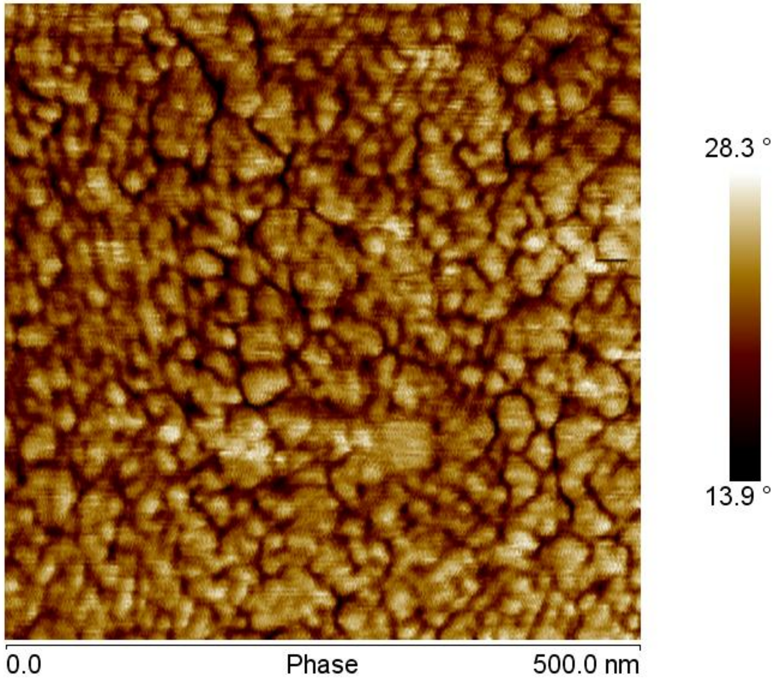

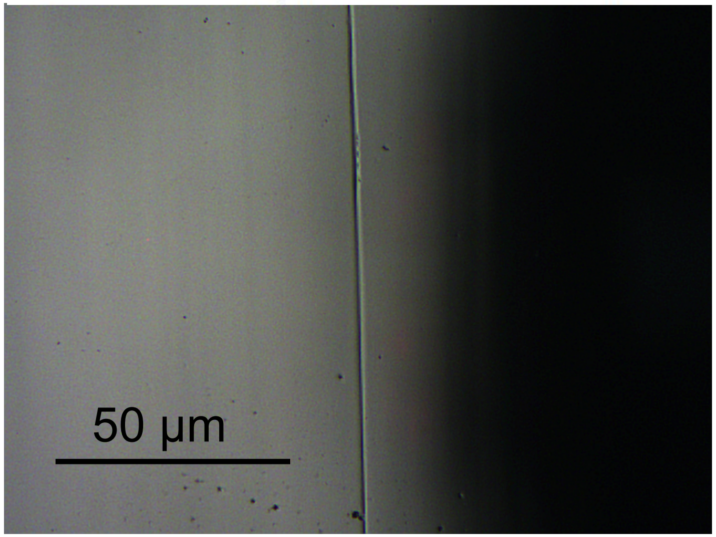

Sample

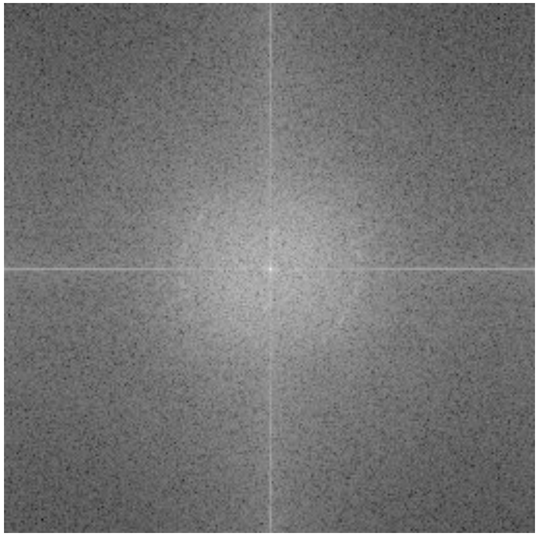

FFT

Figure S53 – Individual 5, Unstretched, Fiber 10, Image 45, 2018/02/08

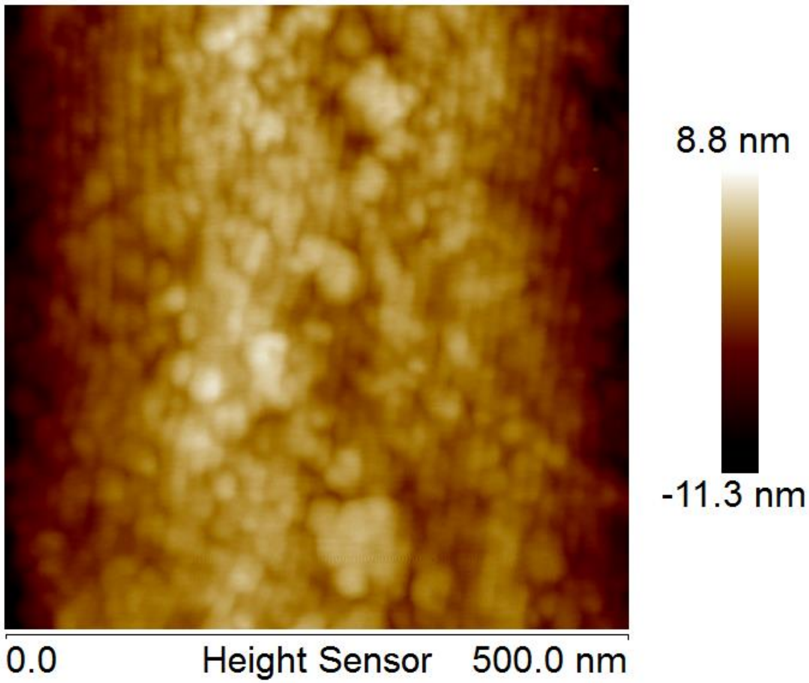

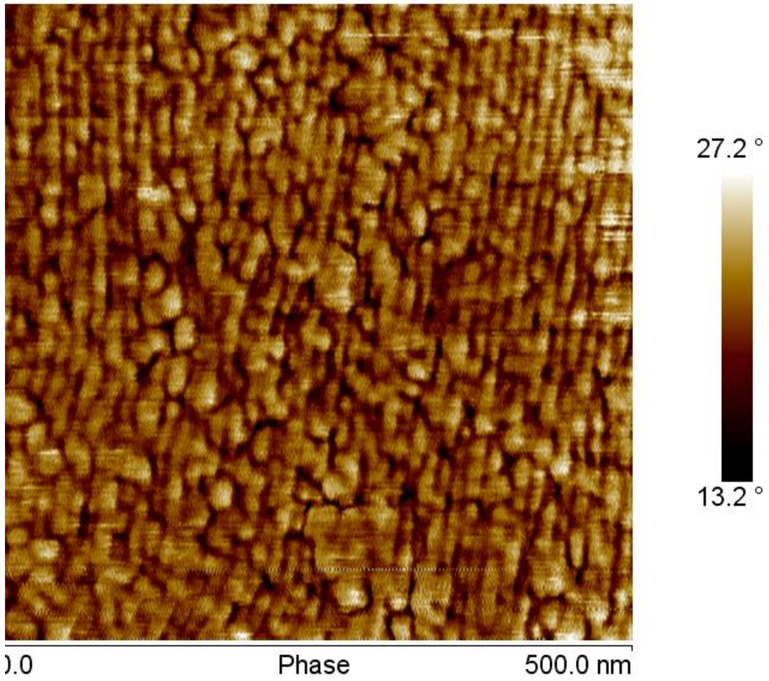

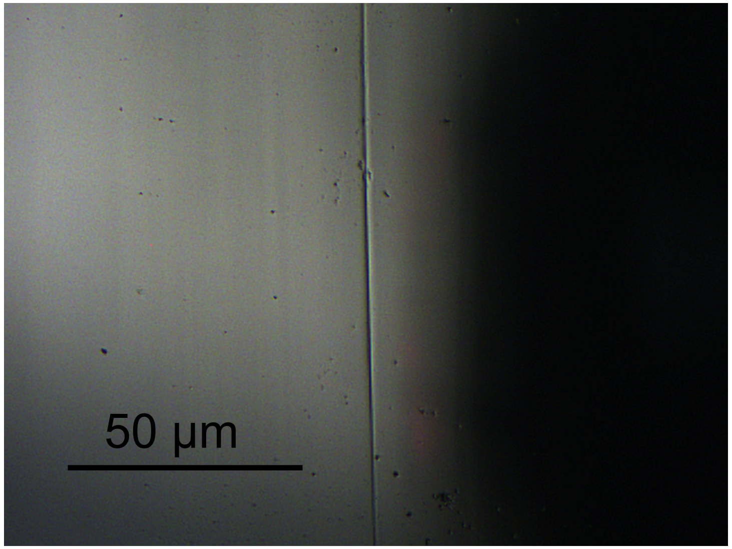

Sample

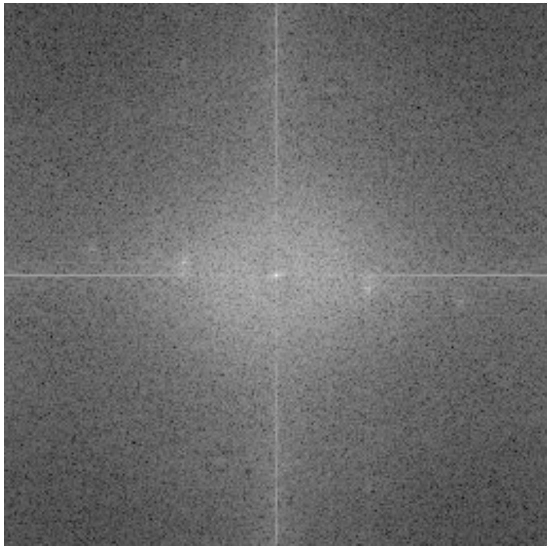

FFT

Figure S54 – Individual 5, Unstretched, Fiber 10, Image 46, 2018/02/08

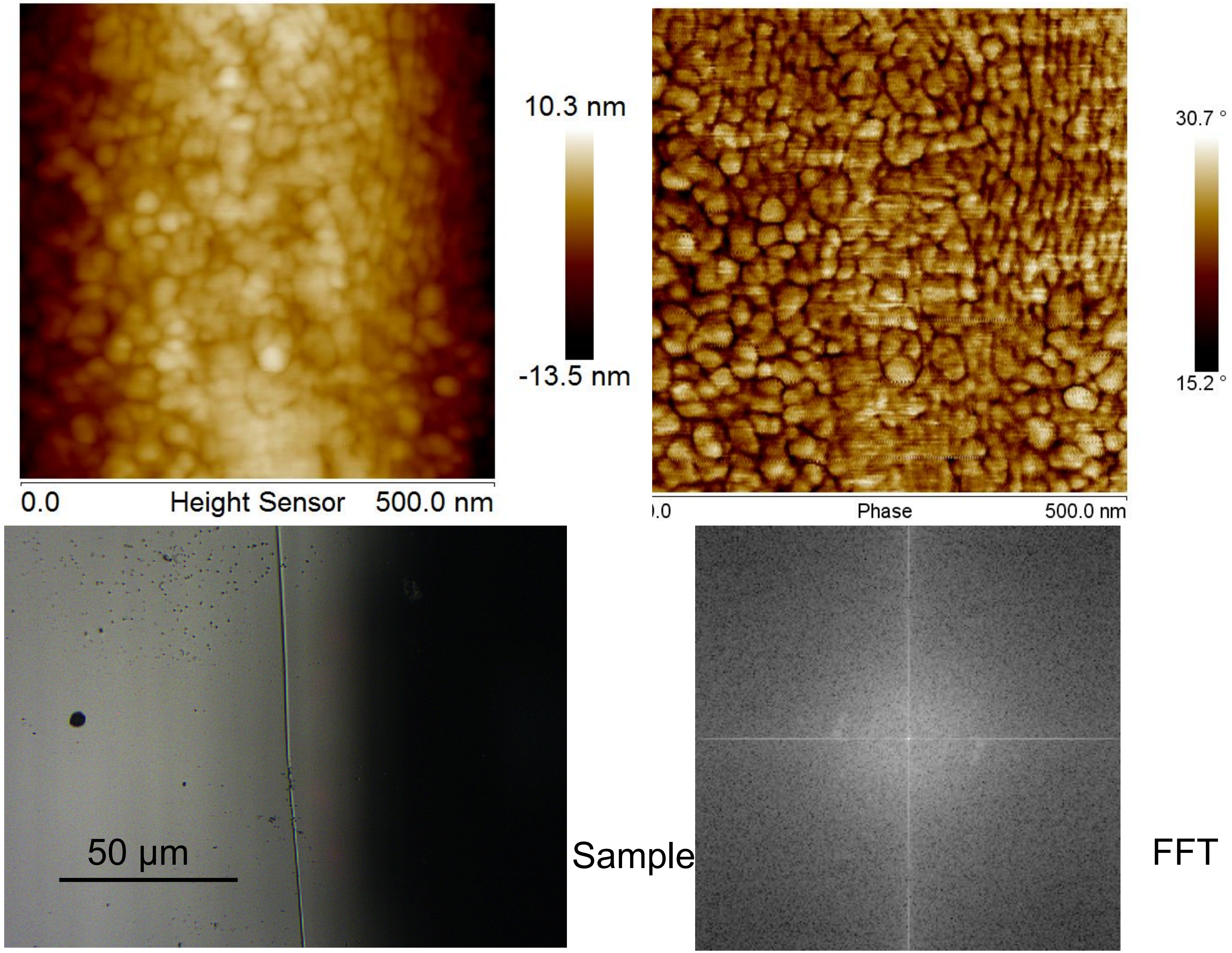

Figure S55 – Individual 5, Unstretched, Fiber 10, Image 47, 2018/02/08

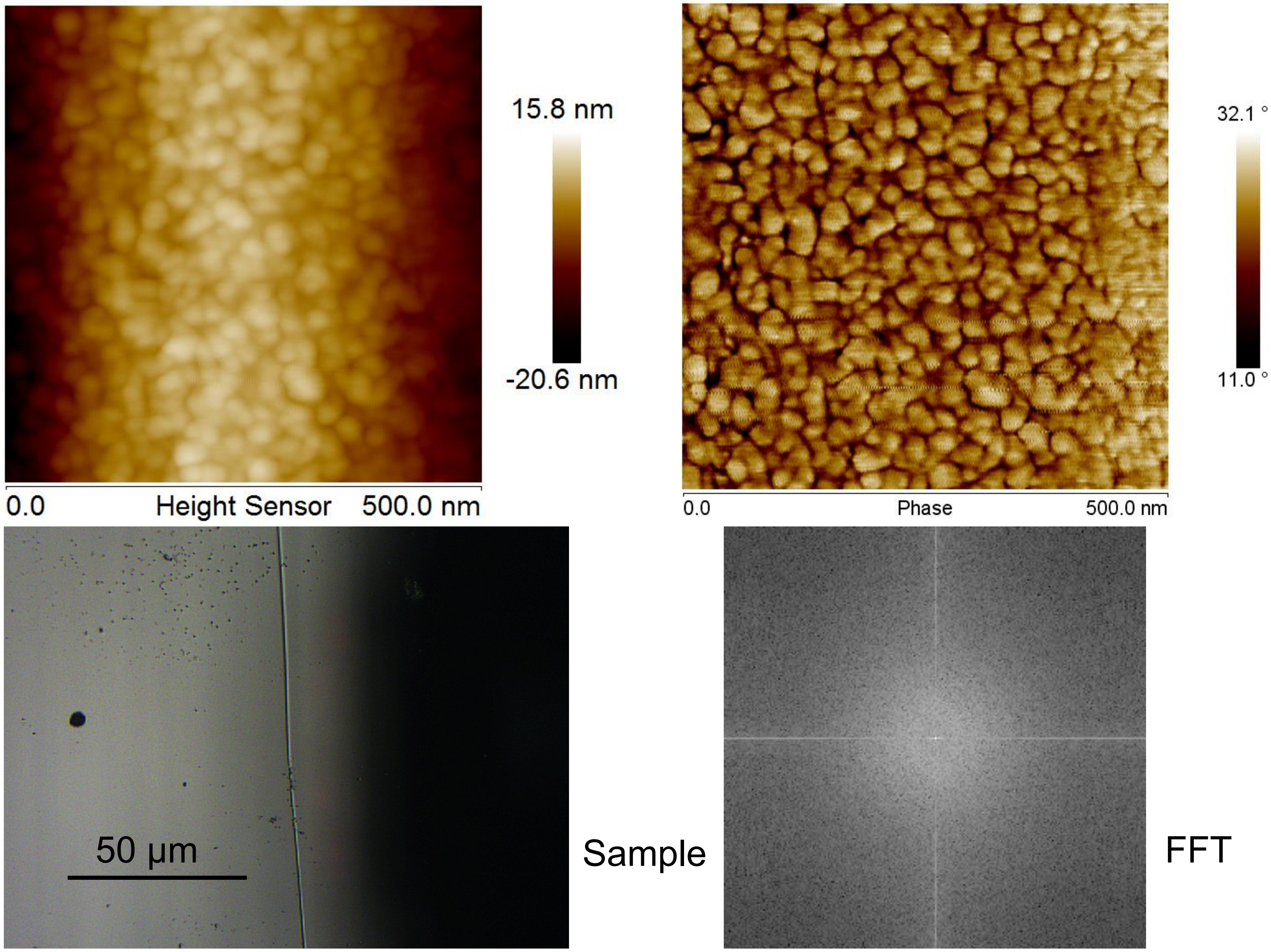

Figure S56 – Individual 6, Stretched, Fiber 11, Image 48, 2018/03/02

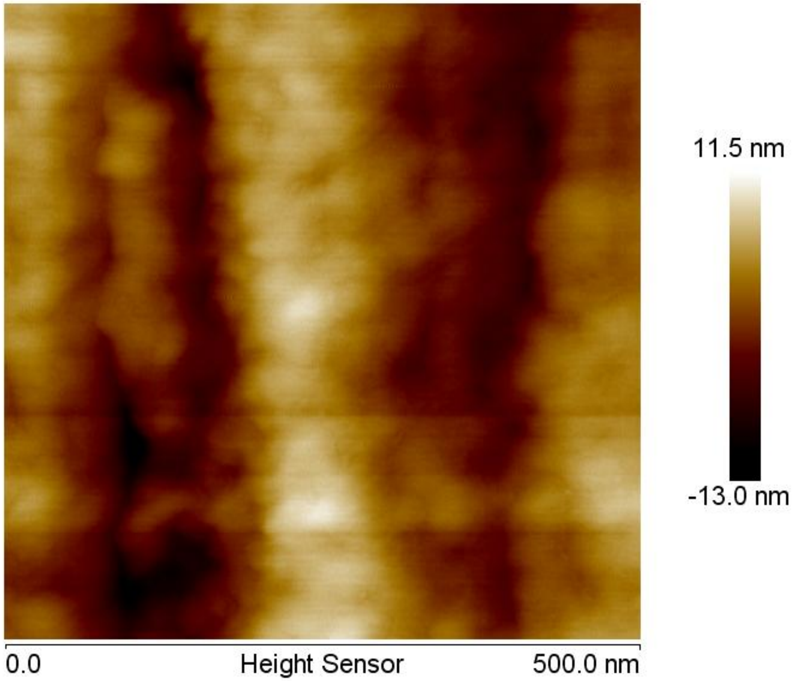

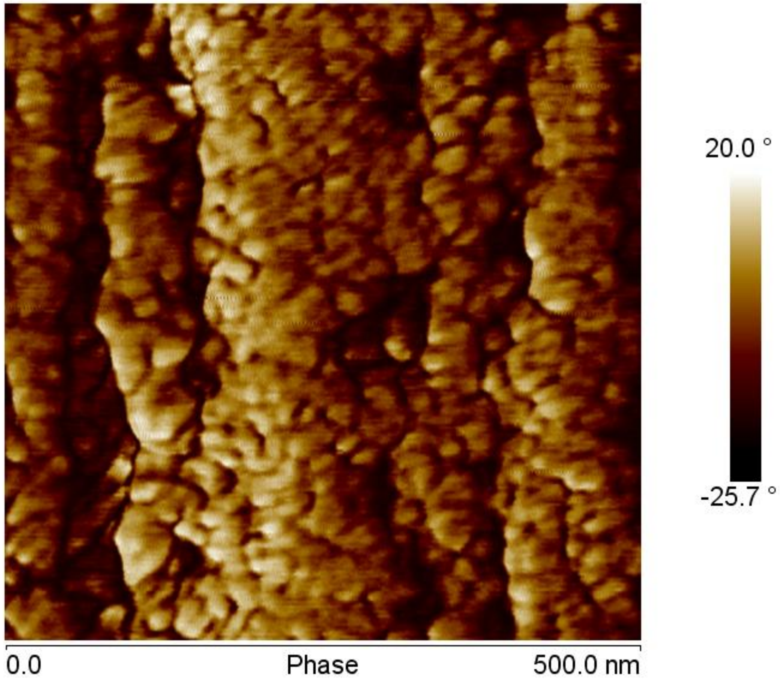

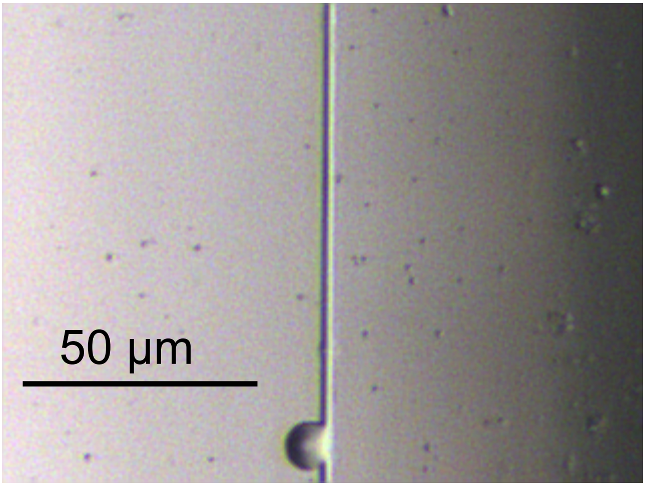

Sample

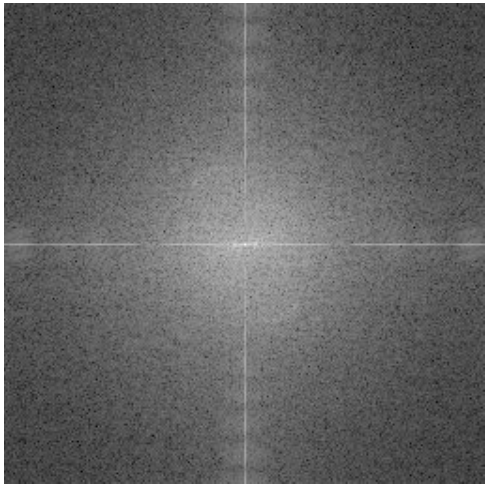

FFT

Figure S57 – Individual 6, Stretched, Fiber 11, Image 49, 2018/03/02

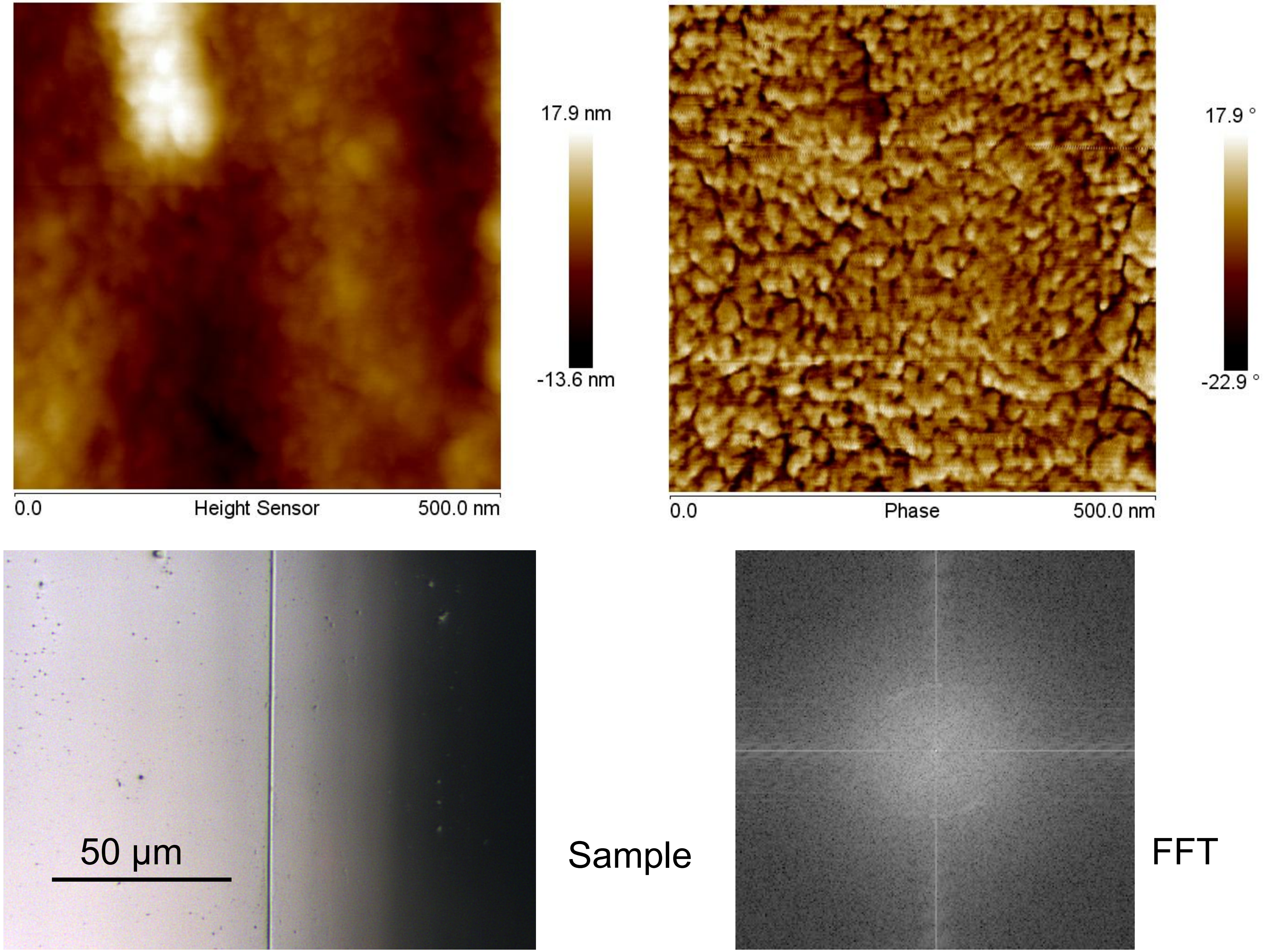

Figure S58 – Individual 6, Stretched, Fiber 11, Image 50, 2018/03/02

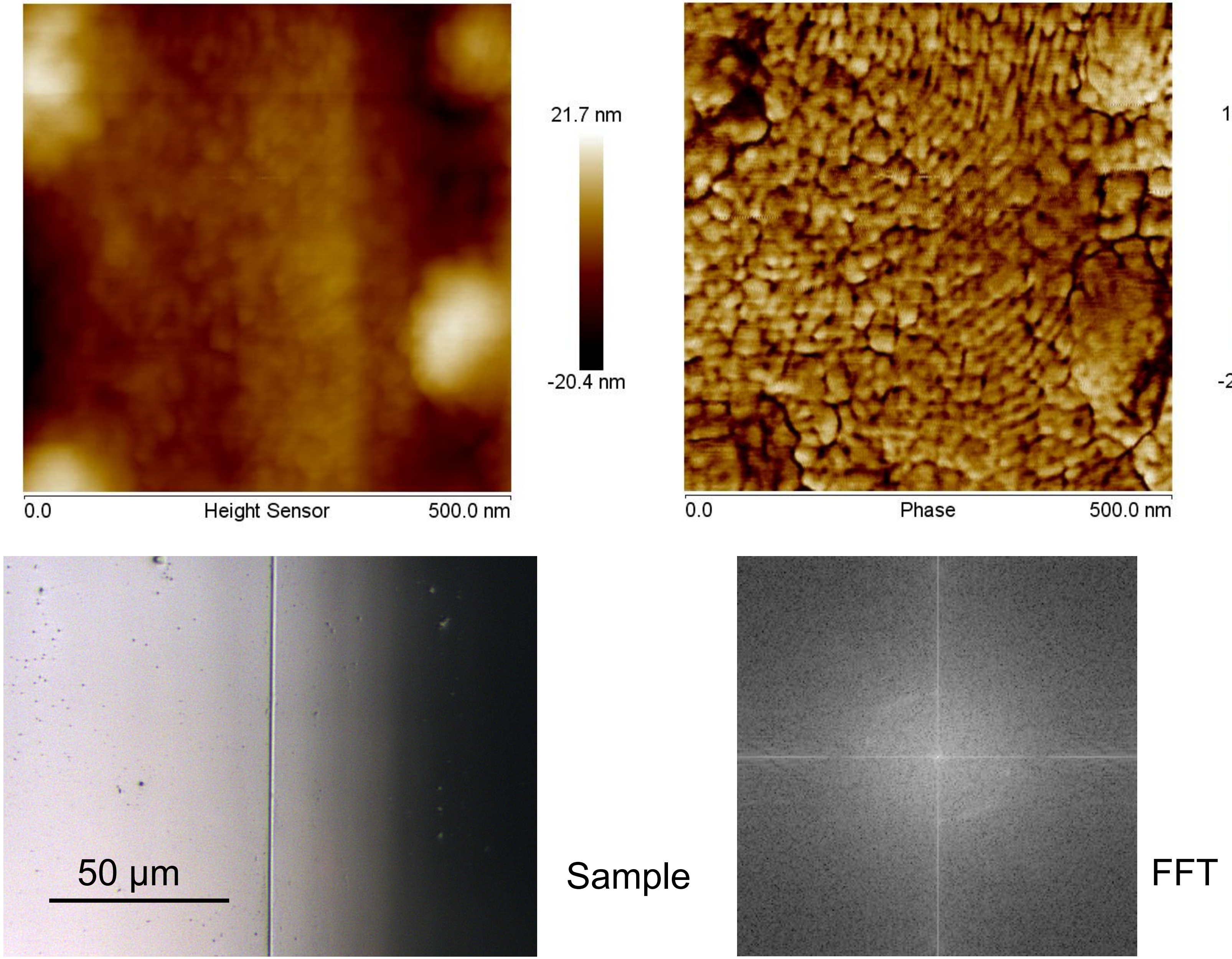

Figure S59 – Individual 6, Stretched, Fiber 11, Image 51, 2018/03/02

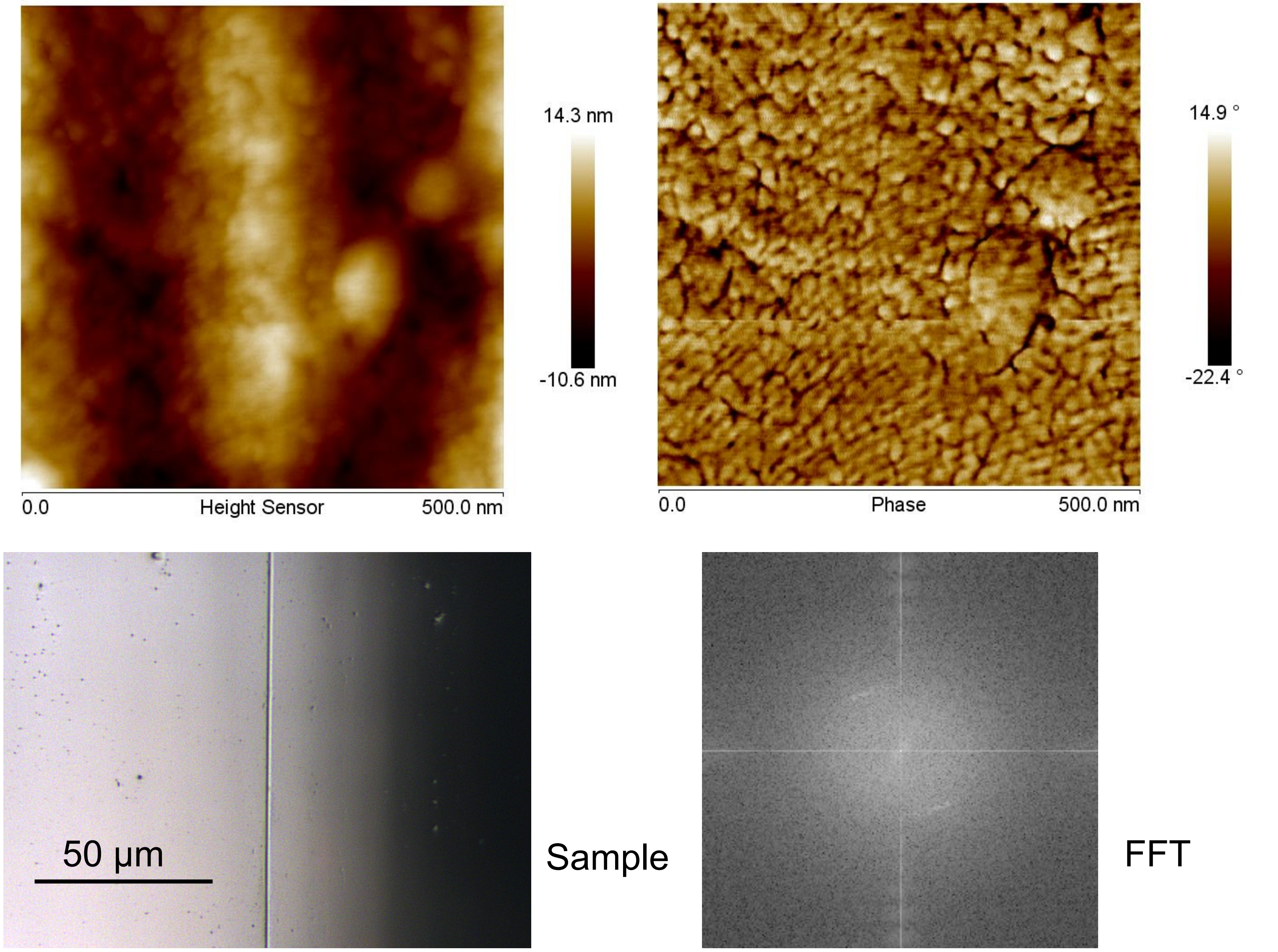

Figure S60 – Individual 6, Stretched, Fiber 11, Image 52, 2018/03/02

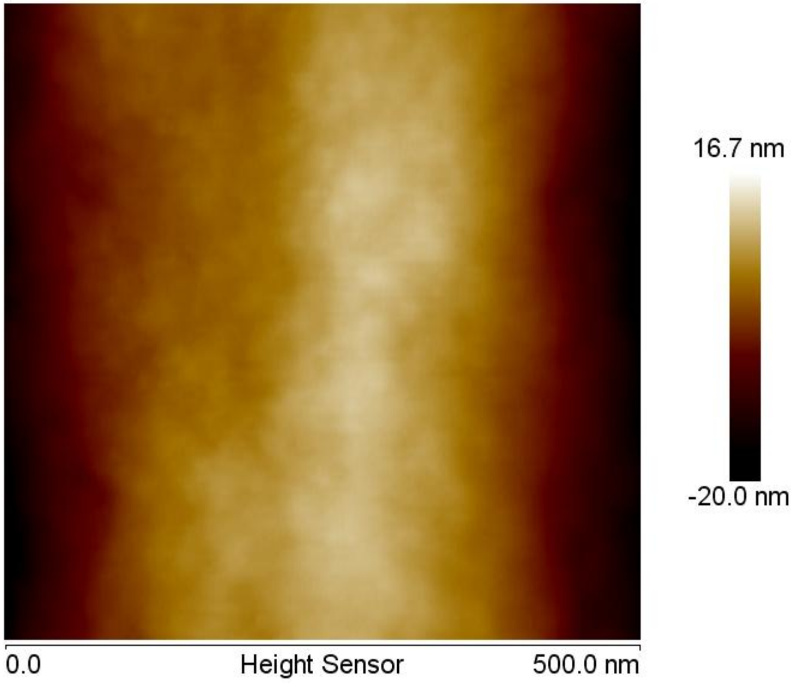

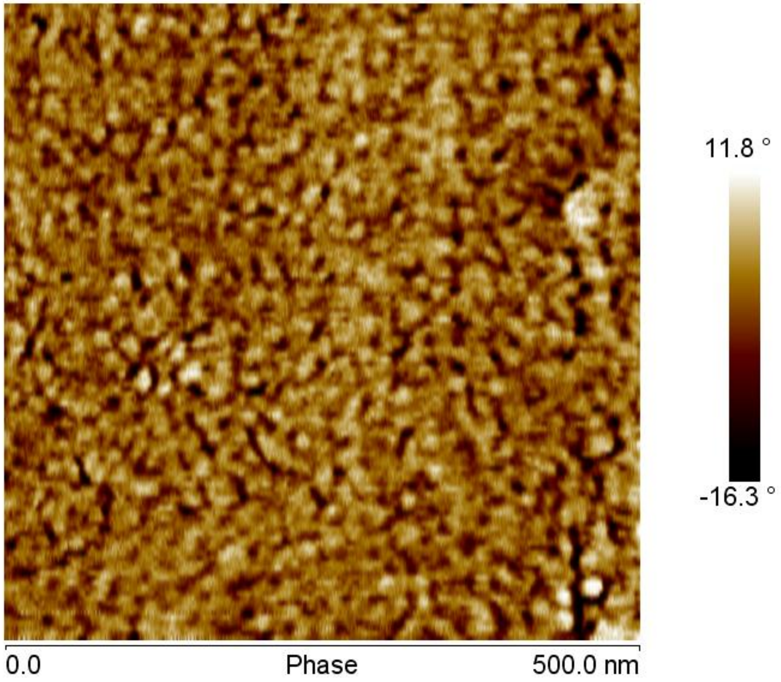

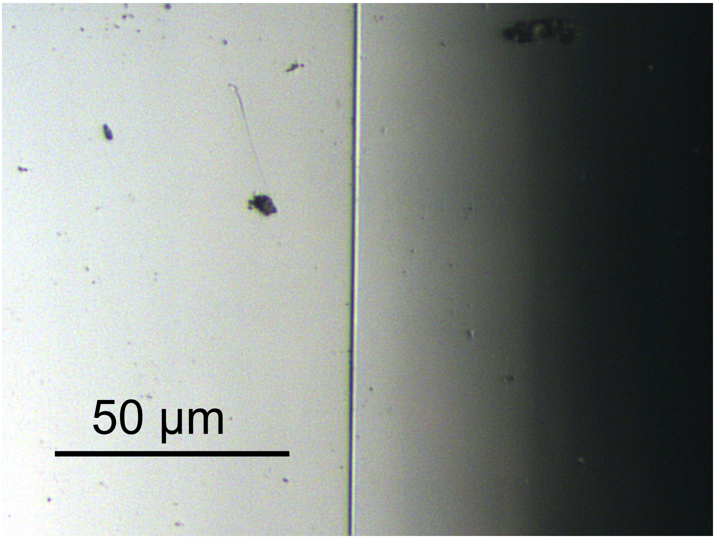

Sample

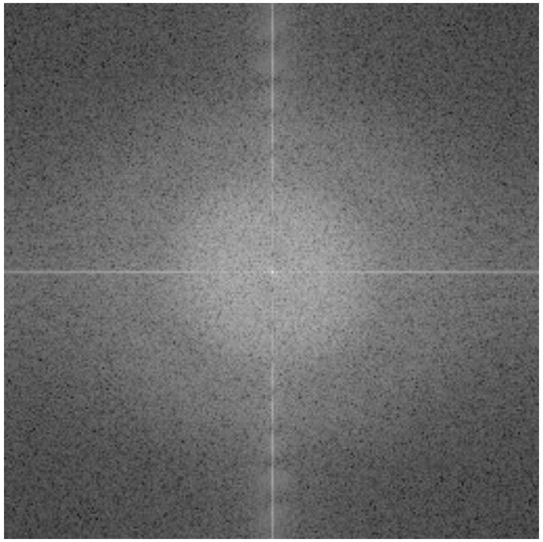

FFT

Figure S61 – Individual 7, Stretched, Fiber 12, Image 53, 2018/03/02

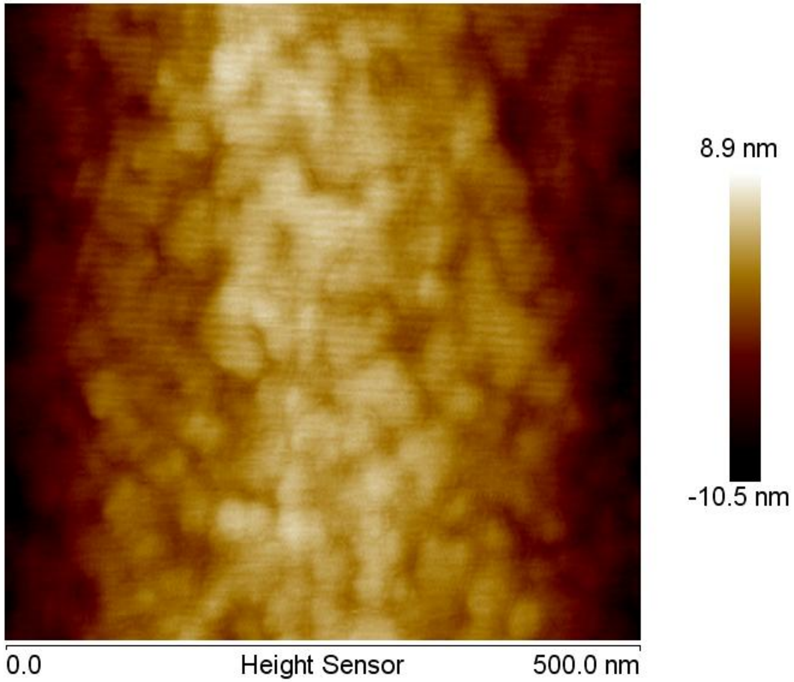

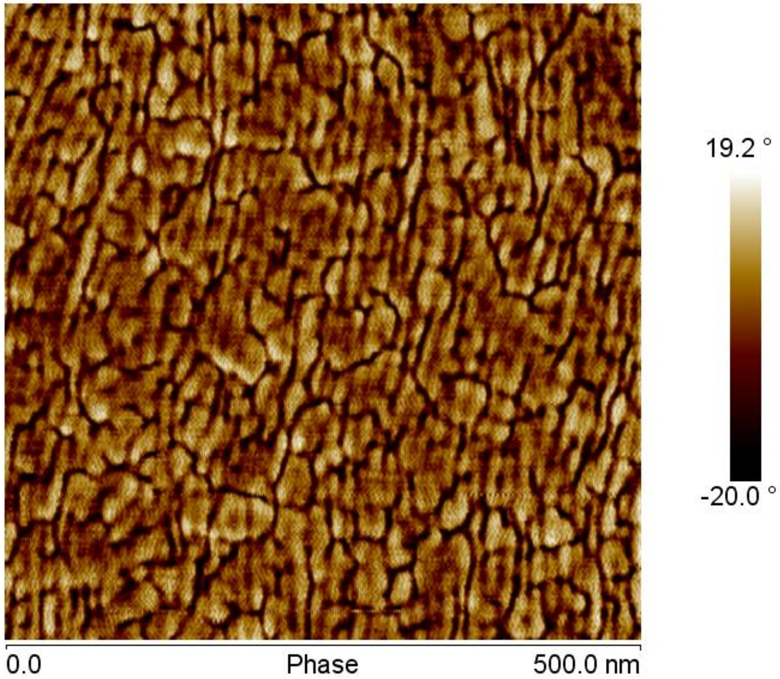

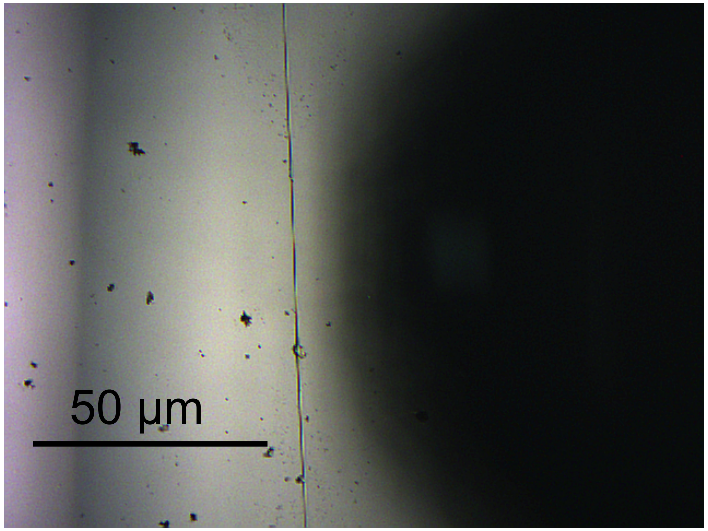

Sample

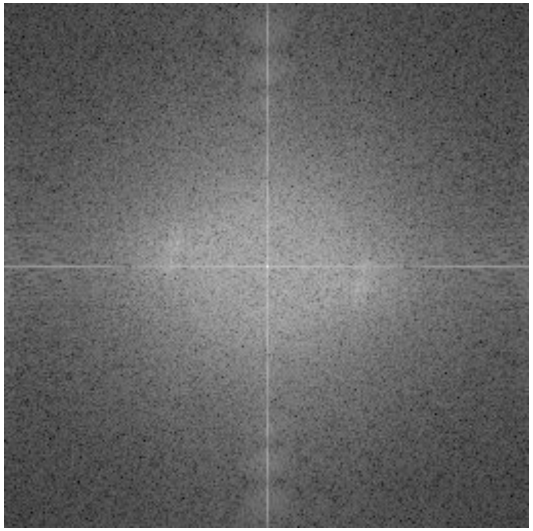

FFT

Figure S62 – Individual 7, Stretched, Fiber 12, Image 54, 2018/03/02

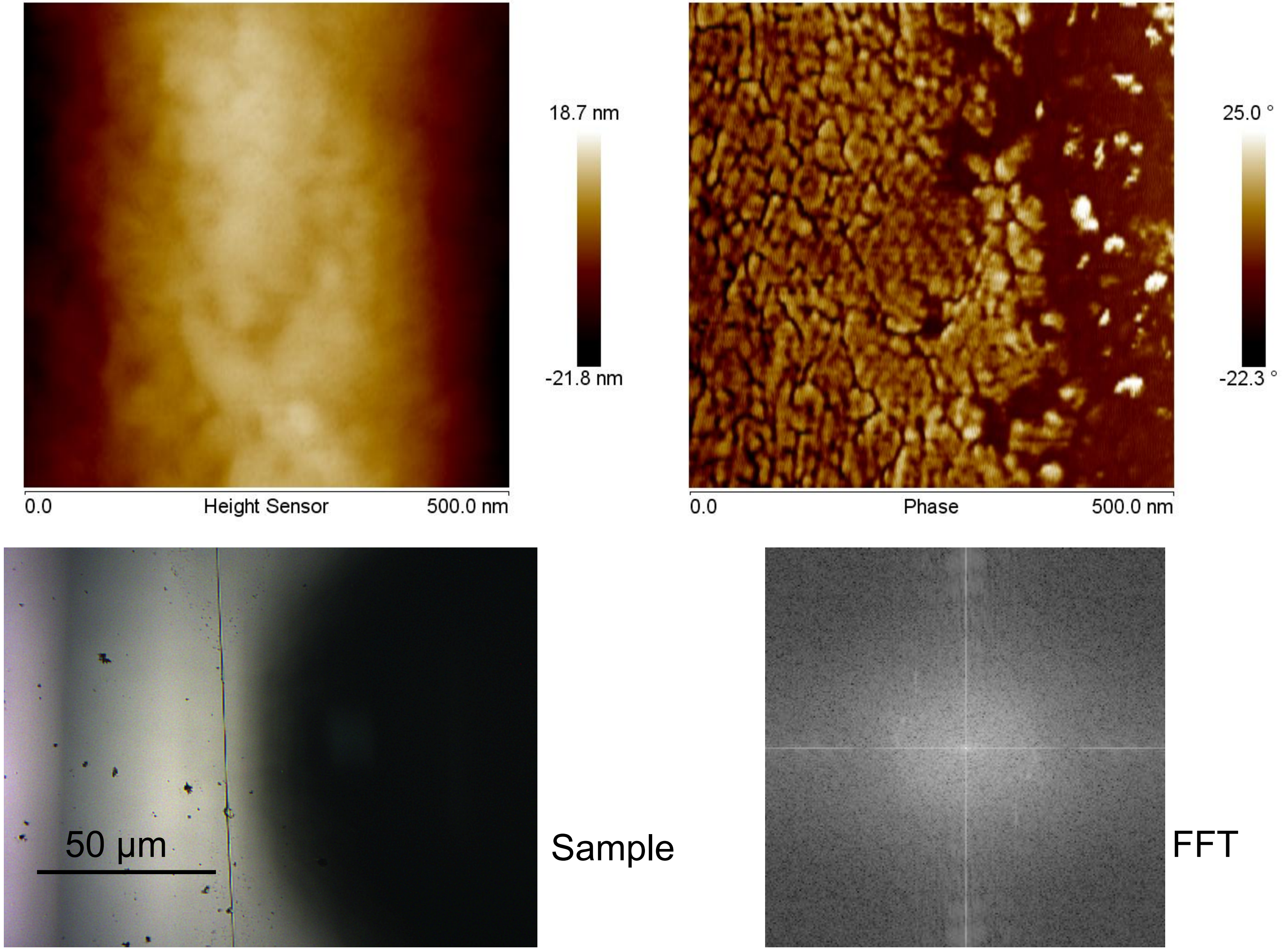

Figure S63 – Individual 7, Stretched, Fiber 12, Image 55, 2018/03/02

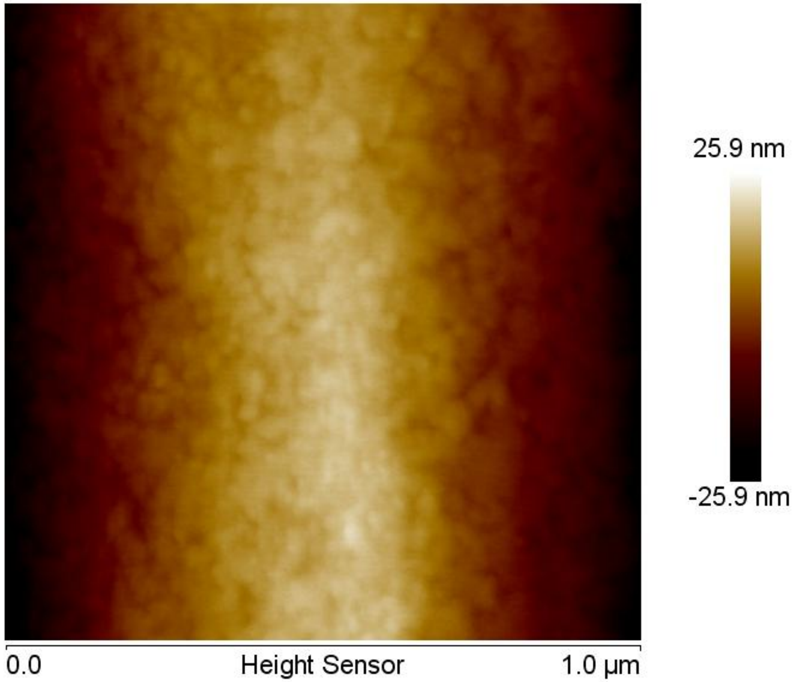

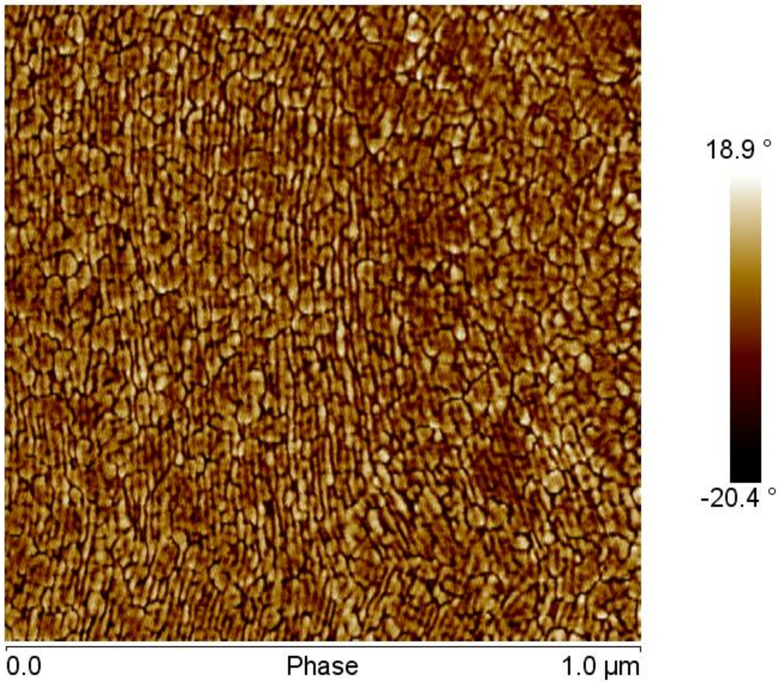

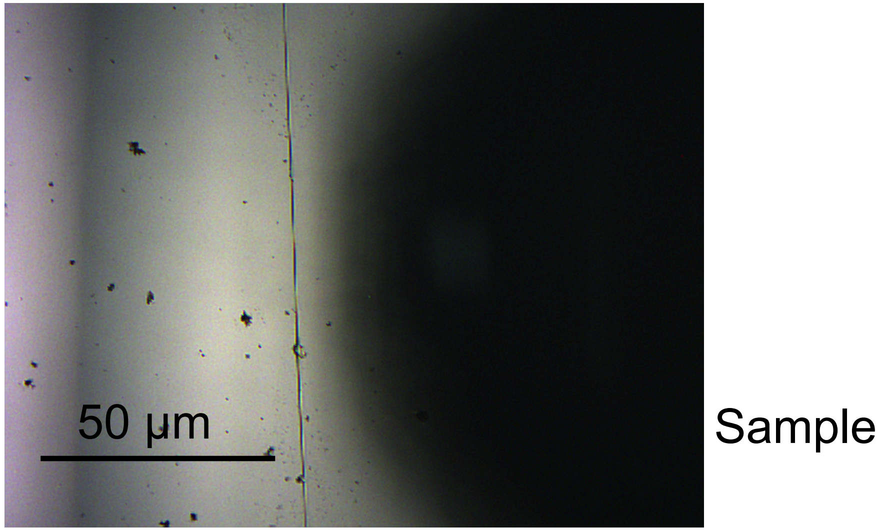

Sample

Figure S64 – Individual 7, Stretched, Fiber 12, Image 56, 2018/03/02

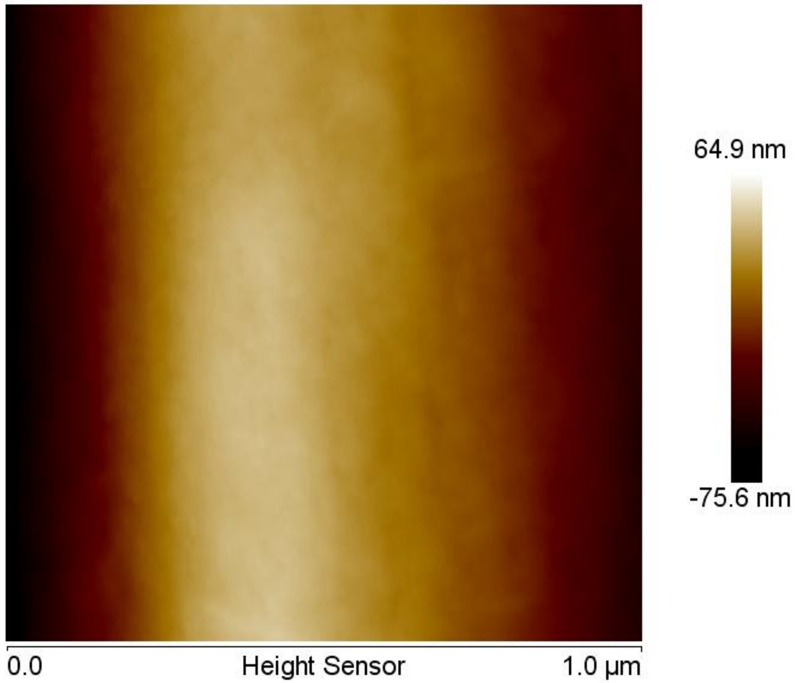

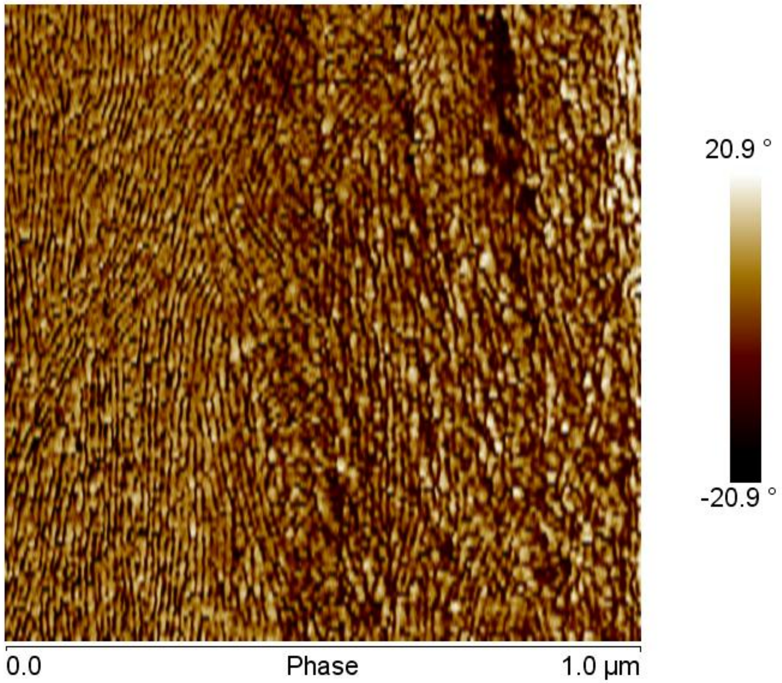

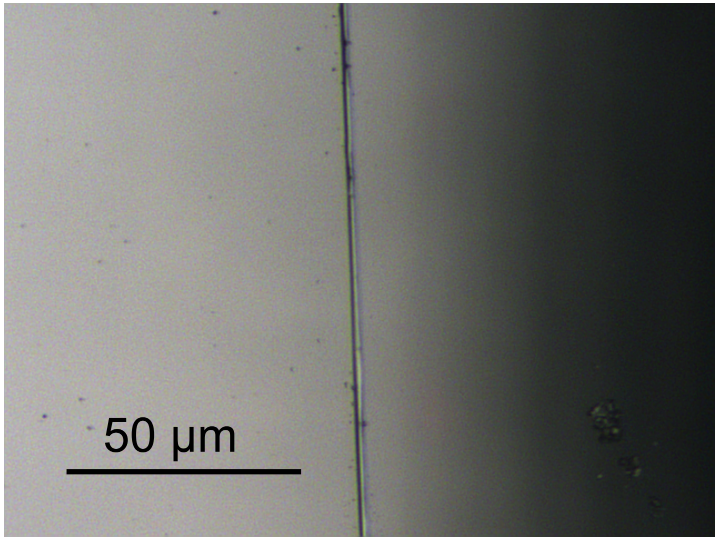

Sample

Figure S65 – Individual 7, Stretched, Fiber 12, Image 57, 2018/03/02

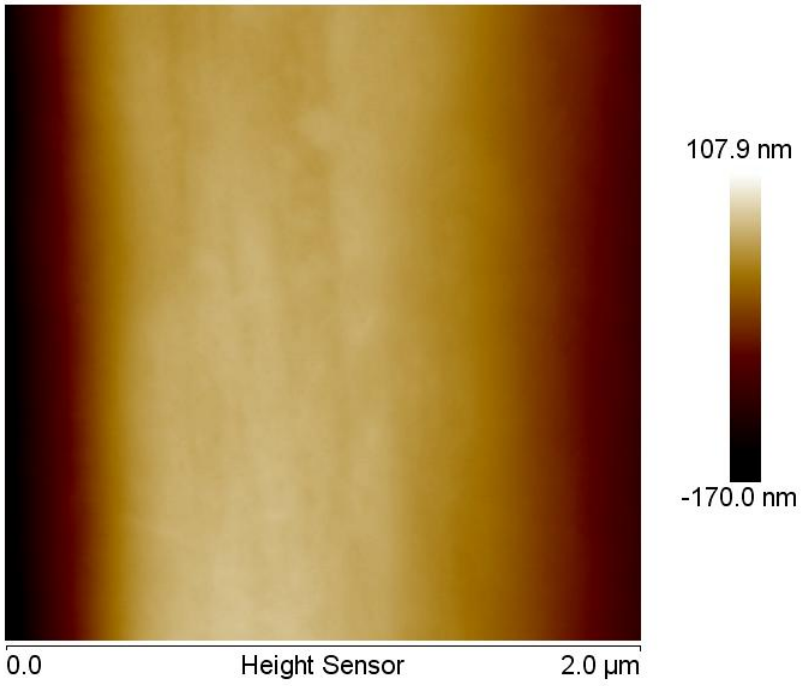

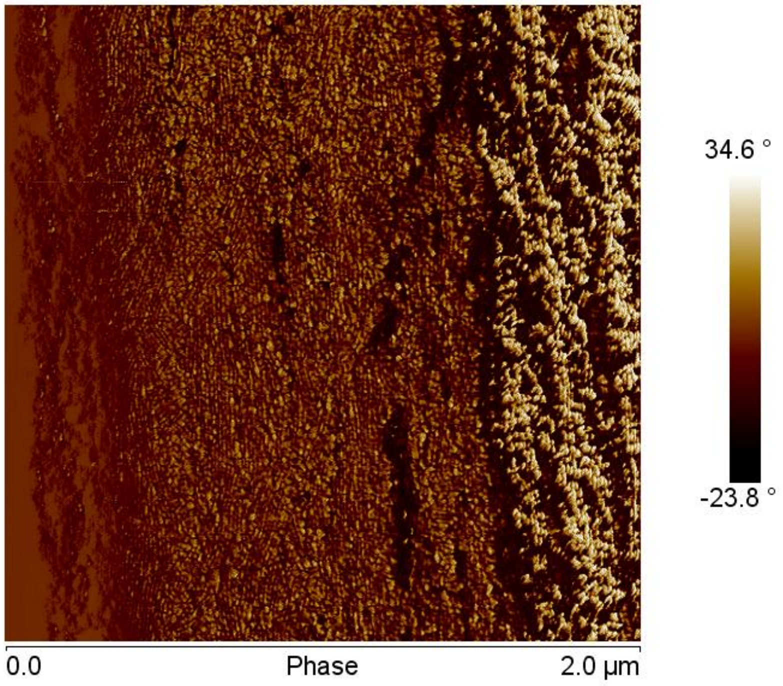

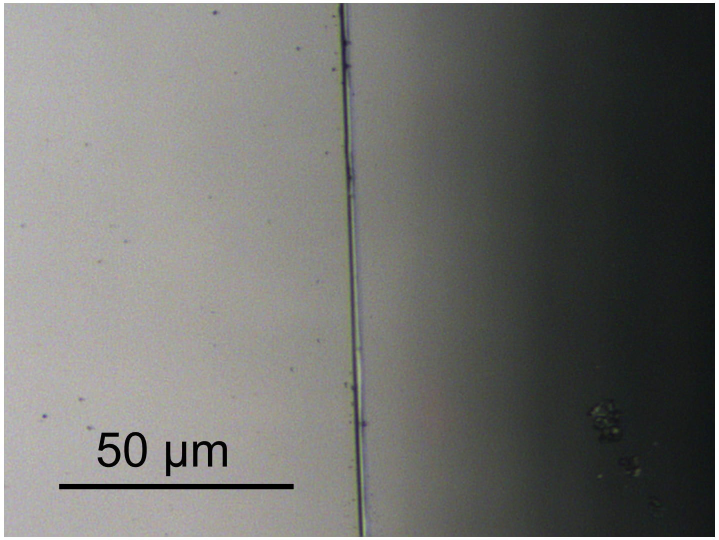

Sample

Figure S66 – Individual 7, Stretched, Fiber 12, Image 58, 2018/03/02

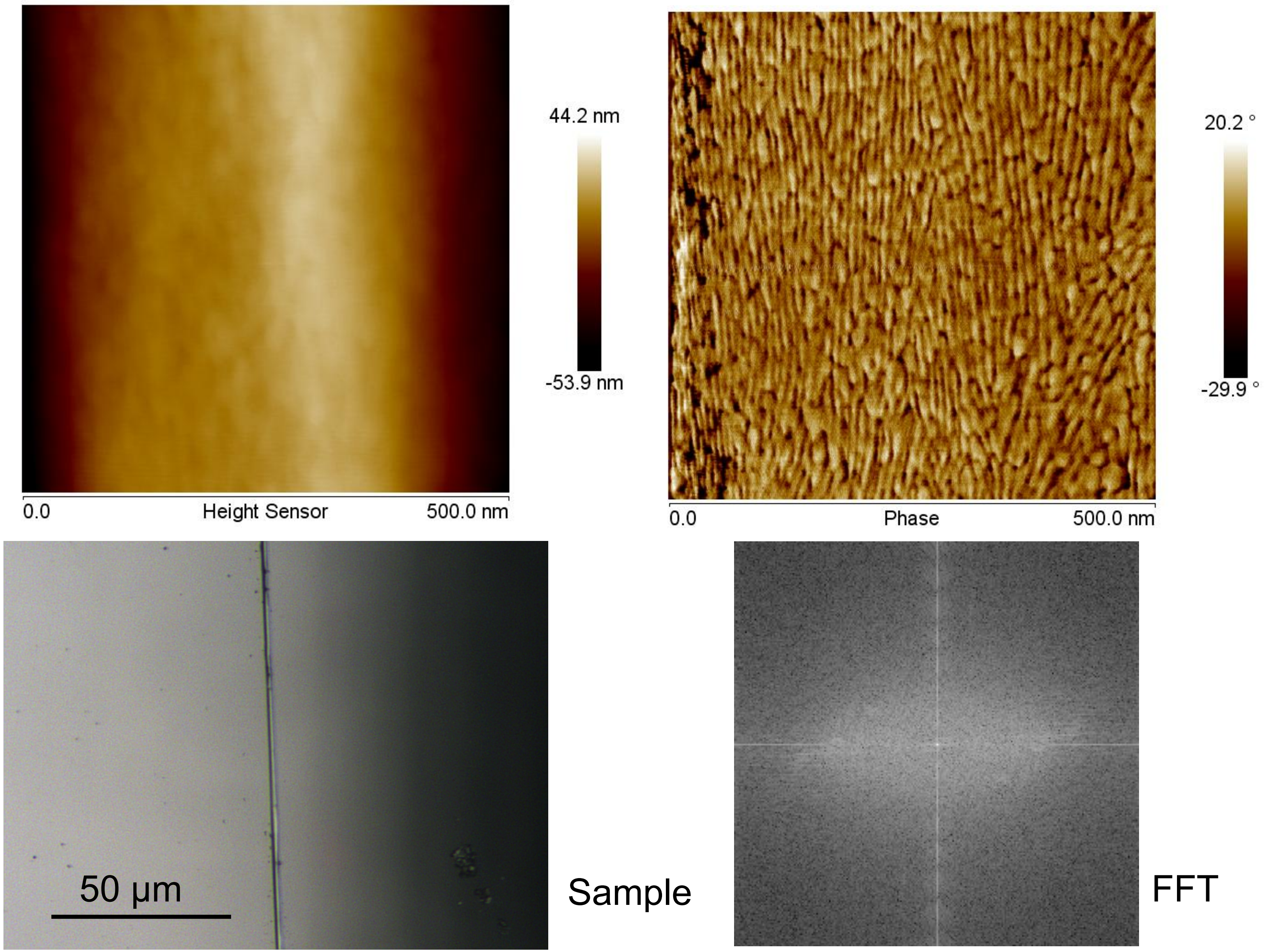

Figure S67 – Individual 7, Stretched, Fiber 12, Image 59, 2018/03/02

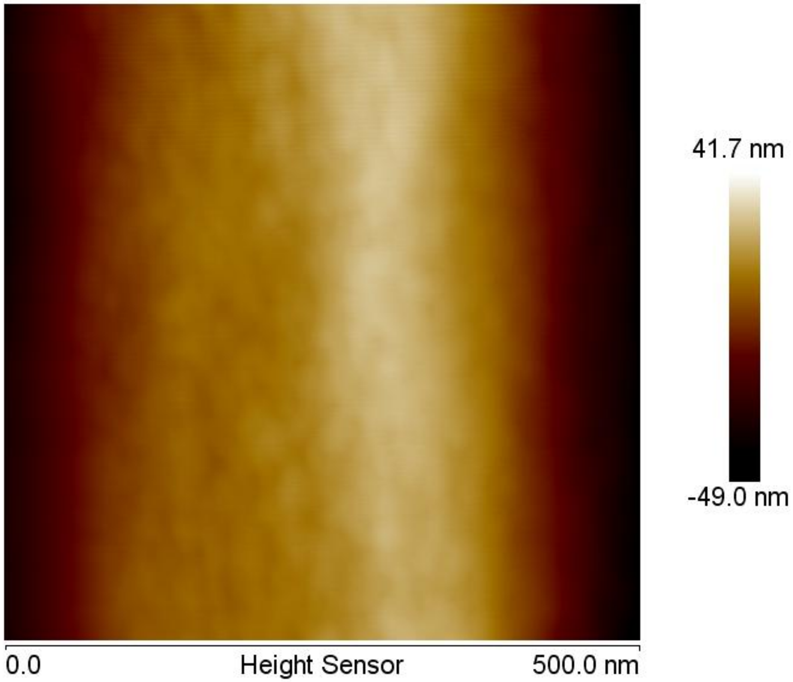

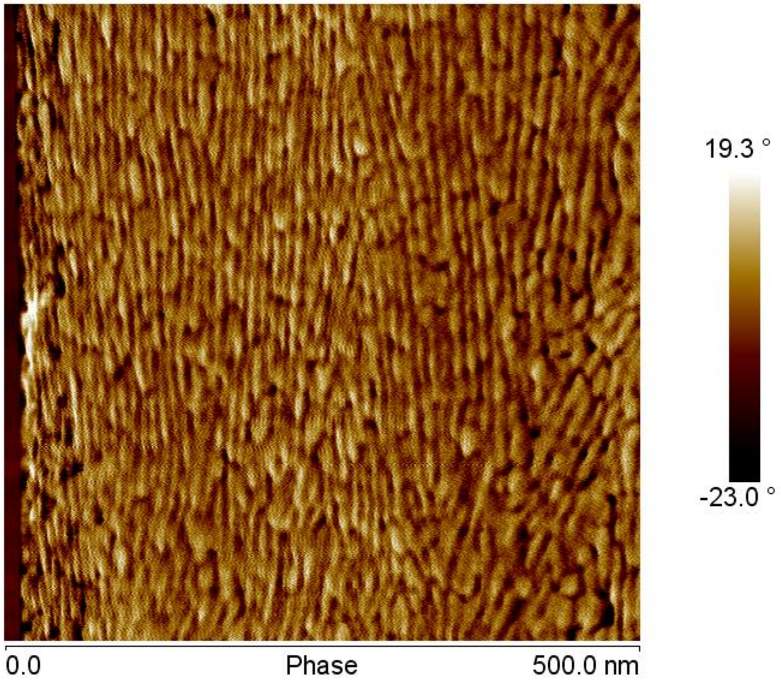

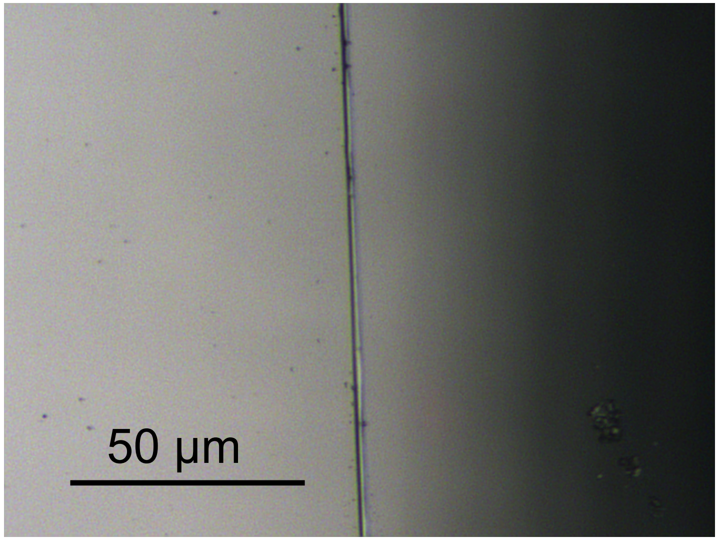

Sample

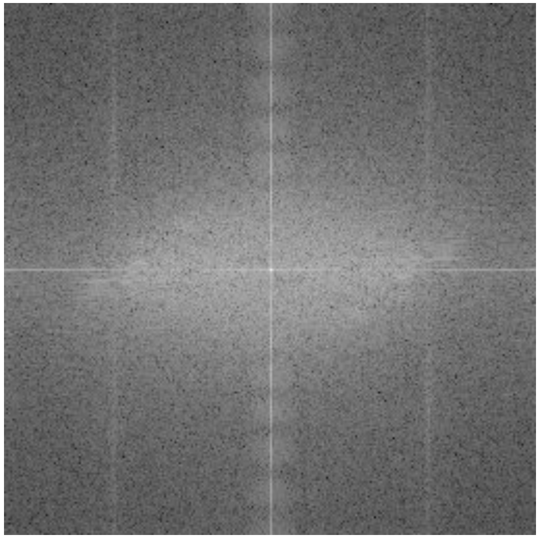

FFT

Figure S68 – Individual 8, Stretched, Fiber 13, Image 60, 2018/03/02

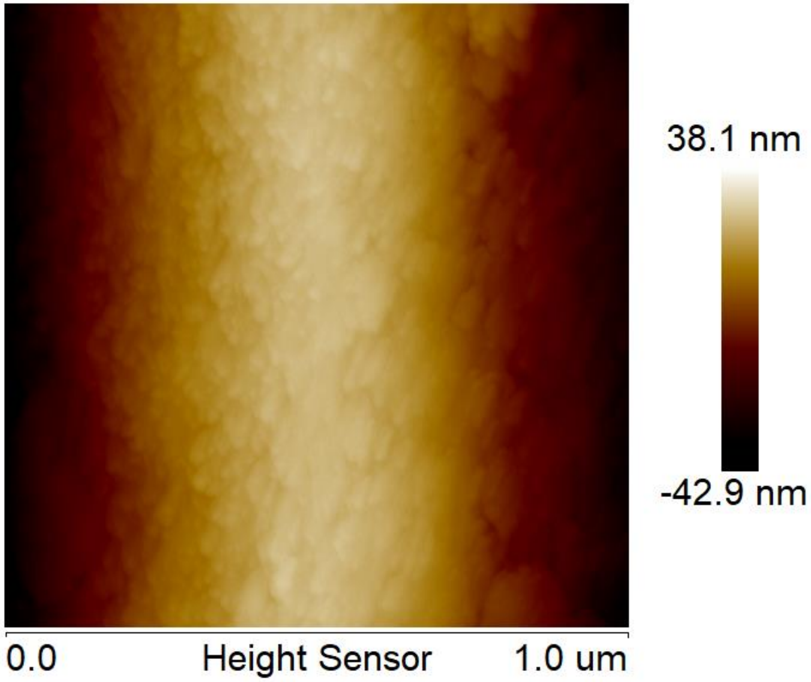

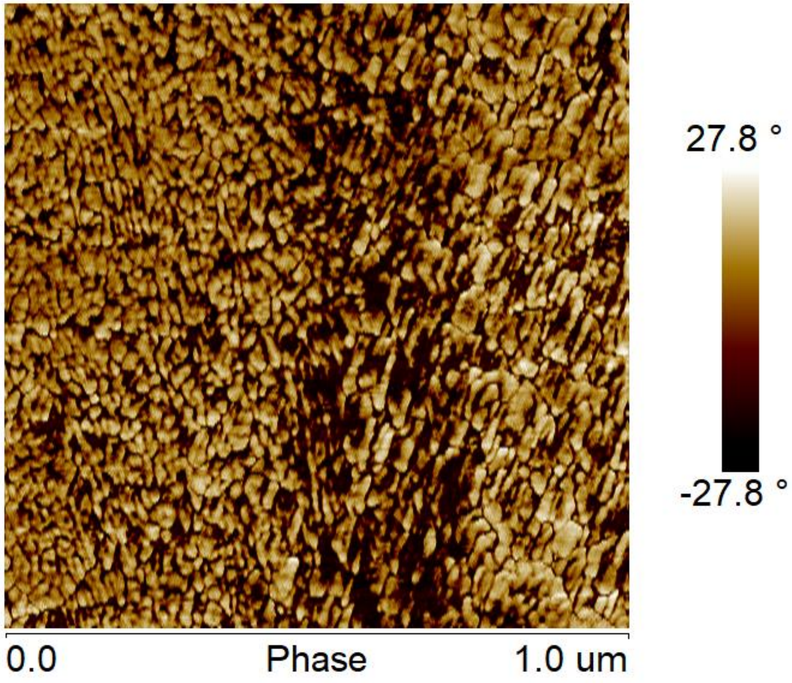

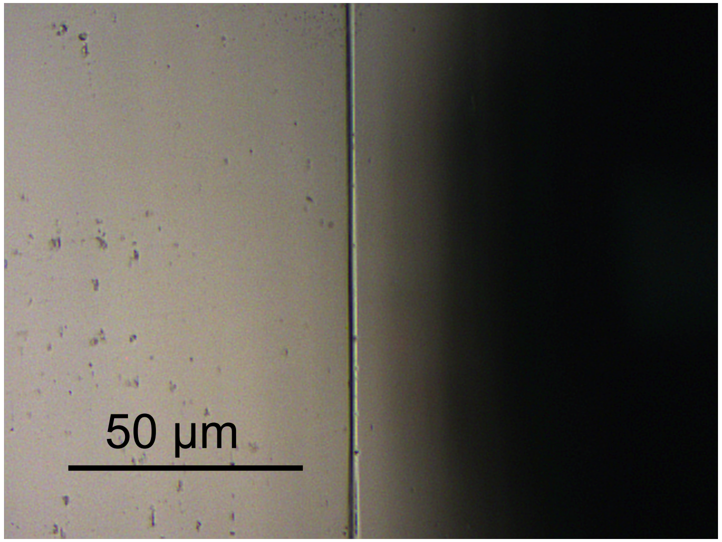

Sample

Figure S69 – Individual 8, Stretched, Fiber 13, Image 61, 2018/03/02

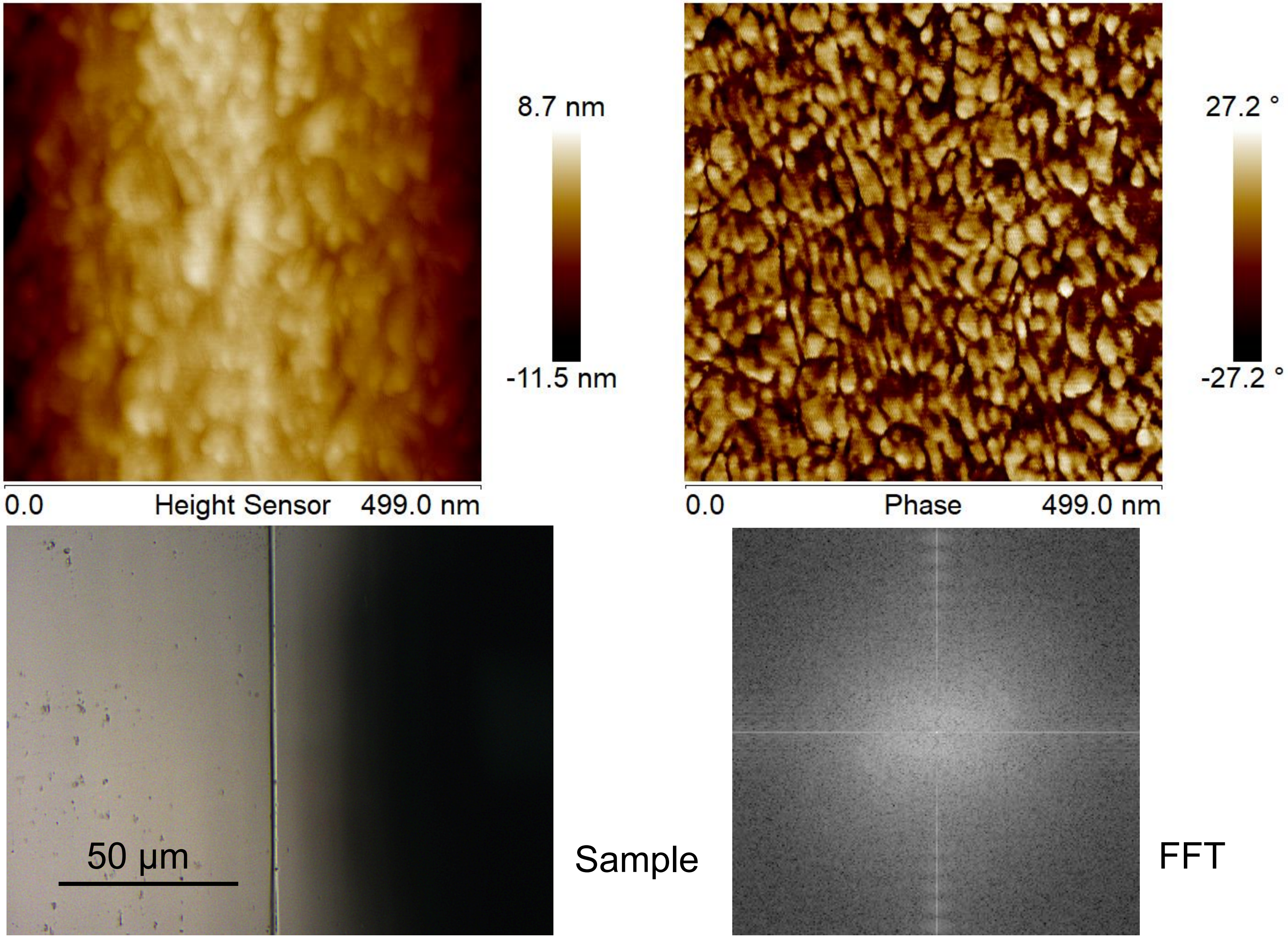

Figure S70 – Individual 8, Stretched, Fiber 13, Image 62, 2018/03/02

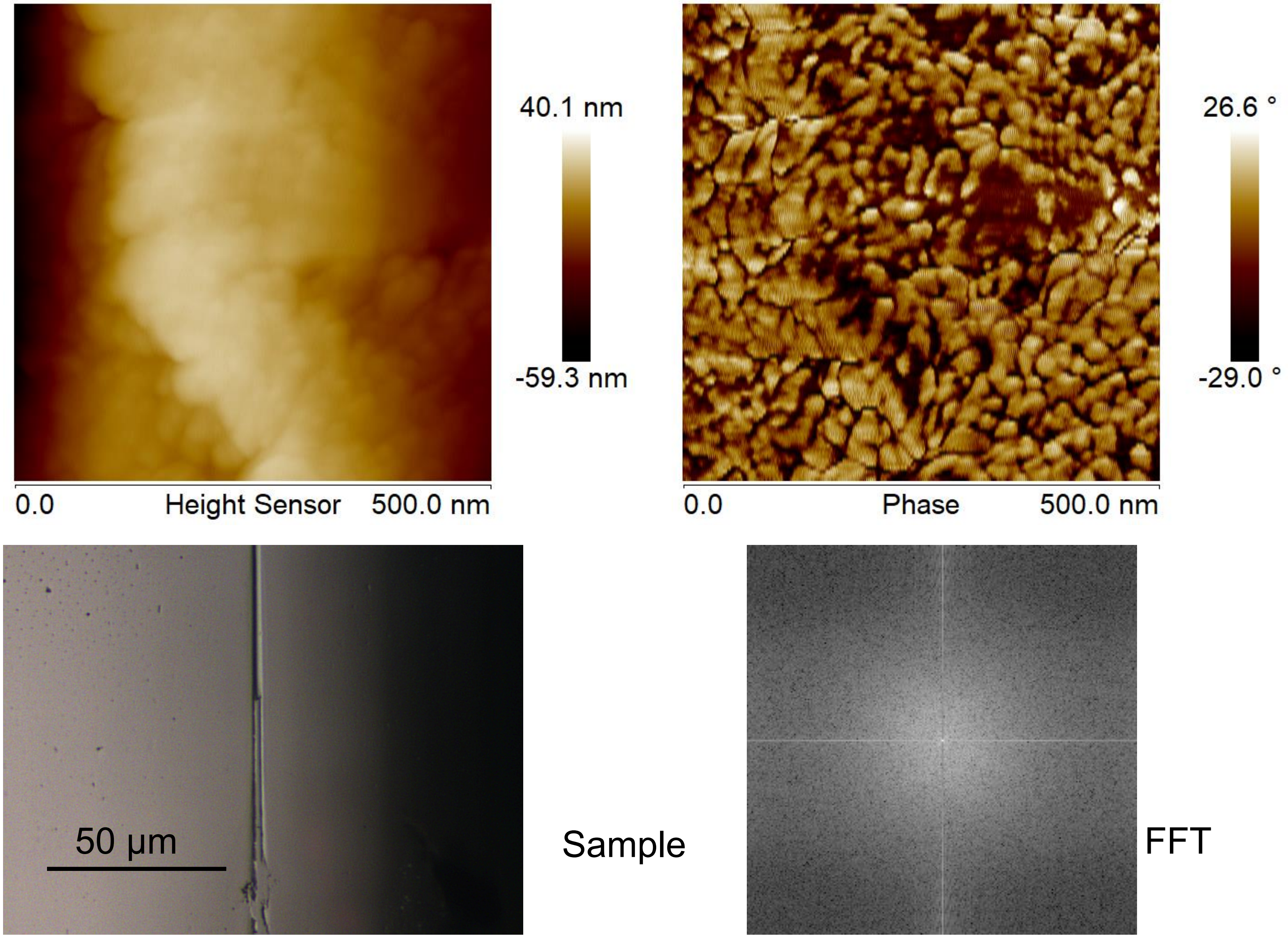

Figure S71 – Individual 8, Stretched, Fiber 13, Image 63, 2018/03/02

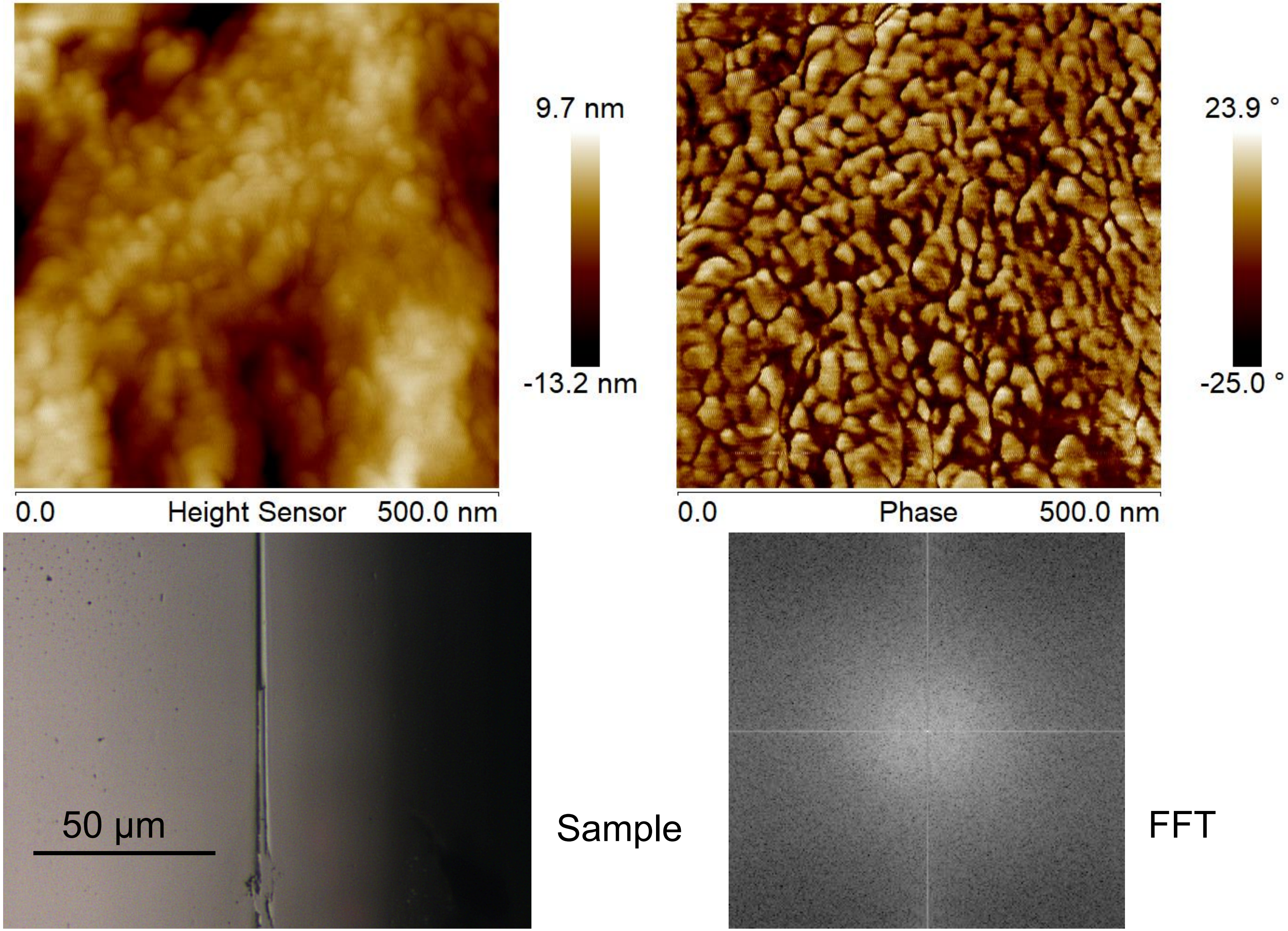

Figure S72– Individual 8, Stretched, Fiber 13, Image 64, 2018/03/02

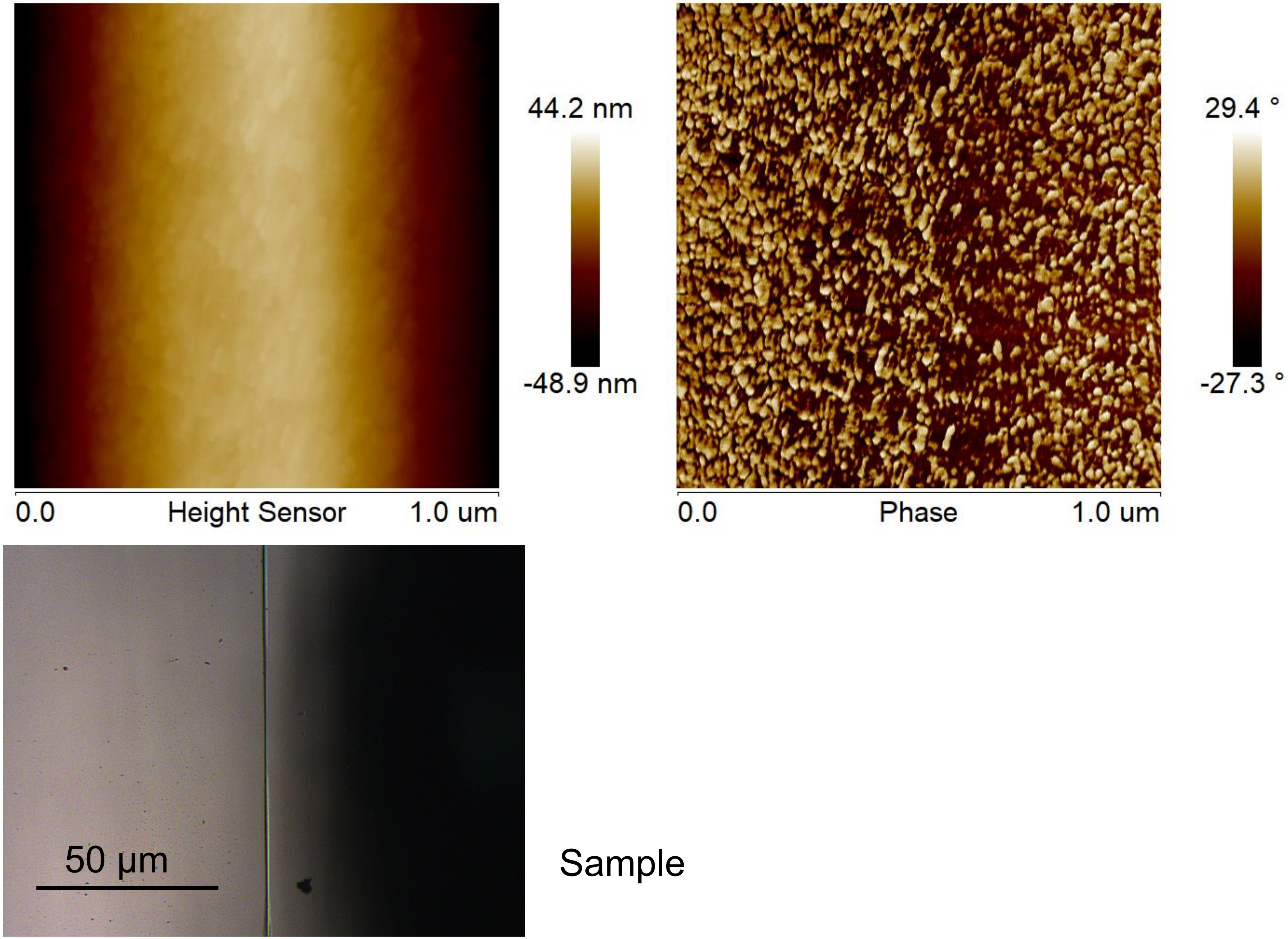

Figure S73 – Individual 8, Stretched, Fiber 13, Image 65, 2018/03/02

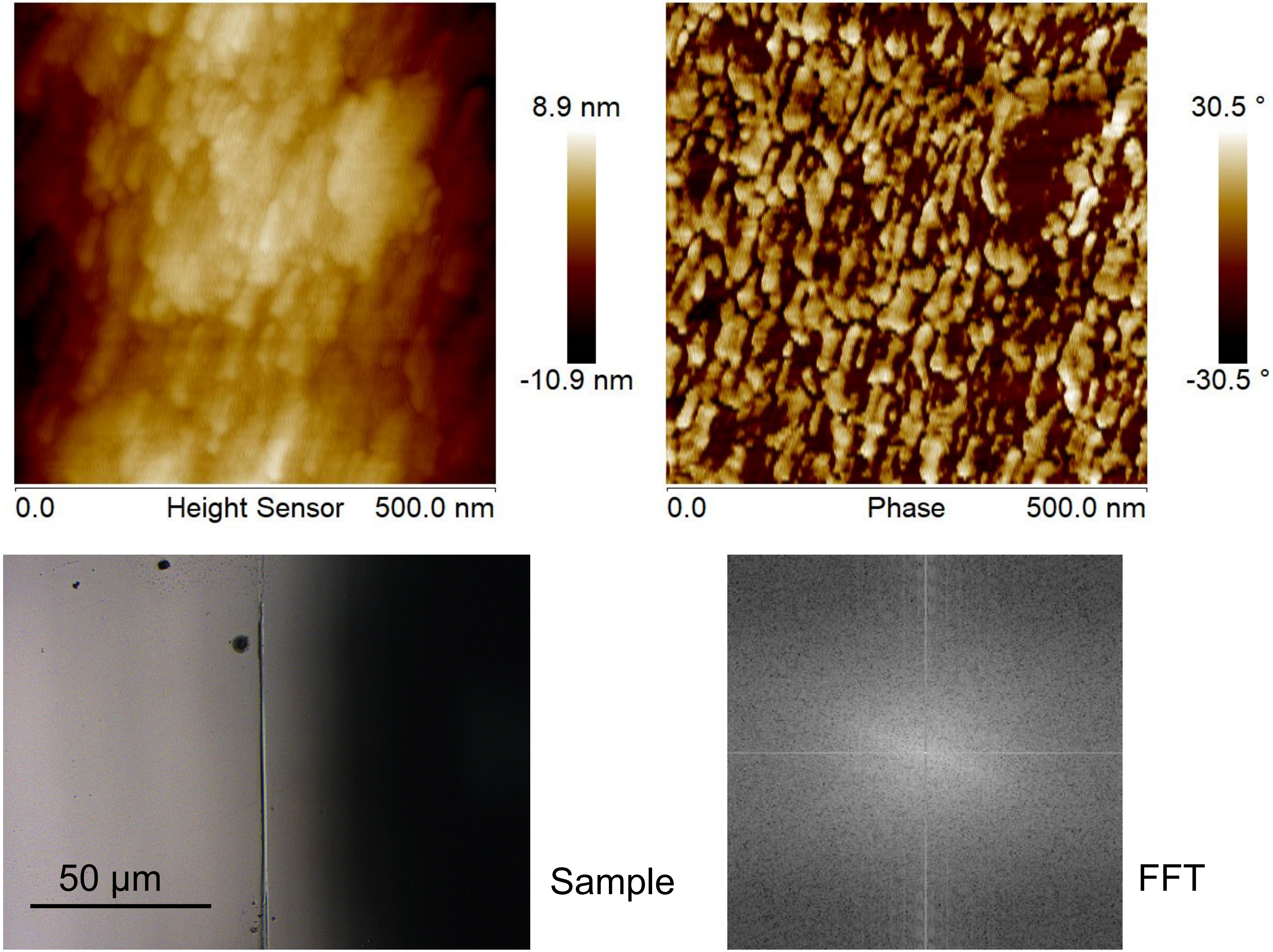

Figure S74 – Individual 8, Stretched, Fiber 13, Image 66, 2018/03/02

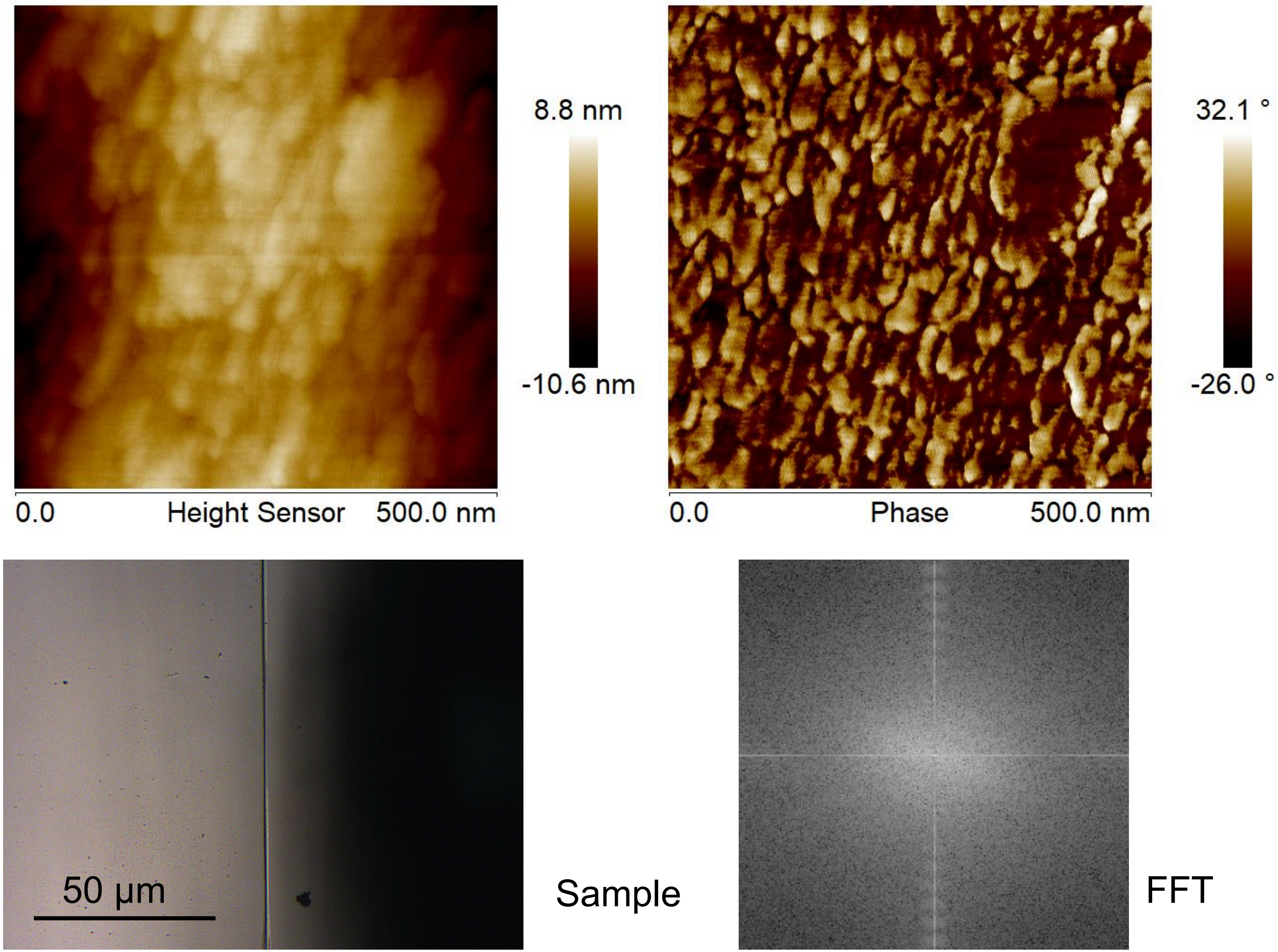

Figure S75 – Individual 6, Unstretched, Fiber 14, Image 67, 2018/03/02

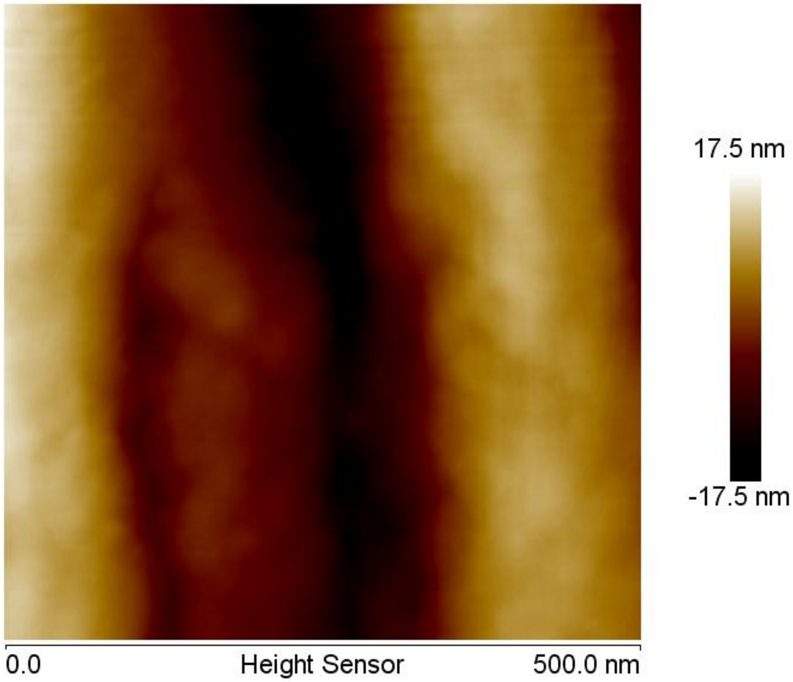

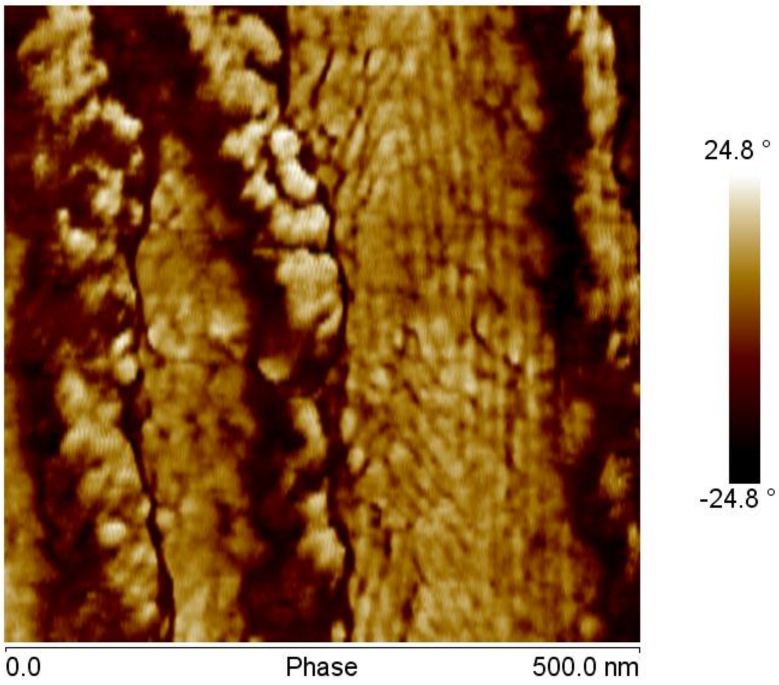

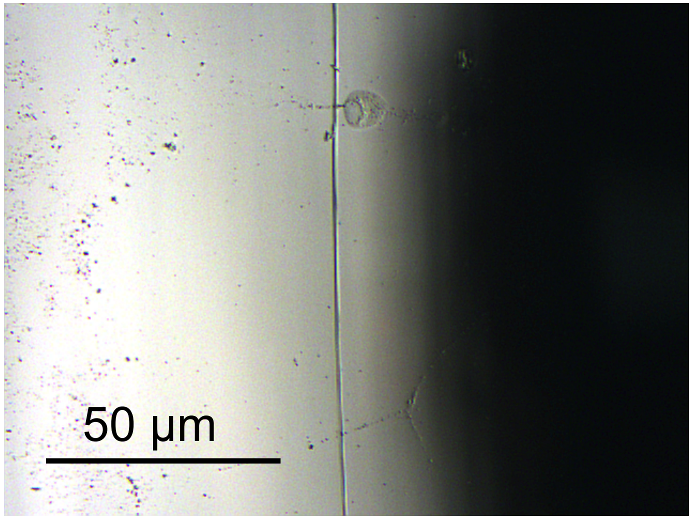

Sample

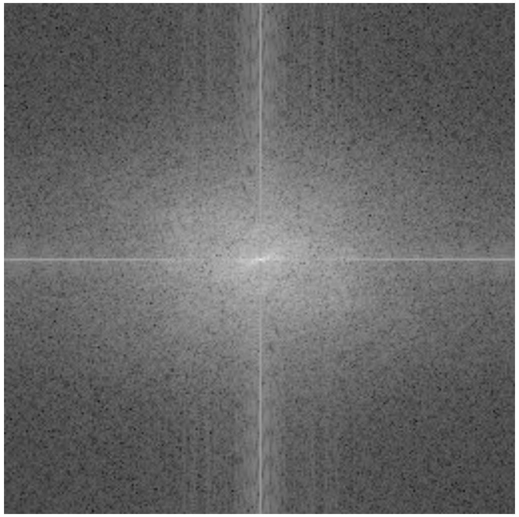

FFT

Figure S76 – Individual 6, Unstretched, Fiber 14, Image 68, 2018/03/02

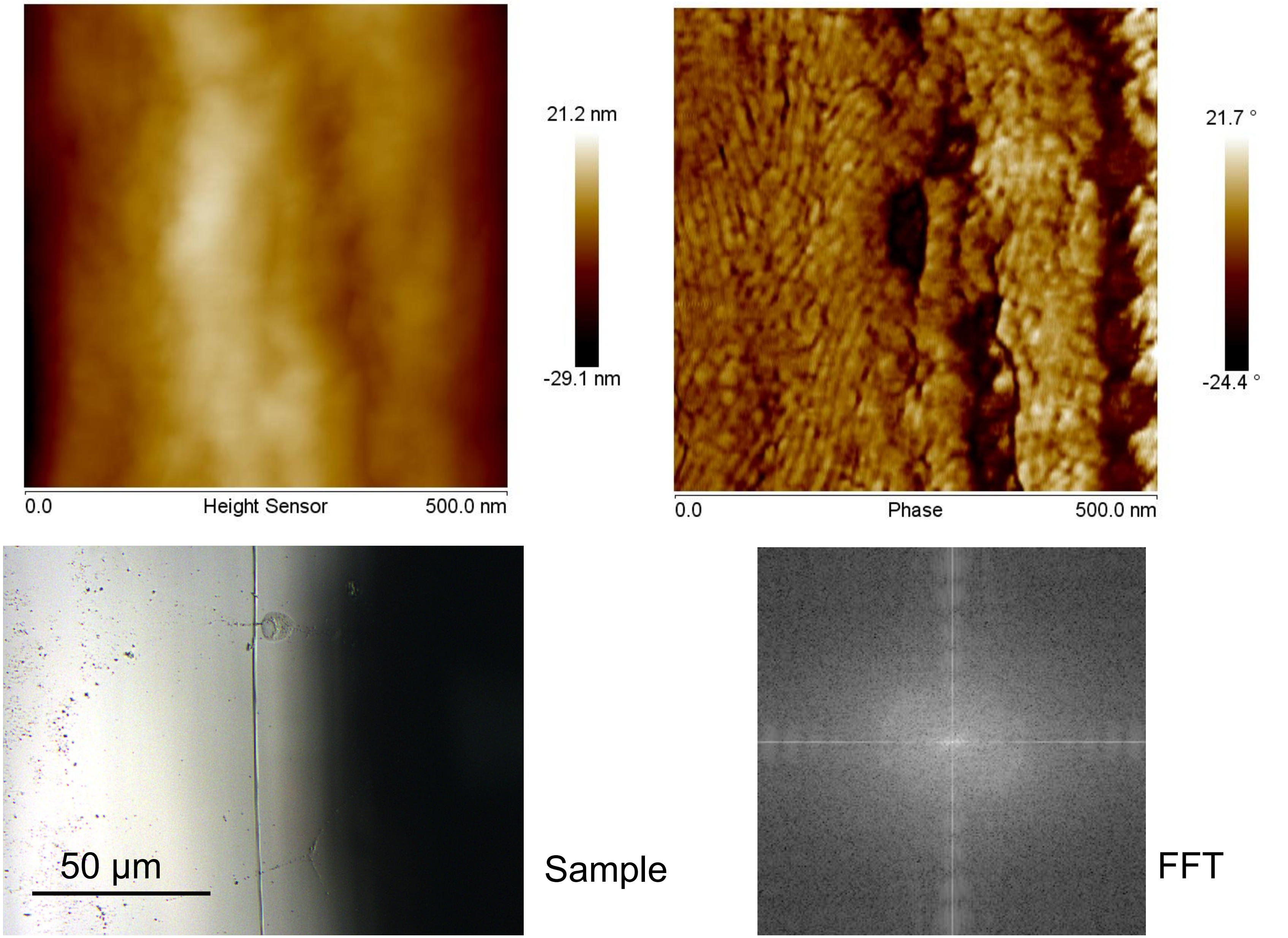

Figure S77 – Individual 6, Unstretched, Fiber 14, Image 69, 2018/03/02

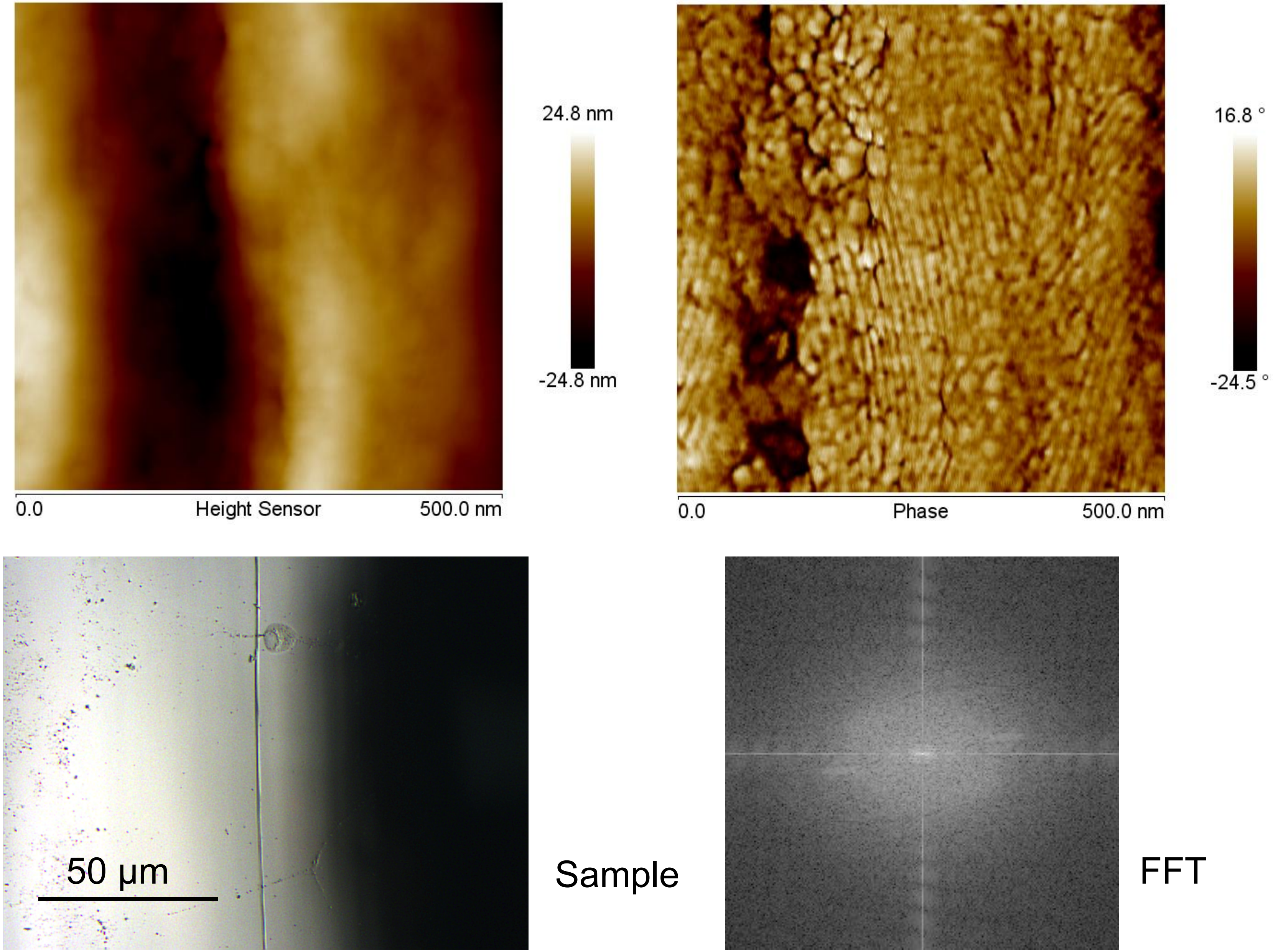

Figure S78 – Individual 6, Unstretched, Fiber 14, Image 70, 2018/03/02

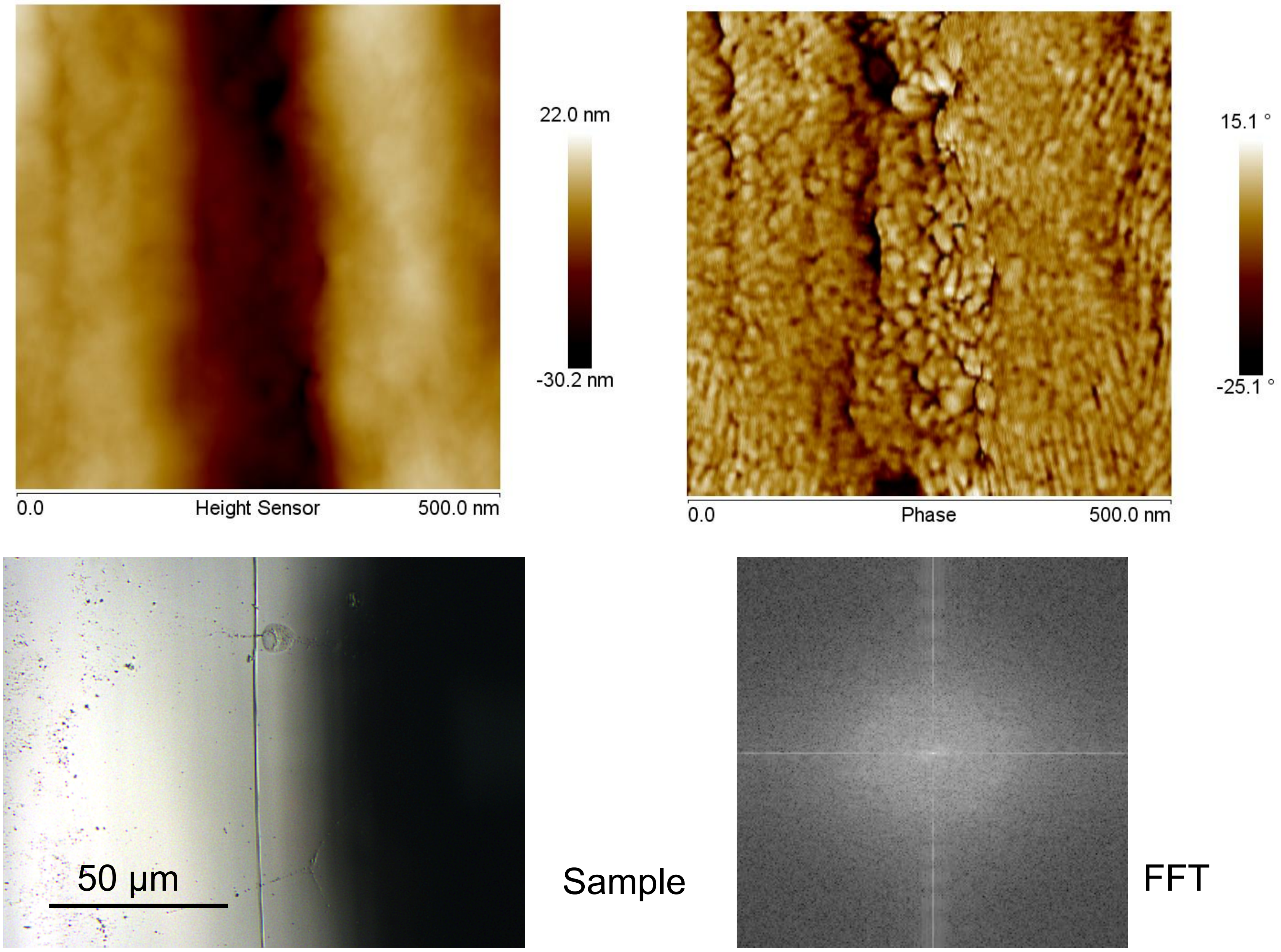

Figure S79 – Individual 6, Unstretched, Fiber 14, Image 71, 2018/03/02

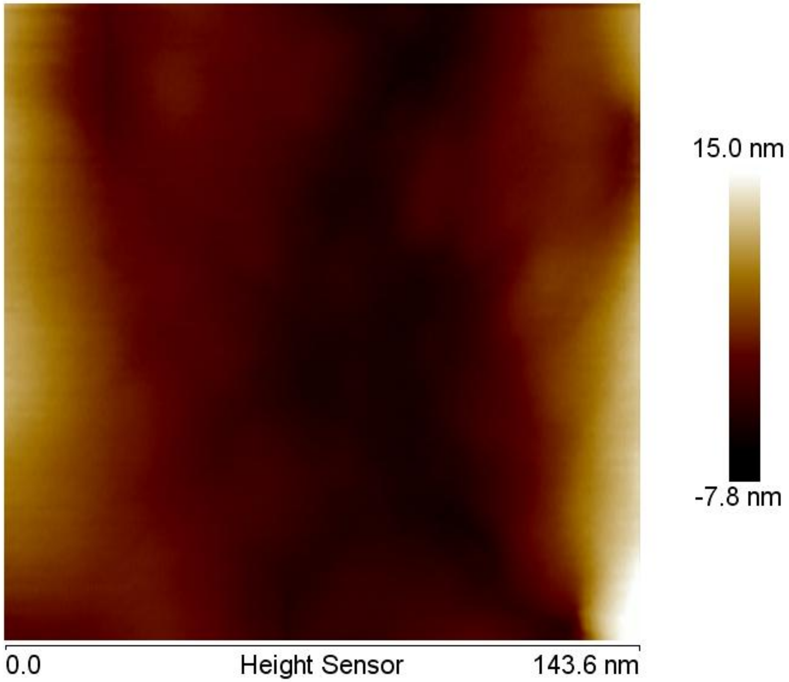

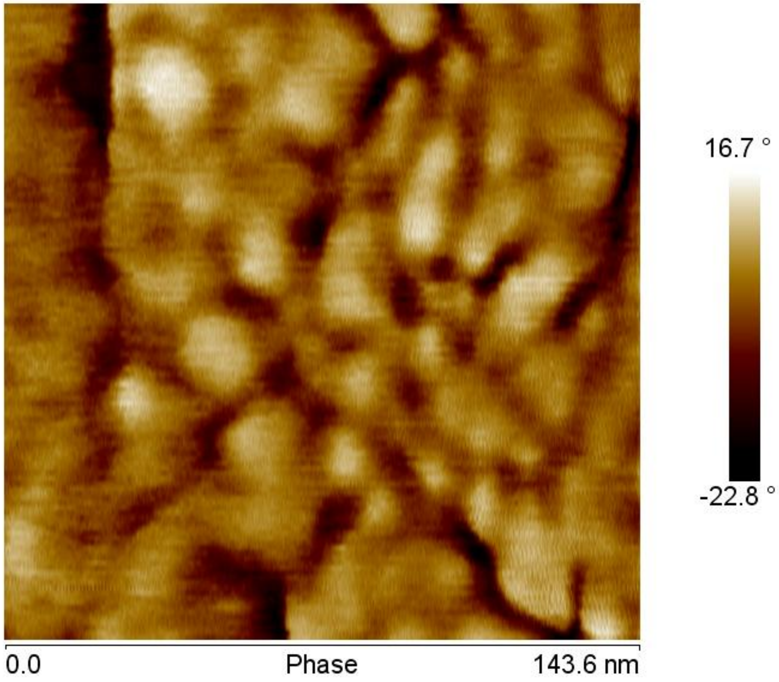

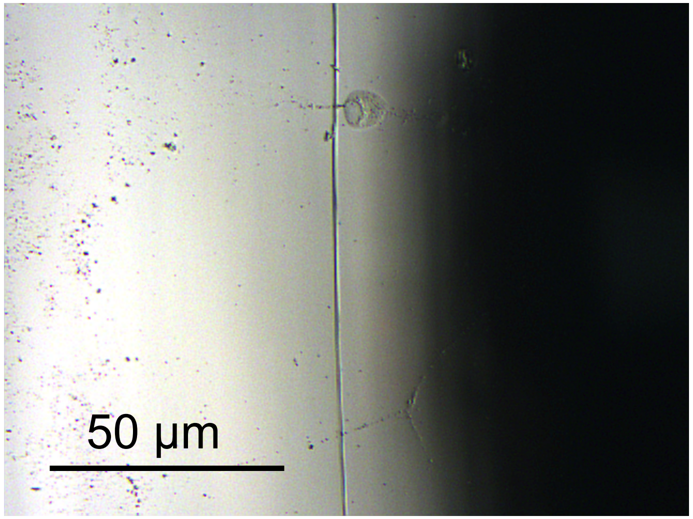

Sample

Figure S80 – Individual 6, Unstretched, Fiber 14, Image 72, 2018/03/02

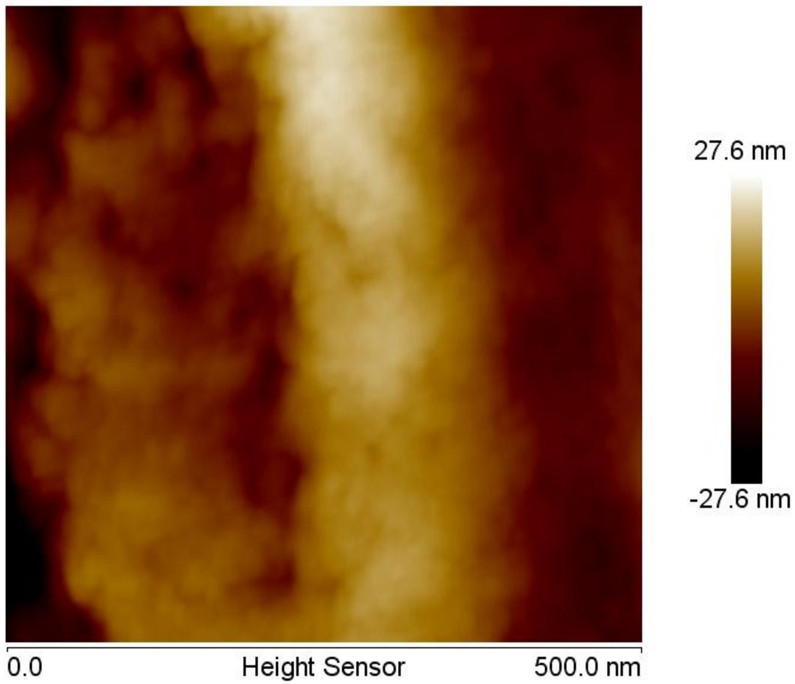

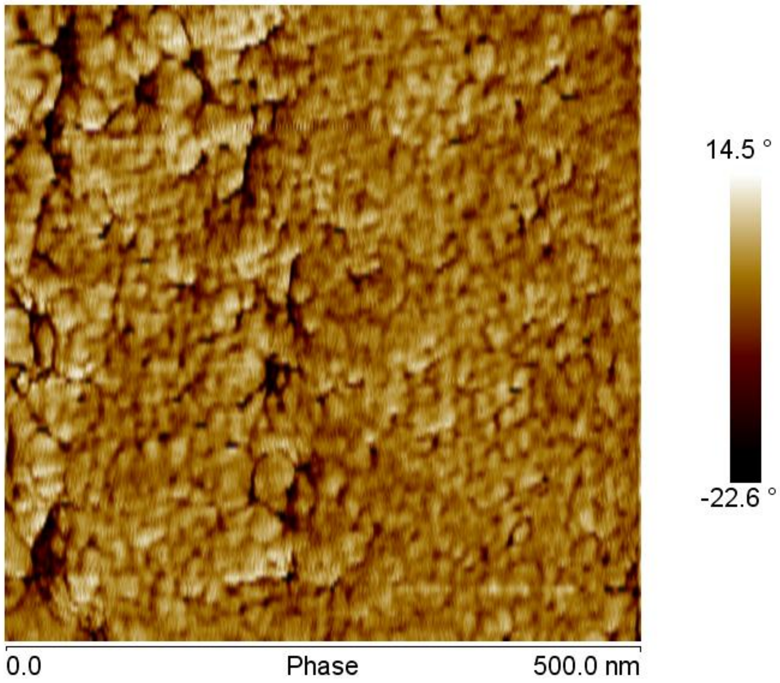

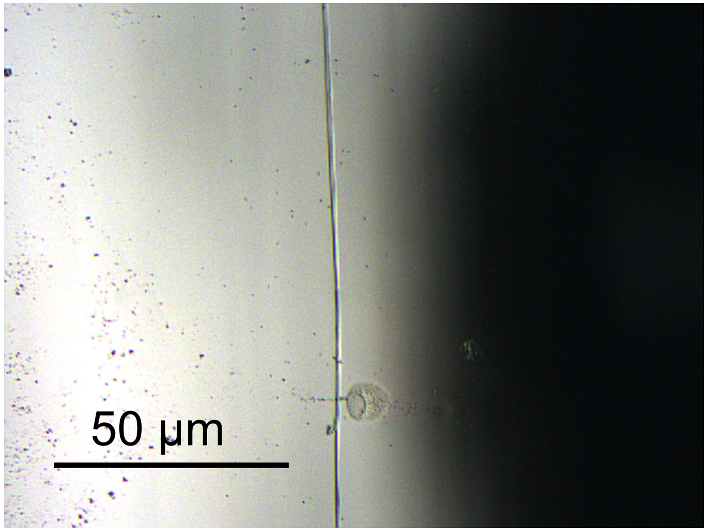

Sample

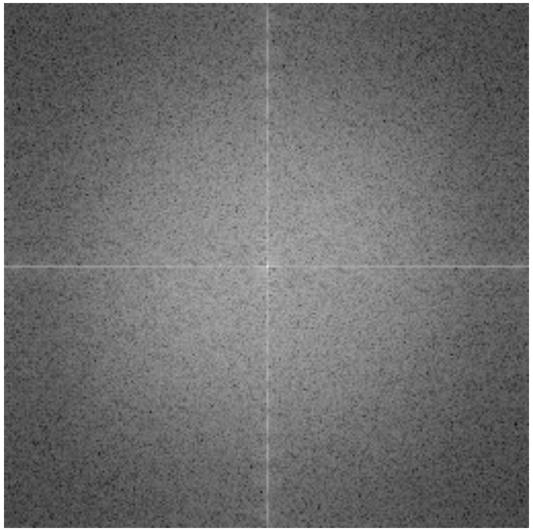

FFT

Figure S81 – Individual 6, Unstretched, Fiber 14, Image 73, 2018/03/02

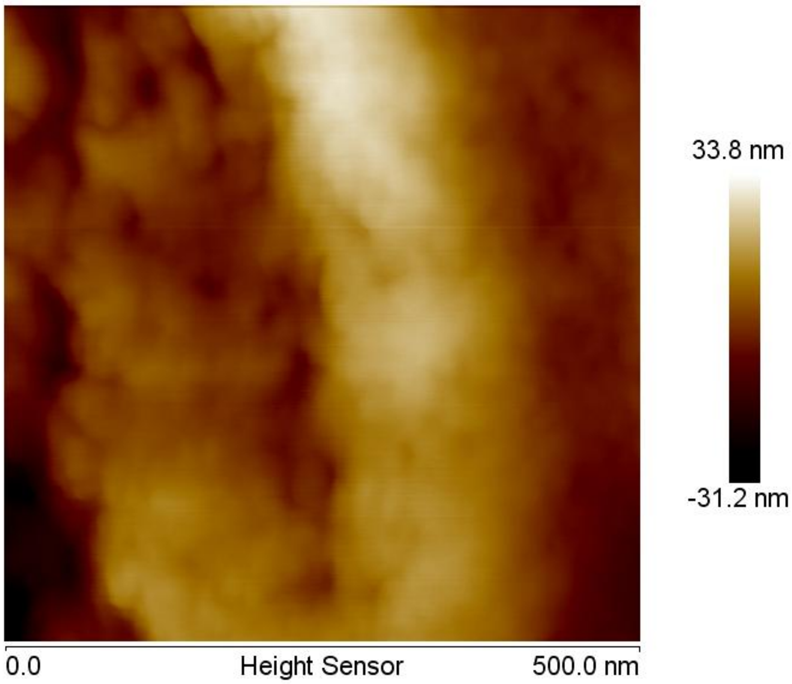

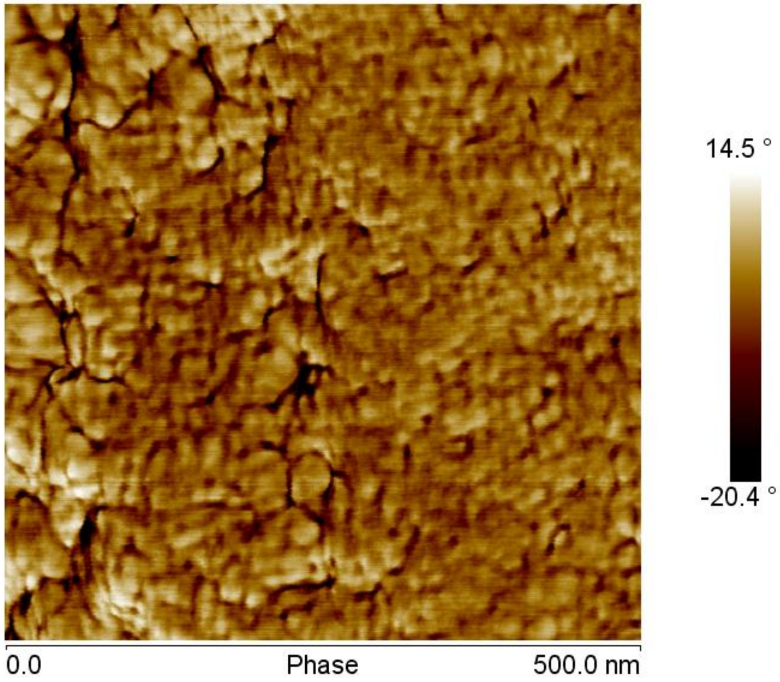

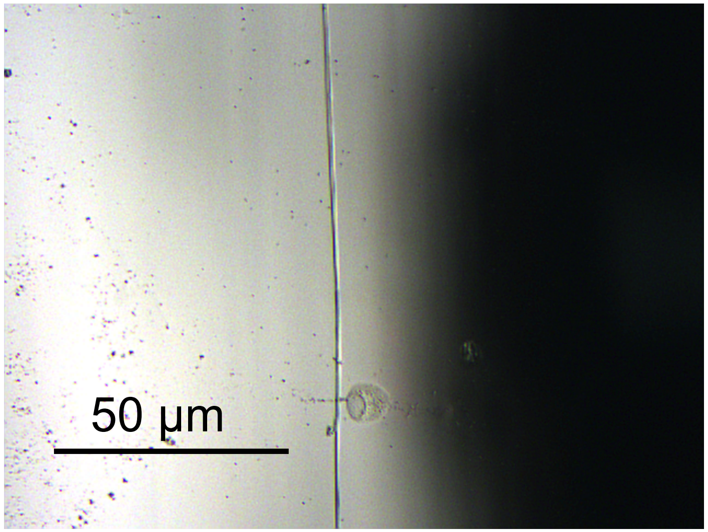

Sample

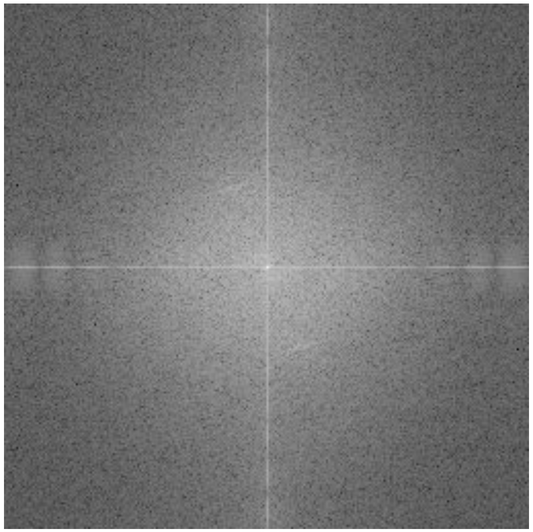

FFT

Figure S82 – Individual 6, Unstretched, Fiber 14, Image 74, 2018/03/22

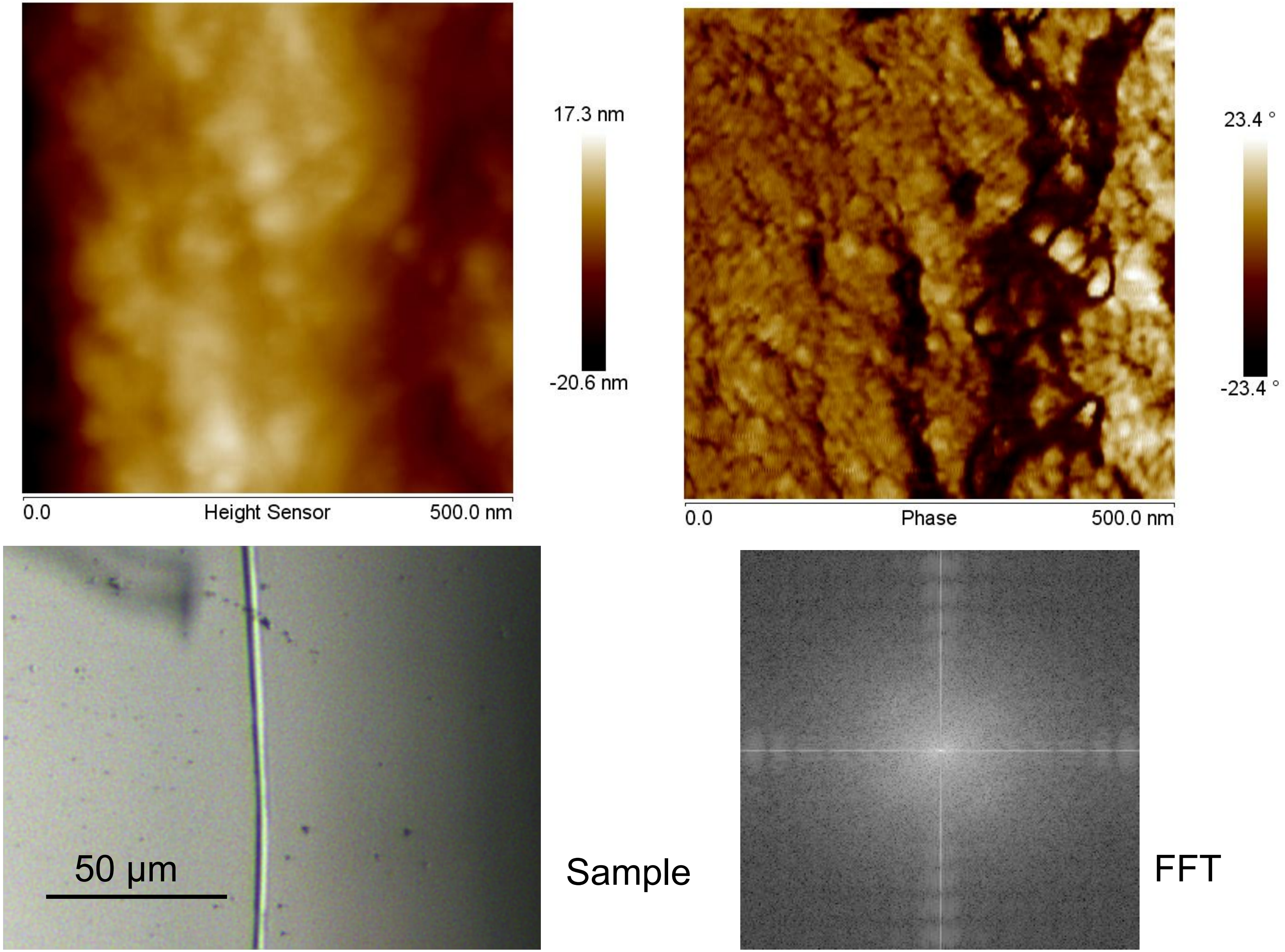

Figure S83 – Individual 7, Unstretched, Fiber 15, Image 75, 2018/03/22

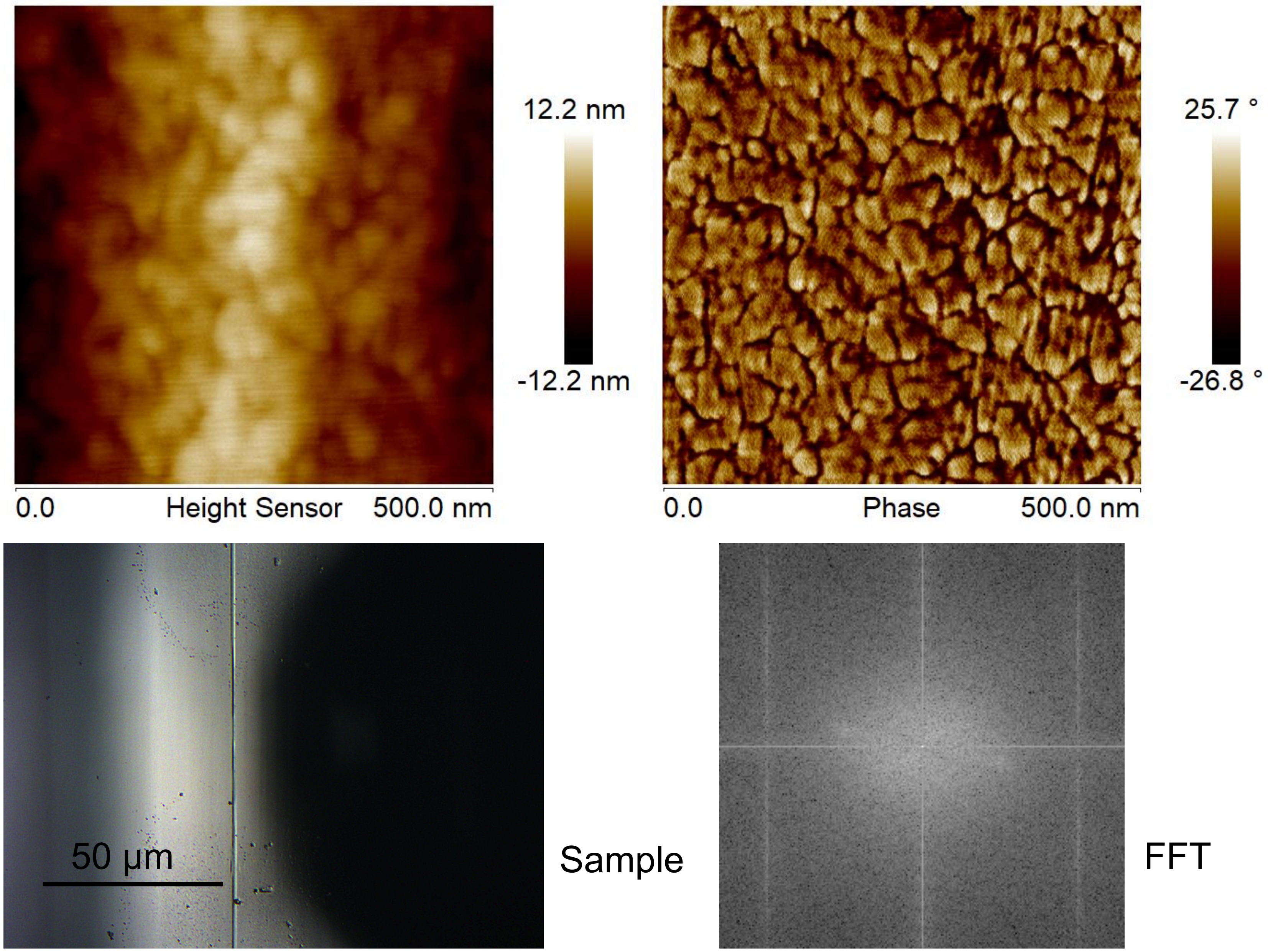

Figure S84 – Individual 7, Unstretched, Fiber 15, Image 76, 2018/03/22

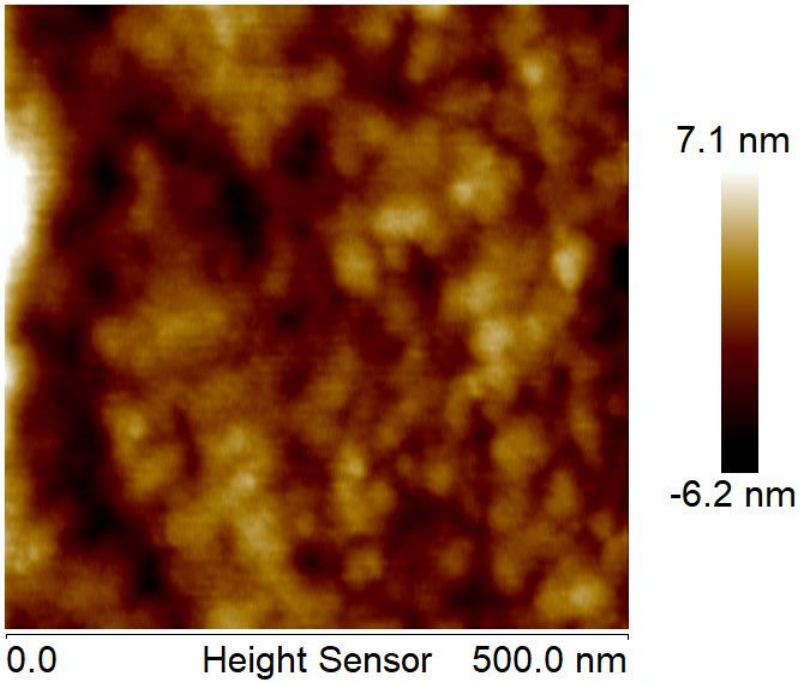

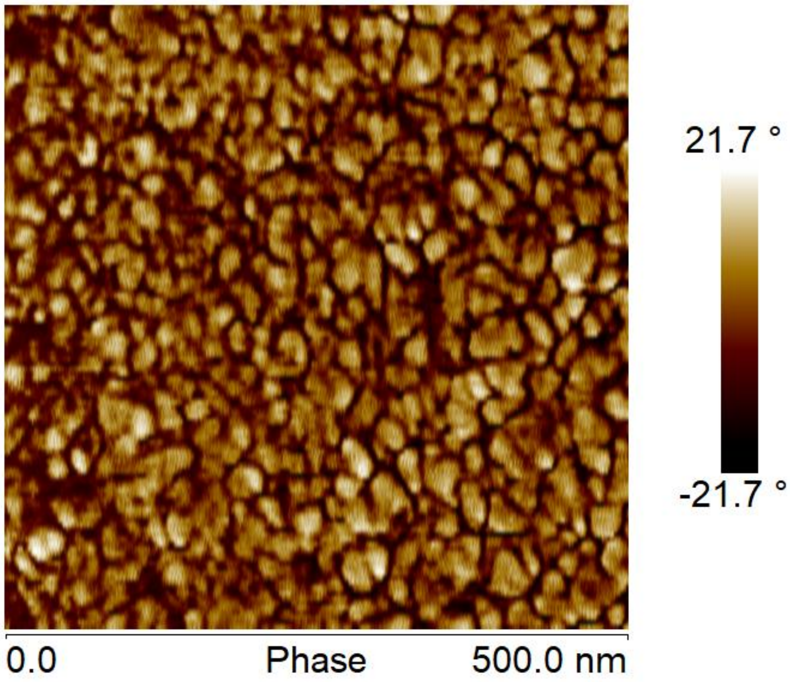

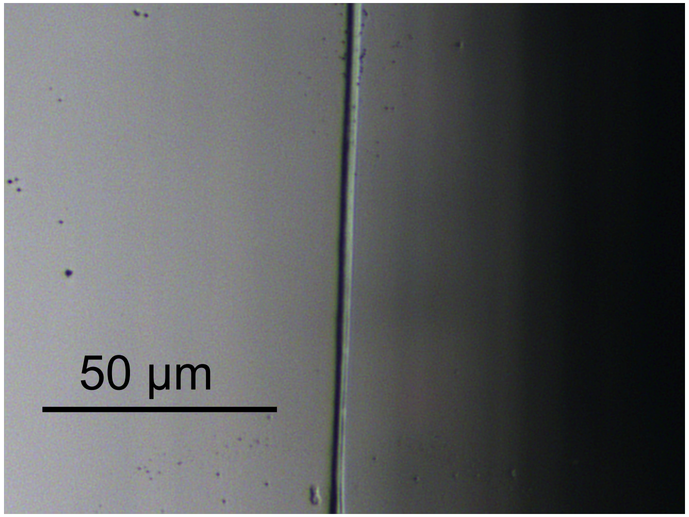

Sample

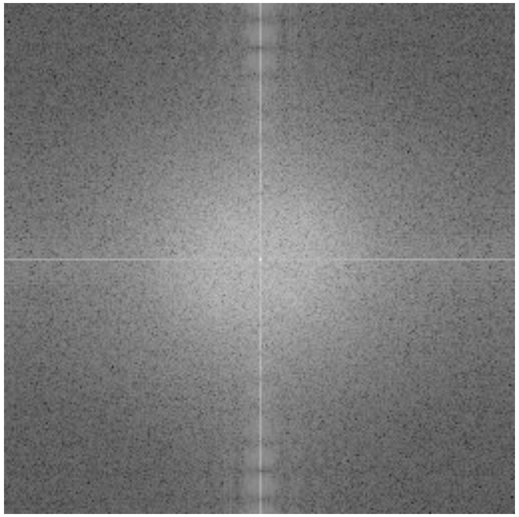

FFT

Figure S85 – Individual 7, Unstretched, Fiber 15, Image 77, 2018/03/22

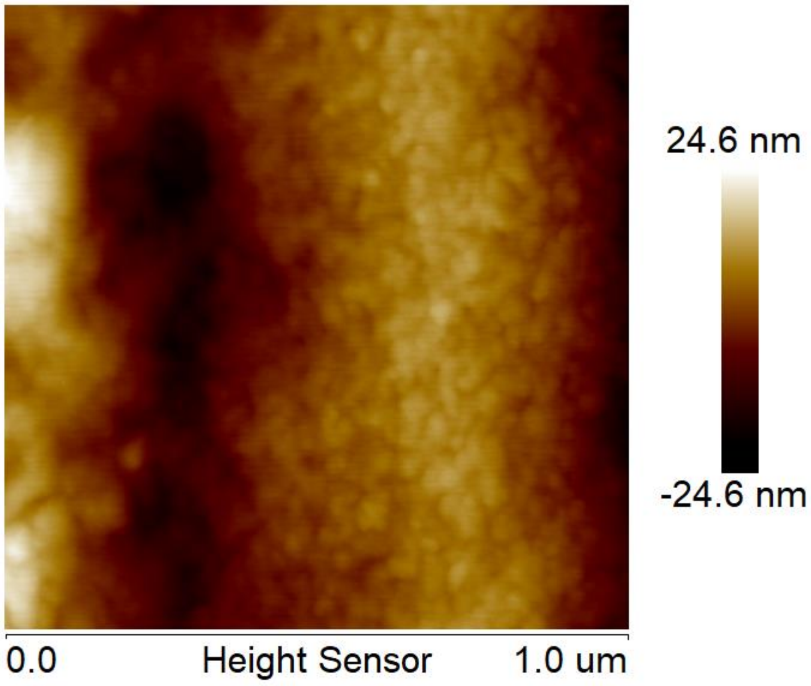

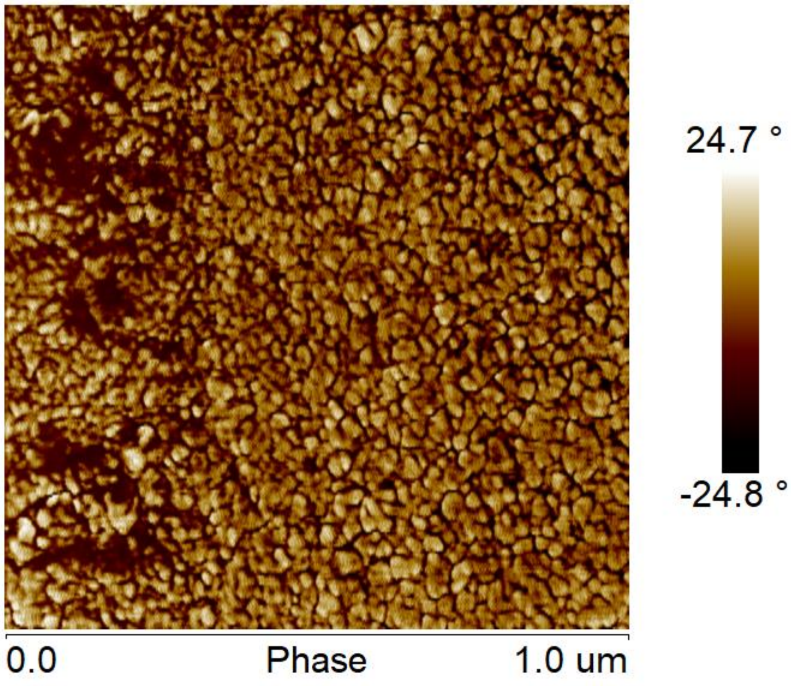

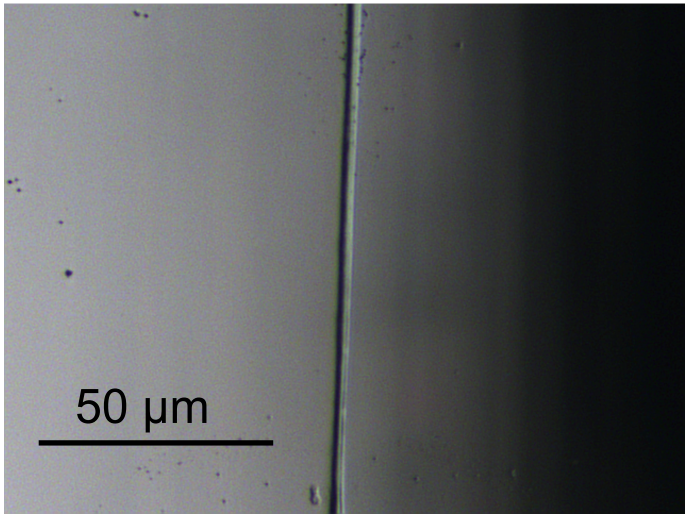

Sample

Figure S86 – Individual 8, Stretched, Fiber 16, Image 78, 2018/03/22

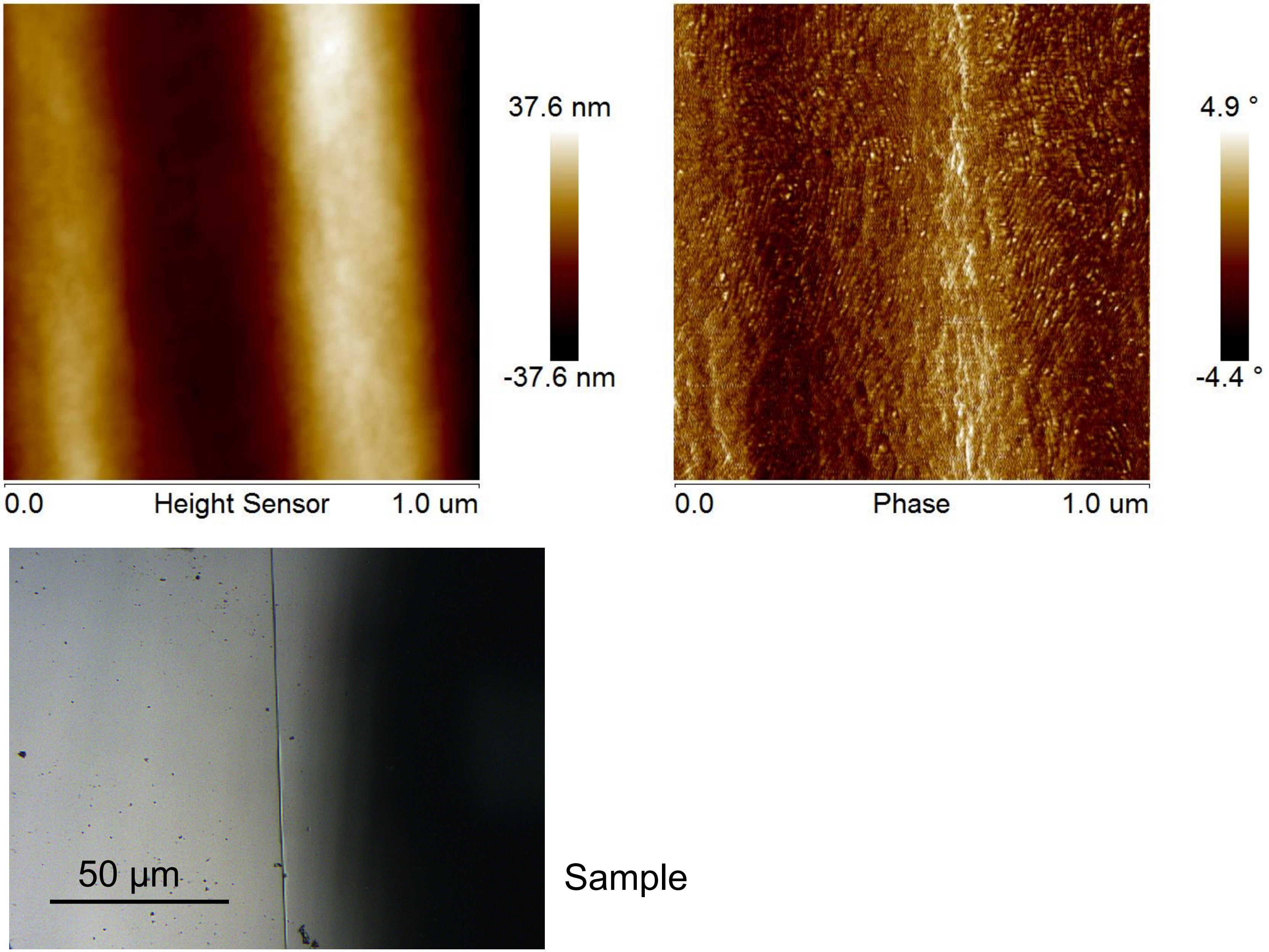

Figure S87 – Individual 9, Stretched, Fiber 16, Image 79, 2018/03/22

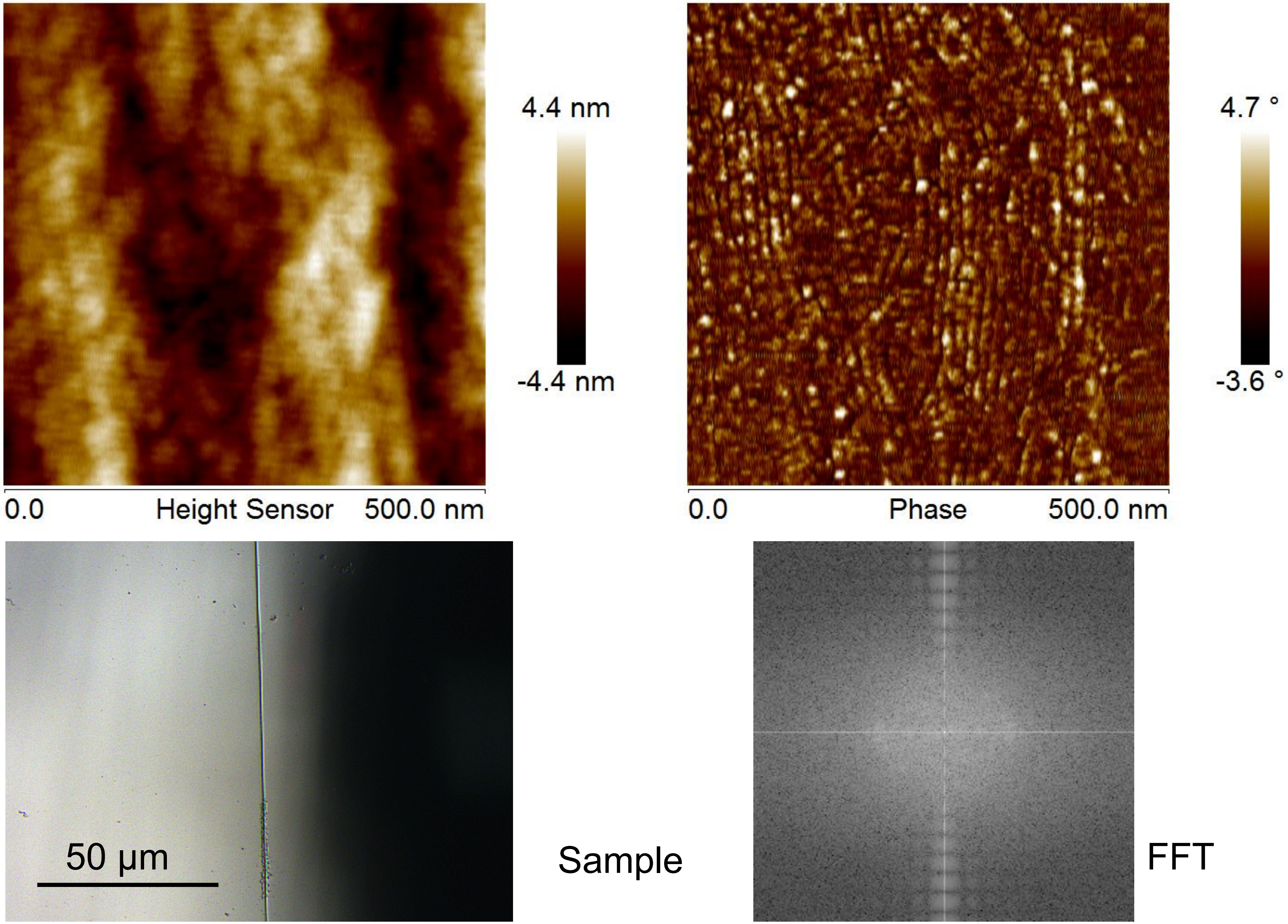

Figure S88 – Individual 9, Stretched, Fiber 16, Image 80, 2018/03/22

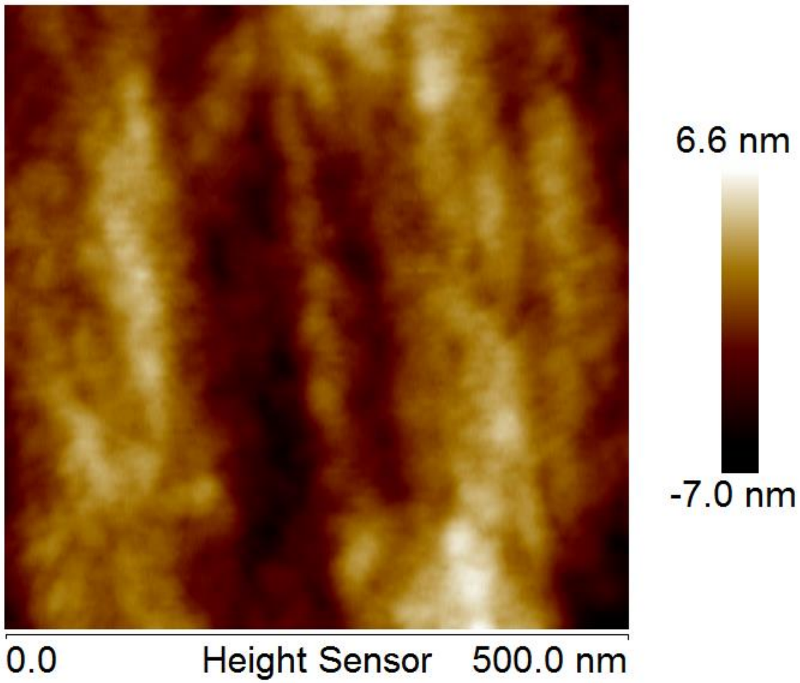

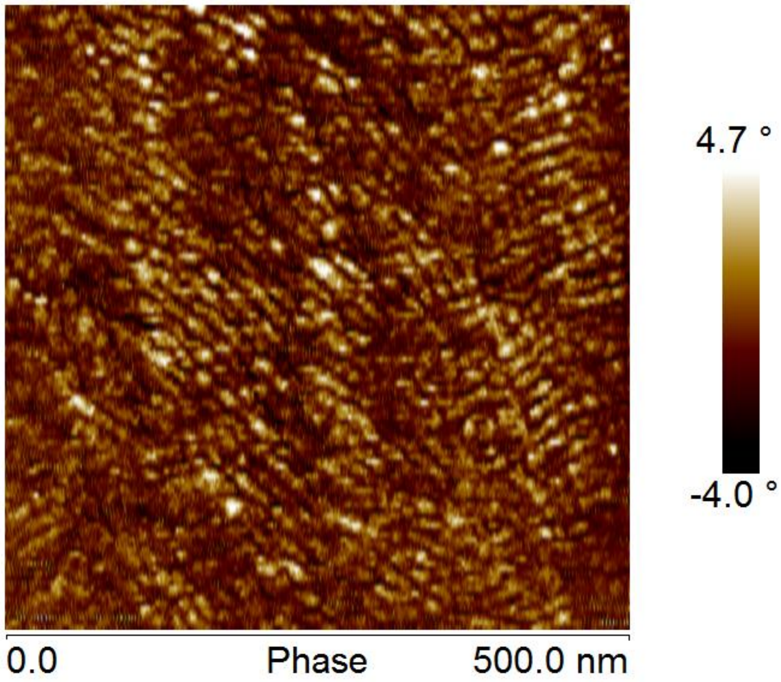

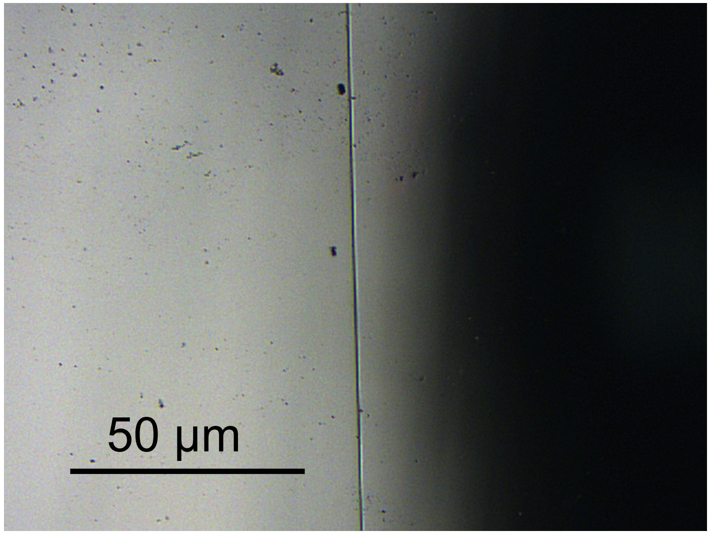

Sample

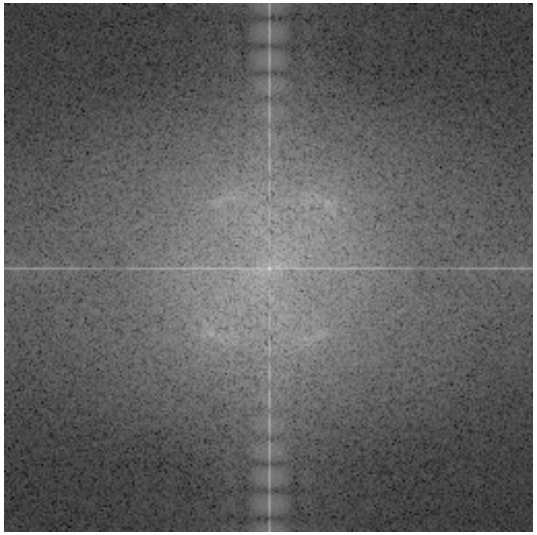

FFT

Figure S89 – Individual 9, Stretched, Fiber 16, Image 81, 2018/03/22

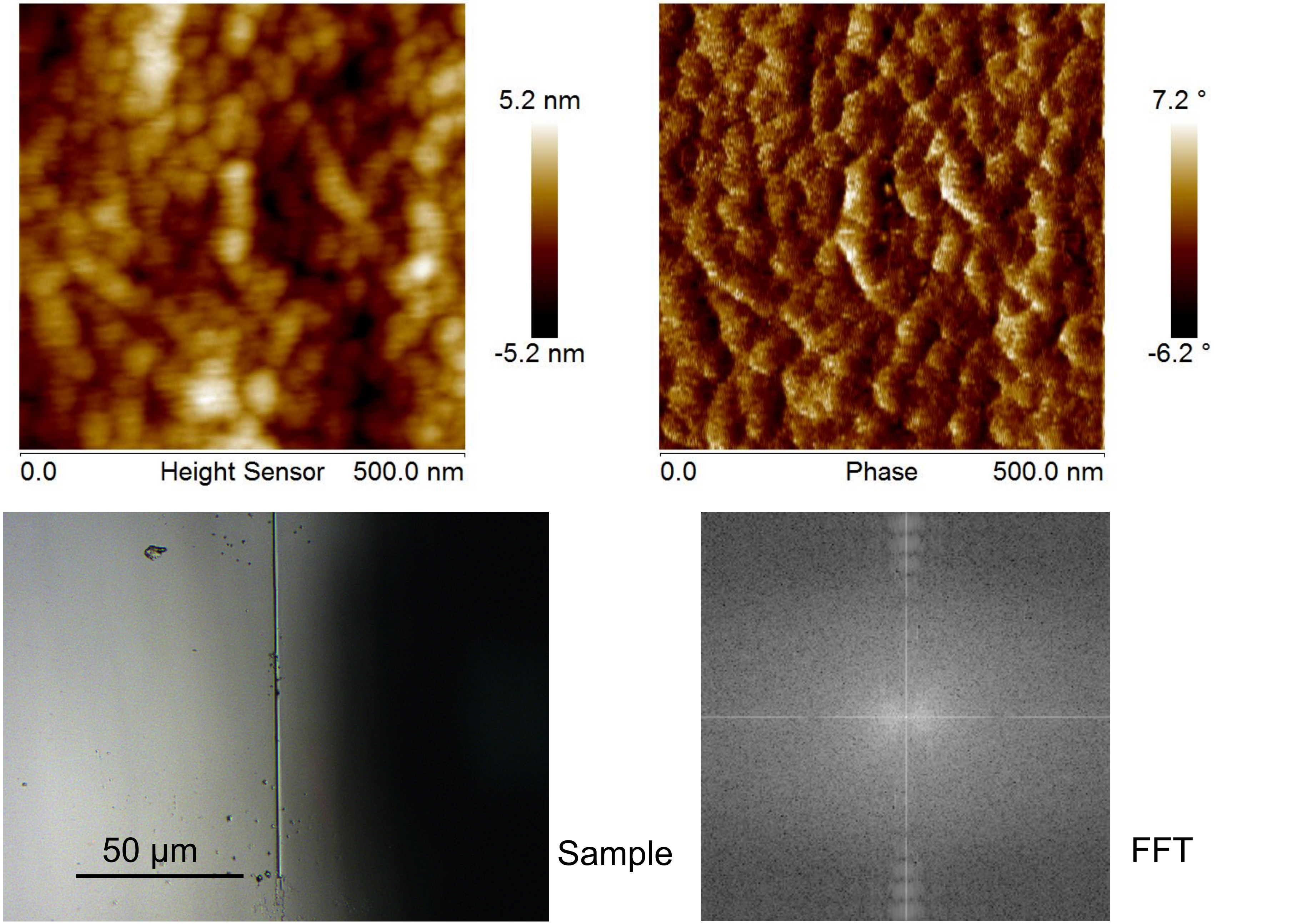

Figure S90 – Individual 9, Stretched, Fiber 16, Image 82, 2018/03/22

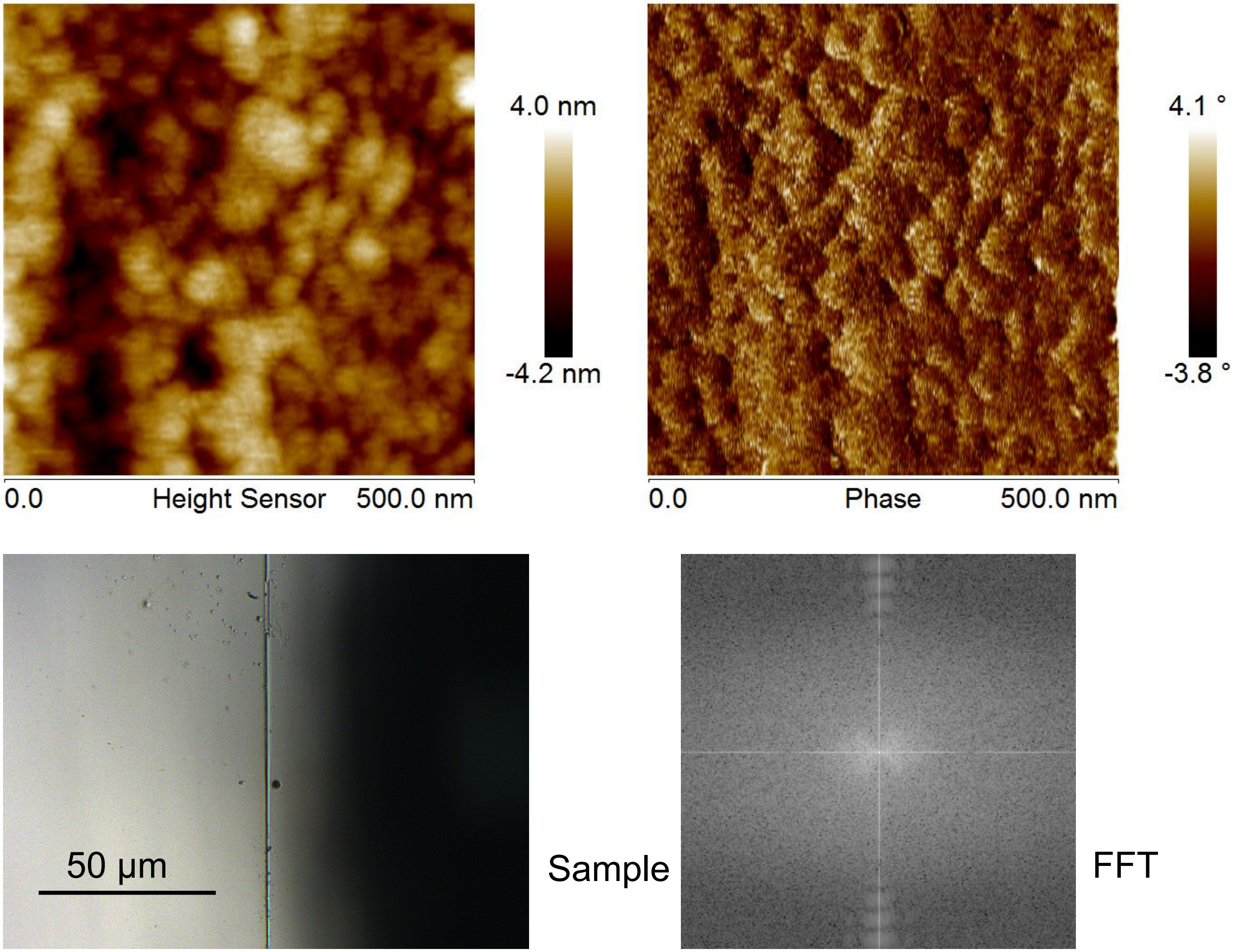

Figure S91 – Individual 9, Stretched, Fiber 16, Image 83, 2018/03/22

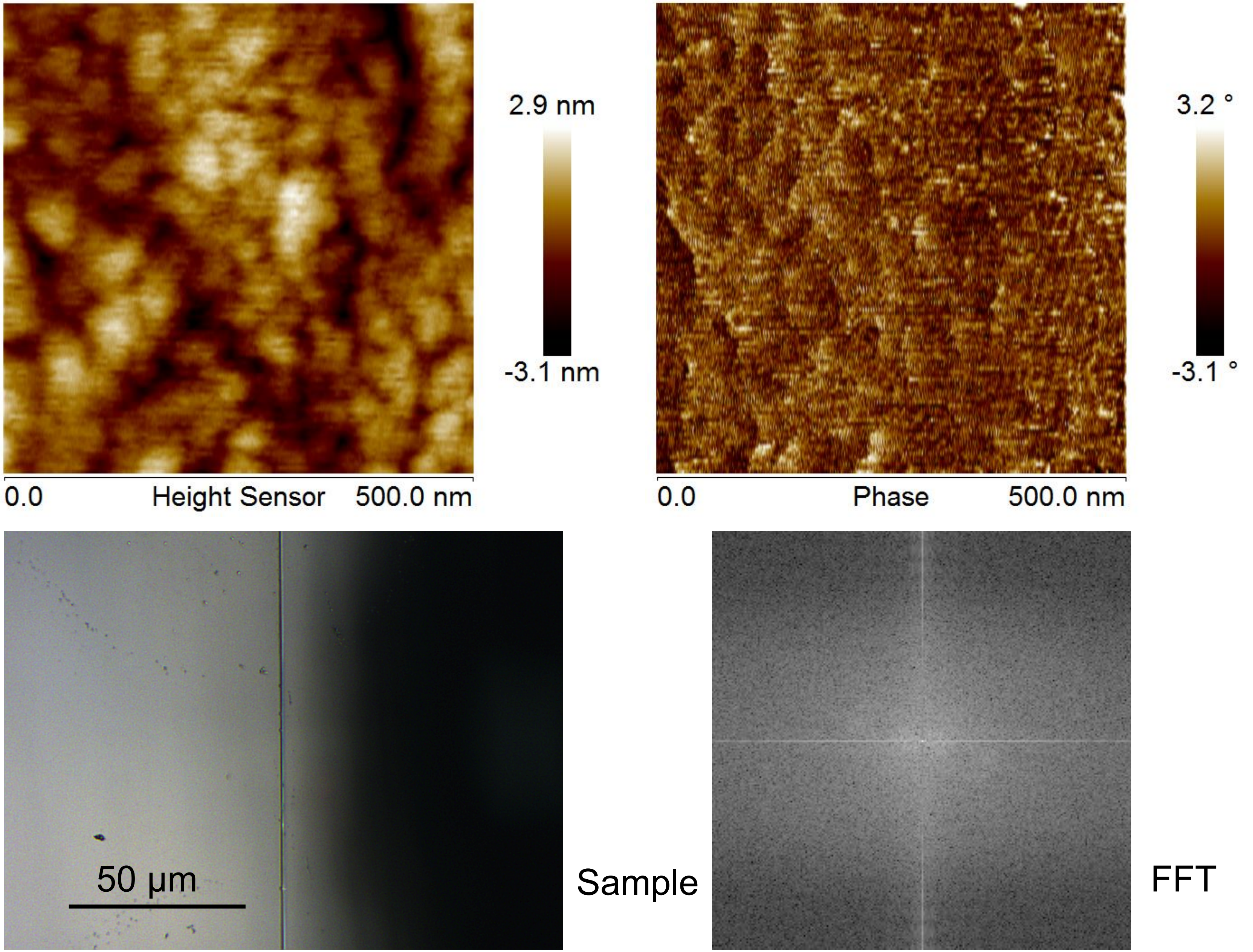

Figure S92 – Individual 9, Stretched, Fiber 16, Image 84, 2018/03/22

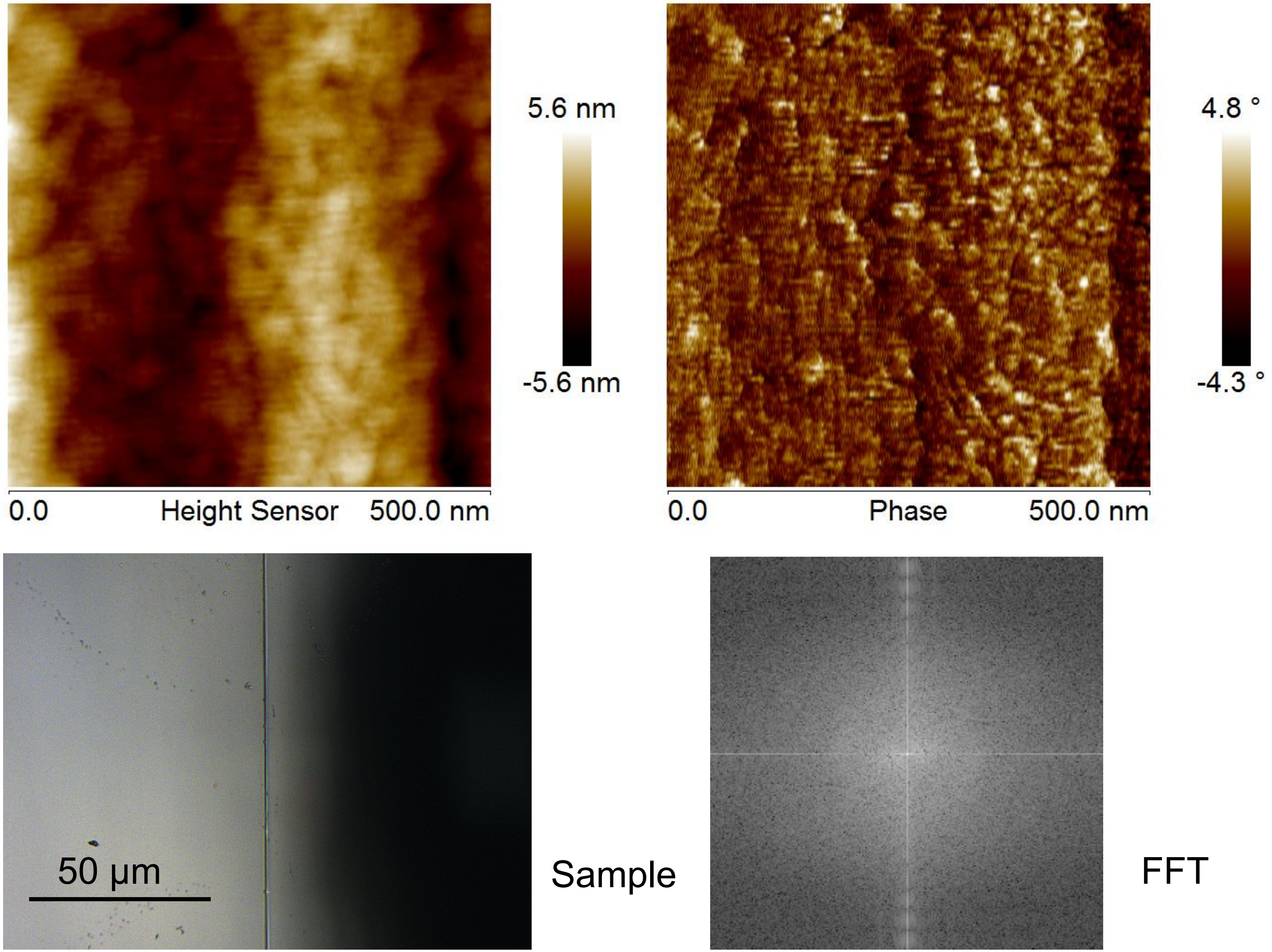

Figure S93 – Individual 10, Stretched, Fiber 17, Image 85, 2018/03/22

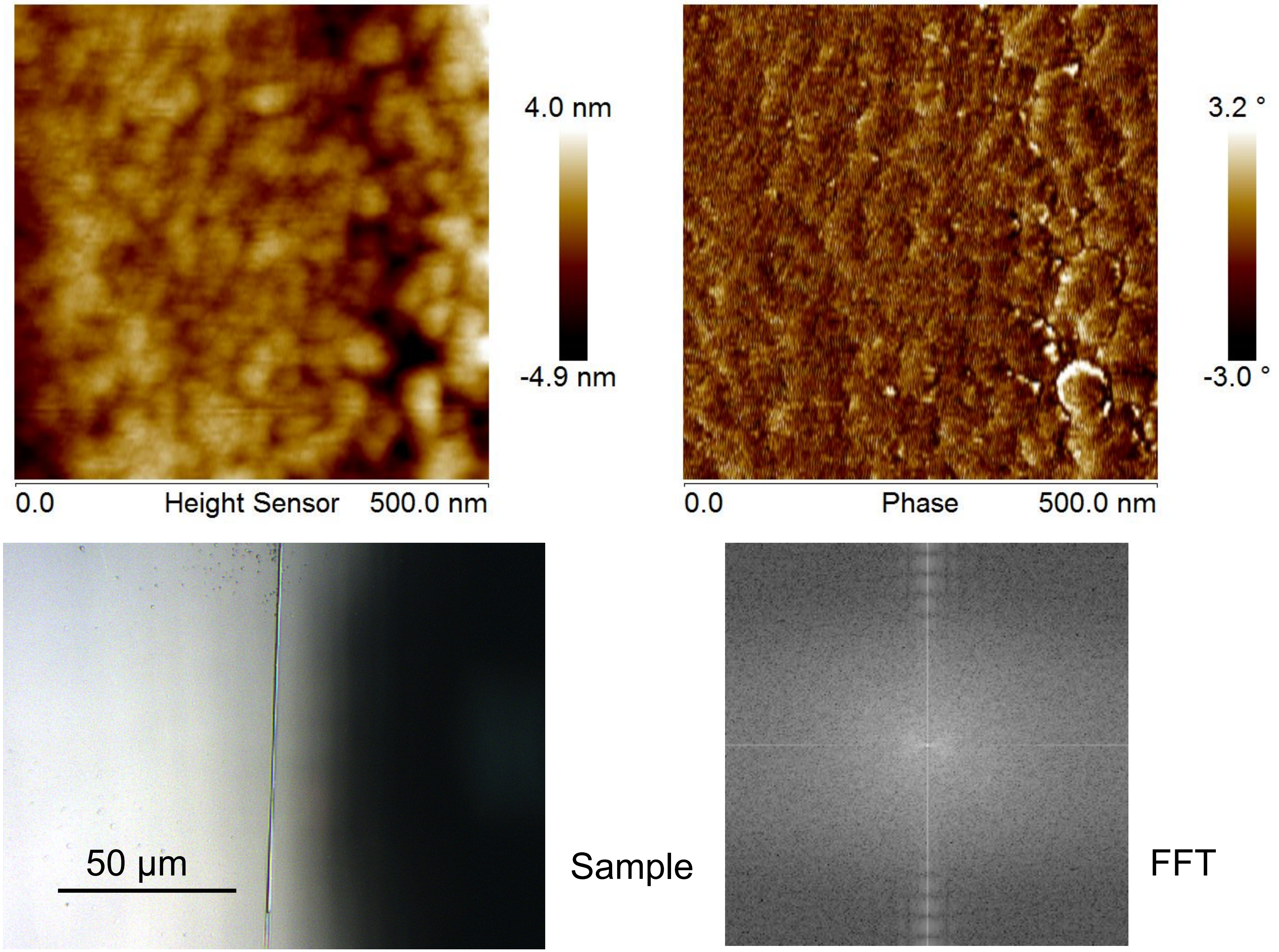

Figure S94 – Individual 10, Stretched, Fiber 17, Image 86, 2018/03/22

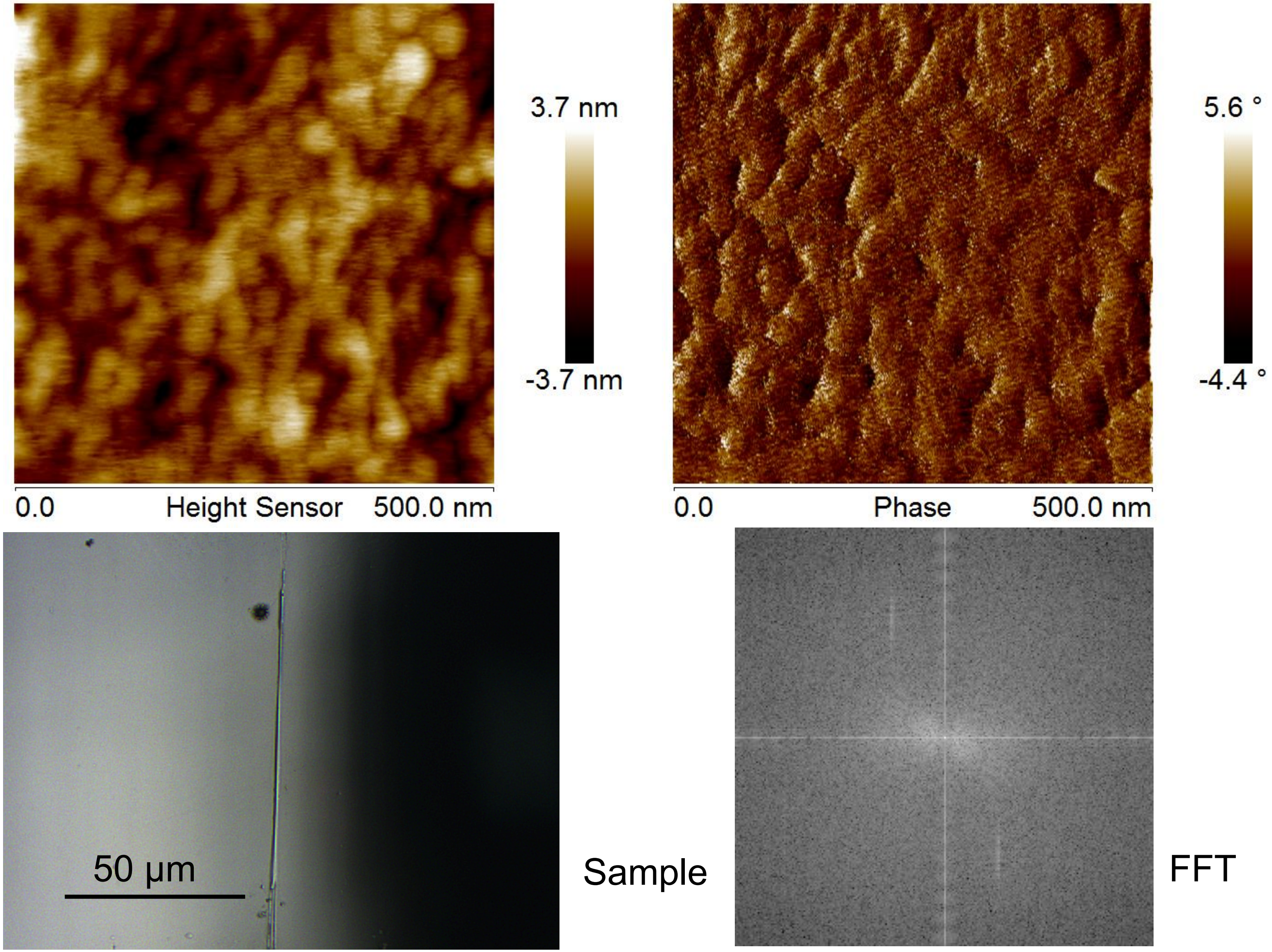

Figure S95 – Individual 10, Stretched, Fiber 17, Image 87, 2018/03/22

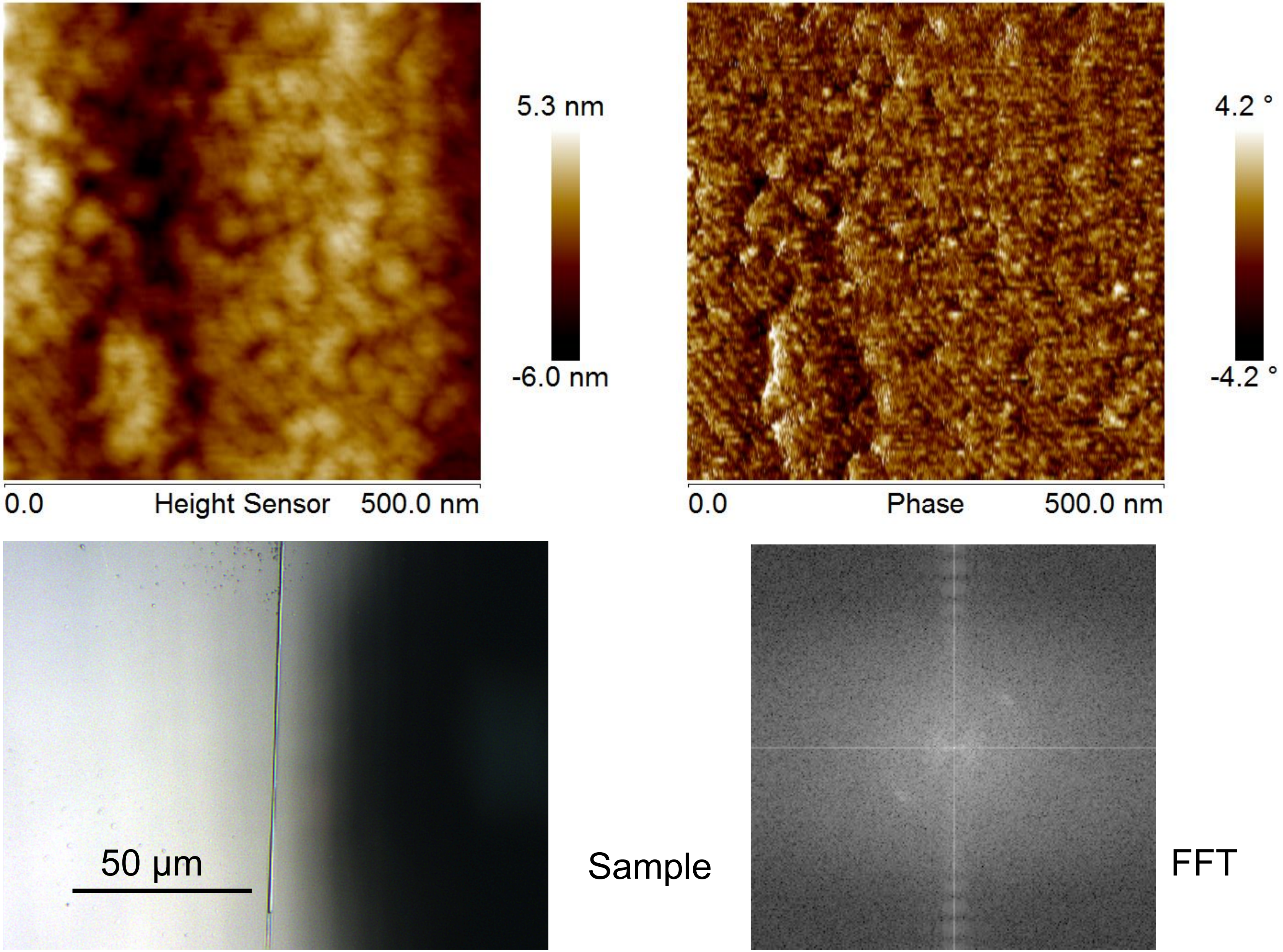

Figure S96 – Individual 10, Unstretched, Fiber 18, Image 88, 2018/03/22

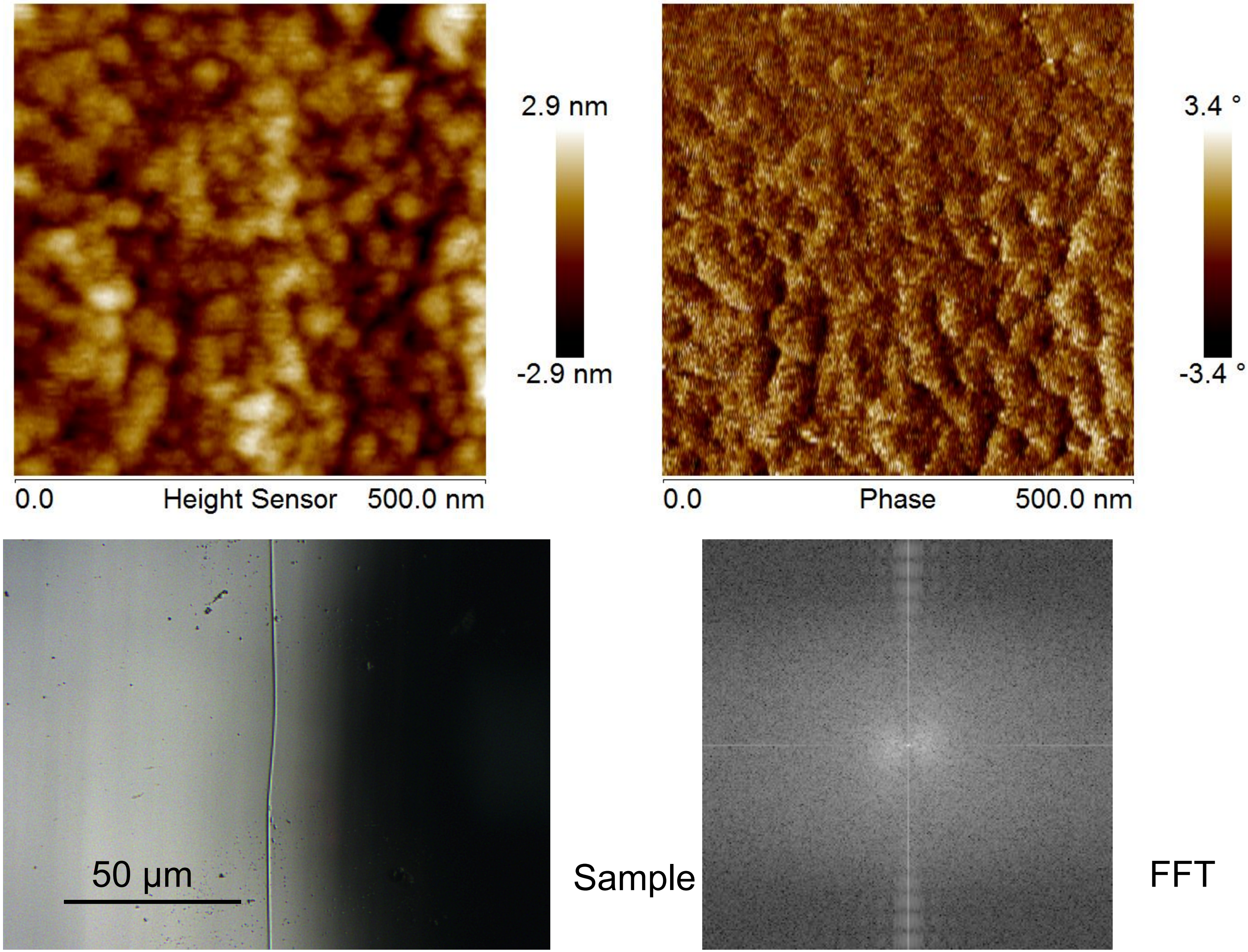

Figure S97 – Individual 9, Unstretched, Fiber 19, Image 89, 2018/03/22

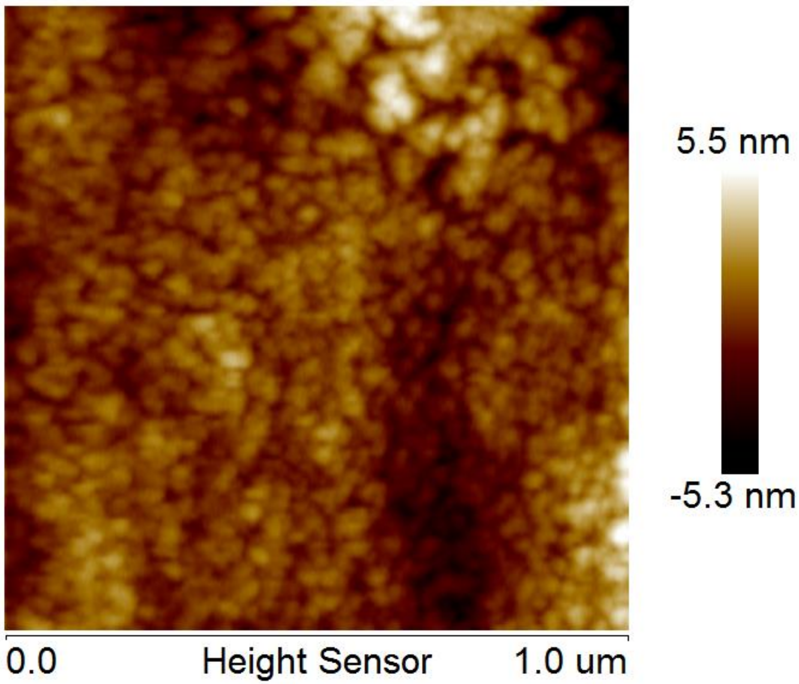

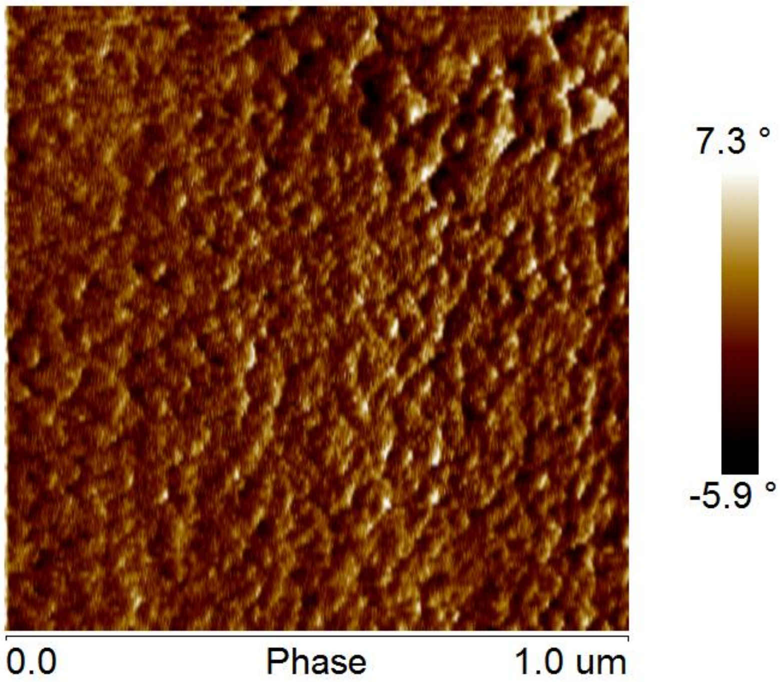

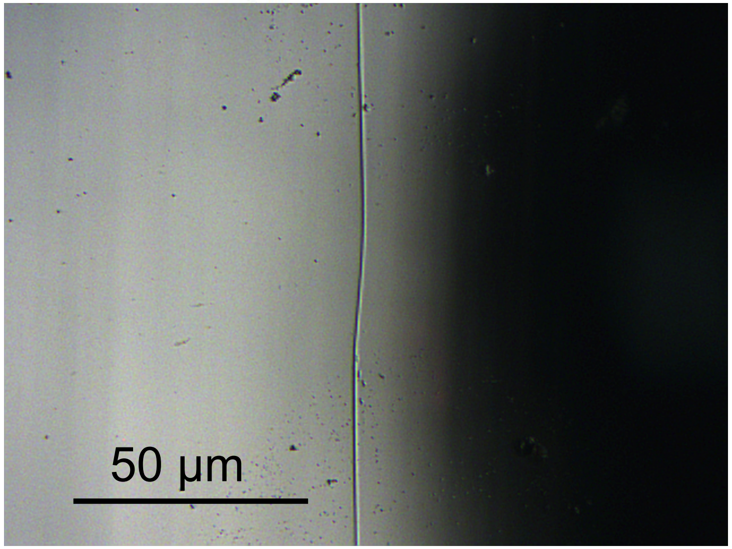

Sample

Figure S98 – Individual 9, Unstretched, Fiber 18, Image 90, 2018/03/22

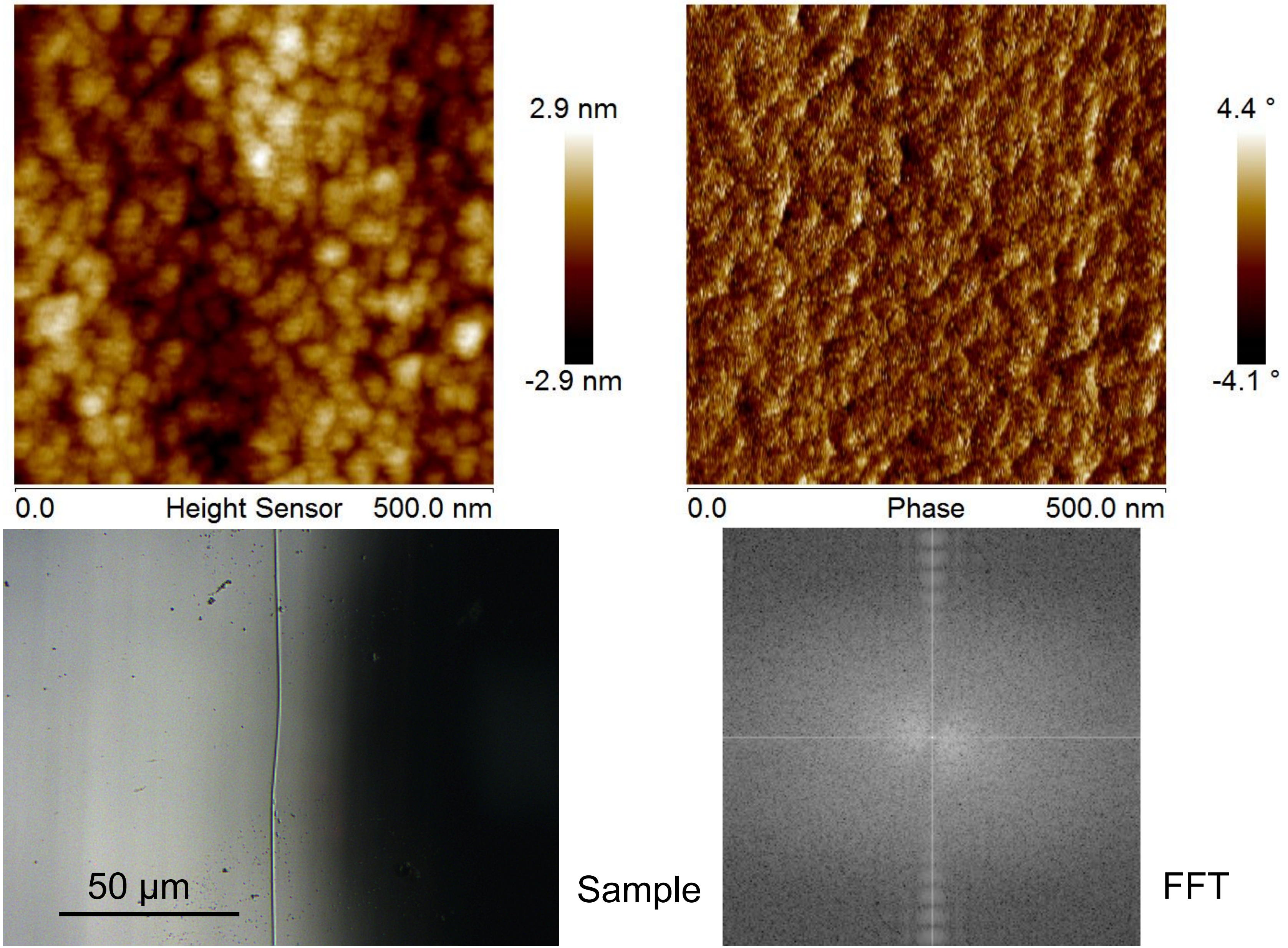

Figure S99 – Individual 10, Unstretched, Fiber 19, Image 91, 2018/03/22

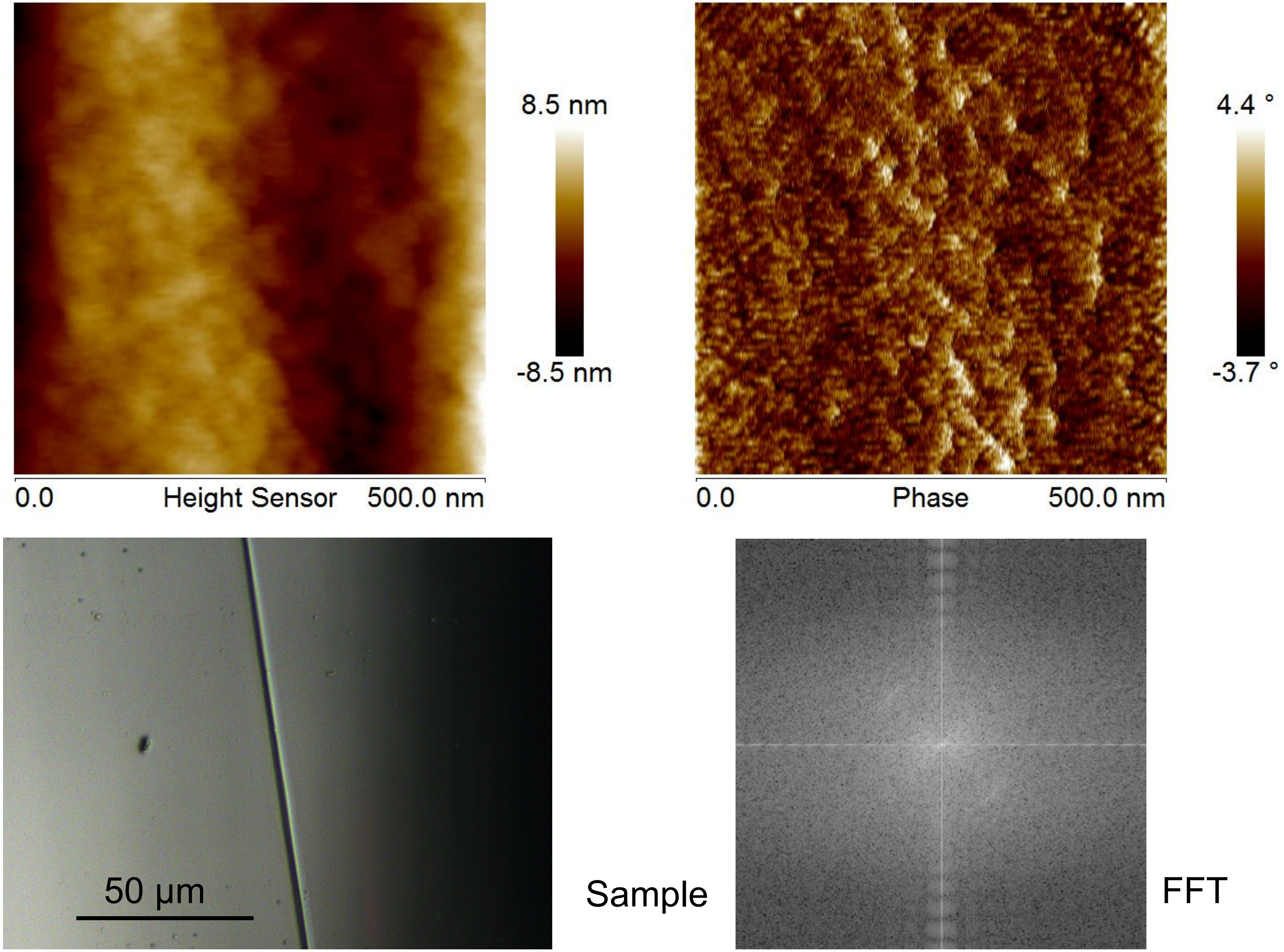

Figure S100 – Individual 10, Unstretched, Fiber 19, Image 92, 2018/03/22

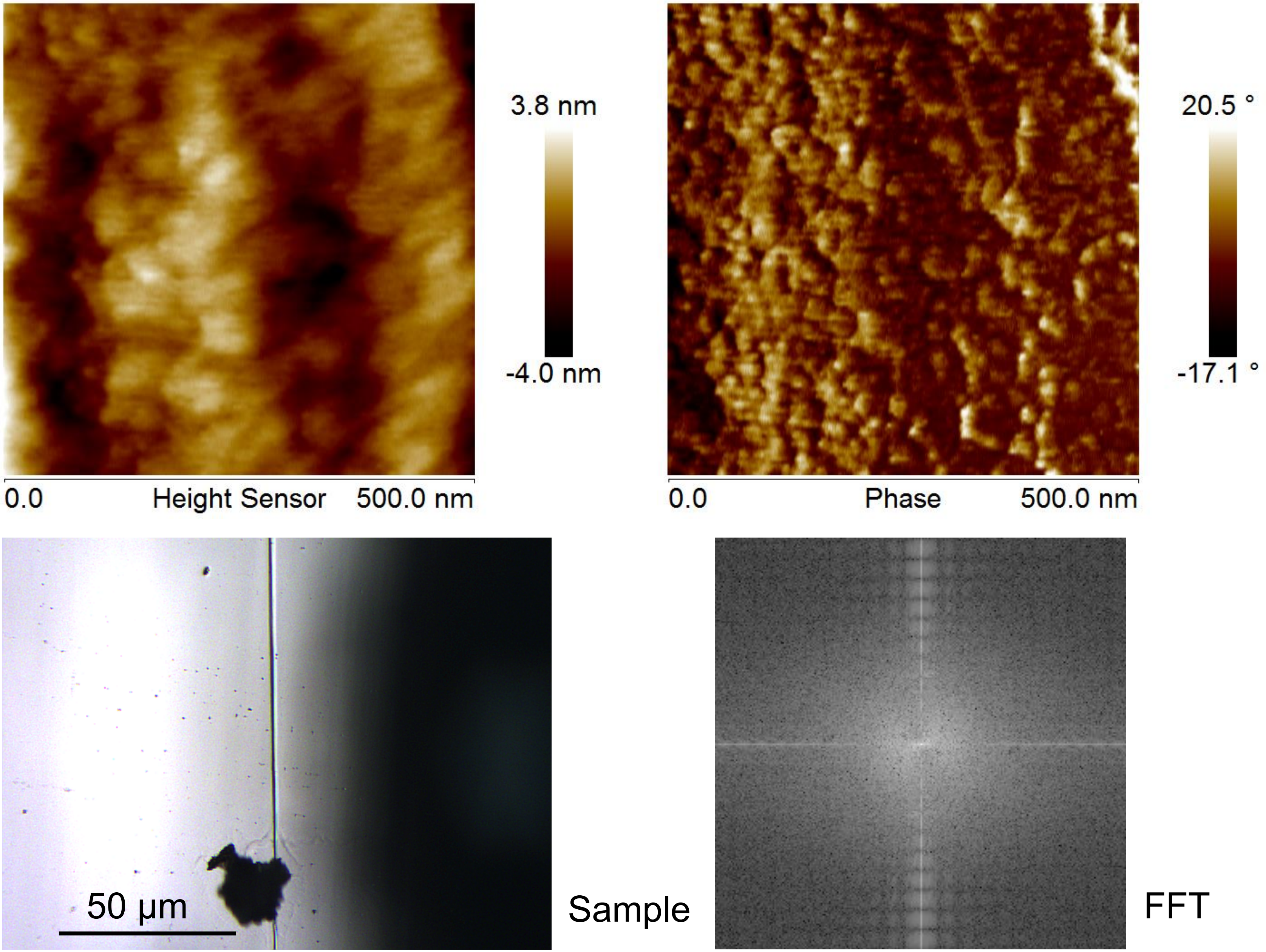

Figure S101 – Individual 10, Unstretched, Fiber 19, Image 93, 2018/03/22

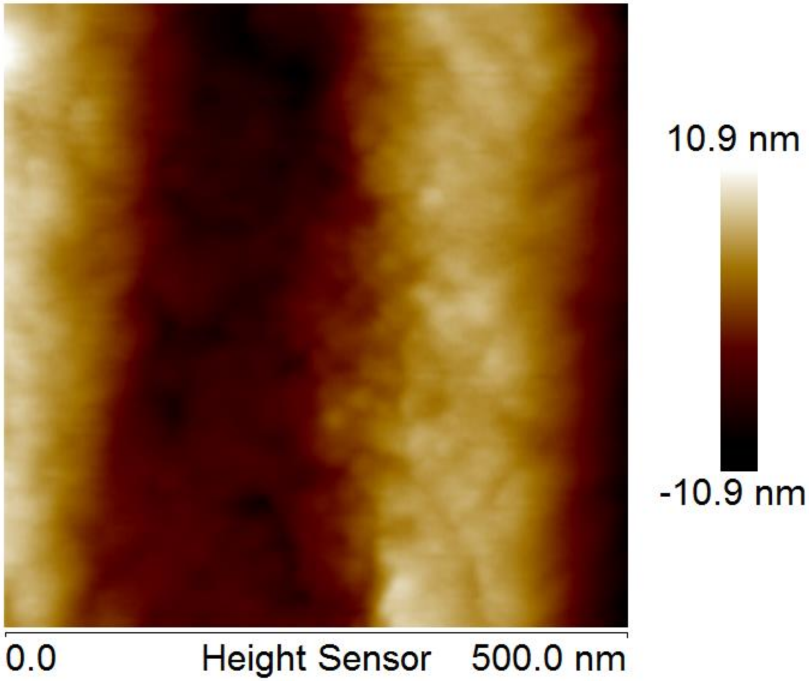

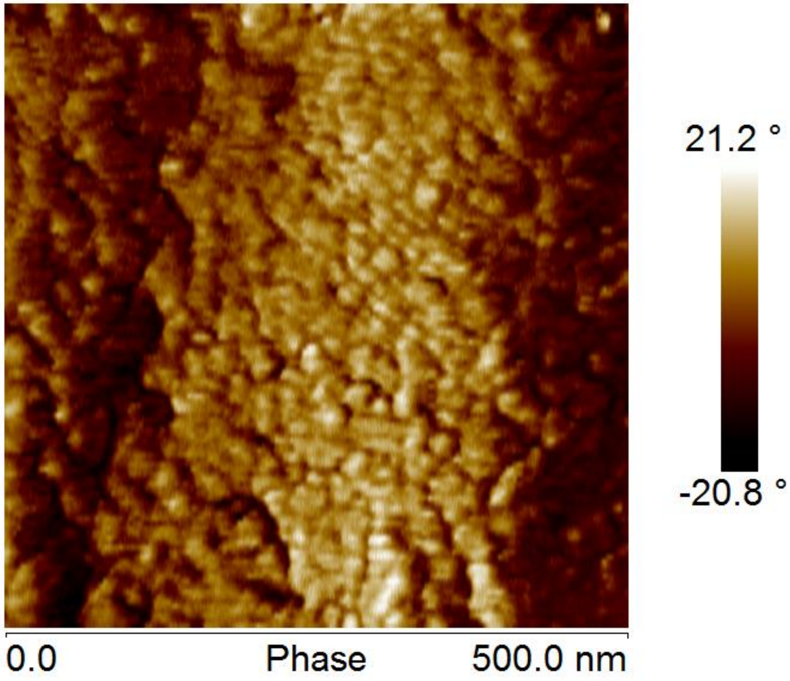

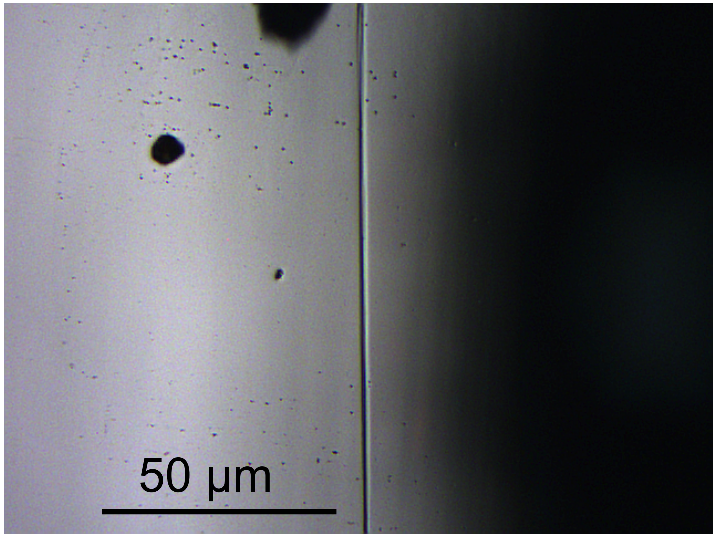

Sample

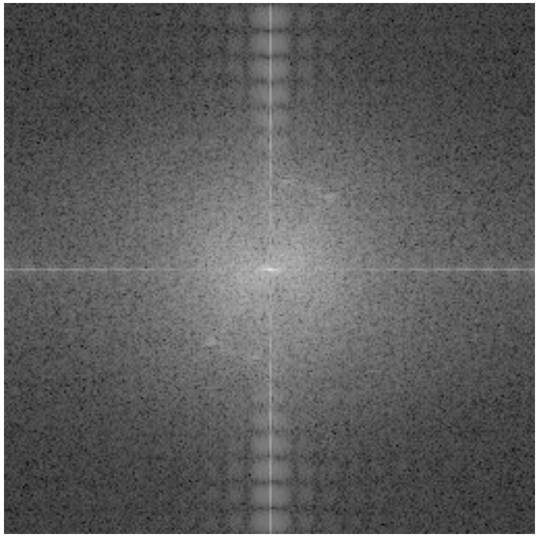

FFT

Figure S102 – Individual 10, Unstretched, Fiber 19, Image 94, 2018/03/22

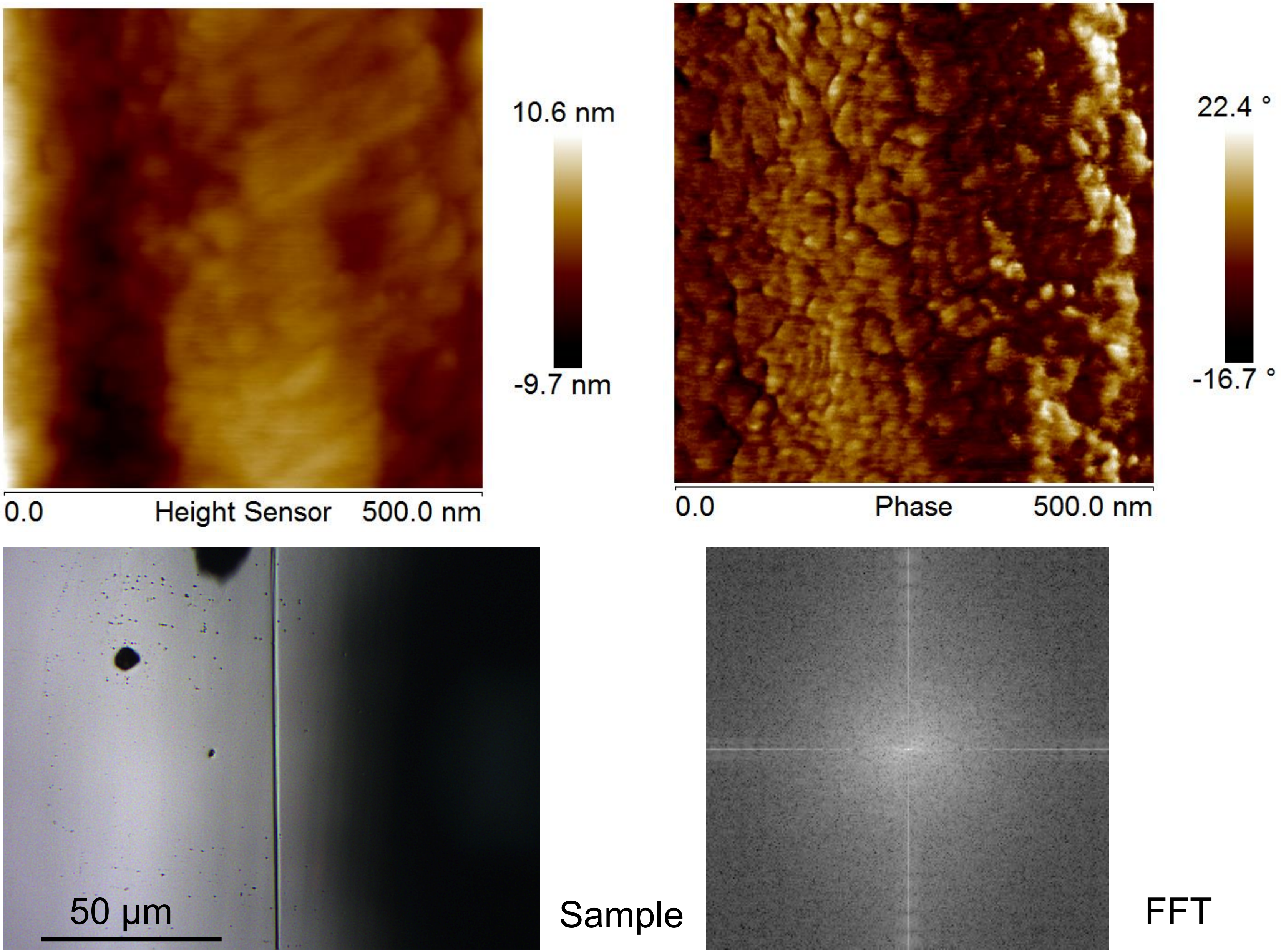

Figure S103 – Glass surface image 95, 2017/12/18

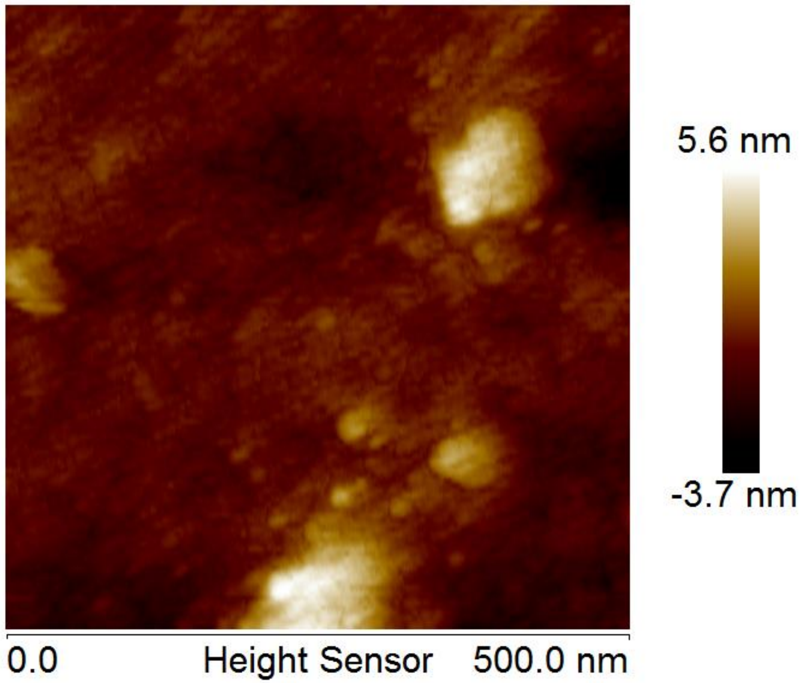

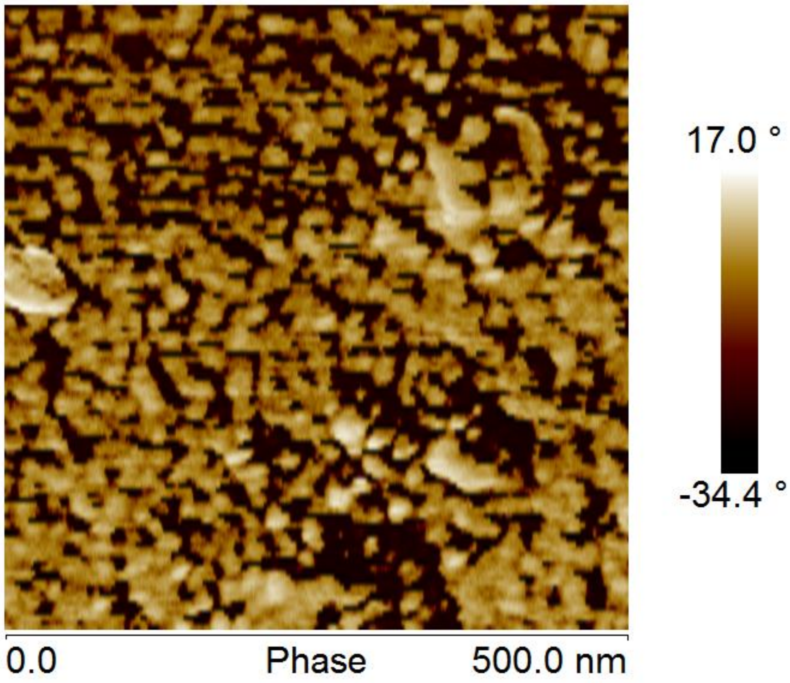

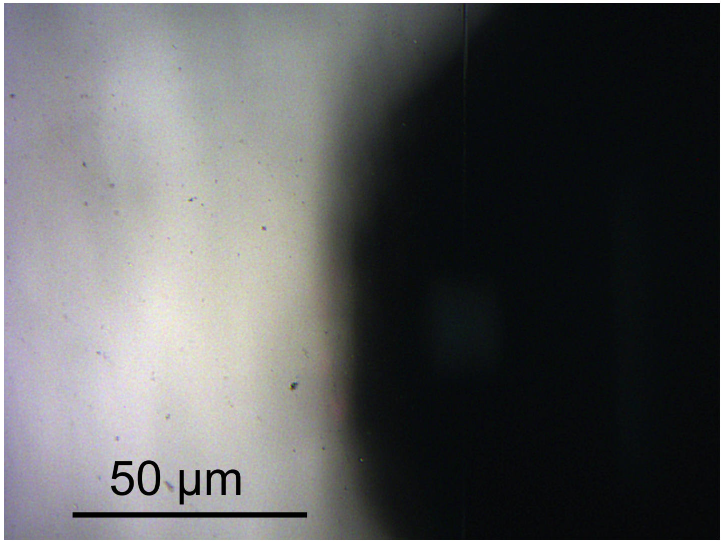

Sample

Figure S104 – Glue on glass surface image 96, 2017/12/18

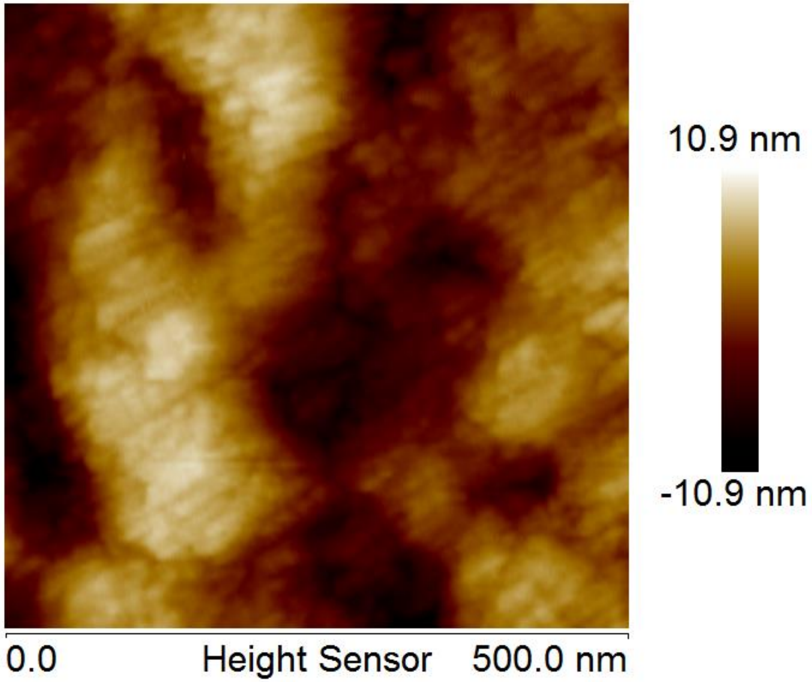

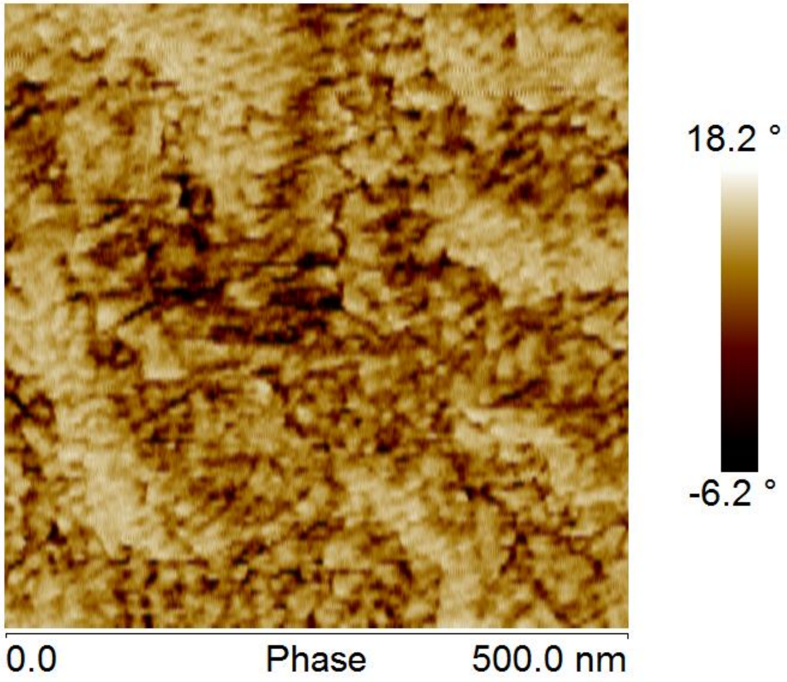

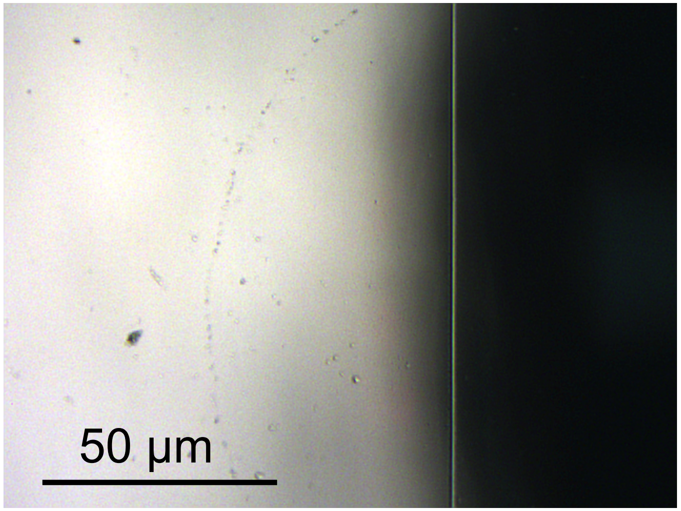

Sample

Figure S105 – Glass surface image 97, 2018/01/26

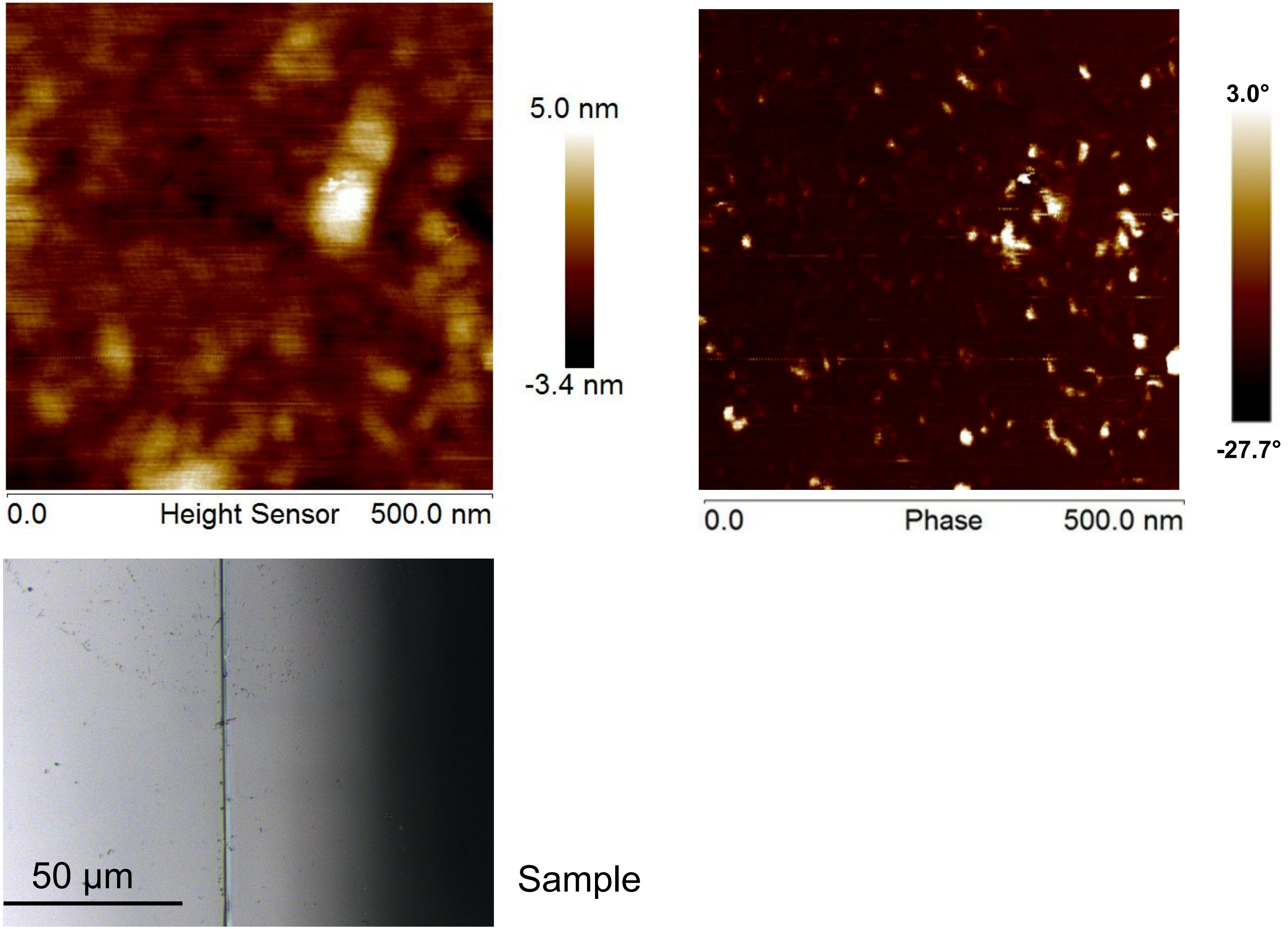

Figure S106 – Glue image 98, 2017/12/18

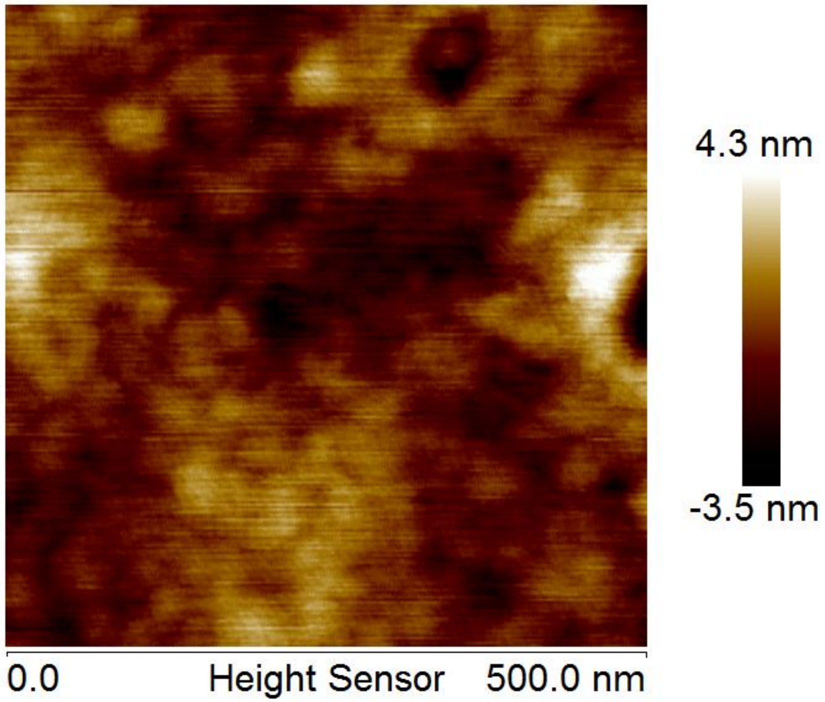

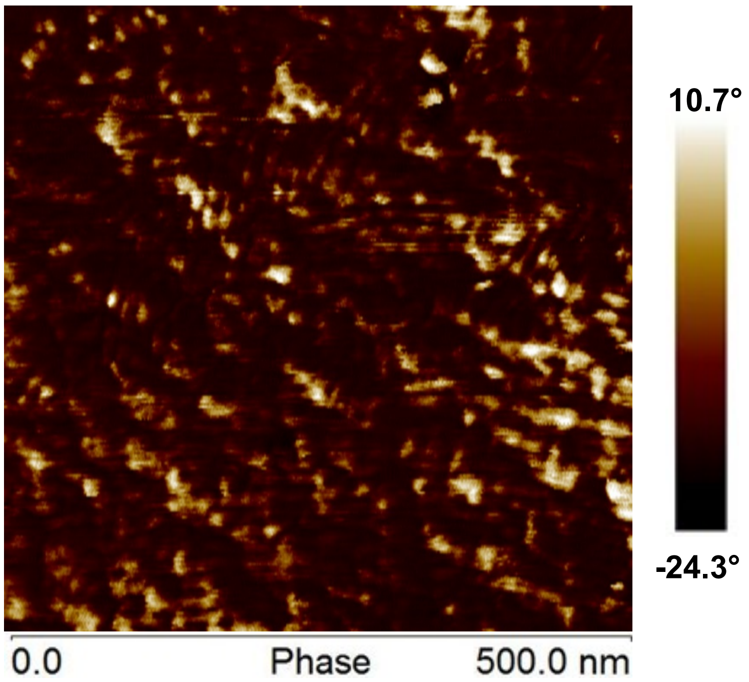

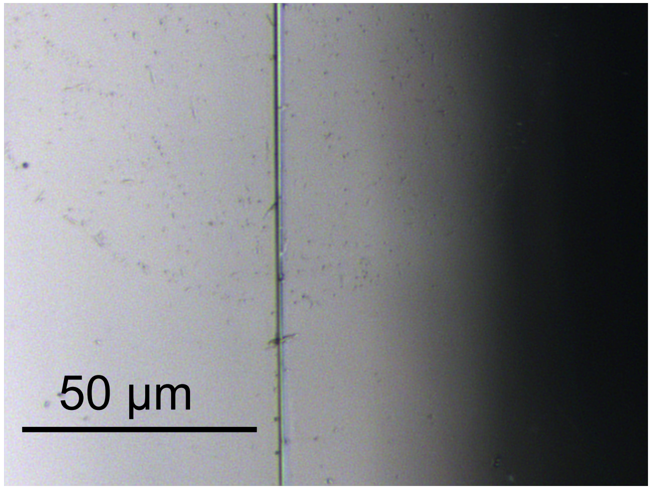

Sample
